# Reductive dearomative arylcarboxylation of indoles with CO_2_ via visible-light photoredox catalysis

**DOI:** 10.1038/s41467-020-17085-9

**Published:** 2020-06-29

**Authors:** Wen-Jun Zhou, Zhe-Hao Wang, Li-Li Liao, Yuan-Xu Jiang, Ke-Gong Cao, Tao Ju, Yiwen Li, Guang-Mei Cao, Da-Gang Yu

**Affiliations:** 1 Key Laboratory of Green Chemistry & Technology of Ministry of Education, College of Chemistry, Sichuan University, Chengdu, 610064 China; 2 College of Chemistry and Chemical Engineering, Neijiang Normal University, Neijiang, 641100 China; 3 College of Polymer Science and Engineering, State Key Laboratory of Polymer Materials Engineering, Sichuan University, Chengdu, 610065 China; 4 Shanghai Key Laboratory of Green Chemistry and Chemical Processes, East China Normal University, School of Chemistry and Molecular Engineering, Shanghai, 200062 China

**Keywords:** Photocatalysis, Sustainability, Synthetic chemistry methodology

## Abstract

Catalytic reductive coupling of two electrophiles and one unsaturated bond represents an economic and efficient way to construct complex skeletons, which is dominated by transition-metal catalysis via two electron transfer. Herein, we report a strategy of visible-light photoredox-catalyzed successive single electron transfer, realizing dearomative arylcarboxylation of indoles with CO_2_. This strategy avoids common side reactions in transition-metal catalysis, including ipso-carboxylation of aryl halides and β-hydride elimination. This visible-light photoredox catalysis shows high chemoselectivity, low loading of photocatalyst, mild reaction conditions (room temperature, 1 atm) and good functional group tolerance, providing great potential for the synthesis of valuable but difficultly accessible indoline-3-carboxylic acids. Mechanistic studies indicate that the benzylic radicals and anions might be generated as the key intermediates, thus providing a direction for reductive couplings with other electrophiles, including D_2_O and aldehyde.

## Abstract (web summary)

Catalytic reductive coupling of two electrophiles and one C = C bond is usually performed by two electron transfer metal catalysis. Herein, the authors show a visible light photoredox-catalyzed successive single electron transfer leading to dearomative arylcarboxylation of indoles with CO_2_ and generating indoline-3-carboxylic acids.

## Introduction

**Fig. 1:**
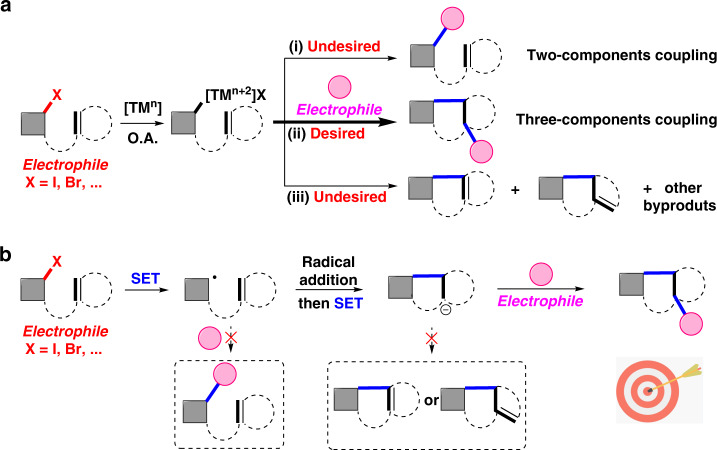
Strategies for tandem reductive couplings. **a** Widely investigated transition-metal-catalyzed reductive coupling via two electron transfer process. (i) ipso reductive coupling, (ii) tandem reductive cyclization/coupling, (iii) β-hydride elimination or isomerization as other side reactions. **b** Rarely investigated radical-type reductive coupling via successive single-electron transfer process. O.A. oxidative addition, SET single-electron transfer. The gray square represents common organic structure. The pink circle represents organic electrophiles.

Catalytic reductive coupling of two different electrophiles (or cross-electrophiles coupling) has emerged as a powerful strategy to form C–C bonds^1^. Compared with the conventional cross-coupling, transition-metal (TM)-catalyzed reductive coupling streamlines the preparation and handling of air- and moisture-sensitive organometallic reagents, thus showing significant advantages, including easily available substrates, operational simplicity, and high-step economy (Fig. 1a, path i)^2,3^. Recently, great achievement has been realized in the three-component reductive coupling, that is, difunctionalization of one unsaturated bond with two electrophiles, which could rapidly generate highly functionalized skeletons by forming two new bonds (Fig. 1a, path ii)^4–9^. Notably, if one electrophile is tethered with the unsaturated bond, a ring could be constructed via intramolecular cyclization^7–9^. Although the transition-metal catalysis with two-electron transfer process (Fig. 1a) is powerful in tuning the reactivity and selectivity, there are still some challenges in the tandem reductive cyclization/cross-coupling. For example, the ipso-functionalization (Fig. 1a, path i), that is, direct two-component coupling, would be favored if the unsaturated bond is not reactive and migratory insertion step is not fast enough. Moreover, the generated organometallic intermediate could undergo other side reactions, including protonation, β-hydride elimination, or isomerization (Fig. 1a, path iii)^10,11^. Therefore, it is highly desirable to develop other strategies, such as successive single-electron transfer (SSET) strategy (Fig. 1b), to prevent side reactions and realize such transformations with high selectivity.

**Fig. 2:**
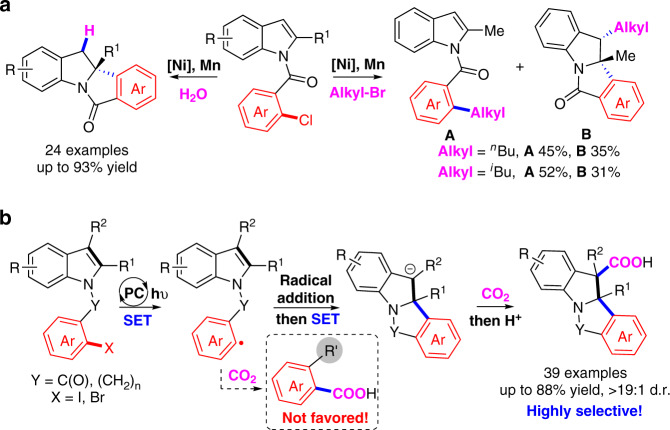
Reductive dearomative difunctionalization of indoles. **a** Tandem Ni-catalyzed asymmetric reductive dearomative functionalization of indoles by using proton or alkyl bromides as another electrophile. **b** Visible-light-promoted reductive dearomative arylcarboxylation of indoles with CO_2_ via SSET process, in which carbon centered benzylic radical and benzylic anion as key intermediate. PC photocatalyst.

Dearomatization of (hetero)arenes is an efficient and powerful method to provide 3D cyclic skeletons, which exist widely in pharmaceuticals and bioactive natural products^12–16^. Dearomative functionalization of indoles are particularly interesting to generate important indolines^15,16^. Although many methods have been developed in this field^17–25^, the dearomative reductive coupling of indoles with two electrophiles is still challenging due to the stability of carbon–carbon double bond within aromaticity and thus slow migratory insertion rate. In 2017, Qin et al. realized an elegant Ni-catalyzed asymmetric reductive hydroarylation of carbon–carbon double bond in indoles with water as proton source (Fig. 2a)^21^. When alkyl bromides were used as quenching electrophiles, however, the desired alkylarylation was not efficient and suffered from competitive side reaction, ipso cross-electrophiles coupling. With our continuous interest in green and sustainable synthesis with CO_2_, which is abundant, recyclable and nontoxic C-1 source, we wondered whether we could realize the dearomative arylcarboxylation of indoles with aryl halides and CO_2_ as electrophiles via dearomative process^26,27^, generating the valuable and difficultly accessible indoline-3-carboxylic acids^28,29^. However, due to the thermodynamic stability of both CO_2_ and aromaticity, it is highly challenging to prevent the ipso-carboxylation of aryl halides and realize selective dearomative carboxylation via transition-metal catalysis^30–34^. Inspired by the merit of visible-light photoredox catalysis^35–45^, especially the visible-light-driven redox-neutral difunctionalization of alkenes with CO_2_^46–49^, we envisioned that the reductive dearomative difunctionalization might be achieved via a radical relay SSET strategy (Fig. 1b). We speculated that the visible-light-induced single-electron reduction of carbon–halide bond could generate a highly active aryl radical, which would prefer to undergo radical addition to the C2–C3 double bond in indoles instead of CO_2_^50,51^ (for visible-light-induced decarboxylation to access aryl radical, in which aryl radical neither reduced to aryl anion nor trapped by carbon dioxide, see ref. ^50^; as long as radical reaction is concerned, carbon radical is hardly coupling with CO_2_ unless CO_2_ is converted to its corresponding radical anion, see ref. ^51^). The resulting benzylic radical might be further reduced via a second SET process to generate carbanion intermediate, which could react with weakly electrophilic CO_2_ to deliver the desired dearomative arylcarboxylation product (Fig. 2b).

Herein, we report the success of such SSET process via visible-light photoredox catalysis, realizing the dearomative arylcarboxylation of indoles with CO_2_ to give indoline-3-carboxylic acids with high selectivities (Fig. 2b). This reaction shows low loading of photocatalyst, generally good yields, mild reaction conditions (room temperature, 1 atm), good functional group tolerance, and broad substrate scope. Mechanistic studies indicate that the benzylic radicals and anions might be generated as the key intermediates. This strategy not only provides a direction for dearomative difunctionalization but also represents a way to realize selective tandem reductive cyclization/cross couplings, preventing the undesired two-component couplings and reductive Heck-type reactions via β-hydride elimination^10,11^.

## Results and discussion

### Optimization study

With this hypothesis in mind, we investigated the reaction of ethyl 1-(2-bromobenzoyl)-1H-indole-2-carboxylate **1** with CO_2_ under visible-light irradiation conditions (Table 1). When we used 1,2,3,5-tetrakis(carbazol-9-yl)-4,6-dicyanobenzene (4CzIPN) as a photocatalyst, Cs_2_CO_3_ as a base, *N*,*N*-diisopropylethylamine (DIPEA) as an electron donor, and DMSO as solvent (Table 1, entry 1), the desired carboxylic acid **2** was obtained in 88% isolated yield with high diastereoselectivity (d.r. >19:1). Control experiments revealed that CO_2_, visible light, photocatalyst, and reductant were all essential for this transformation (Table 1, entries 2–5). Other photocatalysts, such as Ir- and Ru-complex, gave lower yields (Table 1, entries 7 and 8). When Et_3_N was used as a reductant or 2 equiv of DIPEA was used, the yield was dropped to 75% (Table 1, entries 9 and 10), which might arise from lower efficiency for reductive quenching of the excited photocatalyst. Besides Cs_2_CO_3_, other bases, such as K_2_CO_3_ and KOPiv, were also efficient for the reaction, while the reaction with Cs_2_CO_3_ is the most efficient (Table 1, entries 1, 5, 11, and 12), which might quench the in-situ generated acidic components and stabilize the carboxylates. Moreover, the reaction could also be conducted in DMF to give the desired product in 75% yield (Table 1, entry 13). Compared with aryl bromide **1**, aryl iodide **1’** and chloride **1”** were also tested to give lower yields (Table 1, entries 14 and 15). In line with our expectations, not even trace of ipso-carboxylation of Ar–X (X = Cl, Br, I) bond was found in this reaction. The structure of **2** was further confirmed by X-ray crystal analysis, which indicated the cis-relationship between the newly generated two C–C bonds (please see Supplementary Information (SI) for more information).

**Table 1:** Optimization of the reaction conditions^a^.


Entry	Variation from standard conditions	Yield^b^
1	None	88%
2	Without CO_2_	n.d.
3	Without light	n.d.
4	Without 4CzIPN	n.d.
5	Without DIPEA	Trace
6	Without Cs_2_CO_3_	55%
7	Ir[(dF(Me)ppy)_2_(dtbbpy)](PF_6_) instead of 4CzIPN	78%
8	Ru(bpy)_3_(PF_6_)_2_ instead of 4CzIPN	52%
9	Et_3_N instead of DIPEA	75%
10	DIPEA (0.4 mmol, 2.0 equiv)	75%
11	K_2_CO_3_ instead of Cs_2_CO_3_	71%
12	KOPiv instead of Cs_2_CO_3_	74%
13	DMF instead of DMSO	75%
14	ArI **1’** instead of ArBr **1**	77%
15	ArCl **1”** instead of ArBr **1**	20%

*LED* light-emitting diode, *DMF* *N*,*N*-dimethylformamide, *n.d*. not detected.

^a^Reaction conditions: **1** (0.2 mmol), 4CzIPN (0.002 mmol), Cs_2_CO_3_ (0.6 mmol), DIPEA (0.6 mmol), DMSO (2 mL), 1 atm of CO_2_, 30 W blue LEDs, room temperature (RT), 24 h.

^b^Isolated yield.

### Evaluation of substrate scope

**Fig. 3:**
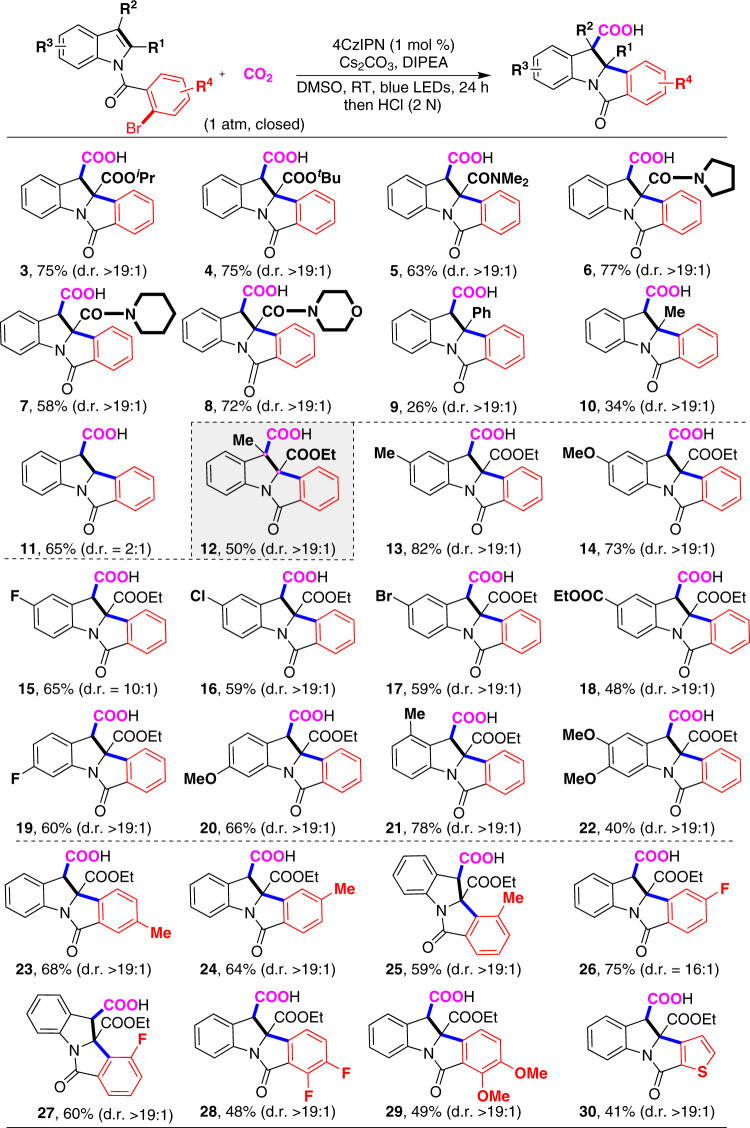
Scope of substrates with substituents on indoles. The standard reaction conditions were used, as shown in Table 1, entry 1. Isolated yields are presented.

With the optimized reaction conditions in hand, we first tested the substituent effect of the indole ring (Fig. 3). Notably, high diastereoselectivity (d.r. >19:1) was observed in most cases. We found that several esters (**2**–**4**) with different alkyl groups showed high reactivity. Moreover, a variety of amides also underwent this reaction well to give corresponding products **5**–**8** in moderate to high yields. In contrast, the substrates bearing phenyl (**9**) and electron-donating methyl group (**10**) at the C2 position, which might hamper the addition of aryl radical to indole, showed low reactivity. When no substitution was located at C2 position, the desired product **11** was obtained in 65% with poor diastereoselectivity of 2:1, which indicates that the substituention located at C2 position is necessary for high diastereoselectivity. Although all-carbon quaternary centers are difficult to synthesize due to the steric hindrance, we further challenged us using the substrate with C2- and C3 disubstitution. Different from previous transition-metal catalysis to give reductive Heck-type products^10,11^, we could obtain the desired product **12** in high chemo- and diastereoselectivity, albeit with moderate conversion, indicating potential of this strategy in organic synthesis. Encouraged by these results, we further investigated other indole derivatives. The substrates with electron-donating groups or electron-withdrawing groups at the C5-position were all suitable for such a reaction to deliver the desired products in moderate to good yields. Many kinds of functional groups, such as methoxyl (**14**), fluoro (**15**), chloro (**16**), bromo (**17**), and ester group (**18**), were all tolerated well. The substrates bearing fluoro (**19**) and methoxyl (**20**) group at C6-position also worked well. To our delight, the reaction was not sensitive to steric hindrance, as the substrate bearing methyl group at C4-position (**21**) also worked well. Notably, the substrates bearing C5- and C6-disubstitution, such as dimethoxyl groups (**22**), were also tolerated. Besides various substitutions on the indole rings, we further tested the substitution effects to the aryl bromides (R^4^). Several functional groups were tolerated at para- and meta-position. Different from previous Pd-catalysis^20^, in which the oxidative addition is sensitive to the steric hindrance, the methyl (**25**) and fluoro (**27**) groups at ortho-position of C–Br bond did not significantly affect the reaction, delivering the desired products in satisfactory yields. Moreover, difluoro (**28**) and dimethoxyl (**29**) substituents were also compatible. Notably, heterocycles, such as thiophene (**30**), which might inhibit transition-metal catalysis due to the strong coordination, could also survive in this reaction.

**Fig. 4:**
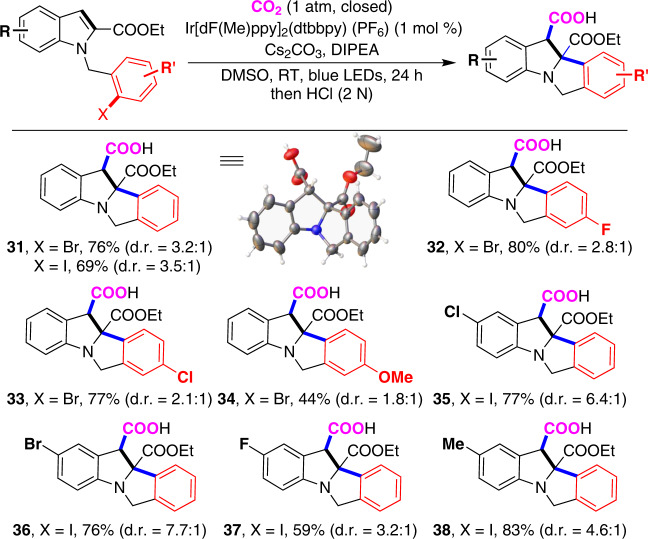
Scope of substrates bearing unactivated aryl bromides and iodides. Reaction conditions: Indole derivatives (0.2 mmol), Ir-catalyst (0.002 mmol), Cs_2_CO_3_ (0.6 mmol), DIPEA (1.3 mmol), DMSO (2 mL), 1 atm of CO_2_, 30 W blue LEDs, RT, 24 h, isolated yield.

In light of these results, we turned our attention to unactivated aryl halides, which are more electron rich and thus more challenging to undergo single-electron reduction by photocatalyst. Under slightly modified conditions (Fig. 4), a variety of aryl bromides and iodides underwent the reaction to generate desired products **31**–**38** in good yields, albeit with moderate diastereoselectivity. The single-crystal analysis of the major isomer of product **31** indicated the trans-relationship between the newly generated C–C bonds (please see Supplementary Information for more information), which is different from **2**. As shown in Fig. 4, both unactivated aryl bromides and iodides could react smoothly with good functional group tolerance. Notably, carbon−halo bonds, especially the C–Br bond (**36**), which are reactive in transition-metal catalysis, survived in our reaction conditions.

**Fig. 5:**
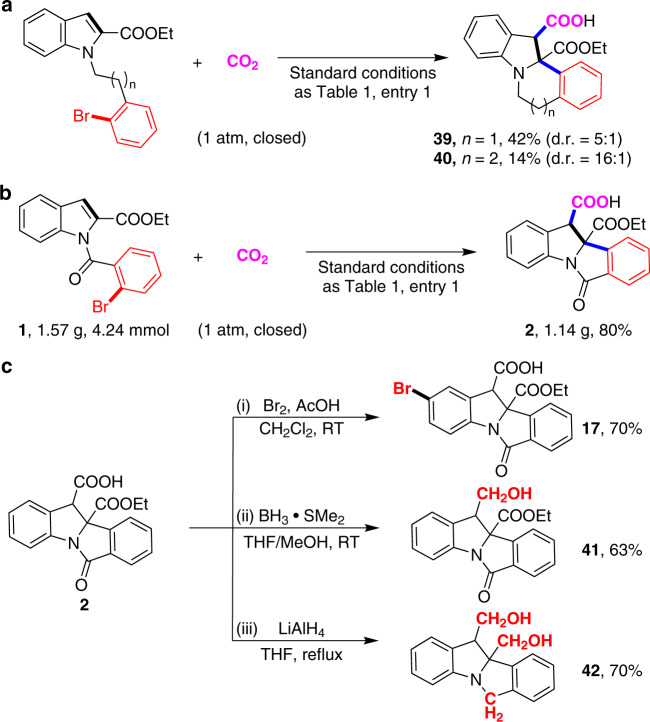
Synthetic applications. **a** Synthesis of 6-membered and 7-membered ring-bearing products under the standard conditions. **b** Gram-scale synthesis of **2**. **c** Transformations of the product **2**. (i) bromination with Br_2_ and AcOH. (ii) selective reduction of the C3-ester group by borane. (iii) reduction of amide and ester groups by LiAlH_4_.

Furthermore, 1-(2-bromophenethyl)-1H-indole-2-carboxylate and ethyl 1-(3-(2-bromophenyl)propyl)-1H-indole-2-carboxylate were tested in the standard reaction conditions (Fig. 5a) to give the corresponding products bearing 6-membered **39** and 7-membered ring **40** in 42% and 14% isolated yields, respectively, demonstrating that this strategy is not restricted to 5-membered ring formation. To further demonstrate the utility of this dearomative arylcarboxylation, we conducted a gram-scale reaction of **1** under standard conditions, which is also smooth to generate **2** in 80% yield (Fig. 5b). Moreover, facile and selective derivatizations, such as bromination and reduction, of carboxylic acid **2** provide easy access to valuable motifs (Fig. 5c).

### Proposed mechanism and mechanistic studies

**Fig. 6:**
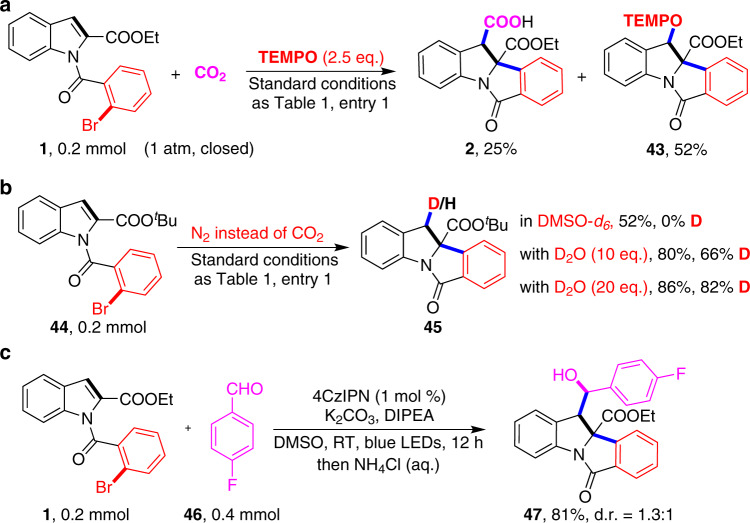
Preliminary mechanistic studies. **a** Trapping experiment by radical scavenger 2,2,6,6-tetramethyl-piperidinyloxyl (TEMPO), supporting that radical process might be involved. **b** Isotopic labeling experiments in DMSO-*d*_6_ or in the presence of different amounts of deuterated water, suggesting that benzylic anion was involved. **c** 4-fluorobenzaldehyde was used as an electrophile instead of CO_2_, further indirectly confirming the formation of benzylic anion intermediate.

To gain more insight into the reaction mechanism, we further did a variety of control experiments (please see Supplementary Figs. 1–9 for more information). First, we found that the desired reaction of **1** was significantly inhibited by 2,2,6,6-tetramethyl-1-piperdinyloxy (TEMPO). Meanwhile, a TEMPO-radical adduct **43** was isolated, indicating that benzylic radical was involved in the reaction (Fig. 6a). As transient aryl radical can be trapped by TEMPO^52^, the fact that no trapping of the aryl radical with TEMPO indicated that the radical cyclization step might be fast. Second, we did isotope-labeling studies (Fig. 6b). When DMSO-*d*_*6*_ was used as solvent in the reaction of **44**, no deuterium incorporation in the hydroarylation product **45** was observed, thus ruling out the possibility of hydrogen-atom transfer with DMSO^53^. We found a 82% deuterium incorporation at C3-position of **45** when 20 equivalent of D_2_O was added under N_2_ atmosphere, indicating the formation of benzylic anion intermediate. Furthermore, with slightly modified the reaction conditions, 4-fluorobenzaldehyde **46** was applied as electrophile instead of CO_2_ to deliver the desired product **47** in 81% yield (Fig. 6c), further confirming that the benzylic anion might be involved in the reaction.

**Fig. 7:**
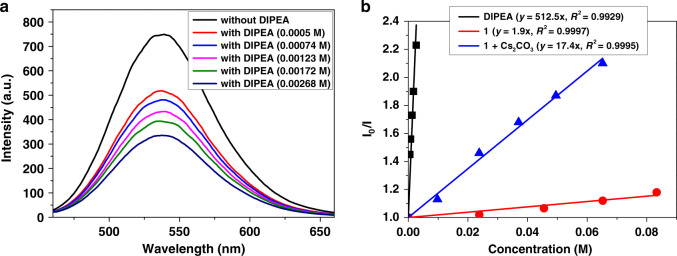
Optical experiment with fluorescence spectrum. **a** Steady-state Stern–Volmer experiment of 4CzIPN and DIPEA, the luminescence of 4CzIPN was readily quenched by DIPEA. **b** Stern–Volmer fluorescence quenching experiments using 4CzIPN with DIPEA, **1** as well as **1** and Cs_2_CO_3_.

Moreover, Stern–Volmer fluorescence quenching experiments were performed (Fig. 7, please see Supplementary Fig. 10 for more information). The luminescence of 4CzIPN at *λ*_max_ = 536 nm was readily quenched by DIPEA with a slope of 512.5, which was much more significant than indole **1** (1.9) or the combination of indole **1** and Cs_2_CO_3_ (17.4). These results suggested that DIPEA had the priority to undergo SET with excited 4CzIPN.

**Fig. 8:**
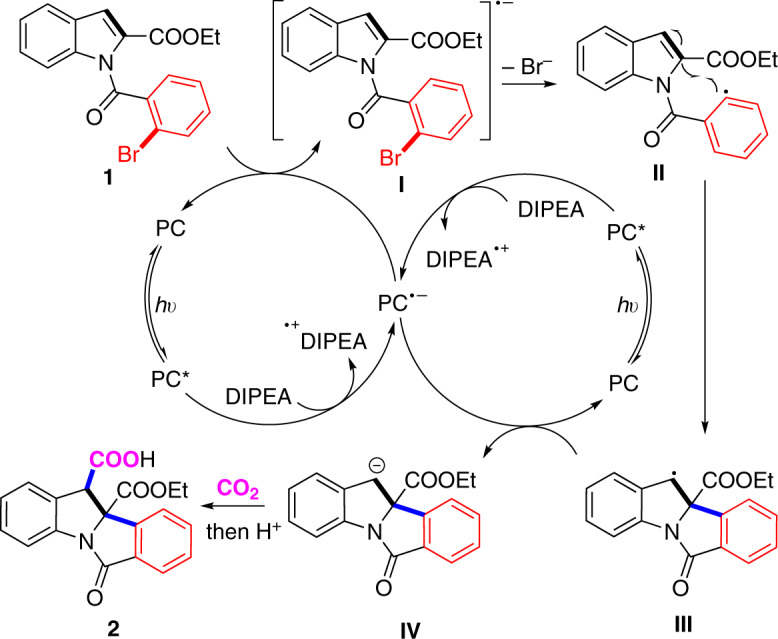
Mechanistic proposal for the arylcarboxylation of 1. PC = 4CzIPN.

Based on above-mentioned results and previous work, we proposed the possible mechanism for the reaction of **1** (Fig. 8). The reductive quenching of photo-activated 4CzIPN (E_1/2_ [4CzIPN/4CzIPN^•−^] = −1.21 V vs. SCE in MeCN)^54^ by DIPEA (E_1/2_^Ox^ = +0.63 V vs. SCE in DMF)^55^ leads to the corresponding radical anion 4CzIPN^•−^ and the radical cation DIPEA^•+^. The reduced 4CzIPN^•−^ undergoes a single-electron reduction of aryl bromide (ArBr) **1**, producing the [ArBr]^•−^ radical anion **I** and regenerating the neutral 4CzIPN to complete one catalytic cycle. Then, the generated [ArBr]^•−^ radical anion undergoes fragmentation to release a bromide anion and an aryl radical **II**^56,57^, which undergoes facile intramolecular radical addition to the C2–C3 double bond of indole to afford the benzylic radical **III**. The following SET with 4CzIPN^•−^ delivers the carbon anion intermediate **IV**^58,59^, which could undergo nucleophilic addition to CO_2_^60–63^. Following protonation provides the dearomative arylcarboxylation product **2**. However, at this stage we could not exclude another possibility that **I** undergoes radical cyclization via C–C bond formation and following re-aromatization via release of bromide anion to give **III**. Further experimental and computational investigations are necessary to explore more details for this process.

In conclusion, we report a strategy of successive single-electron transfer (SSET) process for the tandem reductive cyclization/cross couplings, realizing the first dearomative arylcarboxylation of indoles with CO_2_ via visible-light photoredox catalysis^64^ (during the revision of this paper, Li reported an elegant and very similar visible-light photoredox-catalyzed reductive arylcarboxylation of styrenes, see ref. ^64^). Notably, this reaction is highly chemoselective, as common side reactions, such as ipso coupling of aryl halides and β-hydride elimination, are avoided. This reaction shows high selectivity, low loading of photocatalyst, generally good yields, mild reaction conditions (room temperature, 1 atm), good functional group tolerance and broad substrate scope, providing great potential for the synthesis of valuable but difficultly accessible indoline-3-carboxylic acids. Mechanistic studies indicate that the benzylic radicals and anions might be generated as the key intermediates. Further application of this strategy in other reductive couplings with diverse electrophiles is underway in our laboratory.

## Methods

### General procedure for the synthesis of 2–30

An oven-dried Schlenk tube (10 mL) containing a stirring bar was charged with the indole derivative (0.2 mmol) and 4CzIPN (1 mol %). Subsequently, the Schlenk tube was introduced in a glovebox and was charged with Cs_2_CO_3_ (0.6 mmol). The tube was sealed and evacuated and back-filled with carbon dioxide three times. Then DMSO (2 mL) and DIPEA (0.6 mmol) were added under CO_2_ flow. Once added, the resulting mixture in sealed tube was placed at a distance of 2–4 cm from a 30-W blue LED and stirred at room temperature (25 °C) for 24 h. Then, the mixture was quenched with 1 mL of H_2_O and 2 mL of HCl (2 N), extracted with EtOAc, the combined organic phases were concentrated in vacuo. The residue was purified by silica gel flash chromatography (0.1% AcOH in petroleum ether/EtOAc) to give the corresponding desired product.

### General procedure for the synthesis of Compound 31–38

An oven-dried Schlenk tube (10 mL) containing a stirring bar was charged with the substrate (0.2 mmol) and Ir[dF(Me)ppy]_2_(dtbbpy)(PF_6_) (1 mol %) subsequently. Then, the Schlenk tube was then introduced in a glovebox, where it was charged with Cs_2_CO_3_ (0.6 mmol). The tube was taken out of the glovebox and connected to a vacuum line where it was evacuated and back-filled with CO_2_ three times. Then DMSO (2 mL) and DIPEA (1.3 mmol) were added under CO_2_ flow. Finally, The reaction mixture in sealed tube was placed at a distance of 2–4 cm from a 30-W blue LED and stirred at room temperature (25 °C) for 24 h. Then, the mixture was quenched with 1 mL of H_2_O and 2 mL of HCl (2 N), extracted with EtOAc, then concentrated in vacuo. The residue was purified by silica gel flash chromatography (0.1% AcOH in petroleum ether/EtOAc) to give the corresponding desired product.

## Supplementary information

Supplementary Information

## Data Availability

The authors declare that the data supporting the findings of this study are available within the article and its Supplementary Information files. Extra data are available from the author upon request. The crystallography data have been deposited at the Cambridge Crystallographic Data Center (CCDC) under accession number CCDC: 1950734 (compound **2**) and CCDC: 1972327 (compound **31**) can be obtained free of charge from www.ccdc.cam.ac.uk/getstructures.
