# Supplementary material for: Reductive dearomative arylcarboxylation of indoles with CO2 via visible-light photoredox catalysis
Source: Nat Commun. 2020 Jun 29;11:3263. doi: 10.1038/s41467-020-17085-9 (PMC7324572; doi:10.1038/s41467-020-17085-9)
Supplement: Supplementary file 1 — Supplementary Information [file 41467_2020_17085_MOESM1_ESM.pdf]

Supplementary Information

for

Reductive Dearomative Arylcarboxylation of Indoles with CO_2 via Visible-Light Photoredox Catalysis

Zhou et al.

Supplementary Methods

General Information

All reactions were set up with glovebox and carried out under a carbon dioxide atmosphere in Schlenk tubes. Anhydrous solvent (including DMSO, 99.8%, Water< 0.005%) were purchased from J&K, and used as received. Commercially available compounds were obtained from J&K, Accela, Adamas, Across, TCI and used as received unless otherwise stated.

^{1}H and ^{13}C NMR spectra were recorded on a Bruker Advance 400 spectrometer (^{1}H: 400 MHz, ^{13}C: 101 MHz, ^{19}F: 376 MHz). Chemical shifts (δ) for ^{1}H and ^{13}C NMR spectra are given in ppm relative to TMS, The residual solvent signals were used as references for ^{1}H and ^{13}C NMR spectra and the chemical shifts converted to the TMS scale ($CDCl_3$: δH = 7.26 ppm, δC = 77.16 ppm; DMSO-d_6: δH = 2.50 ppm, δC = 39.52 ppm). The following abbreviations were used to explain the multiplicities: s = singlet, d = doublet, t = triplet, q = quartet, m = multiplet, br = broad.

TLC was performed using commercially prepared 100-400 mesh silica gel plates (GF254), and visualization was effected at 254 nm. Exact ESI mass spectra were recorded on a SHIMADZU LCMS-IT-TOF. ESI-MS were obtained on a Thermo-LTQ mass spectrometer.

The synthesis of substrate 1

ethyl 1-(2-bromobenzoyl)-1H-indole-2-carboxylates used in this work were prepared according to the methods reported in literature.[1,2] ethyl 1-(2-iodobenzyl)-1H-indole-2-carboxylate used in this work were prepared according to the methods reported in literature.[3]

General procedure for the synthesis of indolines 2-30

An oven-dried Schlenk tube (10 mL) containing a stirring bar was charged with the substrate (0.2 mmol) and 4CzIPN (0.002 mmol, 1.6 mg, 1 mol %) subsequently. the Schlenk tube was then introduced in a glovebox, where it was charged with Cs_2CO_3 (0.6 mmol, 195 mg, 3.0 equiv). The tube was taken out of the glovebox and connected to a vacuum line where it was evacuated and back-filled with CO_2 for 3 times. Then DMSO (2 mL) and DIPEA (0.6 mmol, 100 μL, 3.0 equiv) were added under CO_2 flow. Finally, The reaction mixture in sealed tube was placed at a distance of 2 ~ 4 cm from a 30 W blue LED and stirred at room temperature (25 ℃) for 24 h. Then, the mixture was quenched with 1 mL of H_2O and 2 mL of HCl (2 N), extracted with EtOAc, then concentrated in vacuo. The residue was purified by silica gel flash

chromatography (0.1% AcOH in petroleum ether/EtOAc) to give the corresponding desired product.

General procedure for the synthesis of Compound 31-38

An oven-dried Schlenk tube (10 mL) containing a stirring bar was charged with the substrate (0.2 mmol) and Ir[dF(Me)ppy]$_2$(dtbbpy)(PF$_6$) (0.002 mmol, 2.1 mg, 1 mol %) subsequently. the Schlenk tube was then introduced in a glovebox, where it was charged with Cs_2CO_3 (0.6 mmol, 195 mg, 3.0 equiv). The tube was taken out of the glovebox and connected to a vacuum line where it was evacuated and back-filled with CO_2 for 3 times. Then DMSO (2 mL) and DIPEA (1.3 mmol, 217 μL, 6.5 equiv) were added under CO_2 flow. Finally, The reaction mixture in sealed tube was placed at a distance of 2 ~ 4 cm from a 30 W blue LED and stirred at room temperature (25 ℃) for 24 h. Then, the mixture was quenched with 1 mL of H_2O and 2 mL of HCl (2 N), extracted with EtOAc, then concentrated in vacuo. The residue was purified by silica gel flash chromatography (0.1% AcOH in petroleum ether/EtOAc) to give the corresponding desired product.

Gram-scale experiment

The oven-dried Schlenk tube (250 mL) containing a stirring bar was charged with ethyl 1-(2-bromobenzoyl)-1H-indole-2-carboxylate **1a** (1.57 g, 4.24 mmol), 4CzIPN (0.0424 mmol, 33.4 mg, 1 mol %), the Schlenk tube was then introduced in a glovebox, where it was charged with Cs_2CO_3 (12.72 mmol, 4.13 g, 3.0 equiv). The tube was taken out of the glovebox and connected to a vacuum line where it was evacuated and back-filled with CO_2 for 3 times. Then, anhydrous DMSO (42 mL) and DIPEA (12.72 mmol, 2.1 mL, 3.0 equiv) were added under CO_2 flow. The reaction mixture in sealed tube was placed at a distance of 2 ~ 4 cm from a 30 W blue LED and stirred at room temperature (25 ^{o}C) for 24 h. Then, The mixture was quenched with 20 mL of H_2O and 40 mL of HCl (2 N), extracted with EtOAc, then concentrated in vacuo. The residue was purified by silica gel flash chromatography (0.1% AcOH in petroleum ether/EtOAc = 3:1) to give **2** in 80% yield (1.14 g).

10b-(ethoxycarbonyl)-6-oxo-10b,11-dihydro-6H-isoindolo[2,1-a]indole-11-carboxylic acid (2)

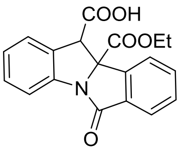

Purification by flash chromatography on silica gel (0.1% AcOH in petroleum ether/EtOAc V/V = 3:1) gave as white solid (59.3 mg, 0.18 mmol, 88% yield);

mp: 194–196 ^{o}C;

$\boldsymbol{R}_{\mathbf{f}}$ = 0.16 (EtOAc/ petroleum ether, v/v = 2/1);

^{1}**H NMR** (400 MHz, $CDCl_3$) δ 7.71 – 7.66 (m, 2H), 7.47 – 7.40 (m, 3H), 7.34 – 7.31 (m, 1H), 7.21 – 7.19 (m, 1H), 7.08 – 7.04 (m, 1H), 4.73 (s, 1H), 4.04 (q, *J* = 7.2 Hz, 2H), 1.13 (t, *J* = 7.2 Hz, 3H);
^{13}**C NMR** (101 MHz, $CDCl_3$) δ 172.3, 169.8, 167.9, 140.9, 140.0, 133.7, 132.9, 131.9, 130.1, 130.0, 125.6, 125.1, 125.0, 123.3, 117.1, 78.5, 63.1, 52.3, 13.8;
HRMS calcd for $C_{19}H_{16}NO_5$ $[M+H]^+$: 338.1023, found for: 338.1025.

10b-(isopropoxycarbonyl)-6-oxo-10b,11-dihydro-6H-isoindolo[2,1-a]indole-11-carboxylic acid (3)

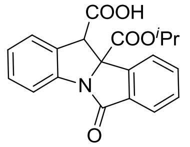

Purification by flash chromatography on silica gel (0.1% AcOH in petroleum ether/EtOAc V/V = 3:1) gave as colorless oil (52.7 mg, 0.15 mmol, 75% yield);
$\boldsymbol{R_f}$ = 0.16 (EtOAc/ petroleum ether, v/v = 2/1);
^{1}**H NMR** (400 MHz, $CDCl_3$) δ 7.77 – 7.73 (m, 2H), 7.52 – 7.46 (m, 3H), 7.41 – 7.37 (m, 1H), 7.27 – 7.25 (m, 2H), 7.14 – 7.10 (m, 1H), 4.98 – 4.88 (m, 1H), 4.76 (s, 1H), 1.09 (dd, *J* = 11.2, 6.4 Hz, 6H);
^{13}**C NMR** (101 MHz, $CDCl_3$) δ 172.1, 169.1, 168.0, 141.0, 139.9, 133.7, 132.8, 132.0, 130.0, 129.9, 125.5, 125.0, 124.9, 123.2, 117.1, 78.6, 71.1, 52.3, 21.3, 21.2;
HRMS calcd for $C_{19}H_{16}NO_3$ $[M-COOH]^-$: 306.1136, found for: 306.1135.

10b-(tert-butoxycarbonyl)-6-oxo-10b,11-dihydro-6H-isoindolo[2,1-a]indole-11-carboxylic acid (4)

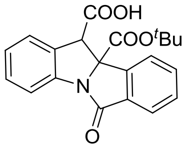

Purification by flash chromatography on silica gel (0.1% AcOH in petroleum ether/EtOAc V/V = 3:1) gave as colorless oil (54.8 mg, 0.15 mmol, 75% yield);
$\boldsymbol{R_f}$ = 0.17 (EtOAc/ petroleum ether, v/v = 2/1);
^{1}**H NMR** (400 MHz, $CDCl_3$) δ 7.78–7.74 (m, 2H), 7.55–7.47 (m, 3H), 7.39–7.38 (m, 1H), 7.29–7.27 (m, 1H), 7.14–7.10 (m, 1H), 4.74 (s, 1H), 1.32 (s, 9H);
^{13}**C NMR** (101 MHz, $CDCl_3$) δ 171.9, 168.3, 167.9, 141.4, 140.1, 133.9, 132.7, 132.2, 129.9, 129.8, 125.5, 124.9, 124.8, 123.3, 117.0, 84.0, 79.0, 52.4, 27.6;
HRMS calcd for $C_{21}H_{19}NNaO_5$ $[M+Na]^+$: 388.1155, found for: 388.1162.

10b-(dimethylcarbamoyl)-6-oxo-10b,11-dihydro-6H-isoindolo[2,1-a]indole-11-carboxylic acid (5)

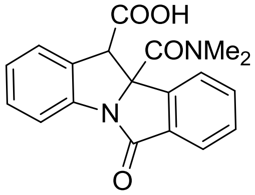

Purification by flash chromatography on silica gel (0.1% AcOH in petroleum ether/EtOAc V/V = 3:1) gave as colorless oil (42.3 mg, 0.13 mmol, 63% yield);
$\boldsymbol{R_f}$ = 0.14 (EtOAc/ petroleum ether, v/v = 2/1);
^{1}**H NMR** (400 MHz, DMSO-d_6) δ 7.84–7.82 (m, 1H), 7.77–7.73 (m, 1H), 7.66–7.58 (m, 3H), 7.42–7.38 (m, 2H), 7.20–7.16 (m, 1H), 5.18 (s, 1H), 2.78 (s, 6H);
^{13}**C NMR** (101 MHz, $CDCl_3$) δ 172.6, 169.3, 168.0, 141.7, 139.6, 134.0, 133.5, 133.1, 130.0, 129.3, 125.8, 125.5, 124.4, 123.8, 117.2, 80.8, 54.1, 39.9, 37.7;
HRMS calcd for $C_{19}H_{16}N_2NaO_4$ $[M+Na]^+$: 359.1002, found for: 359.1008.

6-oxo-10b-(pyrrolidine-1-carbonyl)-10b,11-dihydro-6H-isoindolo[2,1-a]indole-11-carboxylic acid (6)

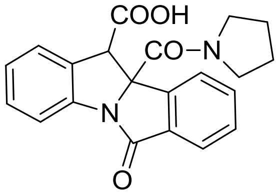

Purification by flash chromatography on silica gel (0.1% AcOH in petroleum ether/EtOAc V/V = 4:1) gave as colorless oil (55.7 mg, 0.15 mmol, 77% yield);
$\boldsymbol{R_f}$ = 0.30 (EtOAc/ petroleum ether, v/v = 2/1);
^{1}**H NMR** (400 MHz, $CDCl_3$) δ 7.77–7.75 (m, 1H), 7.68–7.67 (m, 1H), 7.57–7.43 (m, 3H), 7.39–7.35 (m, 1H), 7.29–7.27 (m, 1H), 7.17–7.13 (m, 1H),

5.24 (s, 1H), 3.48–3.42 (m, 1H), 3.38–3.32 (m, 1H), 3.29–3.23 (m, 1H), 2.99–2.93 (m, 1H), 1.82–1.75 (m, 1H), 1.65–1.50 (m, 3H) ppm;

^{13}C NMR (100 MHz, $CDCl_3$) δ 172.4, 169.1, 166.8, 141.6, 139.7, 133.7, 133.5, 133.2, 129.9, 129.4, 125.9, 125.4, 124.3, 123.9, 116.9, 80.4, 53.6, 49.4, 46.7, 26.9, 22.6;

HRMS calcd for $C_{21}H_{18}N_2NaO_4$ $[M+Na]^+$: 385.1159, found for: 385.1160.

7-oxo-10b-(piperidine-1-carbonyl)-10b,11-dihydro-6H-isoindolo[2,1-a]indole-11-carboxylic acid (7)

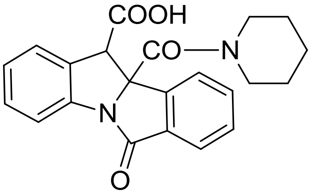

Purification by flash chromatography on silica gel (0.1% AcOH in petroleum ether/EtOAc V/V = 3:1) gave as colorless oil (43.6 mg, 0.12 mmol, 58% yield);

***R*$_f$** = 0.20 (EtOAc/ petroleum ether, v/v = 2/1);

^{1}H NMR (400 MHz, $CDCl_3$) δ 7.80–7.78 (m, 1H), 7.67–7.65 (m, 1H), 7.55–7.54 (m, 2H), 7.50–7.47 (m, 1H), 7.40–7.36 (m, 1H), 7.29–7.27 (m, 1H), 7.17–7.14 (m, 1H), 5.44 (s, 1H), 3.39–3,26 (m, 4H), 1.43–1,40 (m, 2H), 1.27–1.24 (m, 4H);

^{13}C NMR (101 MHz, $CDCl_3$) δ 172.9, 169.7, 166.0, 142.5, 139.8, 134.1, 133.3, 132.7, 129.9, 129.3, 125.6, 125.5, 124.7, 123.8, 117.6, 81.2, 54.1, 46.8, 25.8, 24.1;

HRMS calcd for $C_{22}H_{20}N_2NaO_4$ $[M+Na]^+$: 399.1315, found for: 399.1322.

10b-(morpholine-4-carbonyl)-6-oxo-10b,11-dihydro-6H-isoindolo[2,1-a]indole-11-carboxylic acid (8)

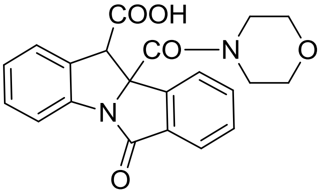

Purification by flash chromatography on silica gel (0.1% AcOH in petroleum ether/EtOAc V/V = 3:1) gave as colorless oil (54.4 mg, 0.14 mmol, 72% yield);

***R*$_f$** = 0.15 (EtOAc/ petroleum ether, v/v = 2/1);

^{1}H NMR (400 MHz, $CDCl_3$) δ 7.80–7.78 (m, 1H), 7.66–7.64 (m, 1H), 7.57–7.56 (m, 2H), 7.52–7.48 (m, 1H), 7.42–7.38 (m, 1H), 7.30–7.28 (m, 1H), 7.20–7.16 (m, 1H), 5.39 (s, 1H), 3.56–3.51(m, 2H), 3.33 (br, 6H);

^{13}C NMR (101 MHz, $CDCl_3$) δ 172.3, 169.6, 166.7, 142.3, 139.8, 134.0, 133.6, 132.7, 130.2, 129.6, 125.8, 125.7, 124.9, 123.8, 117.7, 81.1, 66.5, 54.2, 45.8;

HRMS calcd for $C_{21}H_{18}N_2NaO_5$ $[M+Na]^+$: 401.1108, found for: 401.1115.

6-oxo-10b-phenyl-10b,11-dihydro-6H-isoindolo[2,1-a]indole-11-carboxylic acid (9)

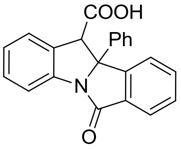

Purification by flash chromatography on silica gel (0.1% AcOH in petroleum ether/EtOAc V/V = 3:1) gave as colorless oil (17.6 mg, 0.05 mmol, 26% yield);

***R*$_f$** = 0.16 (EtOAc/ petroleum ether, v/v = 2/1);

^{1}H NMR (400 MHz, DMSO-d_6) δ 7.79 – 7.74 (m, 2H), 7.70 – 7.67 (m, 3H), 7.64 (td, J = 7.2, 1.2 Hz, 1H), 7.51 (td, J = 7.2, 0.8 Hz, 1H), 7.42 – 7.33 (m, 4H), 7.28 – 7.25 (m, 1H), 7.14 – 7.10 (m, 1H), 4.62 (s, 1H);

^{13}C NMR (101 MHz, DMSO-d_6) δ 170.5, 167.6, 147.2, 142.3, 139.9, 134.7, 133.1, 132.0, 129.1, 129.0, 128.1, 126.0, 125.0, 124.9, 124.2, 124.1, 116.8, 78.8, 57.2;

HRMS calcd for $C_{22}H_{15}NNaO_3$ $[M+Na]^+$: 364.0950, found for: 364.0950.

10b-methyl-6-oxo-10b,11-dihydro-6H-isoindolo[2,1-a]indole-11-carboxylic acid (10)

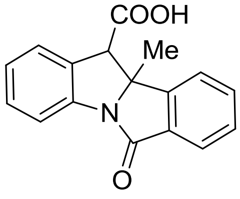

Purification by flash chromatography on silica gel (0.1% AcOH in petroleum ether/EtOAc V/V = 3:1) gave as colorless oil (19.0 mg, 0.07 mmol, 34% yield);

R_f = 0.22 (EtOAc/ petroleum ether, v/v = 2/1);
^{1}H NMR (400 MHz, $CDCl_3$) δ 7.76–7.70 (m, 2H), 7.52–7.49 (m, 1H), 7.45–7.39 (m, 3H), 7.29–7.26 (m, 1H), 7.16–7.12 (m, 1H), 3.96 (s, 1H), 1.65 (s, 3H);
^{13}C NMR (101 MHz, $CDCl_3$) δ 172.9, 168.1, 147.4, 139.7, 133.3, 133.0, 132.7, 129.7, 129.0, 125.9, 124.8, 124.7, 122.4, 117.7, 73.9, 55.7, 27.8;
HRMS calcd for $C_{16}H_{12}NO$ $[M\text{-}COOH]^-$: 234.0924, found for: 234.0926.

6-oxo-10b,11-dihydro-6H-isoindolo[2,1-a]indole-11-carboxylic acid (11)

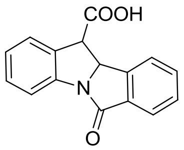

Purification by flash chromatography on silica gel (0.1% AcOH in petroleum ether/EtOAc V/V = 3:1) gave as colorless oil (34.5 mg, 0.13 mmol, 65% yield);
R_f = 0.17 (EtOAc/ petroleum ether, v/v = 2/1);
^{1}H NMR (400 MHz, DMSO-d_6) δ 7.77 – 7.75 (m, 1H), 7.72 – 7.64 (m, 2H), 7.58 – 7.54 (m, 2H), 7.41 – 7.36 (m, 2H), 7.16 – 7.12 (m, 1H), 5.91 (d, J = 8.0 Hz, 1H), 4.43 (d, J = 8.0 Hz, 1H);
^{13}C NMR (101 MHz, DMSO-d_6) δ 170.6, 167.6, 143.4, 140.6, 135.5, 134.0, 132.6, 128.9, 128.8, 125.9, 124.5, 124.1, 123.8, 116.0, 66.8, 48.8;
HRMS calcd for $C_{16}H_{12}NO_3$ $[M+H]^+$: 266.0817, found for: 266.0820.

10b-(ethoxycarbonyl)-11-methyl-6-oxo-10b,11-dihydro-6H-isoindolo[2,1-a]indole-11-carboxylic acid (12)

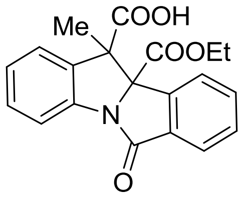

Purification by flash chromatography on silica gel (0.1% AcOH in petroleum ether/EtOAc V/V = 3:1) gave as colorless oil (35.1 mg, 0.10 mmol, 50% yield);
R_f = 0.28 (EtOAc/ petroleum ether, v/v = 2/1);
^{1}H NMR (400 MHz, DMSO-d_6) δ 7.83–7.81 (m, 1H), 7.76–7.71 (m, 2H), 7.63–7.55 (m, 2H), 7.40–7.29 (m, 2H), 7.17–7.13 (m, 1H), 4.12 (q, J = 7.2 Hz, 2H), 1.73 (s, 3H), 1.13 (t, J = 7.2 Hz, 3H);
^{13}C NMR (101 MHz, DMSO-d_6) δ 171.4, 167.8, 167.7, 142.3, 141.6, 139.8, 133.4, 133.2, 130.5, 129.3, 126.3, 125.1, 124.1, 123.9, 116.5, 82.3, 62.7, 57.4, 18.5, 14.2 ;
HRMS calcd for $C_{20}H_{17}NNaO_5$ $[M+Na]^+$: 374.0999, found for: 374.1008.

10b-(ethoxycarbonyl)-2-methyl-6-oxo-10b,11-dihydro-6H-isoindolo[2,1-a]indole-11-carboxylic acid (13)

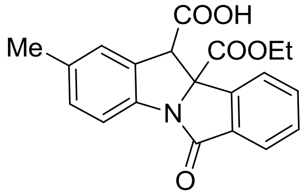

Purification by flash chromatography on silica gel (0.1% AcOH in petroleum ether/EtOAc V/V = 3:1) gave as colorless oil (57.6 mg, 0.16 mmol, 82% yield);
R_f = 0.19 (EtOAc/ petroleum ether, v/v = 2/1);
^{1}H NMR (400 MHz, $CDCl_3$) δ 7.77–7.75 (m, 1H), 7.63–7.61 (m, 1H), 7.54–7.45 (m, 3H), 7.20–7.18 (m, 1H), 7.09–7.08 (m, 1H), 4.75 (s, 1H), 4.11 (q, J = 7.2 Hz, 2H), 2.33 (s, 3H), 1.12 (t, J = 7.2 Hz, 3H);
^{13}C NMR (101 MHz, $CDCl_3$) δ 172.2, 169.9, 168.0, 140.9, 137.6, 134.9, 133.9, 132.7, 132.1, 130.4, 130.0, 126.2, 124.9, 123.2, 116.8, 78.8, 63.1, 52.4, 21.1, 13.8;
HRMS calcd for $C_{20}H_{17}NNaO_5$ $[M+Na]^+$: 374.0999, found for: 374.0994.

10b-(ethoxycarbonyl)-2-methoxy-6-oxo-10b,11-dihydro-6H-isoindolo[2,1-a]indole-11-carboxylic acid

(14)

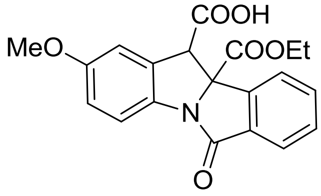

Purification by flash chromatography on silica gel (0.1% AcOH in petroleum ether/EtOAc V/V = 3:1) gave as white solid (53.6 mg, 0.15 mmol, 73% yield);

mp: 199–201 °C;

$\boldsymbol{R_f}$ = 0.11 (EtOAc/ petroleum ether, v/v = 2/1);

^{1}H NMR (400 MHz, $CDCl_3$) δ 7.68 (d, *J* = 6.8 Hz, 1H), 7.61 (d, *J* = 8.6 Hz, 1H), 7.52–7.41 (m, 3H), 6.90–6.87 (m, 1H), 6.83–6.82 (m, 1H), 4.74 (s, 1H), 4.11 (q, *J* = 7.2 Hz, 2H), 3.76 (s, 3H), 1.11 (t, *J* = 7.2 Hz, 3H);

^{13}C NMR (101 MHz, $CDCl_3$) δ 172.0, 169.8, 168.2, 157.4, 140.8, 133.8, 133.5, 133.4, 132.8, 130.1, 124.8, 123.2, 117.7, 114.7, 111.9, 79.0, 63.1, 55.7, 52.7, 13.8;

HRMS calcd for $C_{20}H_{17}NNaO_6$ $[M+Na]^+$: 390.0948, found for: 390.0953.

10b-(ethoxycarbonyl)-2-fluoro-6-oxo-10b,11-dihydro-6H-isoindolo[2,1-a]indole-11-carboxylic acid (15)

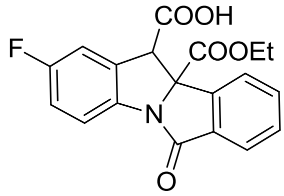

Purification by flash chromatography on silica gel (0.1% AcOH in petroleum ether/EtOAc V/V = 3:1) gave as white solid (46.2 mg, 0.13 mmol, 65% yield);

mp: 192–194 °C;

$\boldsymbol{R_f}$ = 0.19 (EtOAc/ petroleum ether, v/v = 2/1);

^{1}H NMR (400 MHz, $CDCl_3$) δ 7.72–7.70 (m, 1H), 7.67–7.64 (m, 1H), 7.53–7.44 (m, 3H), 7.10–7.05 (m, 1H), 7.00–6.98 (m, 1H), 4.77 (s, 1H), 4.12 (q, *J* = 7.2 Hz, 2H), 1.12 (t, *J* = 7.2 Hz, 3H);

^{13}C NMR (101 MHz, $CDCl_3$) δ 171.3, 169.6, 168.1, 160.1 (d, *J* = 244.7 Hz), 140.8, 136.2 (d, *J* = 2.4 Hz), 133.8 (d, *J* = 8.8 Hz), 133.5, 133.0, 130.2, 125.0, 123.4, 117.9 (d, *J* = 8.6 Hz), 116.5 (d, *J* = 23.5 Hz), 113.3 (d, *J* = 25.0 Hz), 78.9, 63.2, 52.5, 13.8;

^{19}F NMR (376 MHz, $CDCl_3$) δ -116.74, -117.09;

HRMS calcd for $C_{19}H_{14}FNNaO_5$ $[M+Na]^+$: 378.0748, found for: 378.0745.

2-chloro-10b-(ethoxycarbonyl)-6-oxo-10b,11-dihydro-6H-isoindolo[2,1-a]indole-11-carboxylic acid (16)

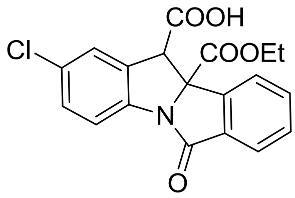

Purification by flash chromatography on silica gel (0.1% AcOH in petroleum ether/EtOAc V/V = 3:1) gave as white solid (43.8 mg, 0.12 mmol, 59% yield);

mp: 192–194 °C;

$\boldsymbol{R_f}$ = 0.17 (EtOAc/ petroleum ether, v/v = 2/1);

^{1}H NMR (400 MHz, DMSO-d_6) δ 7.84–7.82 (m, 1H), 7.80–7.72 (m, 2H), 7.68–7.64 (m, 1H), 7.62–7.57 (m, 2H), 7.49–7.46 (m, 1H), 4.80 (s, 1H), 4.15 (q, *J* = 7.2 Hz, 2H), 1.12 (t, *J* = 7.2 Hz, 3H);

^{13}C NMR (101 MHz, DMSO-d_6) δ 169.6, 167.7, 141.4, 139.3, 136.1, 134.1, 133.1, 131.0, 129.6, 129.4, 126.7, 124.9, 124.8, 117.8, 79.0, 63.5, 53.1, 14.1;

HRMS calcd for $C_{19}H_{14}ClNNaO_5$ $[M+Na]^+$: 394.0453, found for: 394.0466.

2-bromo-10b-(ethoxycarbonyl)-6-oxo-10b,11-dihydro-6H-isoindolo[2,1-a]indole-11-carboxylic acid (17)

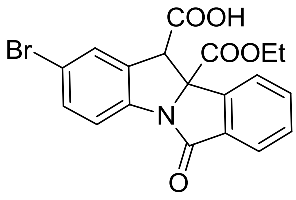

Purification by flash chromatography on silica gel (0.1% AcOH in petroleum ether/EtOAc V/V = 3:1) gave as white solid (49.0 mg, 0.12 mmol, 59% yield);
mp: 194–196 ^{o}C;
***R*f** = 0.15 (EtOAc/ petroleum ether, v/v = 2/1);
^{1}H NMR (400 MHz, $CDCl_3$) δ 7.78–7.76 (m, 1H), 7.63–7.61 (m, 1H), 7.55–7.49 (m, 4H), 7.42–7.41 (m, 1H), 4.78 (s, 1H), 4.13 (q, *J* = 7.2 Hz, 2H), 1.13 (t, *J* = 7.2 Hz, 3H);
^{13}C NMR (101 MHz, $CDCl_3$) δ 171.8, 169.4, 167.9, 140.6, 139.1, 133.9, 133.3, 133.1, 132.9, 130.2, 128.7, 125.1, 123.4, 118.4, 117.8, 78.6, 63.3, 52.2, 13.8;
HRMS calcd for $C_{19}H_{14}BrNNaO_5$ $[M+Na]^+$: 437.9948, found for: 437.9953.

2,10b-bis(ethoxycarbonyl)-6-oxo-10b,11-dihydro-6H-isoindolo[2,1-a]indole-11-carboxylic acid (18)

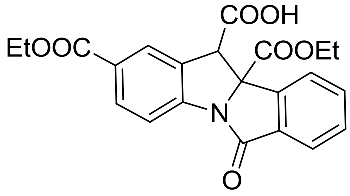

Purification by flash chromatography on silica gel (0.1% AcOH in petroleum ether/EtOAc V/V = 3:1) gave as colorless oil (39.3 mg, 0.10 mmol, 48% yield);
***R*f** = 0.14 (EtOAc/ petroleum ether, v/v = 2/1);
^{1}H NMR (400 MHz, $CDCl_3$) δ 7.80–7.78 (m, 1H), 7.59–7.46 (m, 4H), 7.27–7.21 (m, 1H), 6.85–6.80 (m, 1H), 4.76 (s, 1H), 4.13 (q, *J* = 7.2 Hz, 2H), 3.63 (q, *J* = 6.8 Hz, 2H), 1.79 (t, *J* = 6.8 Hz, 3H), 1.14 (t, *J* = 7.2 Hz, 3H);
^{13}C NMR (101 MHz, $CDCl_3$) δ 172.1, 169.4, 167.7, 165.9, 143.7, 140.9, 133.3, 133.2, 132.3, 132.2, 130.2, 127.2, 127.0, 125.2, 123.5, 116.4, 78.7, 63.3, 61.2, 52.2, 14.3, 13.8;
HRMS calcd for $C_{22}H_{20}NO_7$ $[M+H]^+$: 410.1234, found for: 410.1240.

10b-(ethoxycarbonyl)-3-fluoro-6-oxo-10b,11-dihydro-6H-isoindolo[2,1-a]indole-11-carboxylic acid (19)

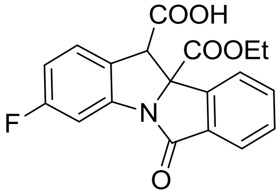

Purification by flash chromatography on silica gel (0.1% AcOH in petroleum ether/EtOAc V/V = 3:1) gave as white solid (42.6 mg, 0.12 mmol, 60% yield);
mp: 188–190 ^{o}C;
***R*f** = 0.28 (EtOAc/ petroleum ether, v/v = 2/1);
^{1}H NMR (400 MHz, DMSO-*d$_6$*) δ 7.84–7.82 (m, 1H), 7.79–7.75 (m, 1H), 7.72–7.71 (m, 1H), 7.68–7.64 (m, 1H), 7.48–7.44 (m, 1H), 7.41–7.38 (m, 1H), 7.03–6.98 (m, 1H), 4.74 (s, 1H), 4.15 (q, *J* = 7.2 Hz, 2H), 1.11 (t, *J* = 7.2 Hz, 3H);
^{13}C NMR (101 MHz, DMSO-*d$_6$*) δ 170.0, 169.7, 167.8, 163.0 (d, *J* = 244.1 Hz), 141.7, 141.5, 134.2, 133.0, 131.0, 130.1, 130.0 (d, *J* = 1.9 Hz), 127.7 (d, *J* = 10.0 Hz), 124.8 (d, *J* = 32.7 Hz), 111.9 (d, *J* = 22.5 Hz), 104.5 (d, *J* = 27.1 Hz), 79.6, 63.4, 52.7, 14.1;
^{19}F NMR (376 MHz, DMSO-*d$_6$*) δ -111.99;
HRMS calcd for $C_{19}H_{14}FNNaO_5$ $[M+Na]^+$: 378.0748, found for: 378.0746.

10b-(ethoxycarbonyl)-3-methoxy-6-oxo-10b,11-dihydro-6H-isoindolo[2,1-a]indole-11-carboxylic acid (20)

Purification by flash chromatography on silica gel (0.1% AcOH in petroleum ether/EtOAc V/V = 2:1) gave as white solid (48.4 mg, 0.13 mmol, 66% yield);

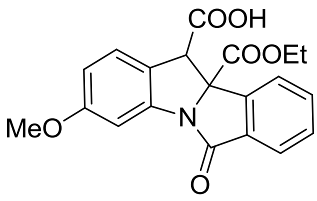

mp: 198–200 ^{o}C;
***R*f** = 0.06 (EtOAc/ petroleum ether, v/v = 2/1);
^{1}H NMR (400 MHz, DMSO-*d$_6$*) δ 7.81–7.62 (m, 4H), 7.32–7.30 (m, 1H),

7.15–7.14 (m, 1H), 6.73–6.71 (m, 1H), 4.65 (s, 1H), 4.14 (q, *J* = 7.2 Hz, 2H), 3.82 (s, 3H), 1.11 (t, *J* = 7.2 Hz, 3H);

^{13}C NMR (101 MHz, DMSO-d_6) δ 169.9, 169.5, 167.2, 160.3, 141.2, 140.9, 133.4, 133.0, 130.3, 126.4, 125.2, 124.3, 123.8, 110.3, 102.4, 79.1, 62.8, 55.8, 52.1, 13.7;

HRMS calcd for $C_{20}H_{17}NNaO_6$ $[M+Na]^+$: 390.0948, found for: 390.0955.

10b-(ethoxycarbonyl)-1-methyl-6-oxo-10b,11-dihydro-6H-isoindolo[2,1-a]indole-11-carboxylic acid (21)

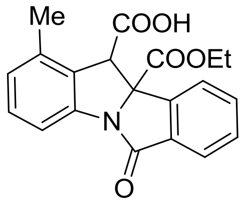

Purification by flash chromatography on silica gel (0.1% AcOH in petroleum ether/EtOAc V/V = 3:1) gave as colorless oil (54.7 mg, 0.16 mmol, 78% yield);

***R*$_f$** = 0.17 (EtOAc/ petroleum ether, v/v = 2/1);

^{1}H NMR (400 MHz, $CDCl_3$) δ 7.80–7.78 (m, 1H), 7.60–7.47 (m, 4H), 7.31–7.28 (m, 1H), 6.96–6.94 (m, 1H), 4.79 (s, 1H), 4.12 (q, *J* = 7.2 Hz, 2H), 2.24 (s, 3H), 1.11 (t, *J* = 7.2 Hz, 3H);

^{13}C NMR (101 MHz, $CDCl_3$) δ 171.4, 170.0, 168.0, 140.9, 139.9, 135.6, 133.9, 132.8, 130.9, 130.1, 129.8, 126.3, 124.9, 123.3, 114.6, 78.5, 63.1, 51.5, 18.3, 13.8;

HRMS calcd for $C_{20}H_{18}NO_5$ $[M+H]^+$: 352.1179, found for: 352.1191.

10b-(ethoxycarbonyl)-2,3-dimethoxy-6-oxo-10b,11-dihydro-6H-isoindolo[2,1-a]indole-11-carboxylic acid (22)

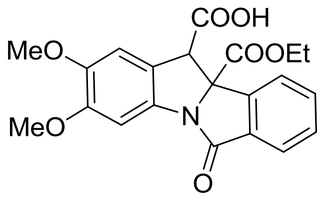

Purification by flash chromatography on silica gel (0.1% AcOH in petroleum ether/EtOAc V/V = 3:1) gave as colorless oil (31.8 mg, 0.08 mmol, 40% yield);

***R*$_f$** = 0.31 (EtOAc/ petroleum ether, v/v = 2/1);

^{1}H NMR (400 MHz, $CDCl_3$) δ 7.78–7.76 (m, 1H), 7.56–7.45 (m, 3H), 7.36 (s, 1H), 6.82 (s, 1H), 4.73 (s, 1H), 4.13 (q, *J* = 7.2 Hz, 2H), 3.96 (s, 3H), 3.83 (s, 3H), 1.14 (t, *J* = 7.2 Hz, 3H);

^{13}C NMR (101 MHz, $CDCl_3$) δ 171.9, 170.0, 167.9, 150.4, 146.7, 140.9, 134.0, 133.9, 132.7, 130.0, 124.8, 123.2, 122.6, 108.7, 101.3, 79.4, 63.1, 56.4, 56.3, 52.5, 13.8;

HRMS calcd for $C_{21}H_{19}NNaO_7$ $[M+Na]^+$:420.1054, found for: 420.1062.

10b-(ethoxycarbonyl)-8-methyl-6-oxo-10b,11-dihydro-6H-isoindolo[2,1-a]indole-11-carboxylic acid (23)

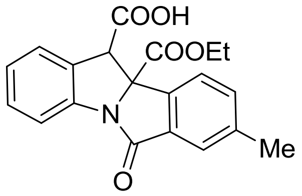

Purification by flash chromatography on silica gel (0.1% AcOH in petroleum ether/EtOAc V/V = 3:1) gave as white solid (47.7 mg, 0.14 mmol, 68% yield);

mp: 170–172 ^oC;

***R*$_f$** = 0.22 (EtOAc/ petroleum ether, v/v = 2/1);

^{1}H NMR (400 MHz, $CDCl_3$) δ 7.73–7.71 (m, 1H), 7.59–7.58 (m, 1H), 7.42–7.35 (m, 2H), 7.32–7.30 (m, 1H), 7.27–7.25 (m, 1H), 7.13–7.09 (m, 1H), 4.79 (s, 1H), 4.09 (q, *J* = 7.2 Hz, 2H), 2.39 (s, 3H), 1.10 (t, *J* = 7.2 Hz, 3H);

^{13}C NMR (101 MHz, $CDCl_3$) δ 172.4, 170.0, 168.1, 140.4, 139.9, 138.1, 133.8, 133.7, 132.0, 129.8, 125.5, 125.2, 124.9, 123.0, 117.0, 78.3, 63.0, 52.3, 21.3, 13.7;

HRMS calcd for $C_{20}H_{17}NNaO_5$ $[M+Na]^+$: 374.0999, found for: 374.1000.

10b-(ethoxycarbonyl)-9-methyl-6-oxo-10b,11-dihydro-6H-isoindolo[2,1-a]indole-11-carboxylic acid (24)

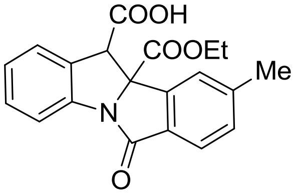

Purification by flash chromatography on silica gel (0.1% AcOH in petroleum ether/EtOAc V/V = 3:1) gave as white solid (44.9 mg, 0.13 mmol, 64% yield);
mp: 175–177 ^{o}C;
$\boldsymbol{R}_{\mathbf{f}}$ = 0.13 (EtOAc/ petroleum ether, v/v = 2/1);
^{1}H NMR (400 MHz, $CDCl_3$) δ 7.73–7.71 (m, 1H), 7.63–7.61 (m, 1H), 7.39–7.35 (m, 1H), 7.32 (s, 1H), 7.25–7.23 (m, 2H), 7.13–7.09 (m, 1H), 4.78 (s, 1H), 4.09 (q, *J* = 7.2 Hz, 2H), 2.36 (s, 3H), 1.11 (t, *J* = 7.2 Hz, 3H);
^{13}C NMR (101 MHz, $CDCl_3$) δ 172.4, 169.2, 168.2, 139.9, 139.2, 134.7, 134.3, 133.6, 132.7, 130.0, 129.8, 125.5, 125.1, 122.6, 116.9, 78.6, 63.0, 50.8, 18.1, 13.7;
HRMS calcd for $C_{19}H_{16}NO_3$ $[M\text{-}COOH]^-$: 306.1136, found for: 306.1134.

10b-(ethoxycarbonyl)-10-methyl-6-oxo-10b,11-dihydro-6H-isoindolo[2,1-a]indole-11-carboxylic acid (25)

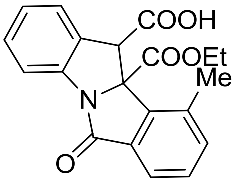

Purification by flash chromatography on silica gel (0.1% AcOH in petroleum ether/EtOAc V/V = 3:1) gave as white solid (41.4 mg, 0.12 mmol, 59% yield);
mp: 174–176 ^{o}C;
$\boldsymbol{R}_{\mathbf{f}}$ = 0.25 (EtOAc/ petroleum ether, v/v = 2/1);
^{1}H NMR (400 MHz, $CDCl_3$) δ 7.74–7.72 (m, 1H), 7.40–7.36 (m, 1H), 7.33–7.31 (m, 2H), 7.26–7.20 (m, 2H), 7.13–7.09 (m, 1H), 4.78 (s, 1H), 4.10 (q, *J* = 7.2 Hz, 2H), 2.66 (s, 3H), 1.12 (t, *J* = 7.2 Hz, 3H);
^{13}C NMR (101 MHz, $CDCl_3$) δ 172.4, 169.2, 168.2, 139.9, 139.2, 134.7, 134.3, 133.6, 132.7, 130.0, 129.8, 125.5, 125.1, 122.6, 116.9, 78.6, 63.0, 50.8, 18.1, 13.7;
HRMS calcd for $C_{19}H_{16}NO_3$ $[M\text{-}COOH]^-$: 306.1136, found for: 306.1134.

10b-(ethoxycarbonyl)-9-fluoro-6-oxo-10b,11-dihydro-6H-isoindolo[2,1-a]indole-11-carboxylic acid (26)

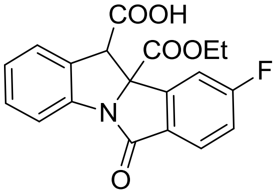

Purification by flash chromatography on silica gel (0.1% AcOH in petroleum ether/EtOAc V/V = 3:1) gave as white solid (53.3 mg, 0.15 mmol, 75% yield);
mp: 181–183 ^{o}C;
$\boldsymbol{R}_{\mathbf{f}}$ = 0.13 (EtOAc/ petroleum ether, v/v = 2/1);
^{1}H NMR (400 MHz, $CDCl_3$) δ 7.74–7.72 (m, 1H), 7.51–7.46 (m, 1H), 7.41–7.37 (m, 1H), 7.32–7.27 (m, 2H), 7.16–7.10 (m, 2H), 4.80 (s, 1H), 4.12 (q, *J* = 7.2 Hz, 2H), 1.12 (t, *J* = 7.2 Hz, 3H);
^{13}C NMR (101 MHz, $CDCl_3$) δ 172.2, 169.5, 164.7 (d, *J* = 2.2 Hz), 159.0 (d, *J* = 263.1 Hz), 143.3 (d, *J* = 2.9 Hz), 139.7, 135.0 (d, *J* = 7.8 Hz), 131.7, 130.0, 125.6, 125.3, 120.8 (d, *J* = 13.8 Hz), 119.3 (d, *J* = 4.0 Hz), 117.6 (d, *J* = 19.2 Hz), 117.2, 78.2, 63.3, 52.4, 13.8;
^{19}F NMR (376 MHz, $CDCl_3$) δ -115.33, -116.00;
HRMS calcd for $C_{19}H_{13}FNO_5$ $[M\text{-}H]^-$: 354.0783, found for: 354.0775.

10b-(ethoxycarbonyl)-10-fluoro-6-oxo-10b,11-dihydro-6H-isoindolo[2,1-a]indole-11-carboxylic acid (27)

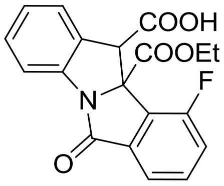

Purification by flash chromatography on silica gel (0.1% AcOH in petroleum

ether/EtOAc V/V = 3:1) gave as white solid (42.6 mg, 0.12 mmol, 60% yield);
mp: 186–188 ^{o}C;
***R*f** = 0.25 (EtOAc/ petroleum ether, v/v = 2/1);
^{1}H NMR (400 MHz, $CDCl_3$) δ 7.73–7.71 (m, 1H), 7.64–7.62 (m, 1H), 7.51–7.46(m, 1H), 7.41–7.35 (m, 2H), 7.22–7.13 (m, 2H), 5.03 (s, 1H), 4.11 (q, *J* = 5.2 Hz, 2H), 1.10 (t, *J* = 7.2 Hz, 3H);
^{13}C NMR (101 MHz, $CDCl_3$) δ 172.6 , 168.1 , 166.8 (d, *J* = 2.3 Hz), 157.2 (d, *J* = 253.7 Hz), 138.9 , 136.6 (d, *J* = 3.3 Hz), 132.3 (d, *J* = 6.6 Hz), 131.9, 129.9, 127.9 (d, *J* = 17.4 Hz), 125.9, 125.4, 121.0 (d, *J* = 3.7 Hz), 119.9 (d, *J* = 19.5 Hz), 116.8, 76.5, 63.3, 50.8, 13.7;
^{19}F NMR (376 MHz, $CDCl_3$) δ -116.90;
HRMS calcd for$C_{18}H_{13}FNO_3$ [M-COOH]$^-$: 310.0885, found for: 310.0886.

10b-(ethoxycarbonyl)-7,8-difluoro-6-oxo-10b,11-dihydro-6H-isoindolo[2,1-a]indole-11-carboxylic acid (28)

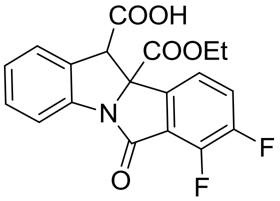

Purification by flash chromatography on silica gel (0.1% AcOH in petroleum ether/EtOAc V/V = 3:1) gave as colorless oil (35.8 mg, 0.10 mmol, 48% yield);
***R*f** = 0.19 (EtOAc/ petroleum ether, v/v = 2/1);
^{1}H NMR (400 MHz, $CDCl_3$) δ 7.72–7.70 (m, 1H), 7.63–7.59 (m, 1H), 7.43–7.38 (m, 2H), 7.31–7.30 (m, 1H), 7.17–7.13 (m, 1H), 4.76 (s, 1H), 4.14 (q, *J* = 7.2 Hz, 2H), 1.15 (t, *J* = 7.2 Hz, 3H);
^{13}C NMR (101 MHz, $CDCl_3$) δ 172.2, 169.1, 165.9, 154.1 (dd, *J* = 163.0, 14.1 Hz), 151.6 (dd, *J* = 159.7, 14.0 Hz), 139.5, 137.4 (dd, *J* = 8.0, 3.2 Hz), 131.4, 130.3 (dd, *J* = 6.6, 2.7 Hz), 130.1, 125.6 (d, *J* = 23.8 Hz), 117.0, 116.8, 113.9 (d, *J* = 19.3 Hz), 112.9 (d, *J* = 20.6 Hz), 77.8, 63.5, 52.3, 13.8;
^{19}F NMR (376 MHz, $CDCl_3$) δ -127.25, -132.50;
HRMS calcd for $C_{19}H_{12}F_2NO_5$ [M-H]$^-$: 372.0689, found for: 372.0682.

10b-(ethoxycarbonyl)-7,8-dimethoxy-6-oxo-10b,11-dihydro-6H-isoindolo[2,1-a]indole-11-carboxylic acid (29)

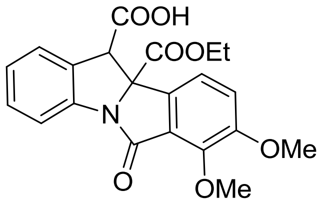

Purification by flash chromatography on silica gel (0.1% AcOH in petroleum ether/EtOAc V/V = 3:1) gave as colorless oil (38.9 mg, 0.10 mmol, 49% yield);
***R*f** = 0.20 (EtOAc/ petroleum ether, v/v = 2/1);
^{1}H NMR (400 MHz, $CDCl_3$) δ 7.72–7.70 (m, 1H), 7.40–7.36 (m, 1H), 7.28–7.25 (m, 2H), 7.14–7.12 (m, 1H), 7.00 (s, 1H), 4.79 (s, 1H), 4.18 – 4.08 (m, 2H), 3.85 (s, 6H), 1.13 (t, *J* = 7.2 Hz, 3H);
^{13}C NMR (101 MHz, $CDCl_3$) δ 170.9, 170.4, 168.6, 153.3, 150.8, 140.3, 135.2, 132.5, 129.5, 126.1, 125.5, 124.7, 116.7, 106.0, 105.7, 78.2, 62.9, 56.2, 52.7, 39.9, 13.8;
HRMS calcd for $C_{21}H_{18}NO_7$ [M-H]$^-$: 396.1089, found for: 396.1086.

3b-(ethoxycarbonyl)-10-oxo-4,10-dihydro-3bH-thieno[3',2':3,4]pyrrolo[1,2-a]indole-4-carboxylic acid (30)

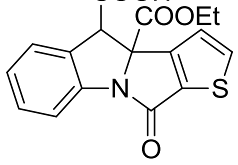

Purification by flash chromatography on silica gel (0.1% AcOH in petroleum ether/EtOAc V/V = 3:1) gave as colorless oil (28.1 mg, 0.08 mmol, 41% yield);
***R*f** = 0.22 (EtOAc/ petroleum ether, v/v = 2/1);
^{1}H NMR (400 MHz, DMSO-*d6*) δ 8.19–8.18 (m, 1H), 7.52–7.50 (m, 1H),

7.41–7.36 (m, 2H), 7.30–7.29 (m, 1H), 7.17–7.15 (m, 1H), 4.68 (s, 1H), 4.16 (q, *J* = 7.2 Hz, 2H), 1.14 (t, *J* = 7.2 Hz, 3H);

^{13}C NMR (101 MHz, DMSO-*d6*) δ 170.2, 168.8, 164.0, 152.8, 140.8, 139.5, 136.4, 133.5, 129.6, 126.6, 125.2, 123.1, 116.3, 77.4, 63.3, 52.9, 14.2;

HRMS calcd for $C_{17}H_{13}NNaO_5S$ $[M+Na]^+$: 366.0407, found for: 366.0403.

10b-(ethoxycarbonyl)-10b,11-dihydro-6H-isoindolo[2,1-a]indole-11-carboxylic acid (31)

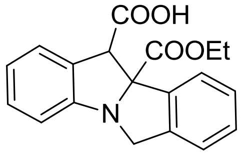

Purification by flash chromatography on silica gel (0.1% AcOH in petroleum ether/EtOAc V/V = 3:1) gave as white solid (49.1 mg, 0.15 mmol, 76% yield, dr = 3.2:1) for X = Br; (44.6 mg, 0.14 mmol, 69% yield, dr = 3.5:1) for X = I;

mp: 162–164 ^{o}C;

***R*$_f$** = 0.14 (EtOAc/ petroleum ether, v/v = 2/1);

^{1}H NMR (400 MHz, $CDCl_3$) δ 7.54–7.52 (m, 1H), 7.28–7.24 (m, 2H), 7.21–7.17 (m, 3H), 6.96–6.94 (m, 1H), 6.85–6.81 (m, 1H), 4.93 (d, *J* = 14.8 Hz, 1H), 4.73 (s, 1H), 4.56 (d, *J* = 14.8 Hz, 1H), 4.16 (q, *J* = 7.2 Hz, 2H), 1.11 (t, *J* = 7.2 Hz, 3H);

^{13}C NMR (101 MHz, $CDCl_3$) δ 177.1, 170.8, 153.6, 140.5, 139.5, 129.6, 129.4, 127.7, 126.0, 124.6, 123.2, 123.0, 121.7, 113.5, 85.0, 61.7, 59.7, 56.5, 14.2;

HRMS calcd for $C_{19}H_{16}NO_4$ $[M-H]^-$: 322.1085, found for: 322.1085.

10b-(ethoxycarbonyl)-8-fluoro-10b,11-dihydro-6H-isoindolo[2,1-a]indole-11-carboxylic acid (32)

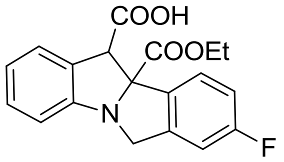

Purification by flash chromatography on silica gel (0.1% AcOH in petroleum ether/EtOAc V/V = 3:1) gave as white solid (54.6 mg, 0.16 mmol, 80% yield, dr = 2.8:1) for X = Br;

mp: 160–162 ^{o}C;

***R*$_f$** = 0.22 (EtOAc/ petroleum ether, v/v = 2/1);

^{1}H NMR (400 MHz, $CDCl_3$) δ 7.49–7.46 (m, 1H), 7.22–7.18 (m, 2H), 6.98–6.83 (m, 4H), 4.89 (d, *J* = 15.2 Hz, 1H), 4.68 (s, 1H), 4.53 (d, *J* = 15.2 Hz, 1H), 4.09 (q, *J* = 7.2 Hz, 2H), 1.13 (t, *J* = 7.2 Hz, 3H);

^{13}C NMR (101 MHz, $CDCl_3$) δ 176.8, 170.7, 163.8 (d, *J* = 248.1 Hz), 153.3, 142.0 (d, *J* = 8.4 Hz), 136.0 (d, *J* = 2.2 Hz), 129.8, 125.8, 124.7, 124.5 (d, *J* = 9.4 Hz), 122.0, 115.1 (d, *J* = 23.5 Hz), 113.5, 110.4 (d, *J* = 23.4 Hz), 84.4, 61.9, 59.4, 56.5, 13.8;

^{19}F NMR (376 MHz, $CDCl_3$) δ -118.55;

HRMS calcd for $C_{19}H_{17}FNO_4$ $[M+H]^+$: 342.1136, found for: 342.1142.

8-chloro-10b-(ethoxycarbonyl)-10b,11-dihydro-6H-isoindolo[2,1-a]indole-11-carboxylic acid (33)

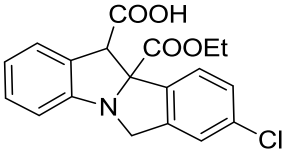

Purification by flash chromatography on silica gel (0.1% AcOH in petroleum ether/EtOAc V/V = 3:1) gave as white solid (55.0 mg, 0.15 mmol, 77% yield, dr = 2.1:1) for X = Br;

mp: 178–180 ^{o}C;

***R*$_f$** = 0.22 (EtOAc/ petroleum ether, v/v = 2/1);

^{1}H NMR (400 MHz, $CDCl_3$) δ 7.45–7.43 (m, 1H), 7.23–7.16 (m, 4H), 6.93–6.91 (m, 1H), 6.86–6.82 (m, 1H), 4.87 (d, *J* = 15.6 Hz, 1H), 4.66 (s, 1H), 4.51 (d, *J* = 15.2 Hz, 1H), 4.07 (q, *J* = 7.2 Hz, 2H), 1.10 (t, *J* = 6.8 Hz, 3H);

^{13}C NMR (101 MHz, $CDCl_3$) δ 176.8, 170.4, 153.1, 141.5, 139.0, 135.3, 129.8, 128.1, 125.6, 124.7, 124.3, 123.4, 122.1, 113.5, 84.5, 61.9, 59.3, 56.4, 13.7;

HRMS calcd for $C_{19}H_{17}ClNO_4$ $[M+H]^+$: 358.0846, found for: 358.0846.

10b-(ethoxycarbonyl)-8-methoxy-10b,11-dihydro-6H-isoindolo[2,1-a]indole-11-carboxylic acid (34)

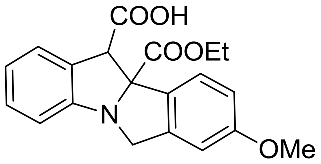

Purification by flash chromatography on silica gel (0.1% AcOH in petroleum ether/EtOAc V/V = 3:1) gave as white solid (31.1 mg, 0.09 mmol, 44% yield, dr = 1.8:1) for X = Br;
mp: 179–181 °C;
***R*f** = 0.13 (EtOAc/ petroleum ether, v/v = 2/1) for X = Br;
^{1}H NMR (400 MHz, $CDCl_3$) δ 7.42–7.39 (m, 1H), 7.20–7.17 (m, 2H), 6.94–6.92 (m, 1H), 6.84–6.78 (m, 2H), 6.72–6.71 (m, 1H), 4.88 (d, *J* = 14.8 Hz, 1H), 4.68 (s, 1H), 4.50 (d, *J* = 15.2 Hz, 1H), 4.08 (q, *J* = 7.2 Hz, 2H), 3.75 (s, 3H), 1.12 (t, *J* = 7.2 Hz, 3H);
^{13}C NMR (101 MHz, $CDCl_3$) δ 177.1, 171.0, 161.0, 153.6, 141.3, 132.4, 129.6, 126.0, 124.7, 123.9, 121.8, 114.0, 113.5, 108.2, 84.5, 61.7, 59.7, 56.5, 55.5, 13.8;
HRMS calcd for $C_{20}H_{20}NO_5$ $[M+H]^+$: 354.1336, found for: 354.1341.

2-chloro-10b-(ethoxycarbonyl)-10b,11-dihydro-6H-isoindolo[2,1-a]indole-11-carboxylic acid (35)

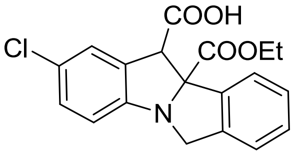

Purification by flash chromatography on silica gel (0.1% AcOH in petroleum ether/EtOAc V/V = 3:1) gave as white solid (55.0 mg, 0.15 mmol, 77% yield, dr = 6.4:1) for X = I;
mp: 181–183 °C;
***R*f** = 0.18 (EtOAc/ petroleum ether, v/v = 2/1);
^{1}H NMR (400 MHz, $CDCl_3$) δ 7.54–7.52 (m, 1H), 7.30–7.28 (m, 2H), 7.23–7.21 (m, 1H), 7.16–7.14 (m, 2H), 6.88–6.86 (m, 1H), 4.92 (d, *J* = 14.8 Hz, 1H), 4.70 (s, 1H), 4.50 (d, *J* = 15.2 Hz, 1H), 4.10 (q, *J* = 7.2 Hz, 2H), 1.14 (t, *J* = 7.2 Hz, 3H);
^{13}C NMR (101 MHz, $CDCl_3$) δ 176.4, 170.4, 152.3, 140.0, 139.2, 129.7, 129.6, 127.9, 127.6, 126.5, 124.7, 123.2, 123.1, 114.4, 85.2, 61.9, 59.7, 56.3, 13.7;
HRMS calcd for $C_{19}H_{17}ClNO_4$ $[M+H]^+$: 358.0841, found for: 358.0844.

2-bromo-10b-(ethoxycarbonyl)-10b,11-dihydro-6H-isoindolo[2,1-a]indole-11-carboxylic acid (36)

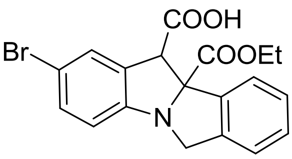

Purification by flash chromatography on silica gel (0.1% AcOH in petroleum ether/EtOAc V/V = 3:1) gave as white solid (61.0 mg, 0.15 mmol, 76% yield, dr = 7.7:1) for X = I;
mp: 186–188 °C;
***R*f** = 0.11 (EtOAc/ petroleum ether, v/v = 2/1);
^{1}H NMR (400 MHz, $CDCl_3$) δ 7.54–7.52 (m, 1H), 7.30–7.28 (m, 4H), 7.23–7.21 (m, 1H), 6.83–6.81 (m, 1H), 4.92 (d, *J* = 15.2 Hz, 1H), 4.71 (s, 1H), 4.49 (d, *J* = 15.2 Hz, 1H), 4.10 (q, *J* = 7.2 Hz, 2H), 1.15 (t, *J* = 7.2 Hz, 3H);
^{13}C NMR (101 MHz, $CDCl_3$) δ 176.0, 170.4, 152.8, 140.0, 139.2, 132.5, 129.6, 128.1, 127.9, 127.5, 123.2, 123.1, 114.9, 113.6, 85.2, 61.9, 59.6, 56.2, 13.7;
HRMS calcd for $C_{19}H_{15}BrNO_4$ $[M-H]^-$: 400.0190, found for: 400.0187.

10b-(ethoxycarbonyl)-2-fluoro-10b,11-dihydro-6H-isoindolo[2,1-a]indole-11-carboxylic acid (37)

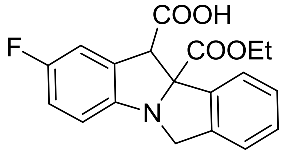

Purification by flash chromatography on silica gel (0.1% AcOH in petroleum ether/EtOAc V/V = 3:1) gave as white solid (40.2 mg, 0.12 mmol, 59% yield, dr = 3.2:1) for X = I;

mp: 180–182 ^{o}C;

$\boldsymbol{R_f}$ = 0.15 (EtOAc/ petroleum ether, v/v = 2/1);

^{1}H NMR (400 MHz, $CDCl_3$) δ 8.49 (br, 1H), 7.49–7.46 (m, 1H), 7.22–7.18 (m, 2H), 6,98–6.83 (m, 4H), 4.89 (d, *J* = 15.2 Hz, 1H), 4.68 (s, 1H), 4.53 (d, *J* = 15.2 Hz, 1H), 4.08 (q, *J* = 7.2 Hz, 2H), 1.11 (t, *J* = 7.2 Hz, 3H);

^{13}C NMR (101 MHz, $CDCl_3$) δ 176.9, 170.6, 163.8 (d, *J* = 248.2 Hz), 153.3, 142.0 (d, *J* = 8.4 Hz), 136.0 (d, *J* = 2.2 Hz), 129.8, 125.8, 124.7, 124.5 (d, *J* = 9.5 Hz), 122.0, 115.1 (d, *J* = 23.4 Hz), 113.6, 110.4 (d, *J* = 23.3 Hz), 84.4, 61.9, 59.5 (d, *J* = 2.2 Hz), 56.5, 13.8;

^{19}F NMR (376 MHz, $CDCl_3$) δ -112.39;

HRMS calcd for $C_{19}H_{17}FNO_4$ $[M+H]^+$: 342.1136, found for: 342.1133.

10b-(ethoxycarbonyl)-2-methyl-10b,11-dihydro-6H-isoindolo[2,1-a]indole-11-carboxylic acid (38)

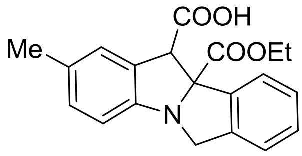

Purification by flash chromatography on silica gel (0.1% AcOH in petroleum ether/EtOAc V/V = 3:1) gave as white solid (55.9 mg, 0.17 mmol, 83% yield, dr = 4.6:1) for X = I;

mp: 179–181 ^{o}C;

$\boldsymbol{R_f}$ = 0.12 (EtOAc/ petroleum ether, v/v = 2/1);

^{1}H NMR (400 MHz, $CDCl_3$) δ 7.53–7.51 (m, 1H), 7.26–7.18 (m, 3H), 7.00–6.98 (m, 2H), 6.86–6.83 (m, 1H), 4.91 (d, *J* = 14.8 Hz, 1H), 4.69 (s, 1H), 4.52 (d, *J* = 14.8 Hz, 1H), 4.09 (q, *J* = 7.2 Hz, 2H), 2.20 (s, 3H), 1.14 (t, *J* = 7.2 Hz, 3H);

^{13}C NMR (101 MHz, $CDCl_3$) δ 177.0, 170.9, 151.3, 140.6, 139.6, 131.3, 130.3, 129.4, 127.7, 125.9, 125.1, 123.2, 123.1, 113.3, 85.2, 61.7, 59.8, 56.6, 20.7, 13.7;

HRMS calcd for $C_{20}H_{20}NO_4$ $[M+H]^+$: 338.1387, found for: 338.1392.

12a-(ethoxycarbonyl)-5,6,12,12a-tetrahydroindolo[2,1-a]isoquinoline-12-carboxylic acid (39)

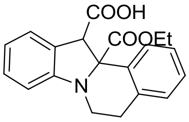

Purification by flash chromatography on silica gel (0.1% AcOH in petroleum ether/EtOAc V/V = 5:1) gave as colorless oil (28.3 mg, 0.08 mmol, 42% yield);

$\boldsymbol{R_f}$ = 0.35 (EtOAc/ petroleum ether, v/v = 2/1);

^{1}H NMR (400 MHz, DMSO-d_6) δ 7.71 (d, *J* = 8.0 Hz, 1H), 7.25 (t, *J* = 8.0 Hz, 1H), 7.20 – 7.11 (m, 2H), 7.06 – 7.01 (m, 2H), 6.84 (d, *J* = 8.0 Hz, 1H), 6.63 (t, *J* = 8.0 Hz, 1H), 4.40 (s, 1H), 4.12 – 4.04 (m, 2H), 3.94 (dd, *J* = 8.0, 4.0 Hz, 2H), 2.89 – 2.80 (m, 1H), 2.55 (s, 1H), 1.19 (t, *J* = 8.0 Hz, 3H) ;

^{13}C NMR (101 MHz, DMSO-d_6) δ 172.0, 170.8, 149.8, 135.6, 129.3, 129.0, 127.7, 127.6, 126.7, 124.5, 118.6, 108.8, 79.2, 74.6, 60.9, 60.1, 22.9, 13.7;

HRMS calcd for $C_{20}H_{18}NO_4$ $[M-H]^-$: 336.1241, found for: 336.1246.

13a-(ethoxycarbonyl)-6,7,13,13a-tetrahydro-5H-benzo[3,4]azepino[1,2-a]indole-13-carboxylic acid (40)

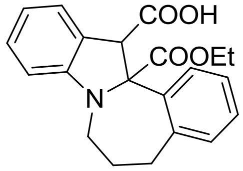

Purification by flash chromatography on silica gel (petroleum ether/EtOAc V/V = 2:1) gave as colorless oil (9.9 mg, 0.03 mmol, 14% yield);

$\boldsymbol{R_f}$ = 0.36 (EtOAc/ petroleum ether, v/v = 1/2);

^{1}H NMR (400 MHz,$CDCl_3$) δ 7.20 – 7.11 (m, 3H), 7.08 – 7.02 (m, 3H), 6.71 (t, *J* = 8.0 Hz, 1H), 6.55 (d, *J* = 8.0 Hz, 1H), 5.29 (s, 1H), 4.23 (q, J = 8.0 Hz, 2H), 3.67 – 3.64 (m, 2H), 2.94 – 2.86 (m, 1H), 2.35 – 2.26 (m, 2H), 1.74 – 1.66 (m, 1H), 1.22 (t, *J* = 8.0 Hz, 3H) ;
^{13}C NMR (101 MHz, $CDCl_3$) δ 174.5, 172.3, 149.3, 131.0, 129.0, 128.5, 128.4, 126.0, 125.5, 124.7, 117.7, 107.3, 81.6, 62.2, 55.9, 41.1, 30.7, 24.1, 14.0;
HRMS calcd for $C_{21}H_{21}NNaO_4$ $[M+Na]^+$: 374.1363, found for: 374.1359.

General procedure for the synthesis of 17[4]

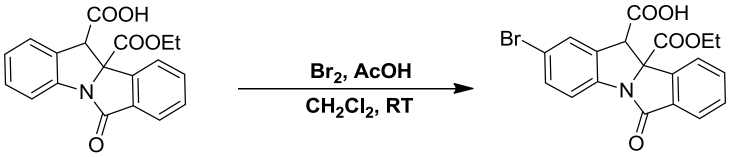

2 **17**

To a suspension of **2** (0.2 mmol, 67.4 mg) in AcOH (2 mL) was added liquid Br_2 (2 mmol, 310 mg) at room temperature. The mixture was stirred for 0.5 h and then CH_2Cl_2 (2 mL) was added to the mixture. The reaction mixture was stirred at room temperature until **2** was fully exhausted (monitored by TLC). the reaction was quenched with saturated $Na_2S_2O_3$ solution and extracted with CH_2Cl_2 . The organic layers were washed with brine and then dried over Na_2SO_4. After filtration and concentration under vacuum, the residue was purified by flash chromatography on silica gel with (0.1% AcOH in petroleum ether/EtOAc V/V = 3:1) to afford **17** in 70% yield.

General procedure for the synthesis of 41[5]

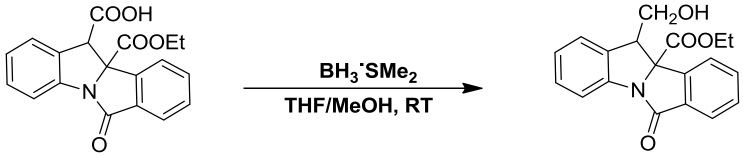

2 **41**

To a solution of **2** (0.2 mmol, 67.4 mg) in THF (2 mL) was added $BH_3 \cdot SMe_2$ (2 mmol, 0.2mL) under argon. The mixture was stirred at room temperature for 24 h. Then, MeOH (1 mL/mmol of substrate) was slowly added. the mixture was concentrated in vacuo and purified with flash chromatography on silica gel, eluting with ethyl acetate/petroleum ether (v/v = 2:1) to afford the desired product **41** in 63% yield.

ethyl 11-(hydroxymethyl)-6-oxo-10b,11-dihydro-6H-isoindolo[2,1-a]indole-10b-carboxylate (41)

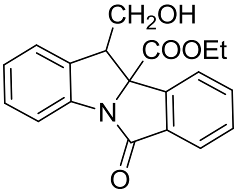

Purification by flash chromatography on silica gel (petroleum ether/EtOAc V/V = 2:1) gave as colorless oil (40.4 mg, 0.13 mmol, 63% yield);
R_f = 0.32 (EtOAc/ petroleum ether, v/v = 1/1);
^{1}H NMR (400 MHz, $CDCl_3$) δ 7.88–7.86 (m, 1H), 7.76–7.71 (m, 2H), 7.63–7.52 (m, 2H), 7.38–7.30 (m, 2H), 7.15–7.11 (m, 1H), 4.12–4.06 (m, 3H), 3.52 (dd, *J* = 11.2, 5.6 Hz, 1H), 3.31 (dd, *J* = 11.2, 5.2 Hz, 1H), 1.12 (t, *J* = 7.2 Hz, 3H);

^{13}C NMR (101 MHz, $CDCl_3$) δ 170.9, 167.8, 140.8, 139.6, 134.5, 134.1, 132.6, 129.8, 129.1, 125.5, 124.9, 124.8, 124.2, 116.7, 78.9, 63.4, 62.6, 49.3, 13.8;
HRMS calcd for $C_{19}H_{18}NO_4$ $[M+H]^+$: 324.1230, found for: 324.1230.

General procedure for the synthesis of 42[6]

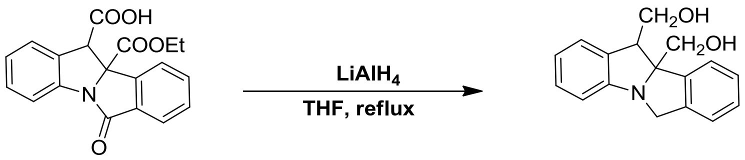

2 **42**

To a stirred suspension of $LiAlH_4$ (1 mmol, 37.9 mg) in THF (1 mL) was added dropwise a solution of **2** (0.2 mmol, 67.4 mg) in THF (1 mL) under argon at room temperature. Then, it was heated to reflux and allowed to stir for further 12 hours. After which, the mixture was quenched with 10% NaOH aqueous solution at room temperature and extracted with ethyl acetate. The organic layers were washed with brine and dried over Na_2SO_4. After filtered and concentrated under vacuum, the residue was purified with flash chromatography on silica gel, eluting with petroleum ether/EtOAc (V/V = 1:1) to afford **42** in 70% yield.

(10b,11-dihydro-6H-isoindolo[2,1-a]indole-10b,11-diyl)dimethanol (42)

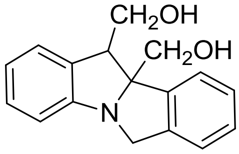

Purification by flash chromatography on silica gel (petroleum ether/EtOAc V/V = 1:1) gave as colorless oil (37.4 mg, 0.14 mmol, 70% yield);
R_f = 0.27 (EtOAc/ petroleum ether, v/v = 1/1);
^{1}H NMR (400 MHz, DMSO-d_6) 7.64–7.62 (m, 1H), 7.22–7.18 (m, 3H), 7.07–7.01 (m, 2H), 6.73–6.71 (m, 1H), 6.68–6.64 (m, 1H), 5.07 (t, J = 4.0 Hz, 1H), 4.87 (t, J = 5.6 Hz, 1H), 4.48 (d, J = 14.8 Hz, 1H), 4.36 (d, J = 15.2 Hz, 1H), 3.74–3.69 (m, 1H), 3.65–3.52 (m, 4H);
^{13}C NMR (101 MHz, DMSO-d_6) δ 154.3, 141.3, 141.2, 131.4, 128.1, 127.9, 126.9, 125.6, 124.3, 122.9, 119.8, 111.5, 85.6, 68.9, 62.2, 57.8, 48.5;
HRMS calcd for $C_{17}H_{18}NO_2$ $[M+H]^+$: 268.1332, found for: 268.1333.

Mechanistic Studies

Control experiments with radical scavengers

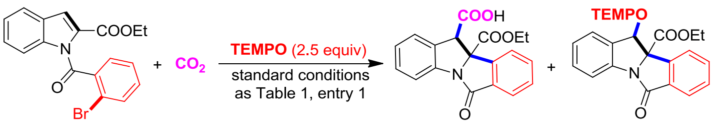

1, 0.2 mmol (1 atm, closed) **2**, 25% **43**, 52%

Following the general procedures, we conducted the reaction of ethyl 1-(2-bromobenzoyl)-1H-indole-2-carboxylate **1** (0.2 mmol, 74 mg, 1.0 equiv), 4CzIPN (1.6 mg, 0.002 mmol, 1 mol%), Cs_2CO_3 (195 mg, 0.6 mmol, 3.0 equiv), DIPEA (100 μL, 0.6 mmol, 3.0 equiv), DMSO (2

mL) and TEMPO (78 mg, 0.5 mmol, 2.5 equiv) in the presence of CO_2 (1 atm, closed) for 24 h at rt. we detected the radical adduct in HRMS (HRMS $[M+Na]^+$ calculated m/z for $[C_{27}H_{32}N_2NaO_4]^+$: 471.2260, found: 471.2259), in addition, obtained **43** in 52% yield. This result further confirmed the formation of benzyl radical. **^{1}H NMR** (400 MHz, DMSO-d_6) δ 7.83 (p, J = 7.5 Hz, 2.8H), 7.75 (d, J = 7.6 Hz, 0.2H), 7.69 – 7.58 (m, 2.8H), 7.56 – 7.52 (m, 0.2H), 7.48 (td, J = 7.7, 1.3 Hz, 0.8H), 7.40 – 7.34 (m, 0.2H), 7.19 (td, J = 7.5, 1.1 Hz, 0.8H), 7.12 (td, J = 7.5, 1.1 Hz, 0.2H), 6.05 (s, 0.2H), 5.71 (s, 0.8H), 4.14 (q, J = 7.1 Hz, 2H), 1.53 – 1.31 (m, 6H), 1.20 – 0.94 (m, 9H), 0.15 (s, 3H), -0.36 (s, 3H). **^{13}C NMR** (101 MHz, DMSO-d_6) δ 169.01, 168.66, 168.14, 167.30, 145.06, 142.20, 141.42, 140.87, 135.55, 134.24, 133.95, 133.46, 132.95, 131.47, 131.12, 130.43, 130.35, 130.27, 129.64, 125.88, 125.05, 124.55, 124.45, 124.20, 124.11, 86.36, 81.40, 81.30, 79.65, 79.12, 63.02, 62.39, 61.56, 61.22, 60.46, 58.92, 35.00, 34.75, 33.59, 30.98, 21.15, 20.90, 20.18, 17.11, 17.00, 14.16, 14.04. **HRMS** calcd for $C_{27}H_{32}N_2NaO_4$ $[M+Na]^+$: 471.2260, found for: 471.2259.

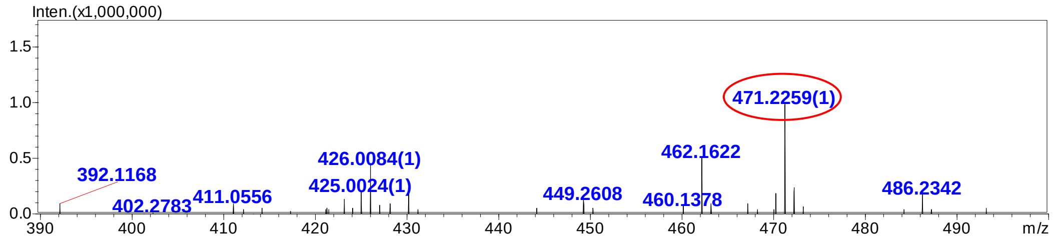

Supplementary Figure 1. HRMS Spectra of compound **43**.

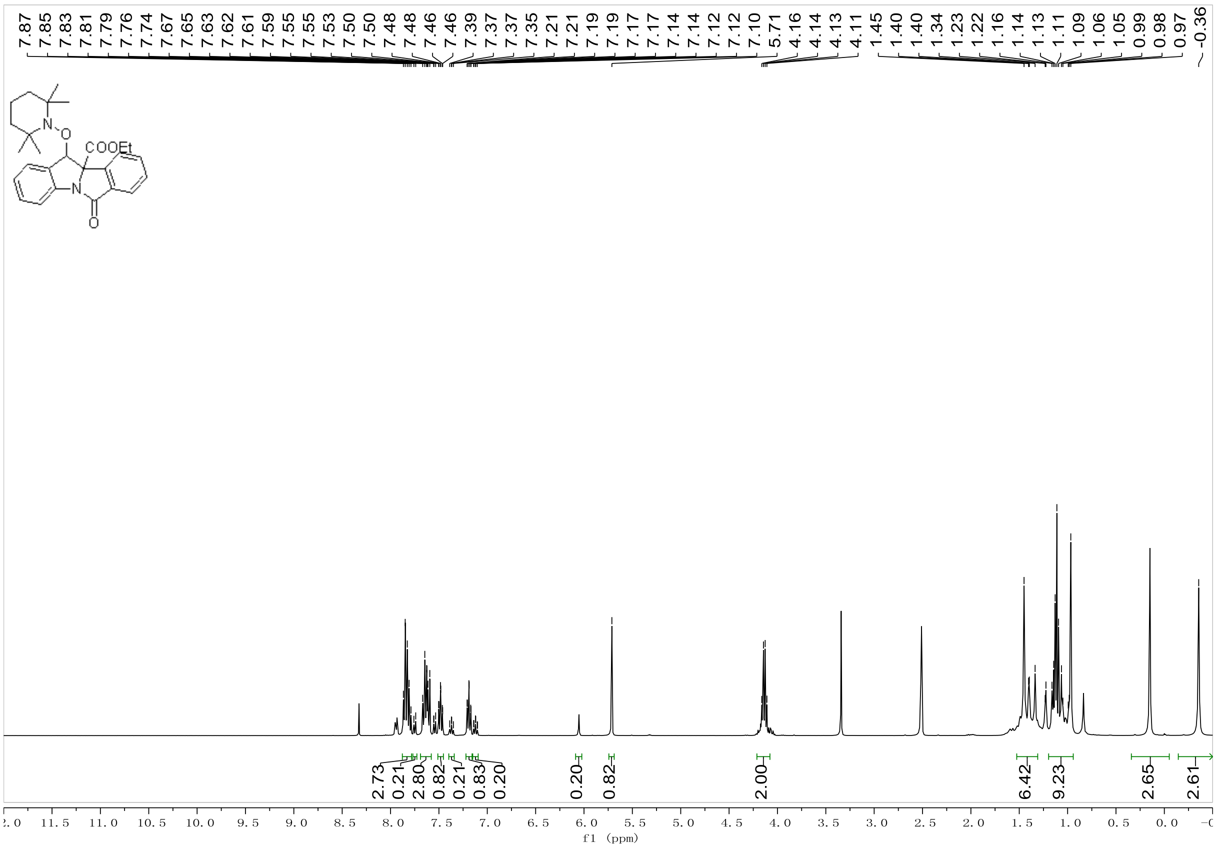

Supplementary Figure 2. ¹H NMR Spectra of compound **43**.

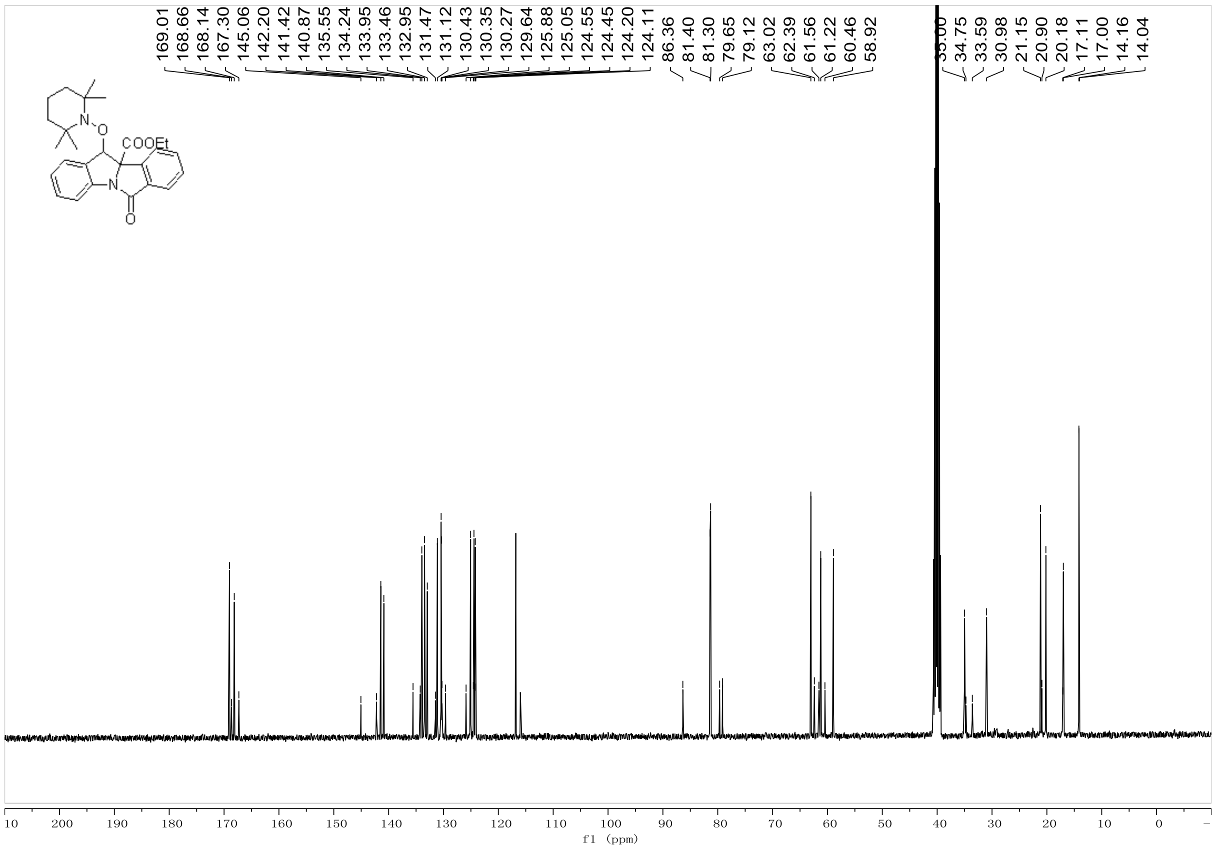

Supplementary Figure 3. ¹³C NMR Spectra of compound **43**.

D-labeling Experiments

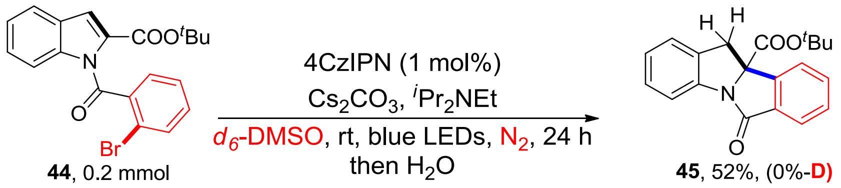

General operation procedure: An oven-dried Schlenk tube (10 mL) containing a stirring bar was charged with the substrate **44** (80 mg, 0.2 mmol, 1.0 equiv) and 4CzIPN (1.6 mg, 0.002 mmol, 1 mol%). The Schlenk tube was then introduced in a glovebox, where it was charged with Cs_2CO_3 (195 mg, 0.6 mmol, 3.0 equiv). The tube was taken out of the glovebox and connected to a vacuum line where it was evacuated and back-filled with N_2 for 3 times. Then d_6-DMSO (2 mL), DIPEA (100 μL, 0.6 mmol, 3.0 equiv) were added under N_2 flow. Finally, the reaction mixture in sealed tube was placed at a distance of 2 ~ 4 cm from a 30 W blue LED and stirred at room temperature (25 ^{o}C) for 24 h. Then, the mixture was quenched with 1 mL of H_2O, 2 mL EtOAc, and extracted with EtOAc, then concentrated in vacuo. The residue was purified by silica gel flash chromatography to give **45** in 52% isolated yield without deuterium incorporation. **^{1}H NMR** (400 MHz, $CDCl_3$) δ 7.88–7.86 (d, J = 8.0 Hz, 1H), 7.72–7.70 (d, J = 8.0 Hz, 1H), 7.64–7.60 (m, 2H), 7.54–7.50 (m, 1H), 7.31–7.26 (m, 1H), 7.22–7.20 (m, 1H), 7.09–7.05 (m, 1H), 3.88 (d, J = 16.0 Hz, 1H), 3.29 (d, J = 16.0 Hz, 1H), 1.35 (s, 9H); **^{13}C NMR** (101 MHz, $CDCl_3$) δ 169.8, 168.2, 144.9, 140.1, 134.2, 133.4, 132.9, 129.5, 128.1, 125.0, 124.9, 124.6, 122.9, 116.6, 83.1, 37.8, 27.6; HRMS calcd for $C_{20}H_{19}NNaO_3$ $[M+Na]^+$: 344.1263, found for: 344.1258.

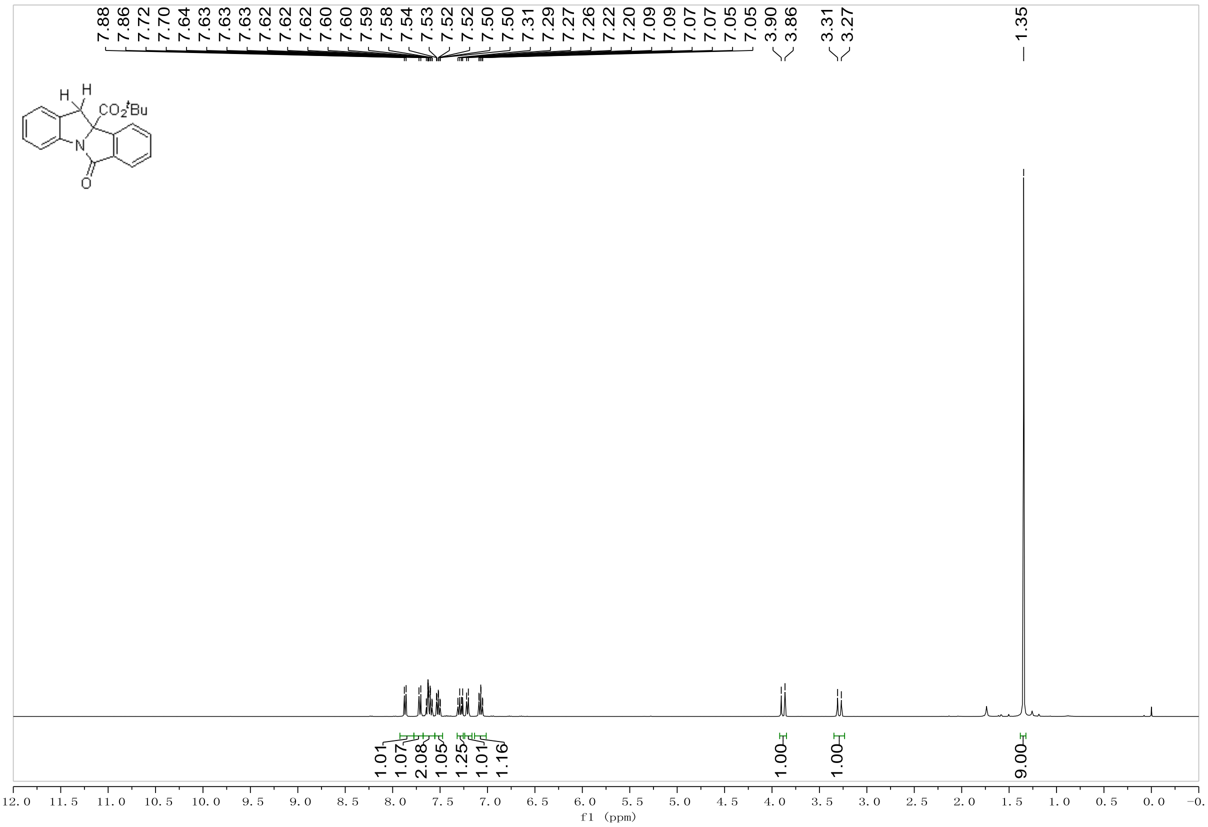

Supplementary Figure 4. ^{1}H NMR Spectra of compound **45**.

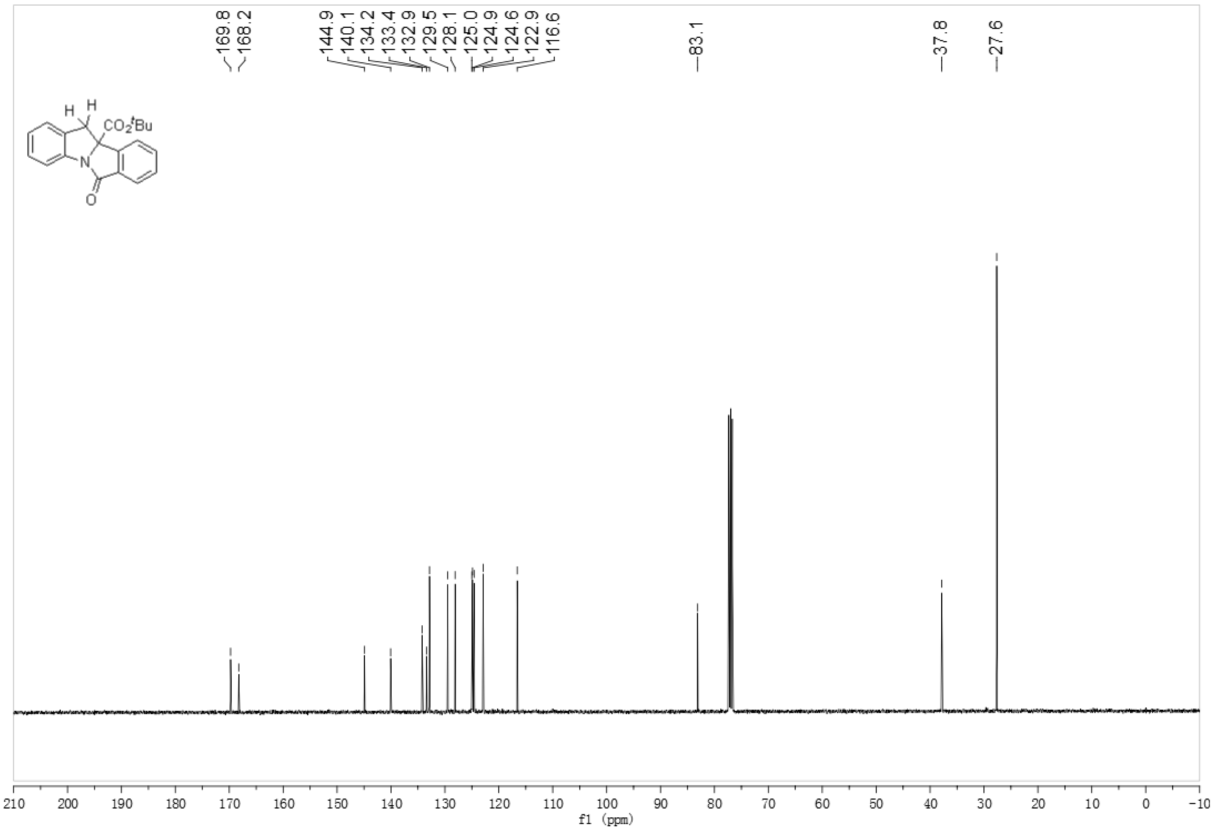

Supplementary Figure 5. ^{13}C NMR Spectra of compound **45**.

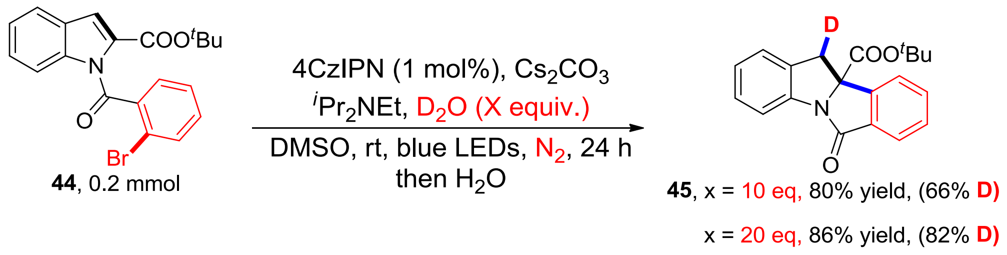

Following the general procedure, the reaction with **44** (80 mg, 0.2 mmol, 1.0 equiv), 4CzIPN (1.6 mg, 0.002 mmol, 1 mol%), Cs_2CO_3 (195 mg, 0.6 mmol, 3.0 equiv), DIPEA (100 μL, 0.6 mmol, 3.0 equiv), DMSO (2 mL) and D_2O (40 μL, 2.0 mmol, 10.0 equiv) under N_2 for 24 h at 25 ℃ afforded **45** as a white solid in 80% yield and 66% deuterium incorporation.

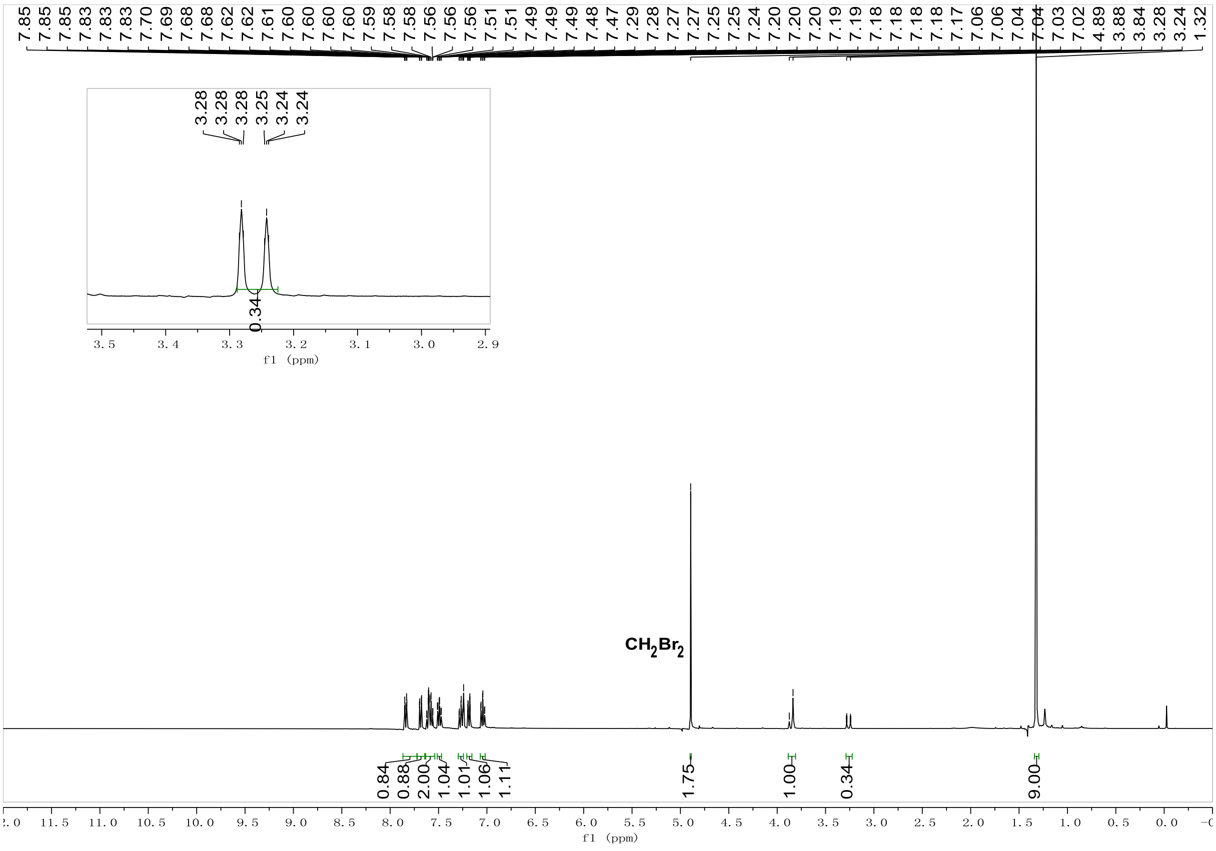

Supplementary Figure 6. ^{1}H NMR Spectra of compound **45**.

Following the general procedure, the reaction with **44** (80 mg, 0.2 mmol, 1.0 equiv), 4CzIPN (1.6 mg, 0.002 mmol, 1 mol%), Cs_2CO_3 (195 mg, 0.6 mmol, 3.0 equiv), DIPEA (100 μL, 0.6 mmol, 3.0 equiv), DMSO (2 mL) and D_2O (80 μL, 4.0 mmol, 20.0 equiv) under N_2 for 24 h at 25 ℃ afforded **45** as a white solid in 86% yield and 82% deuterium incorporation.

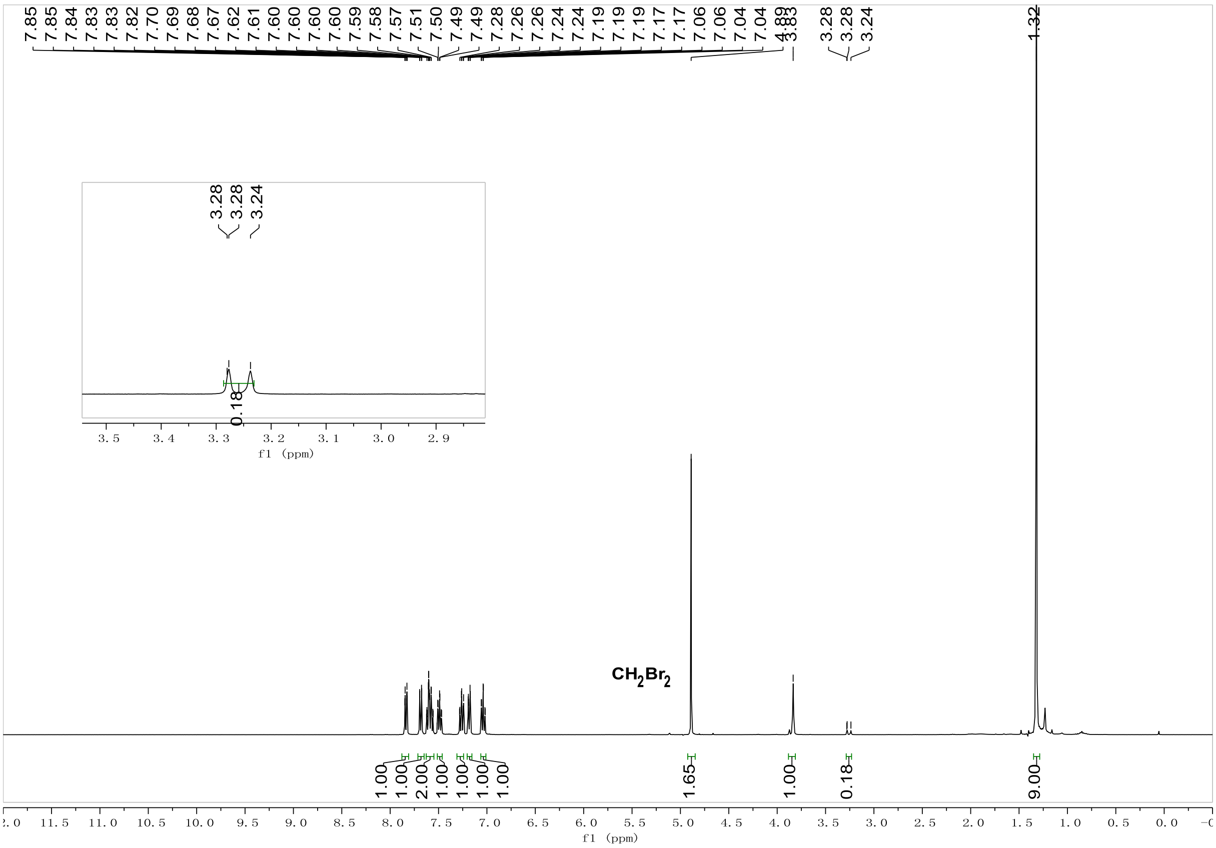

Supplementary Figure 7. ^{1}H NMR Spectra of compound **45**.

Reaction of aldehyde

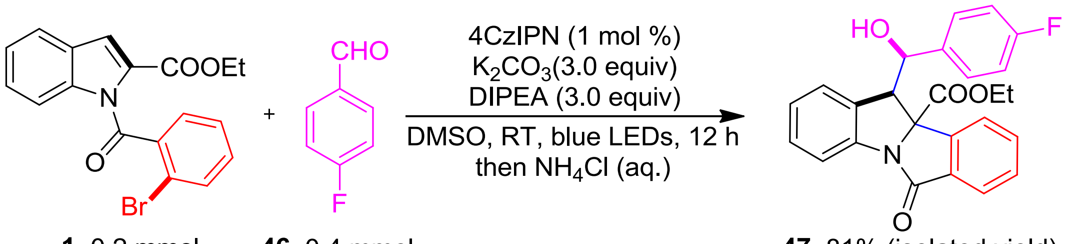

1, 0.2 mmol **46**, 0.4 mmol **47**, 81% (isolated yield)

General operation procedure: An oven-dried Schlenk tube (10 mL) containing a stirring bar was charged with the substrate **1** (0.2 mmol, 74.2 mg, 1.0 equiv) and 4CzIPN (0.002 mmol, 1.6 mg, 1 mol%). the Schlenk tube was then introduced in a glovebox, where it was charged with K_2CO_3 (0.6 mmol, 82.9 mg, 3.0 equiv). The tube was taken out of the glovebox and connected to a vacuum line where it was evacuated and back-filled with N_2 for 3 times. Then DMSO (2 mL), 4-fluorobenzaldehyde (0.4 mmol, 43 μL, 2.0 equiv), DIPEA (0.6 mmol, 100 μL, 3.0 equiv) were added under N_2 flow. Finally, the reaction mixture in sealed tube was placed at a distance of 2 ~ 4 cm from a 30 W blue LED and stirred at room temperature (25 ^{o}C) for 12 h. Then, the mixture was quenched with 1 mL of H_2O, 2 mL EtOAc, and extracted with EtOAc,

then concentrated in vacuo. The residue was purified by silica gel flash chromatography to give **47** in 81% isolated yield (d.r. = 1.3:1). **^{1}H NMR** (400 MHz, DMSO-d_6) δ 7.72 – 7.51 (m, 4H), 7.41 (d, *J* = 7.8 Hz, 1H), 7.30 (t, *J* = 7.3 Hz, 1H), 7.16 – 6.94 (m, 2H), 6.69 (t, *J* = 8.7 Hz, 2H), 6.36 – 6.16 (m, 2H), 5.51 (d, *J* = 3.6 Hz, 1H), 4.51 – 4.37 (m, 1H), 4.33 (d, *J* = 5.4 Hz, 1H), 4.04 (q, *J* = 6.9 Hz, 2H), 1.03 (t, *J* = 7.0 Hz, 3H). HRMS calcd for $C_{25}H_{20}FNO_4$ $[M+Na]^+$: 440.1269, found for: 440.1266.

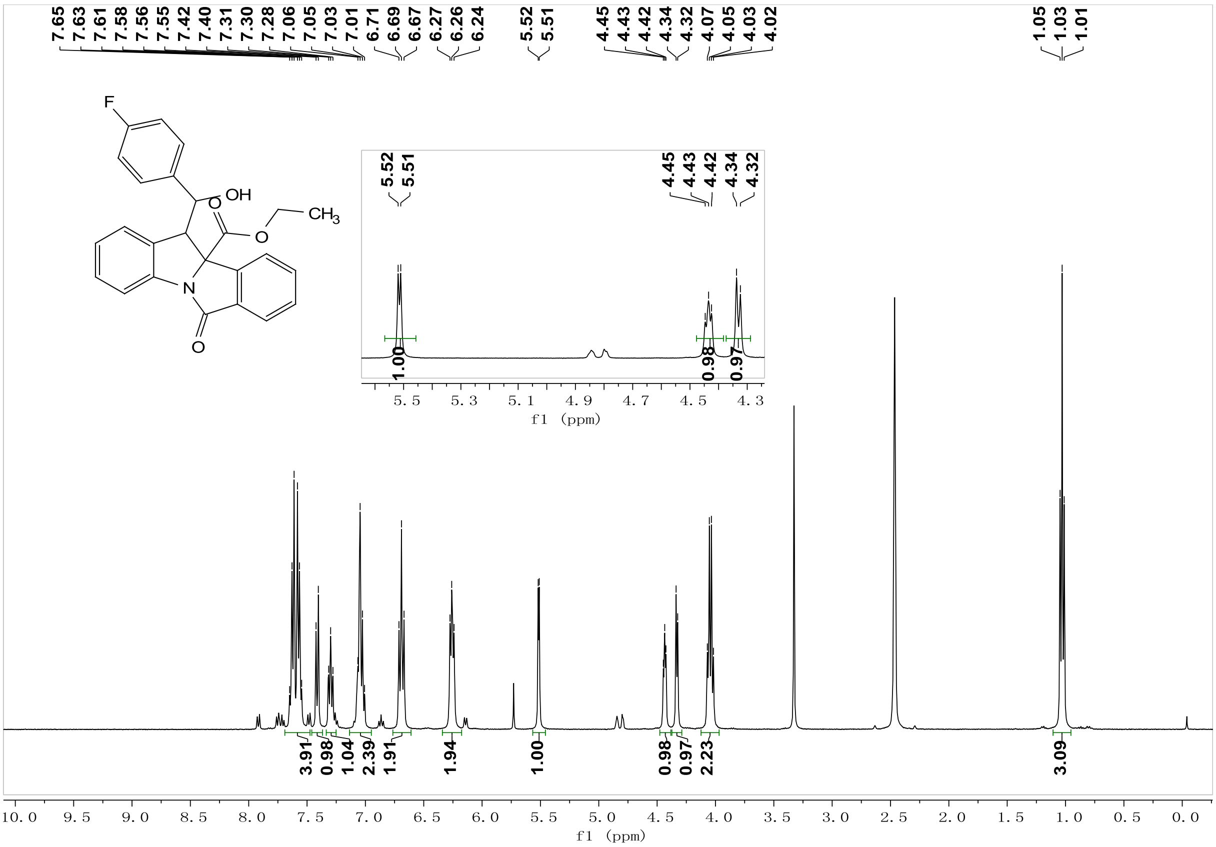

Supplementary Figure 8. ^{1}H NMR Spectra of compound **47**.

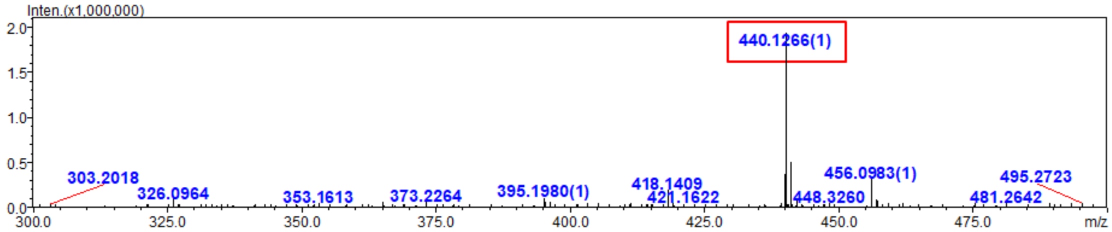

Supplementary Figure 9. HRMS Spectra of compound **47**.

Stern-Volmer emission quenching

Fluorescence quenching experiments were measured on a RF-5301PC Spectrofluorophotometer with a

4 mL quartz cuvette with a cap. Anhydrous DMF was degassed by N_2 bubbling for 30 minutes before using. 4CzIPN was irradiated at 440 nm and the emission intensity at about 536 nm was observed. In a typical experiment, the emission spectrum of a 5*10^{-5} M solution of 4CzIPN in DMF was collected.

DIPEA: A stock solution of DIPEA (0.5 M) was prepared. Then, different amounts of this stock solution were added to 2.5 mL of 4CzIPN in DMF (5*10^{-5} M).

Ethyl 1-(2-bromobenzoyl)-1H-indole-2-carboxylate 1: A stock solution of 1 (0.5 M) was prepared. Then, different amounts of this stock solution were added to 2.5 mL of 4CzIPN in DMF (5*10^{-5} M).

1 + Cs_2CO_3: A stock solution of 2 (185.5 mg, 0.5 mmol) with Cs_2CO_3 (82 mg, 0.25 mmol) in 1 mL of DMF was prepared. Then, different amounts of this stock solution were added to 2.5 mL of 4CzIPN in DMF (5*10^{-5} M).

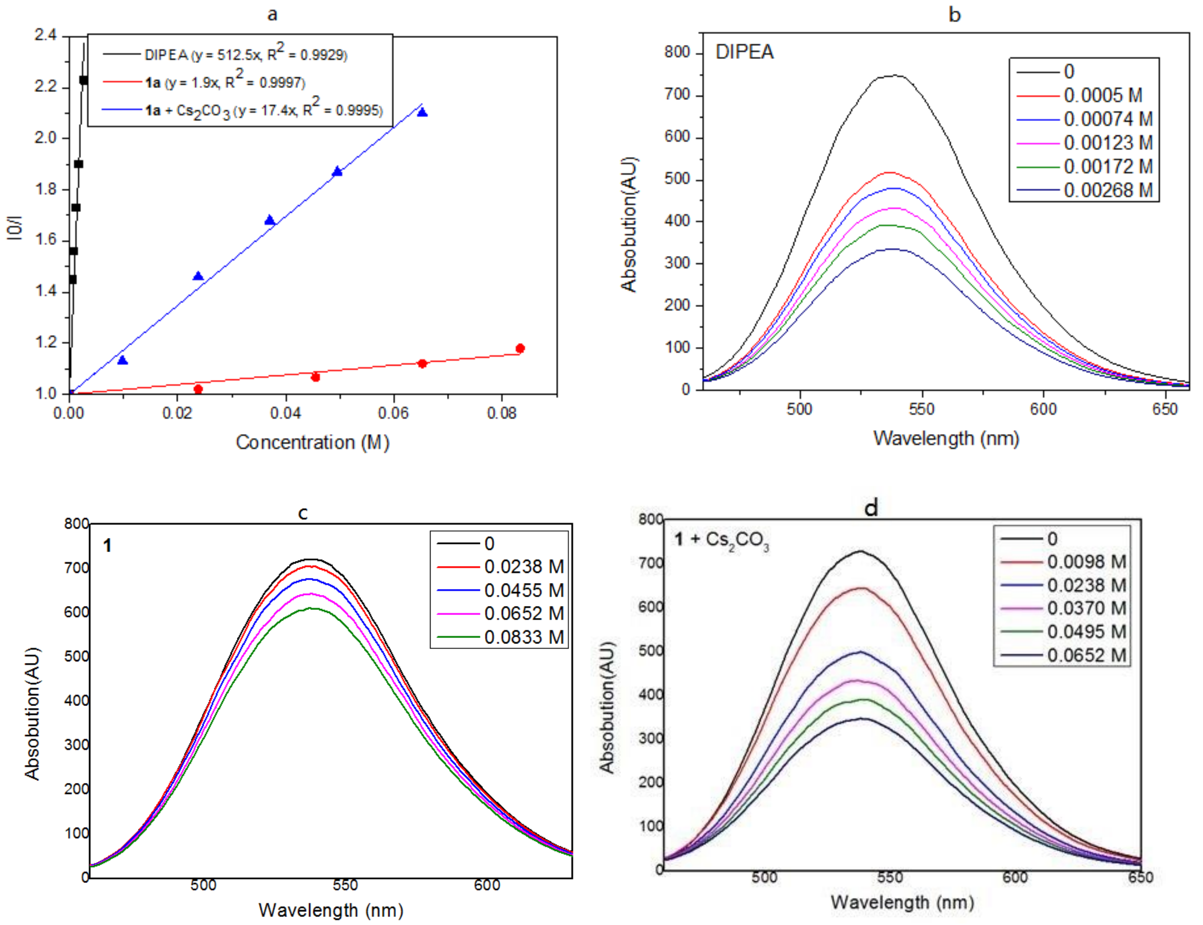

Supplementary Figure 10. Optical experiment with fluorescence spectrum. a Stern－Volmer fluorescence quenching experiments using 4CzIPN with DIPEA, **1** as well as **1** and Cs_2CO_3. b Steady-state Stern－Volmer experiment of 4CzIPN and DIPEA. c Steady-state Stern－Volmer experiment of **1**. d Steady-state Stern－Volmer experiment of **1** and Cs_2CO_3.

The luminescence of 4CzIPN at λ_{max} = 540 nm was readily quenched by DIPEA with a quenching slope of 512.5, which is much more significant than 1 (1.9) and 1 in presence of Cs_2CO_3 (17.4). So we hypothesized that DIPEA ($E_{1/2}^{Ox}$ = +0.63 V vs SCE in DMF)[7] could reductively quenched the photo-exited 4CzIPN ($E_{1/2}$ [4CzIPN/4CzIPN$^{\bullet-}$] = -1.21 V vs. SCE)[8], which was the initial step.

NMR spectra

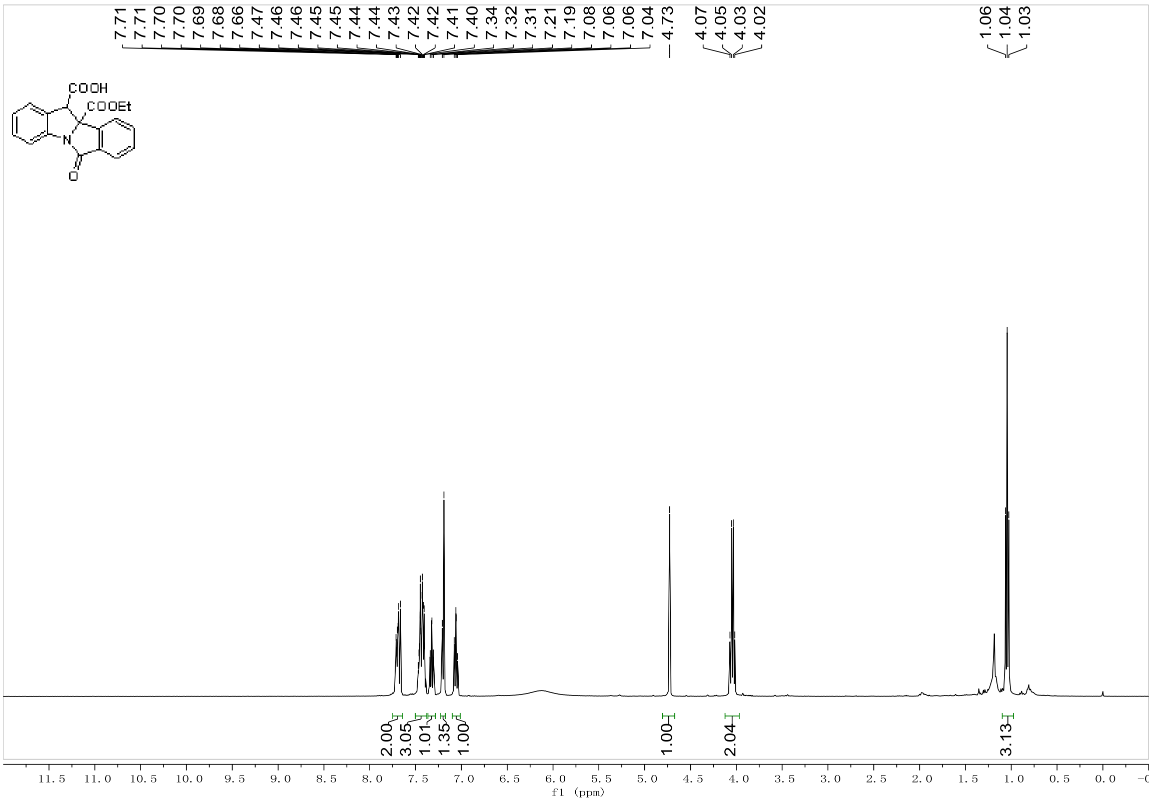

Supplementary Figure 11. ^{1}H NMR Spectra of compound **2**.

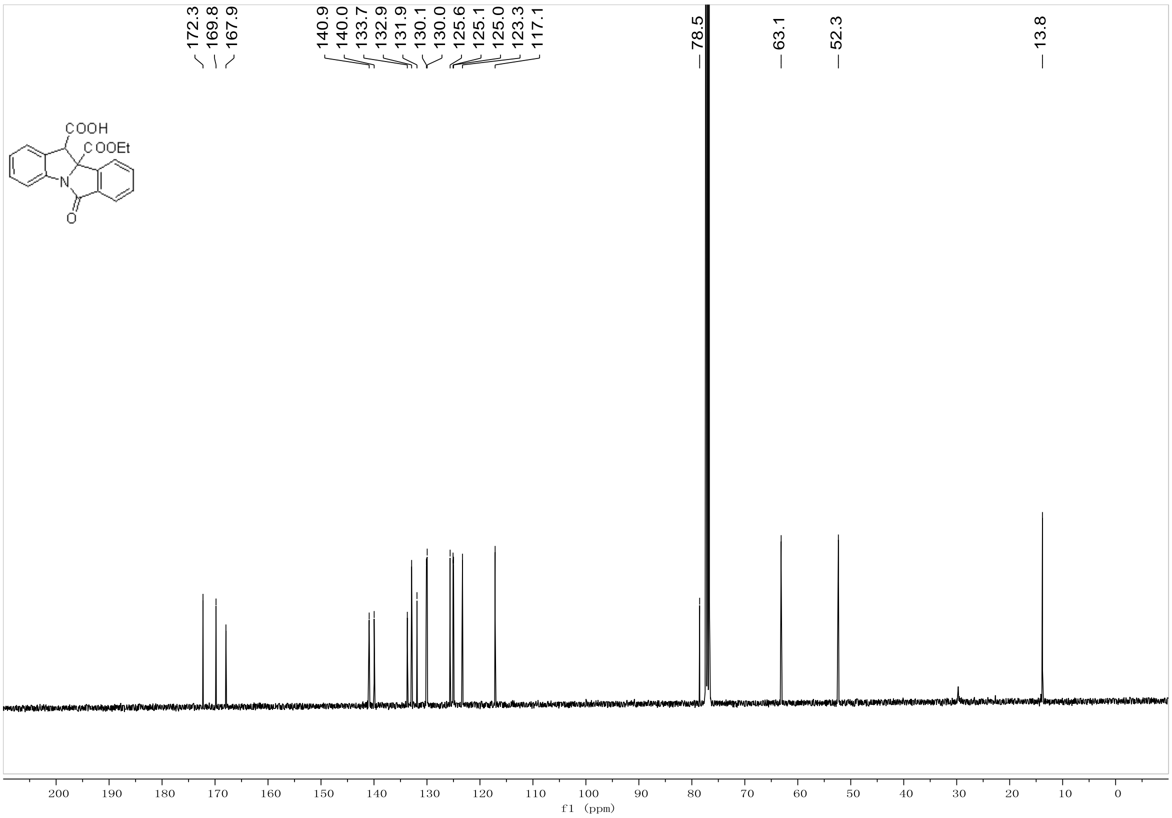

Supplementary Figure 12. ^{13}C NMR Spectra of compound **2**.

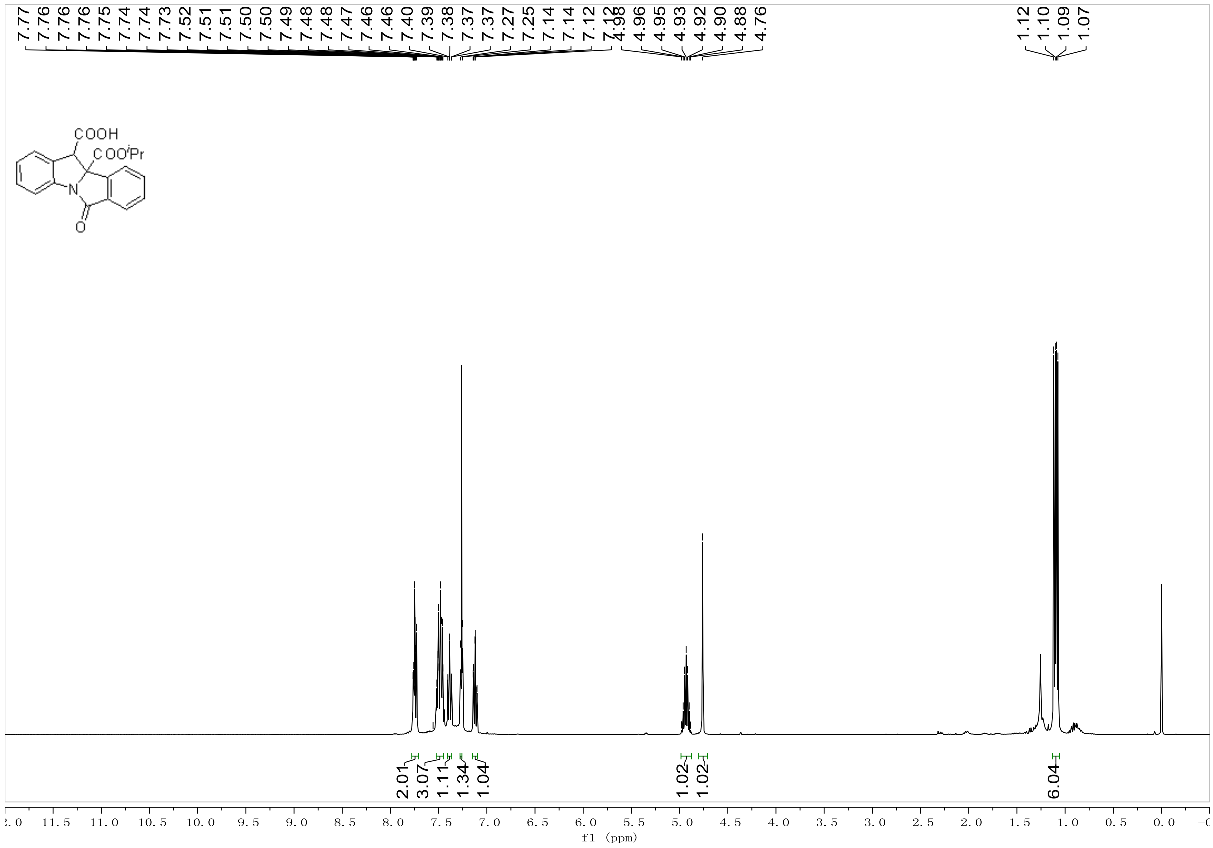

Supplementary Figure 13. ^{1}H NMR Spectra of compound **3**.

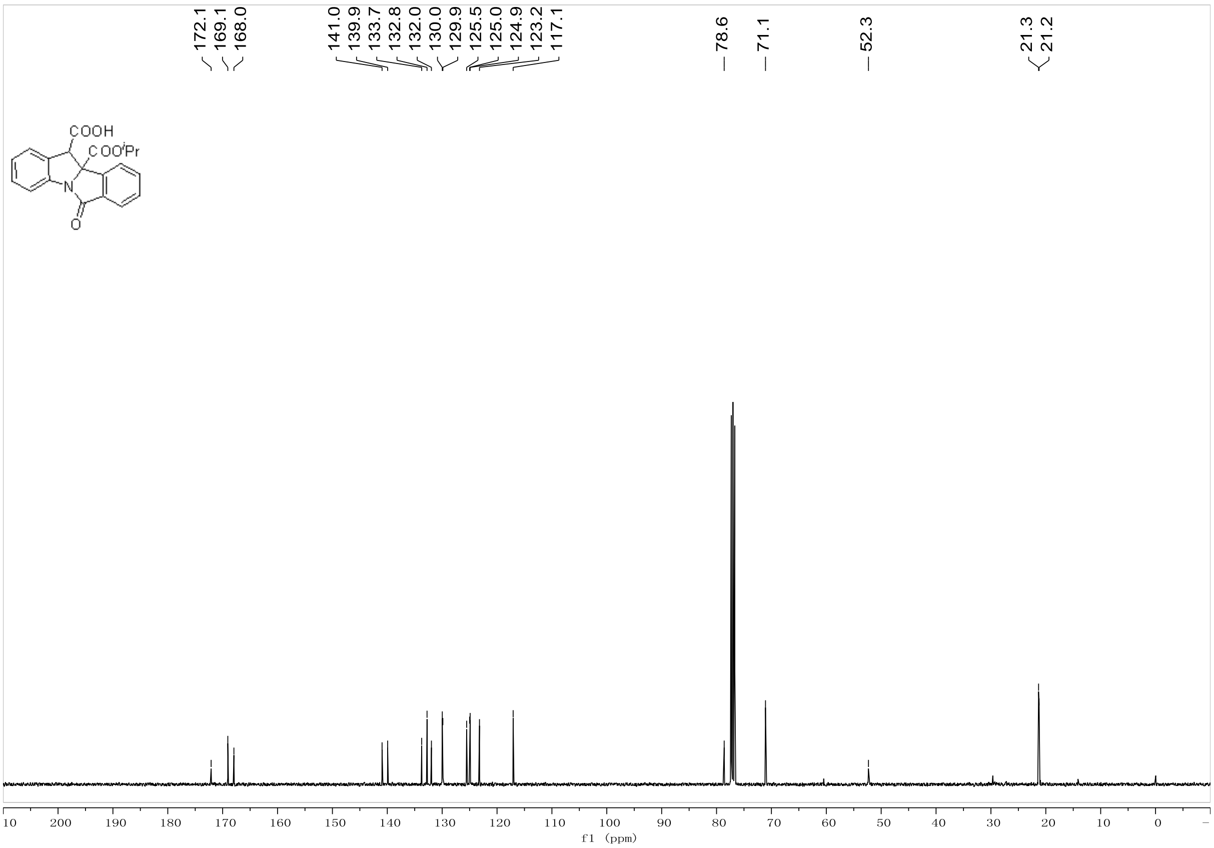

Supplementary Figure 14. ^{13}C NMR Spectra of compound **3**.

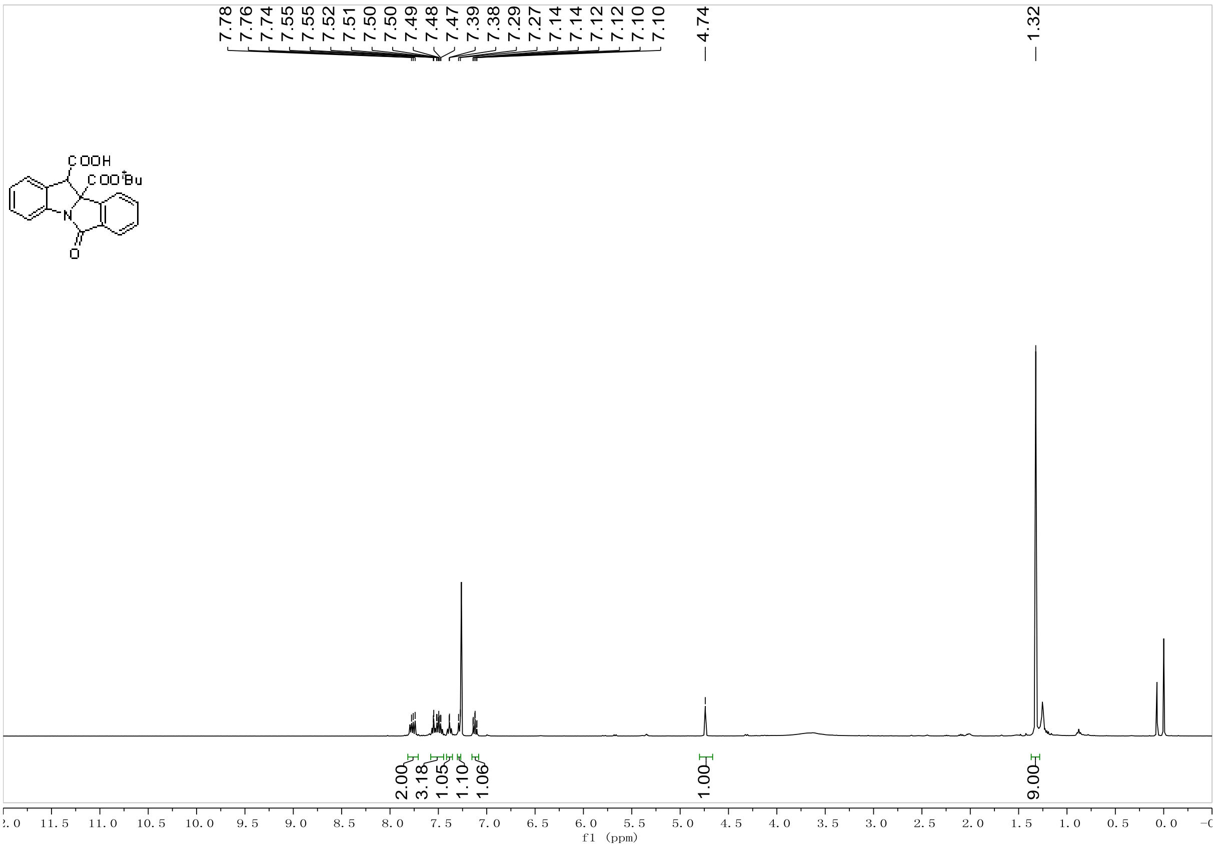

Supplementary Figure 15. ^{1}H NMR Spectra of compound **4**.

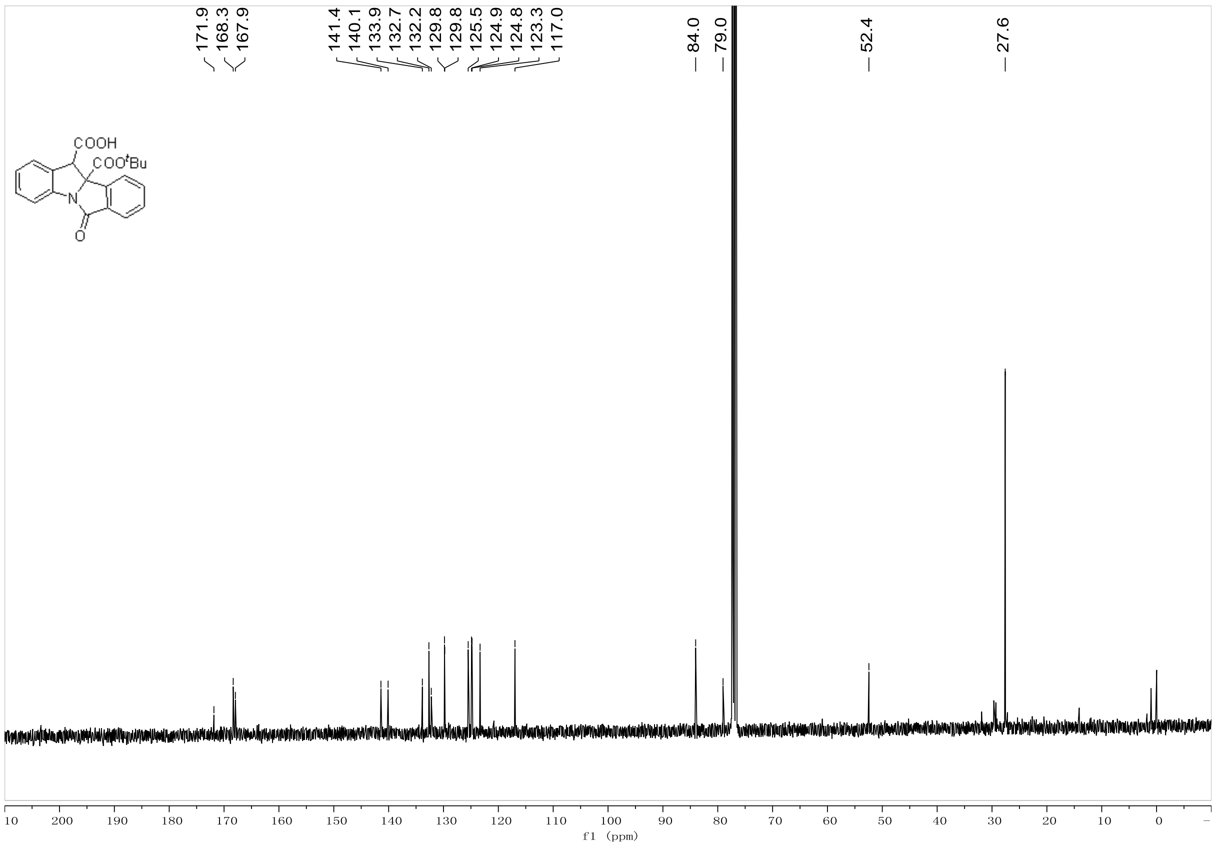

Supplementary Figure 16. ^{13}C NMR Spectra of compound **4**.

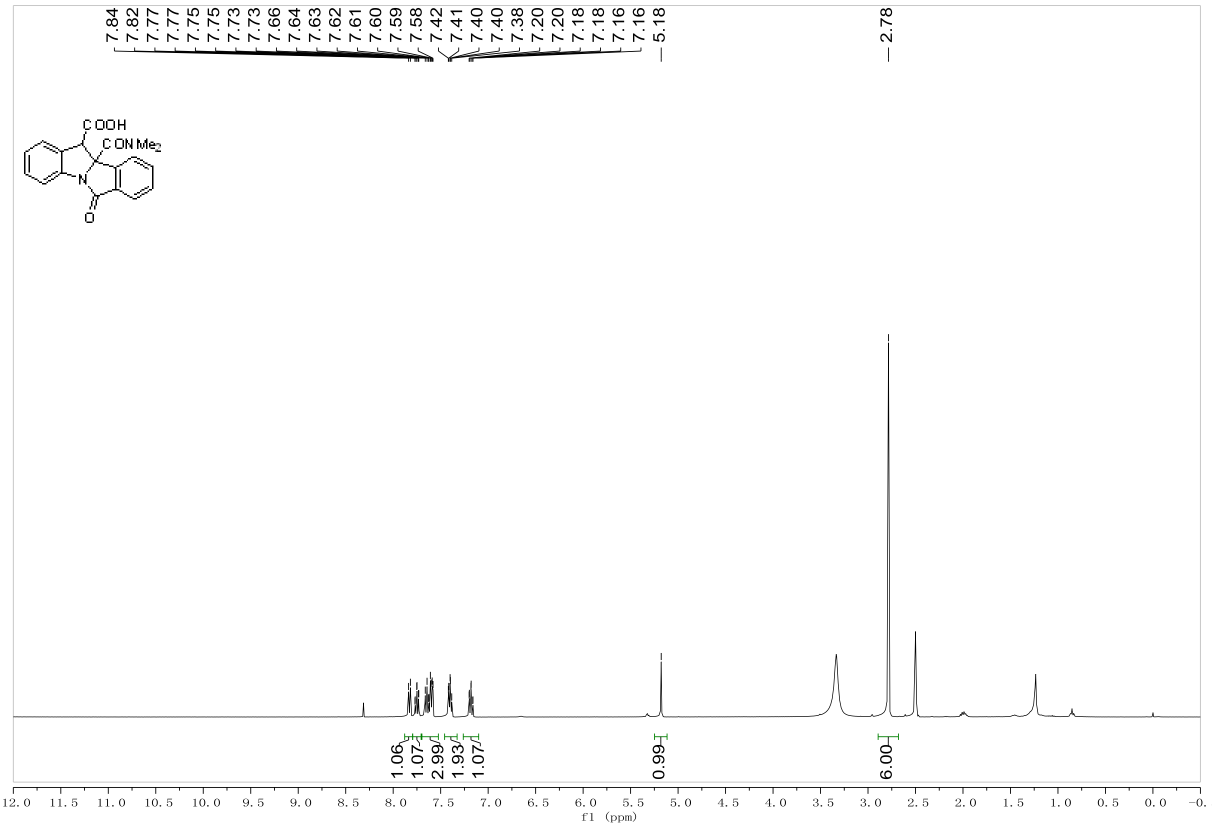

Supplementary Figure 17. ^{1}H NMR Spectra of compound **5**.

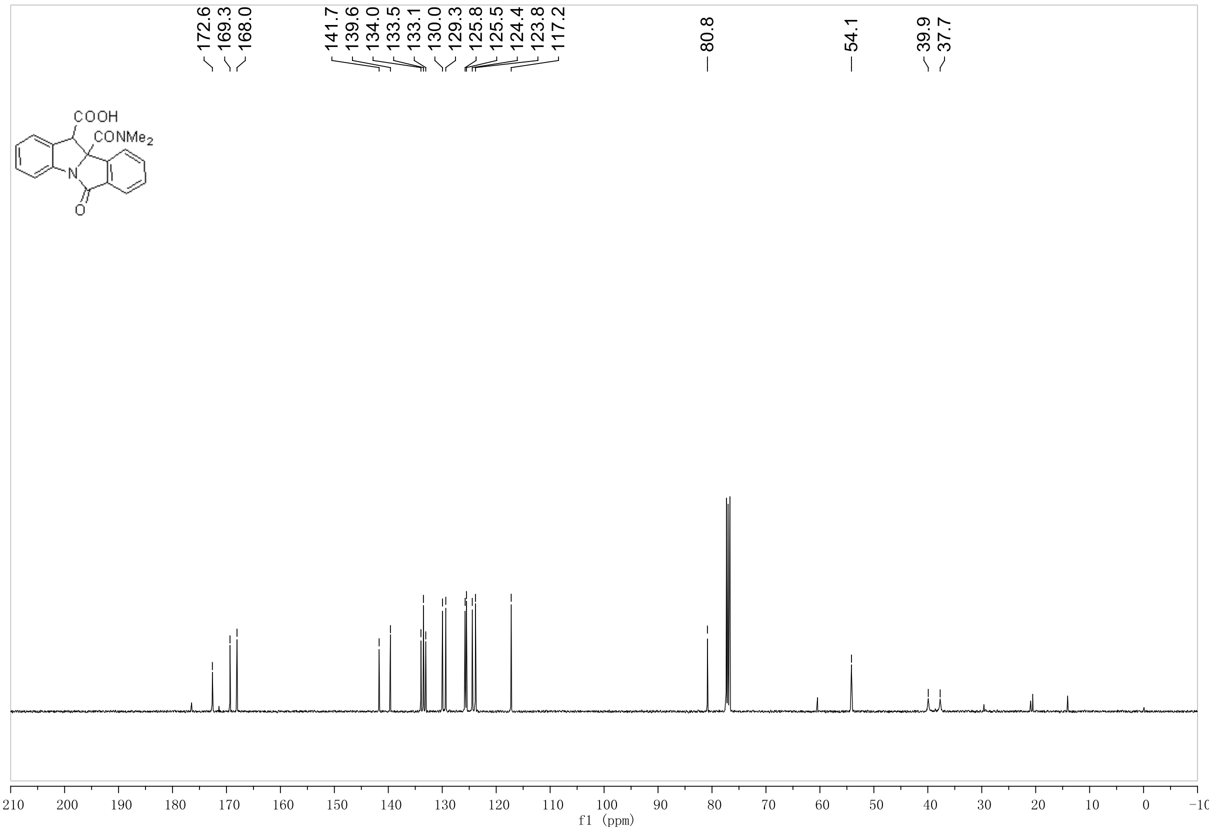

Supplementary Figure 18. ^{13}C NMR Spectra of compound **5**.

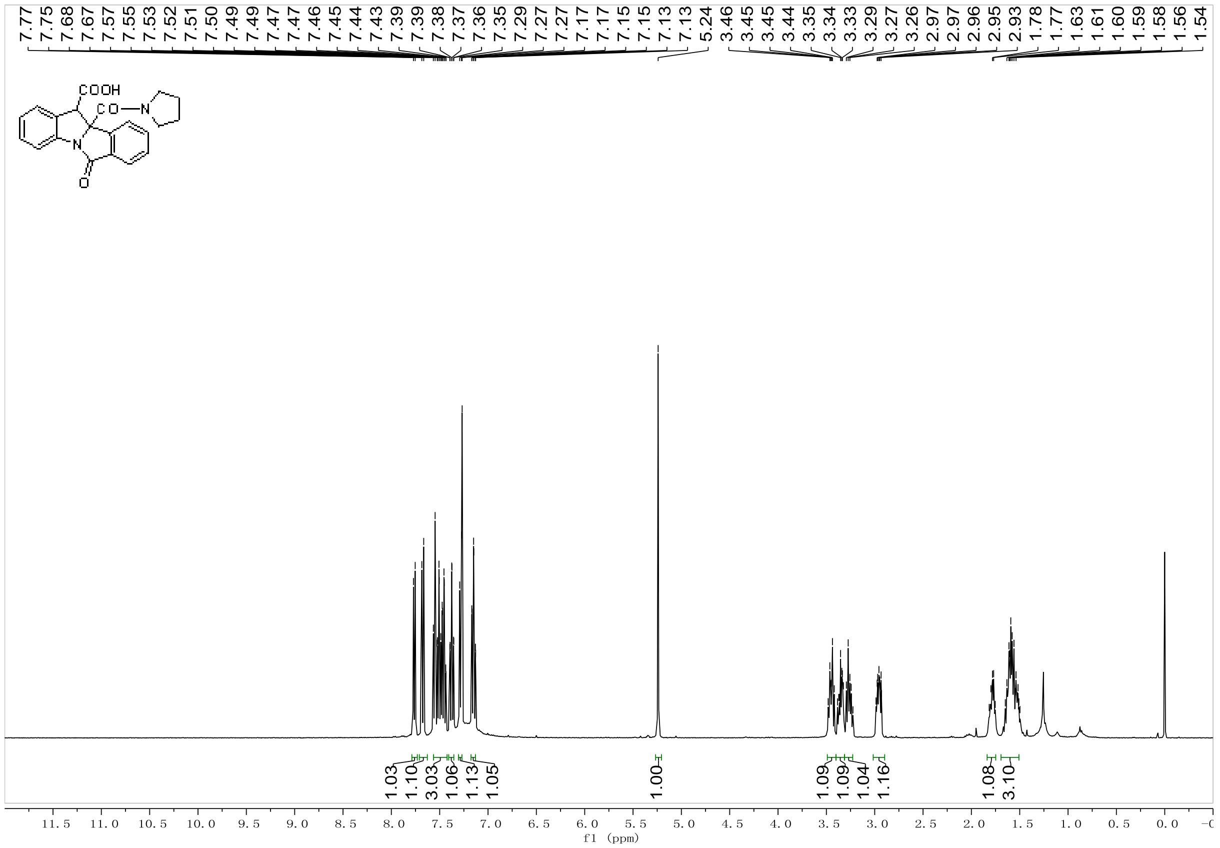

Supplementary Figure 19. ^{1}H NMR Spectra of compound **6**.

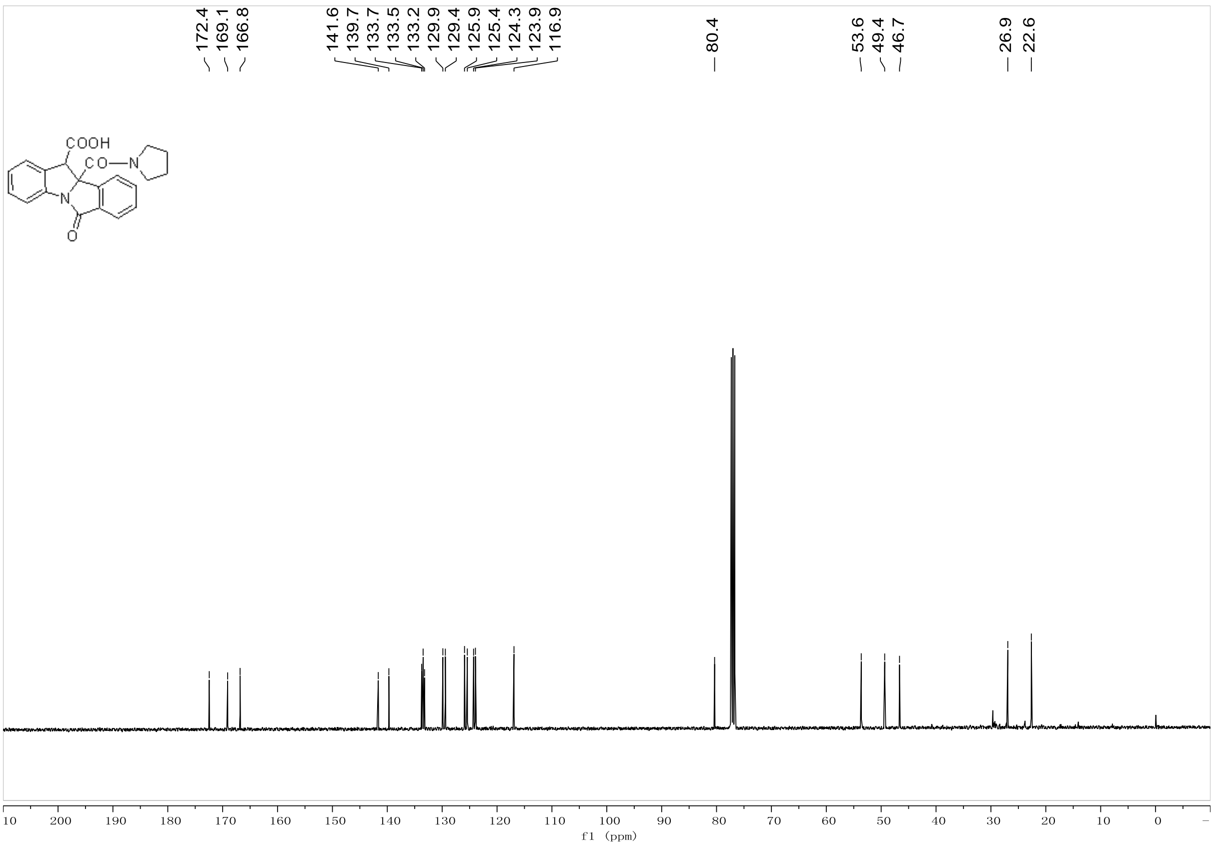

Supplementary Figure 20. ^{13}C NMR Spectra of compound **6**.

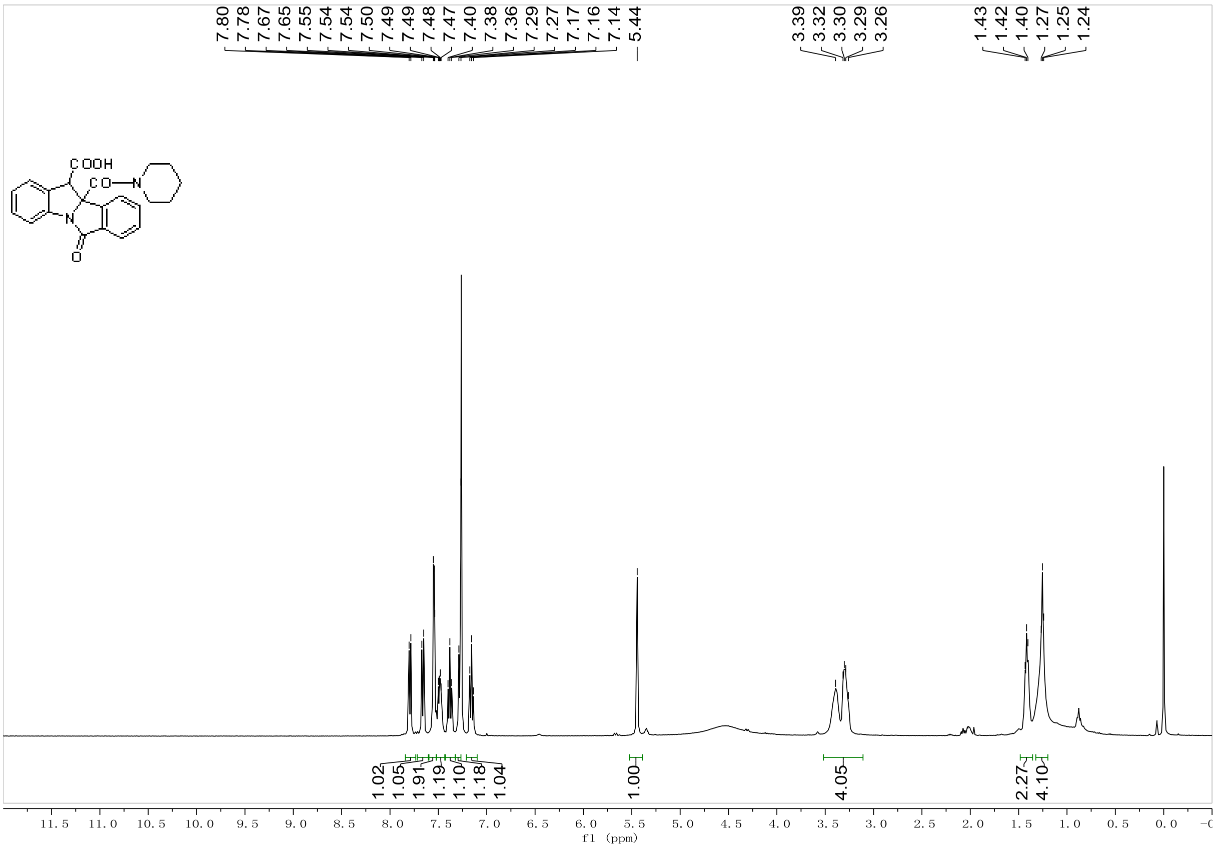

Supplementary Figure 21. ^{1}H NMR Spectra of compound **7**.

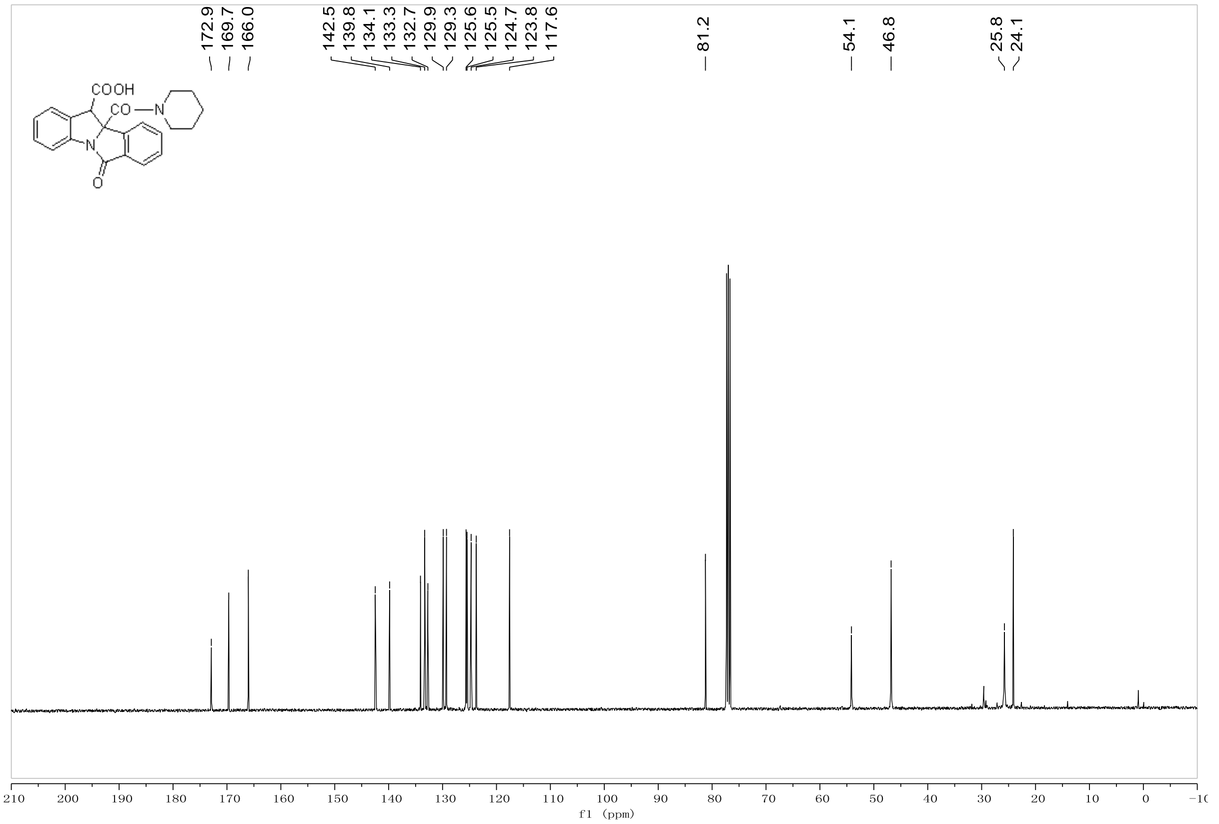

Supplementary Figure 22. ^{13}C NMR Spectra of compound **7**.

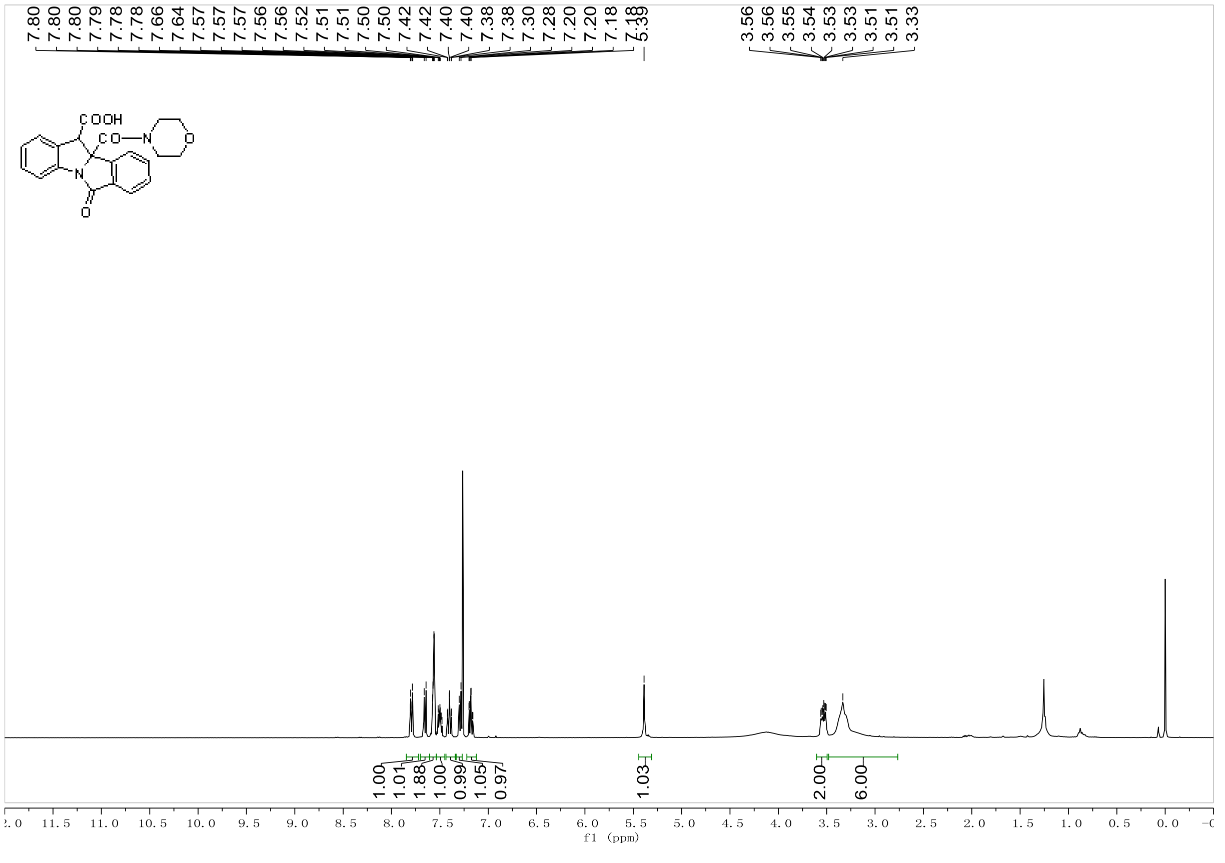

Supplementary Figure 23. ^{1}H NMR Spectra of compound **8**.

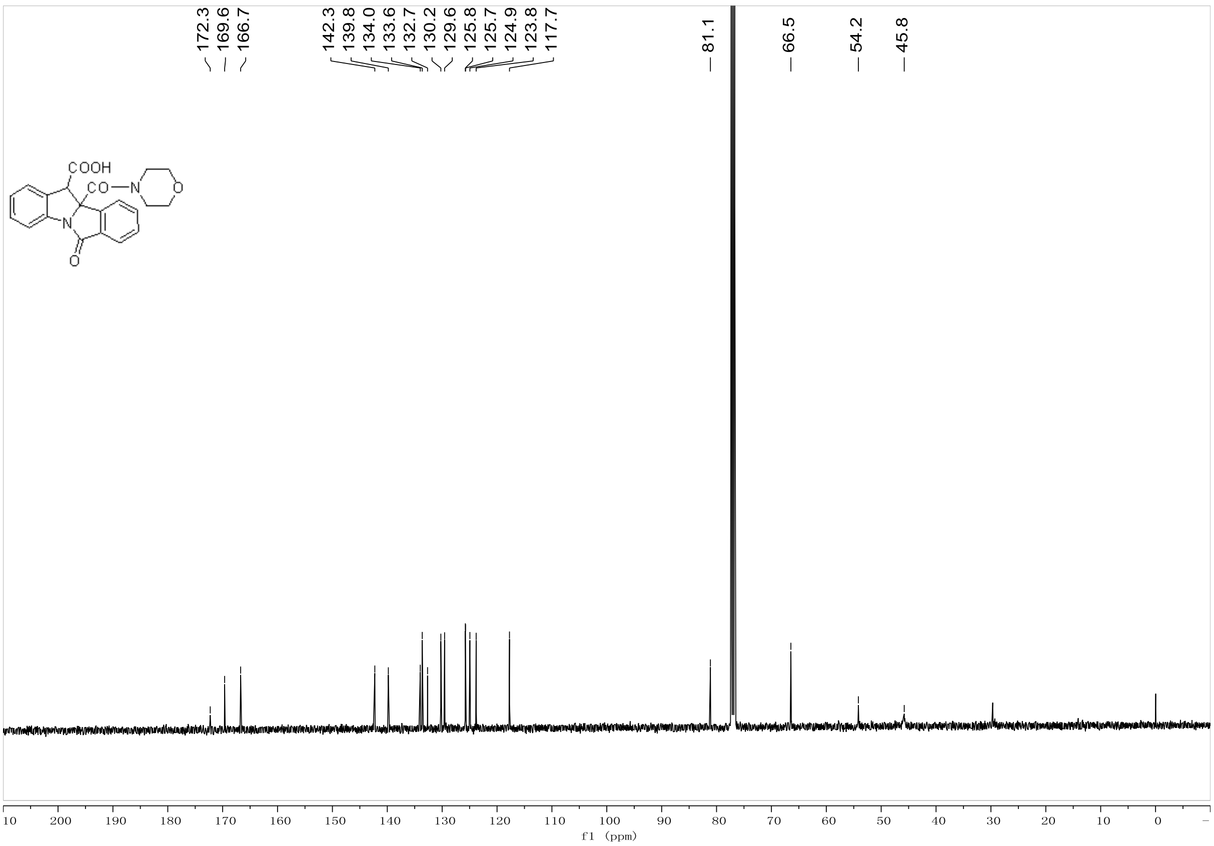

Supplementary Figure 24. ^{13}C NMR Spectra of compound **8**.

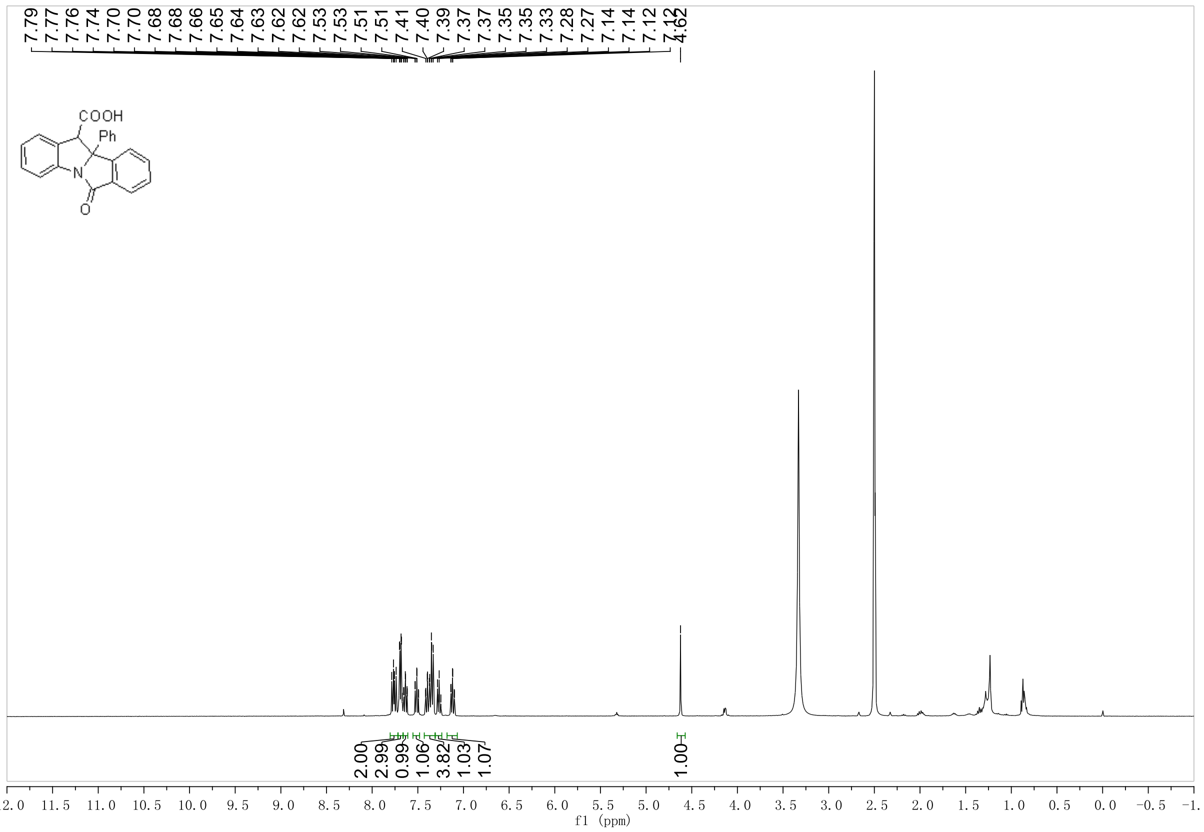

Supplementary Figure 25. ^{1}H NMR Spectra of compound **9**.

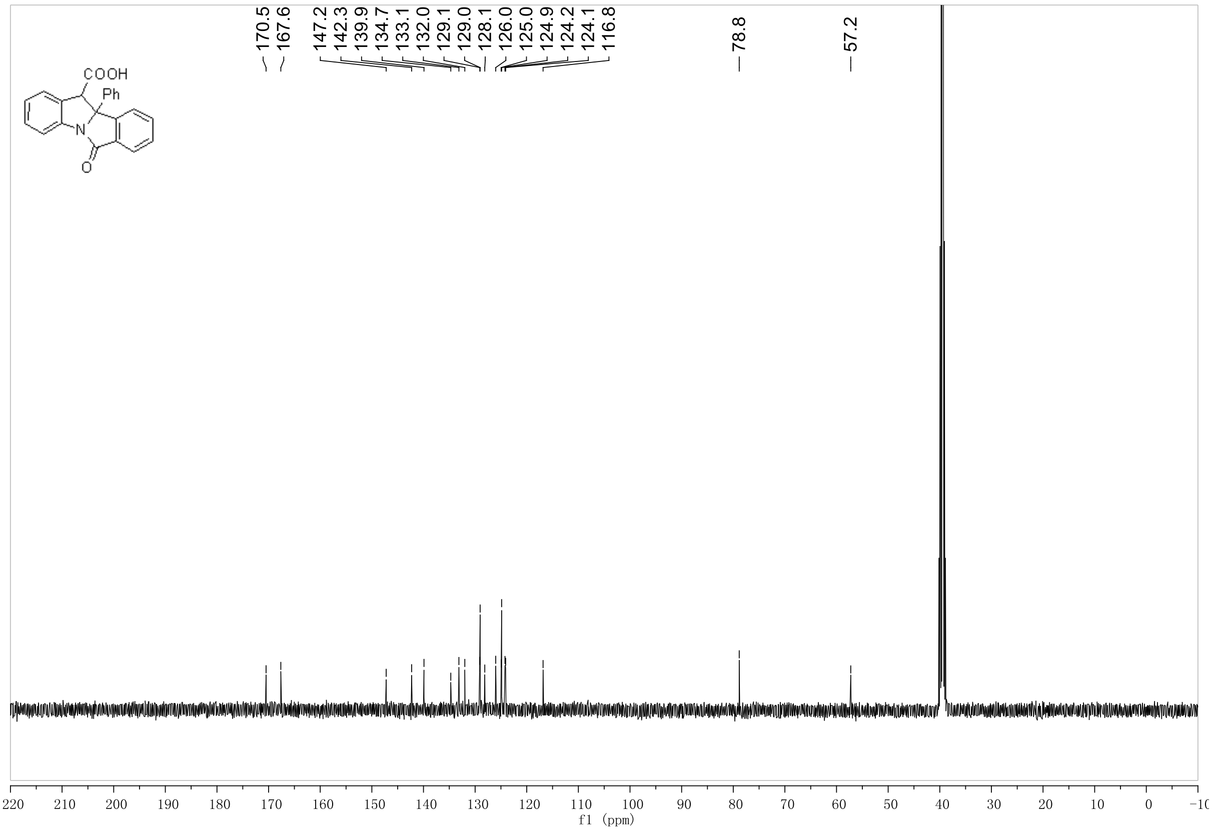

Supplementary Figure 26. ^{13}C NMR Spectra of compound **9**.

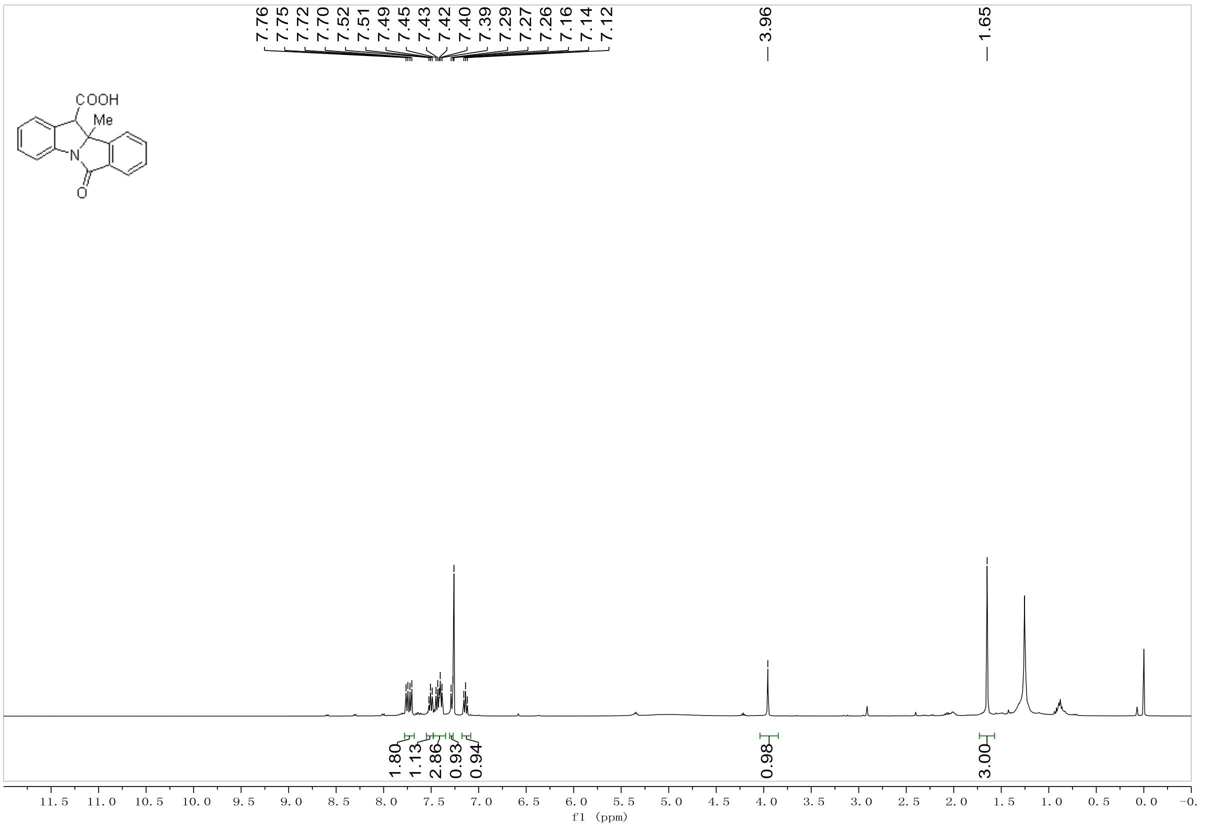

Supplementary Figure 27. ^{1}H NMR Spectra of compound **10**.

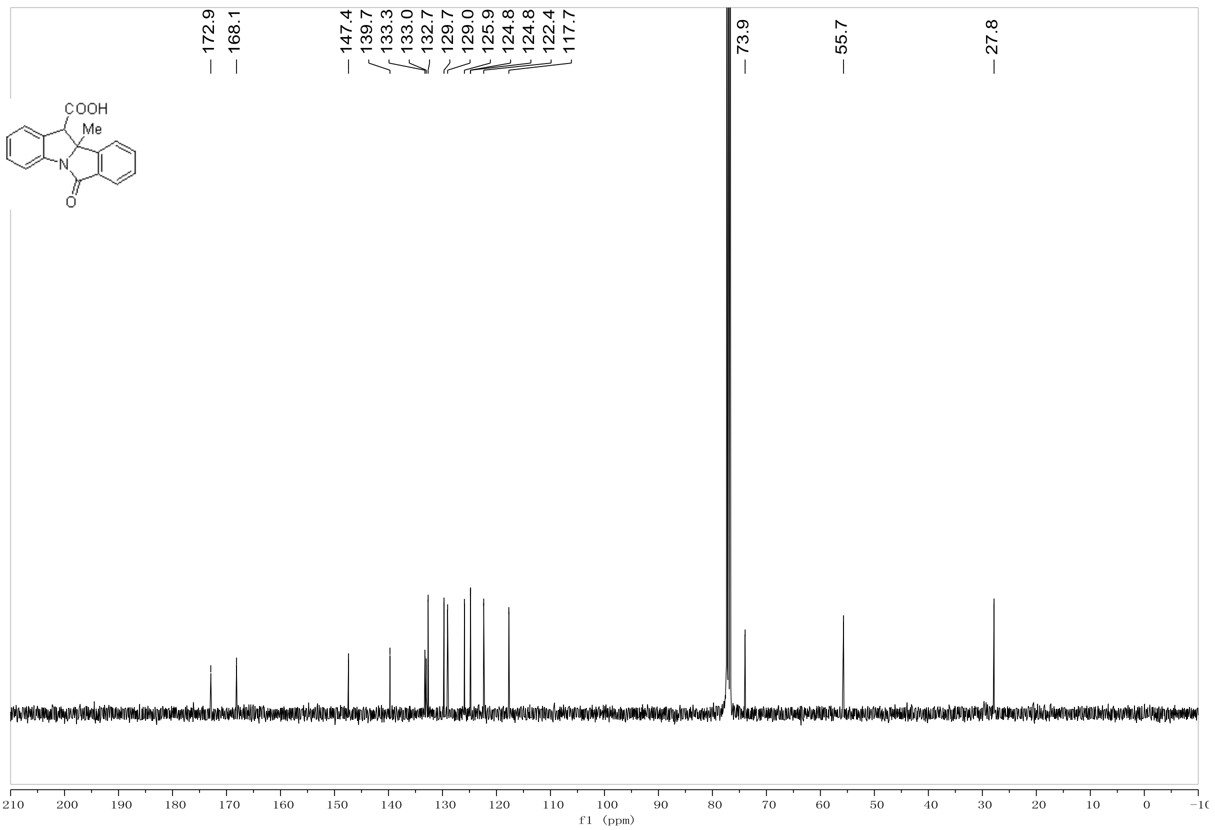

Supplementary Figure 28. ^{13}C NMR Spectra of compound **10**.

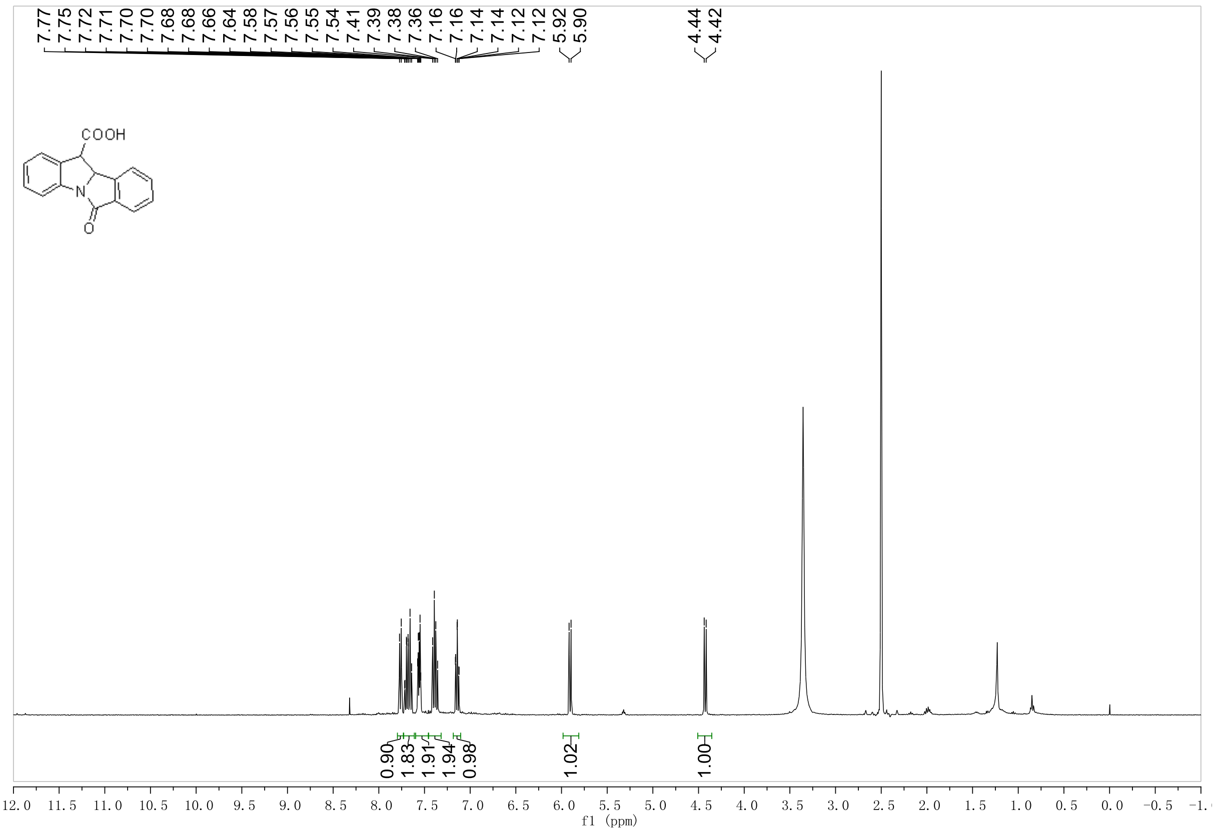

Supplementary Figure 29. ^{1}H NMR Spectra of compound **11**.

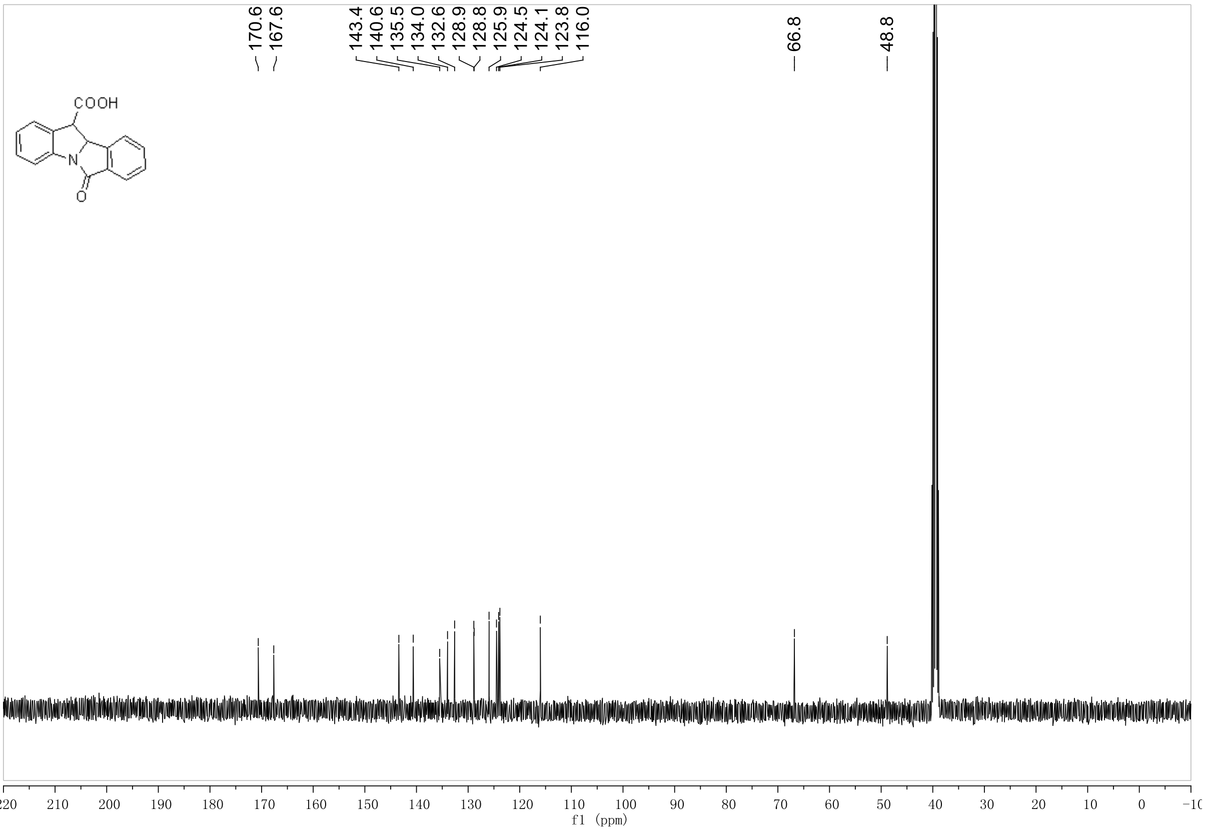

Supplementary Figure 30. ^{13}C NMR Spectra of compound **11**.

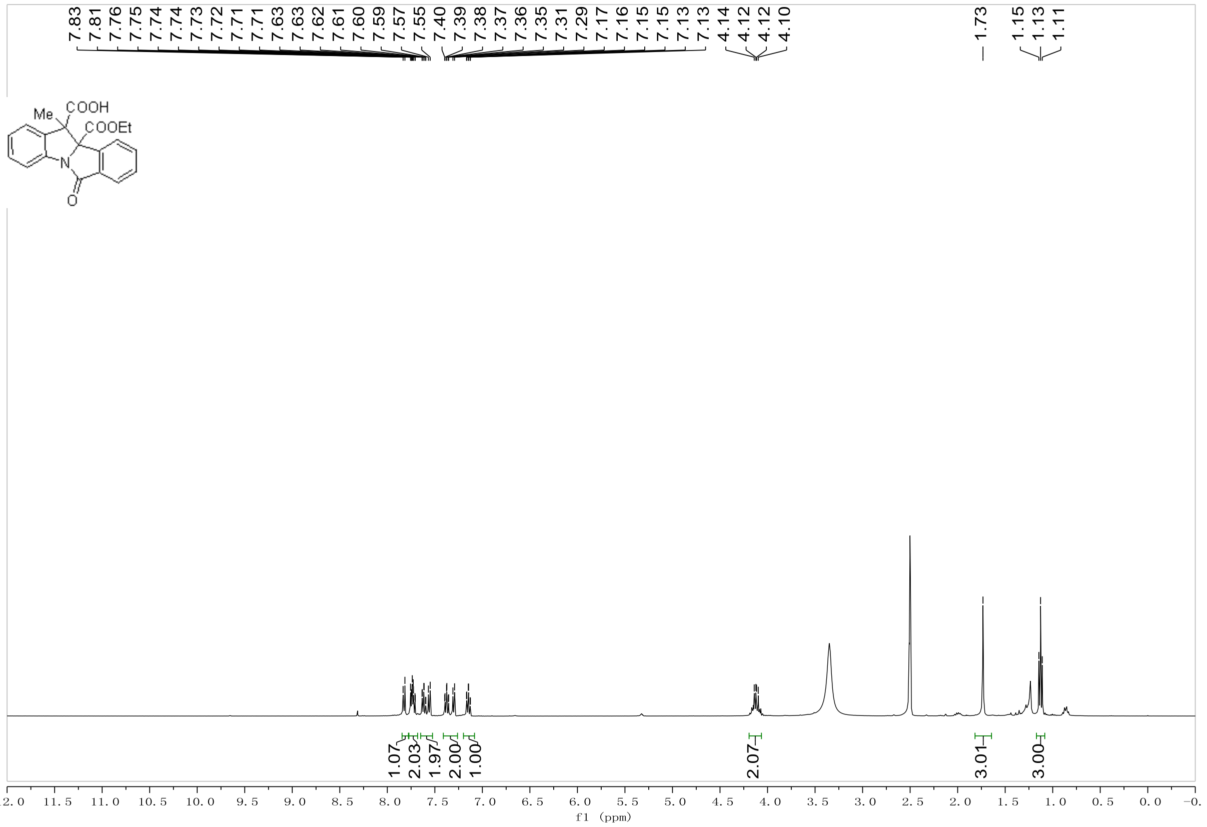

Supplementary Figure 31. ^{1}H NMR Spectra of compound **12**.

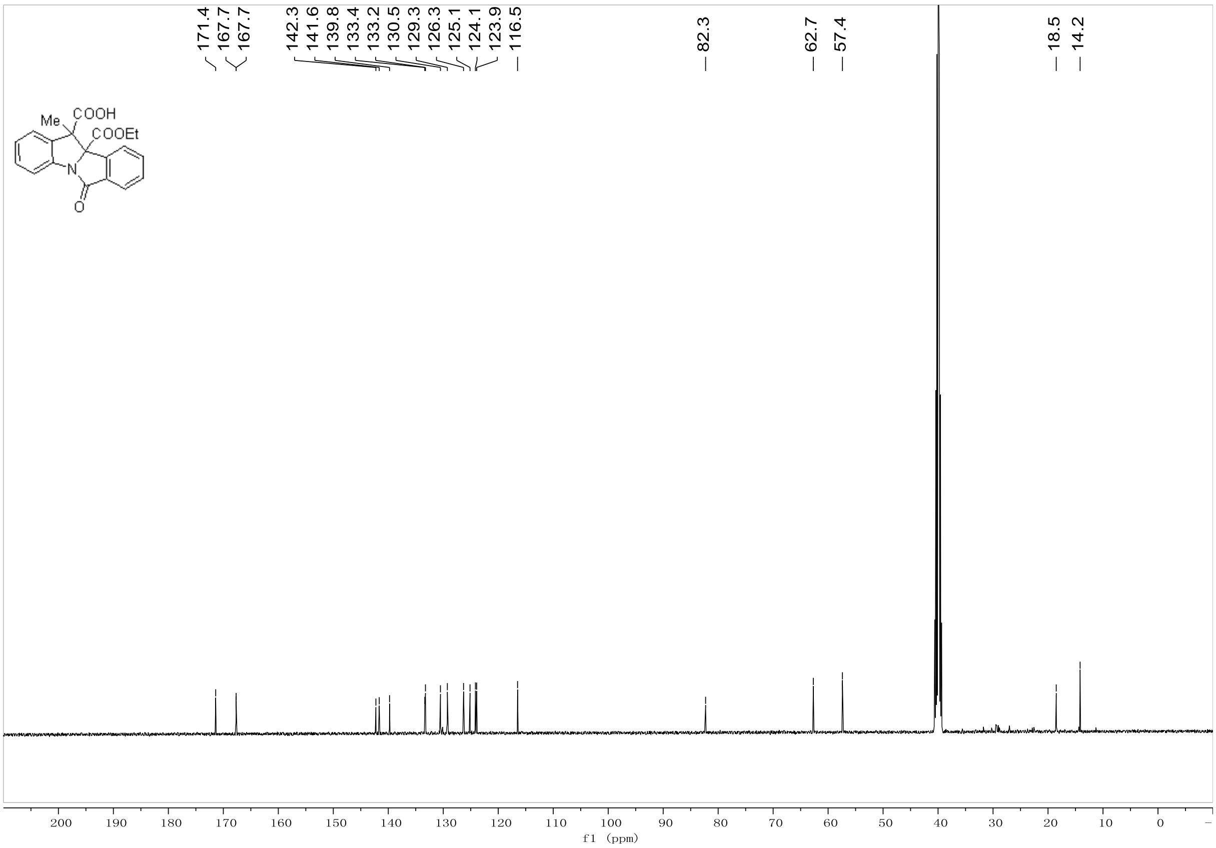

Supplementary Figure 32. ^{13}C NMR Spectra of compound **12**.

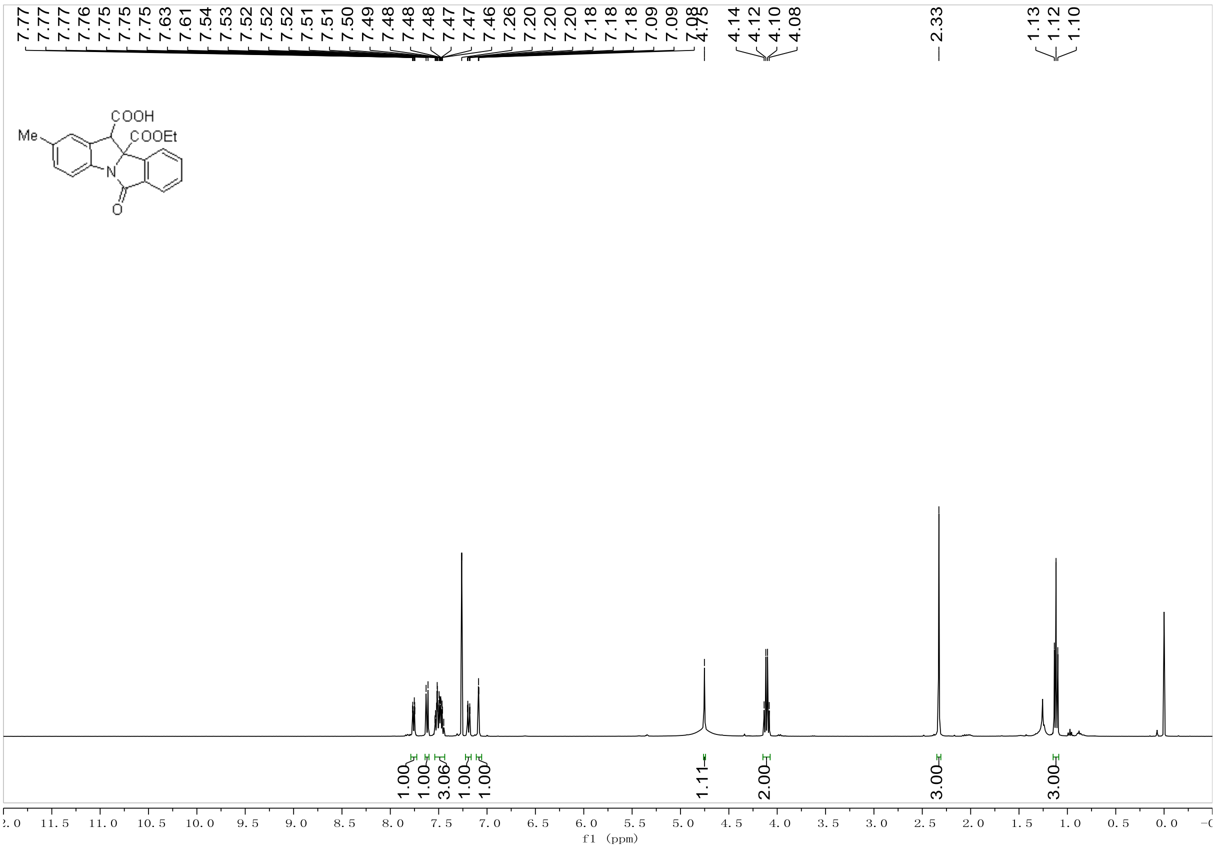

Supplementary Figure 33. ^{1}H NMR Spectra of compound **13**.

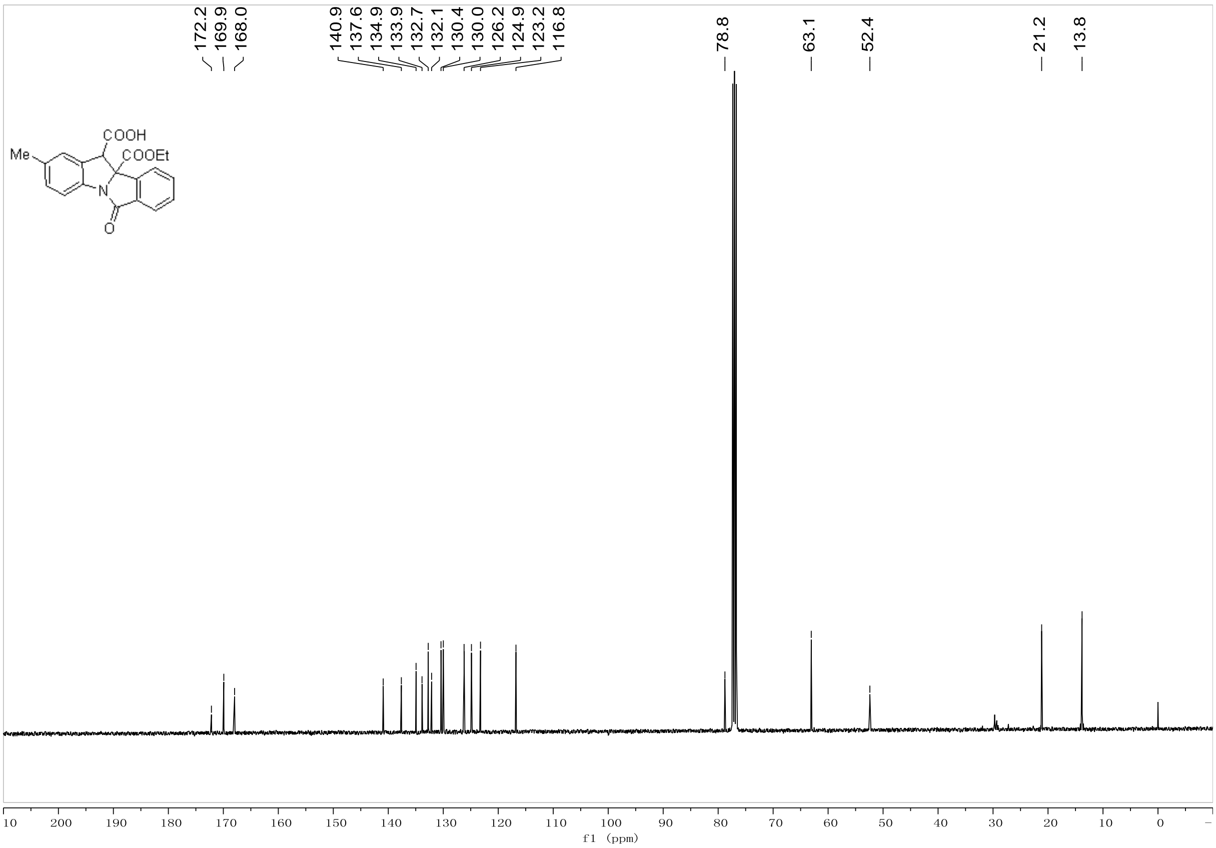

Supplementary Figure 34. ^{13}C NMR Spectra of compound **13**.

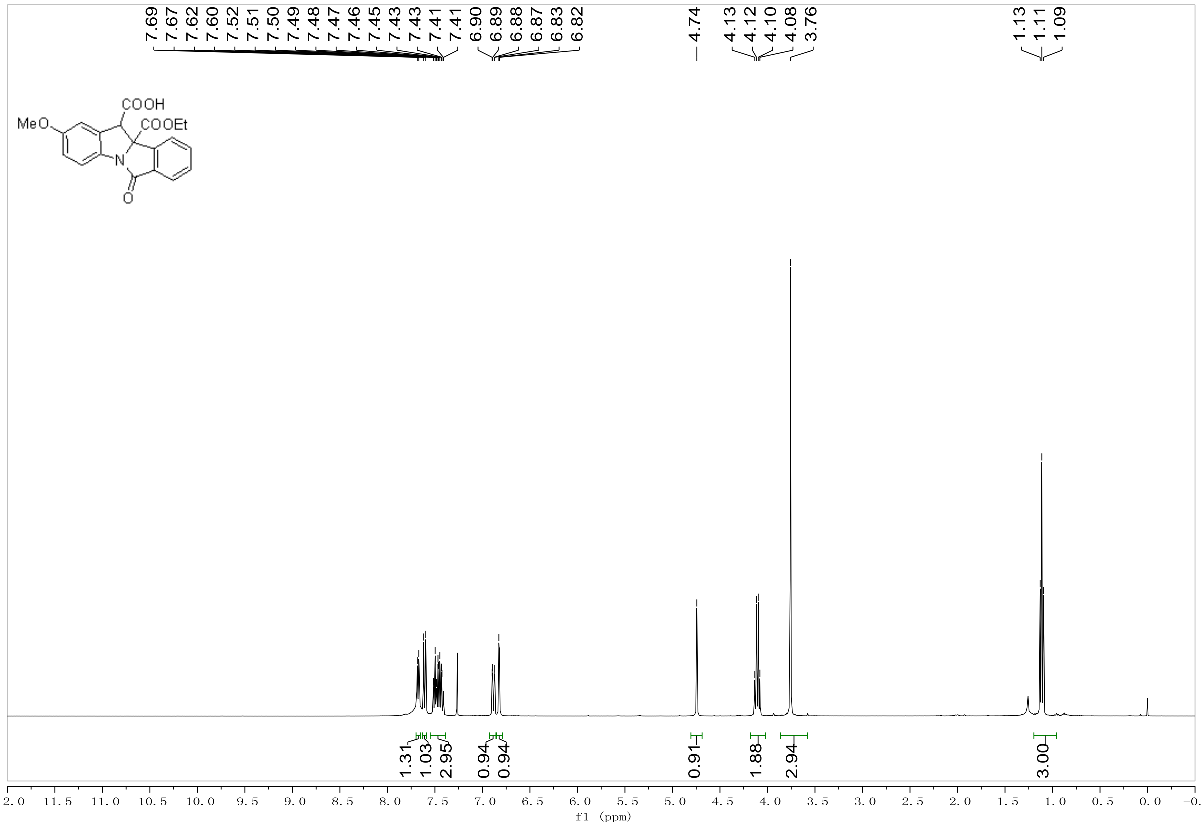

Supplementary Figure 35. ^{1}H NMR Spectra of compound **14**.

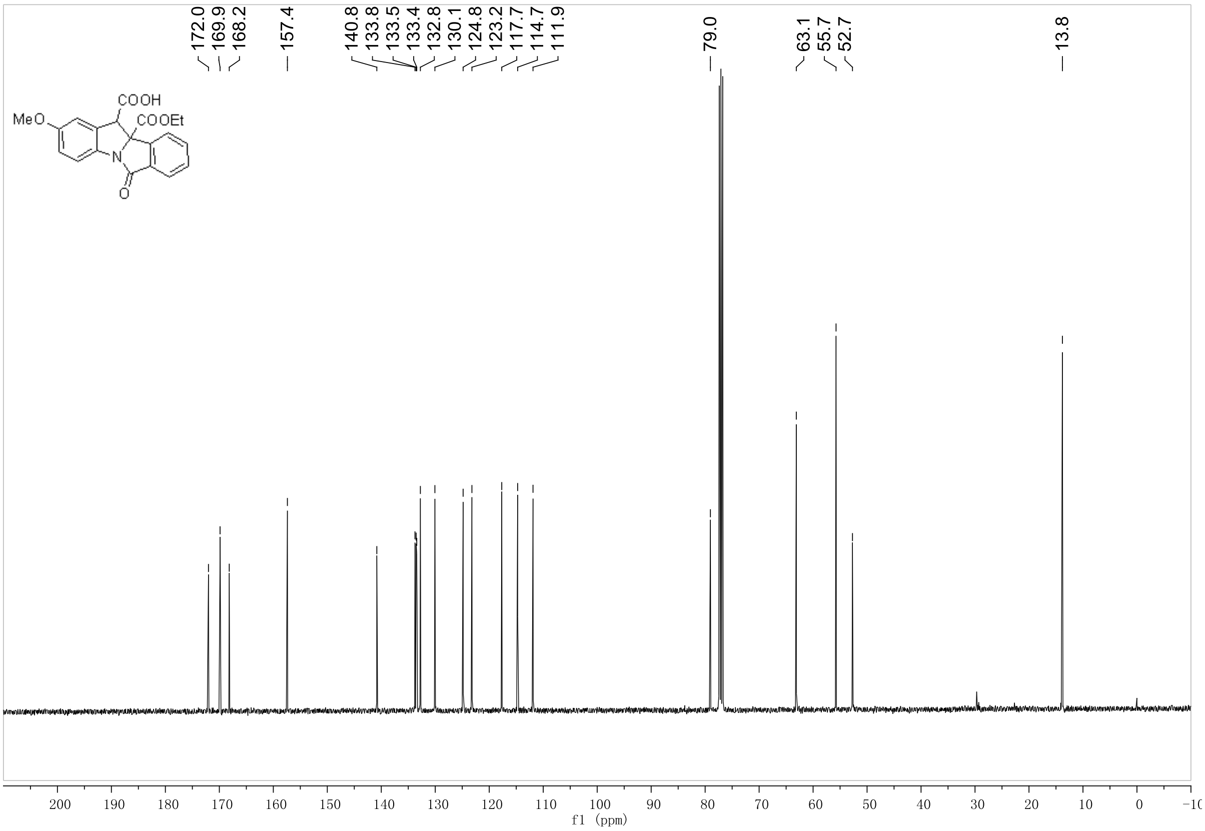

Supplementary Figure 36. ^{13}C NMR Spectra of compound **14**.

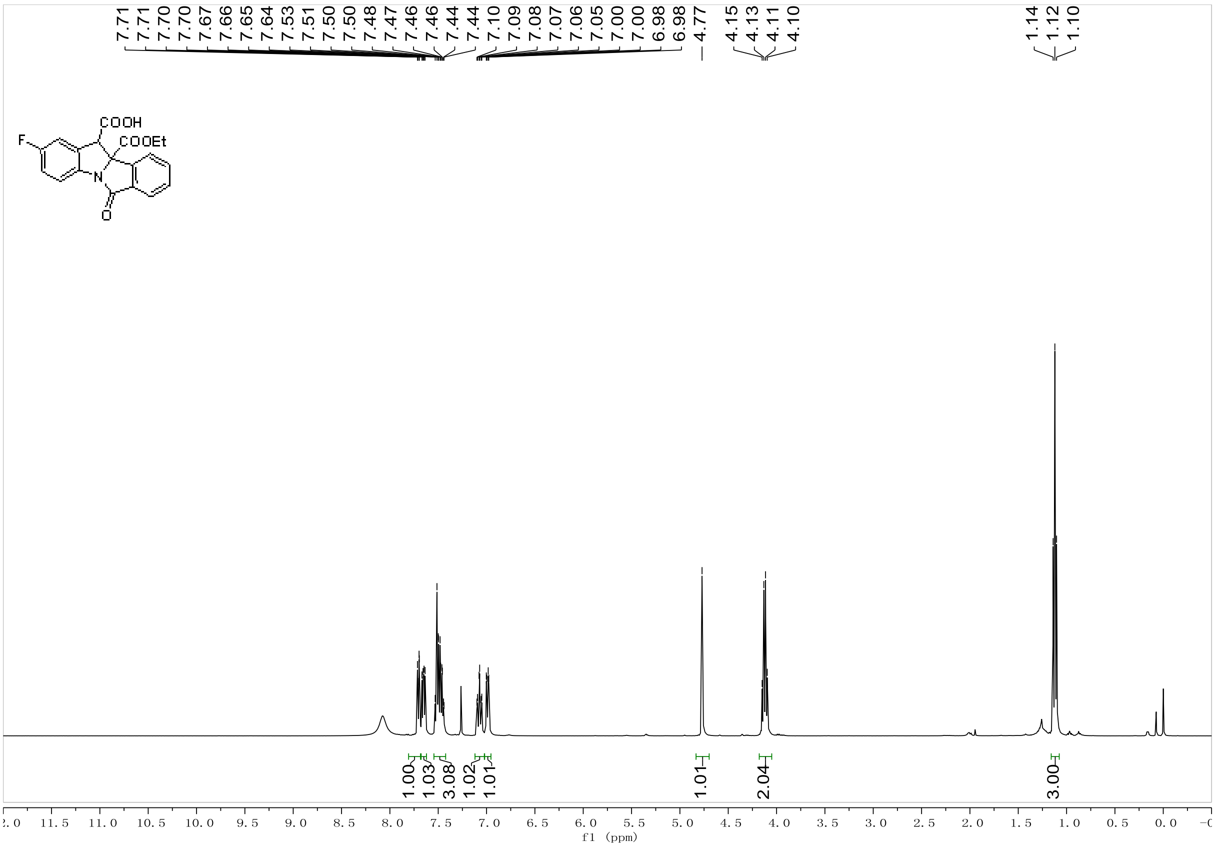

Supplementary Figure 37. ^{1}H NMR Spectra of compound **15**.

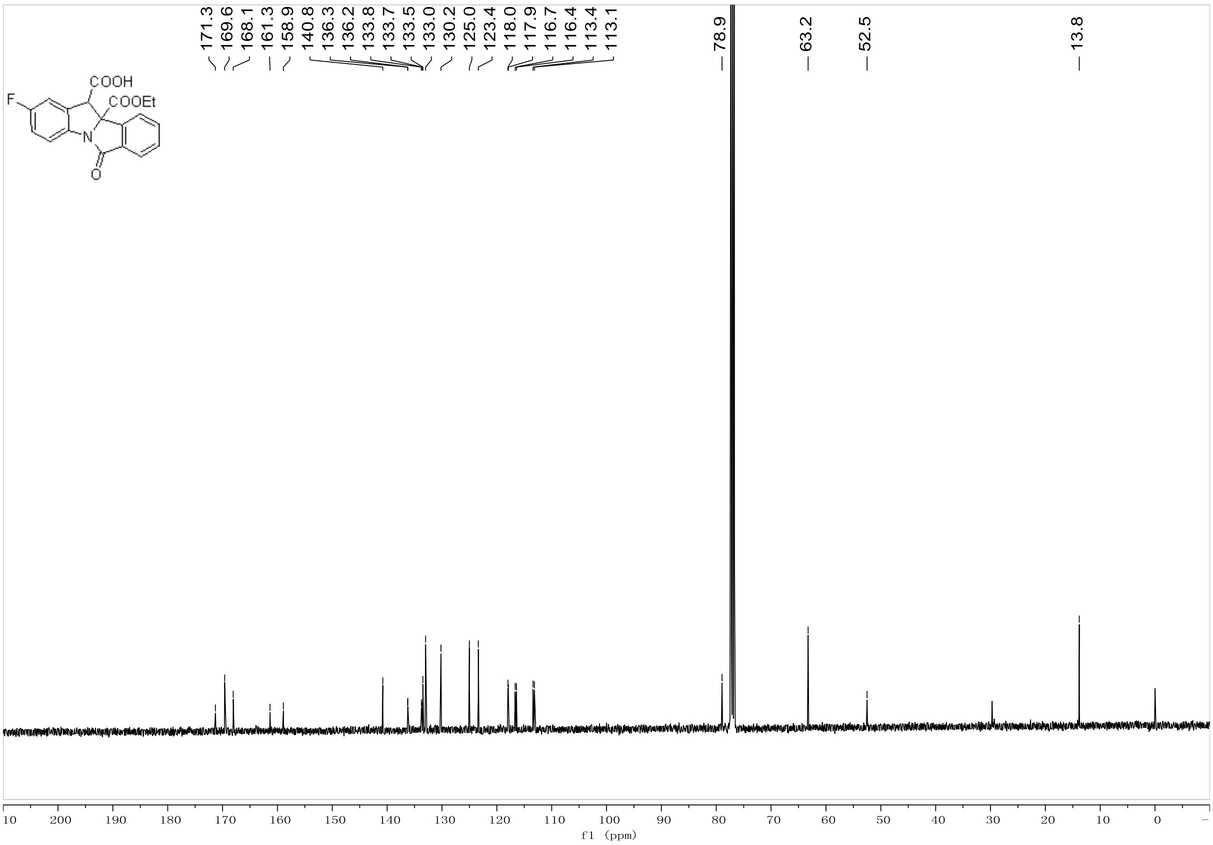

Supplementary Figure 38. ^{13}C NMR Spectra of compound **15**.

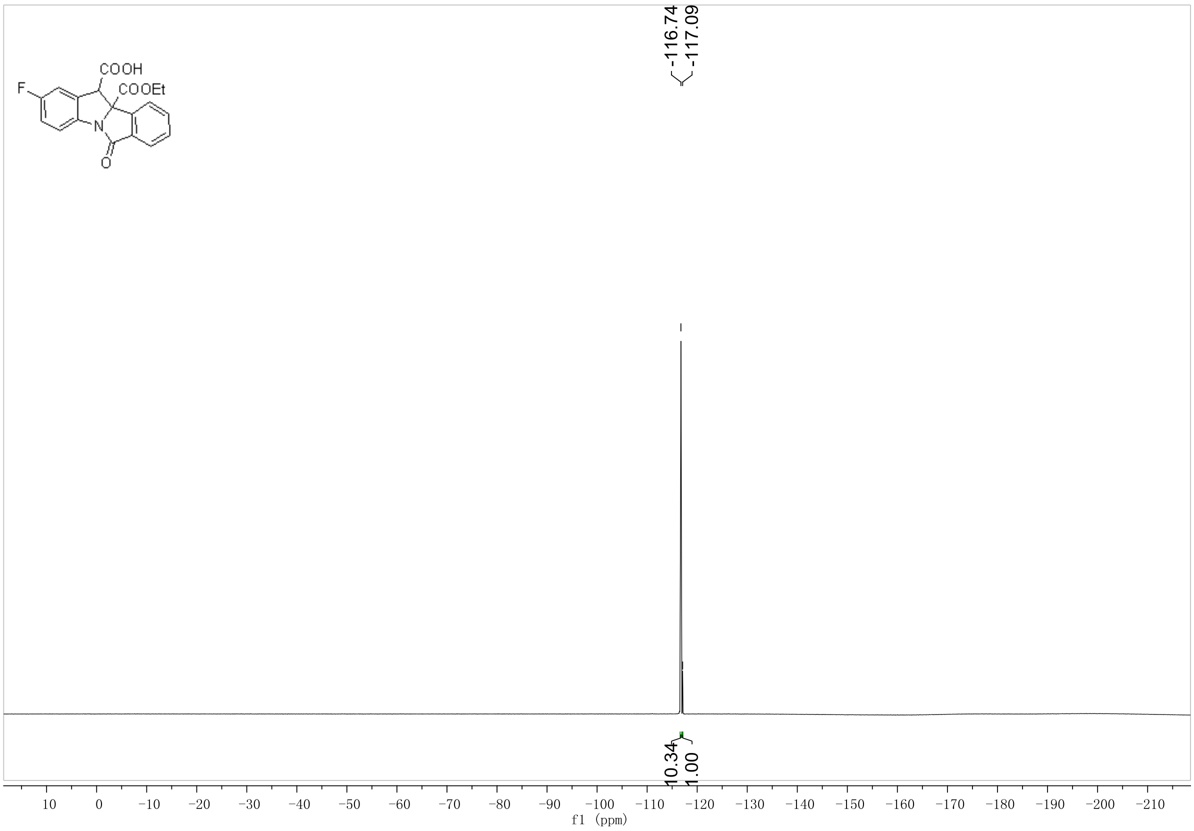

Supplementary Figure 39. ^{19}F NMR Spectra of compound **15**.

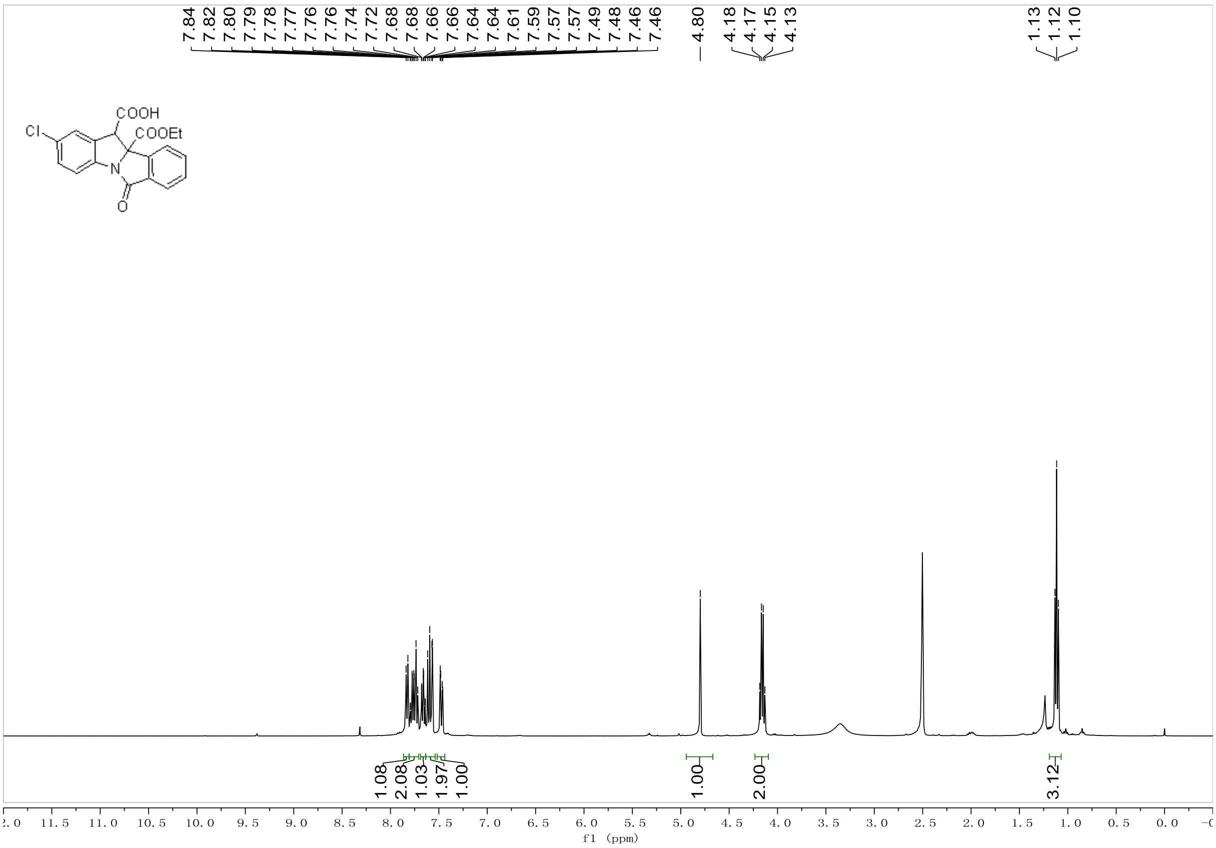

Supplementary Figure 40. ^{1}H NMR Spectra of compound **16**.

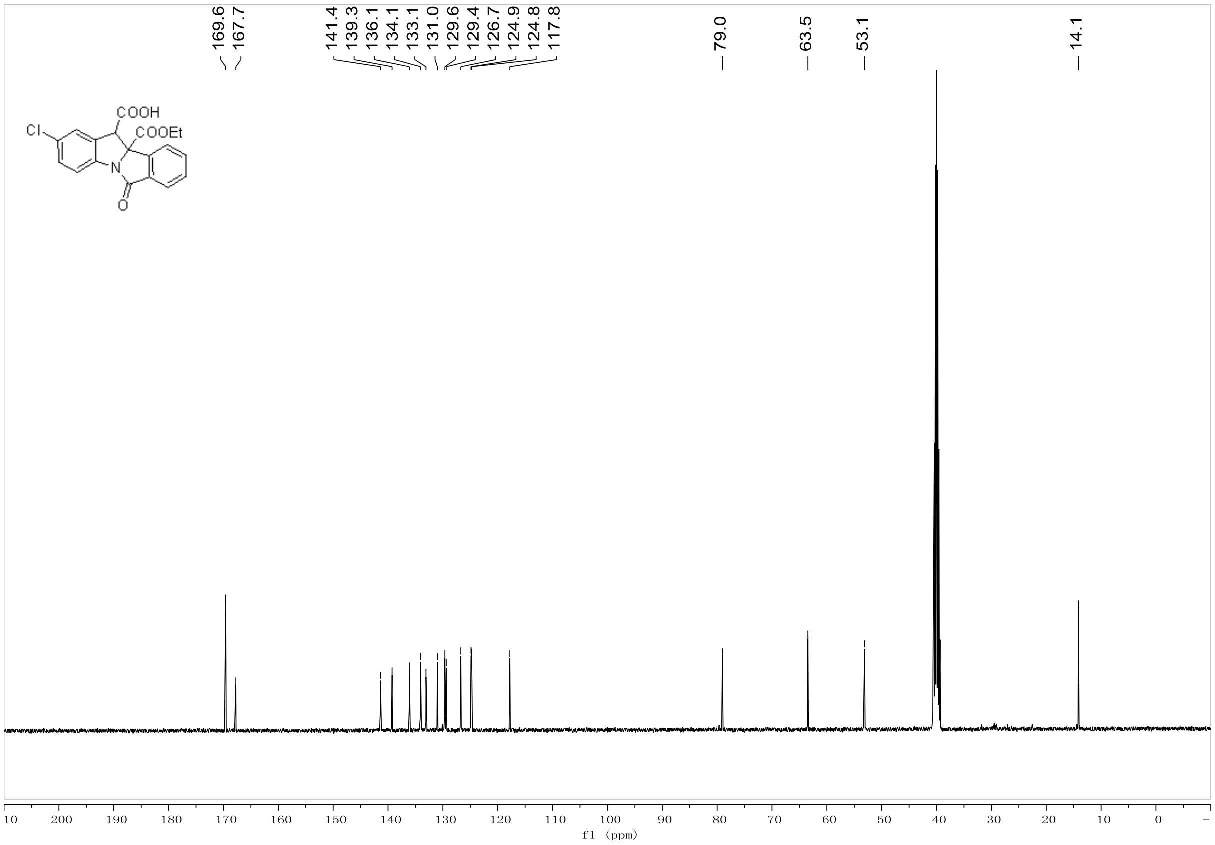

Supplementary Figure 41. ^{13}C NMR Spectra of compound **16**.

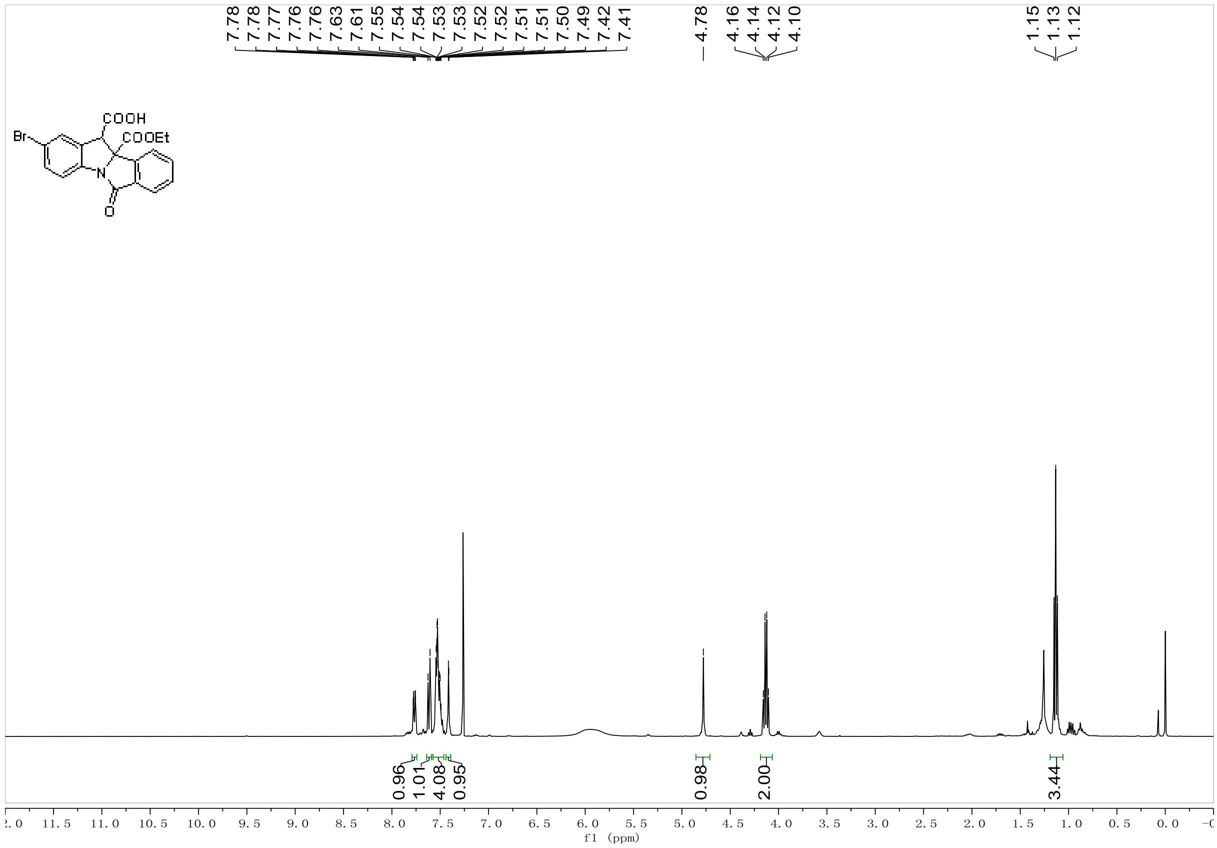

Supplementary Figure 42. ^{1}H NMR Spectra of compound **17**.

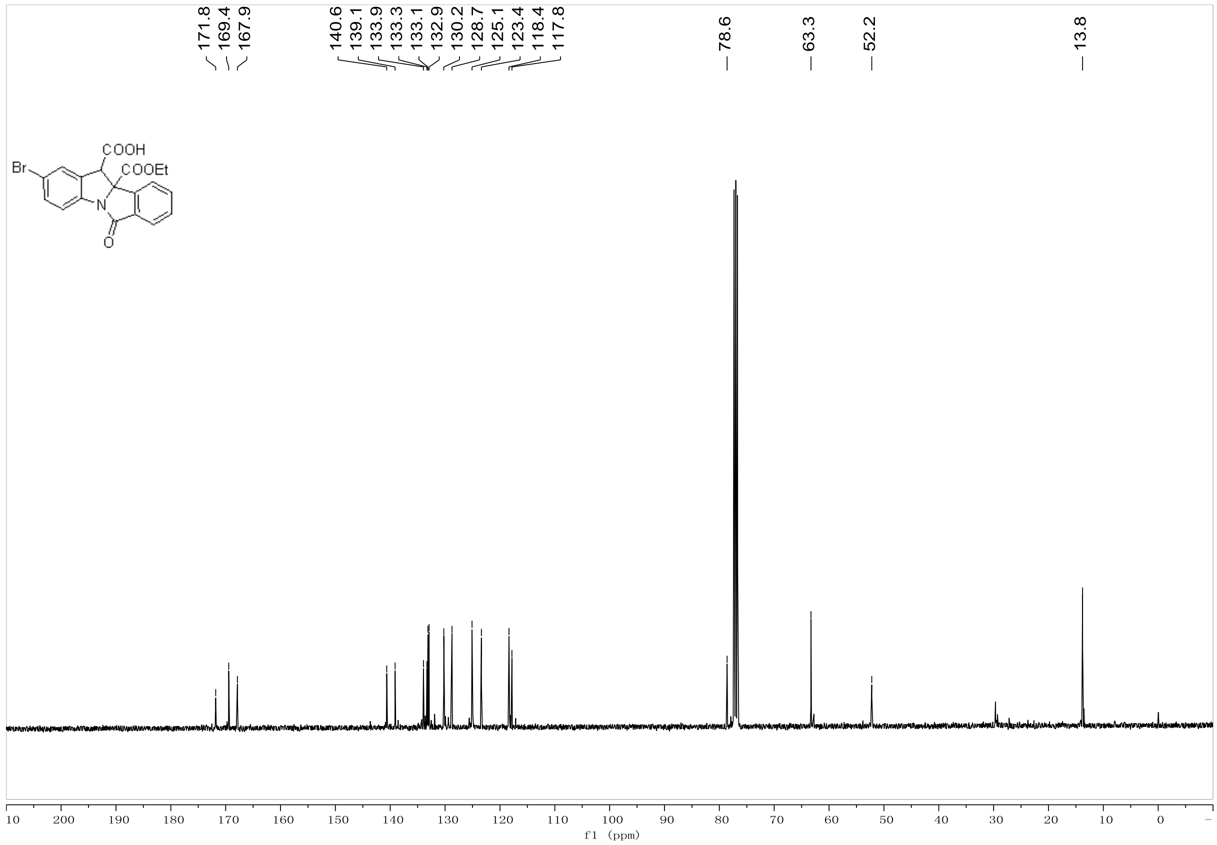

Supplementary Figure 43. ^{13}C NMR Spectra of compound **17**.

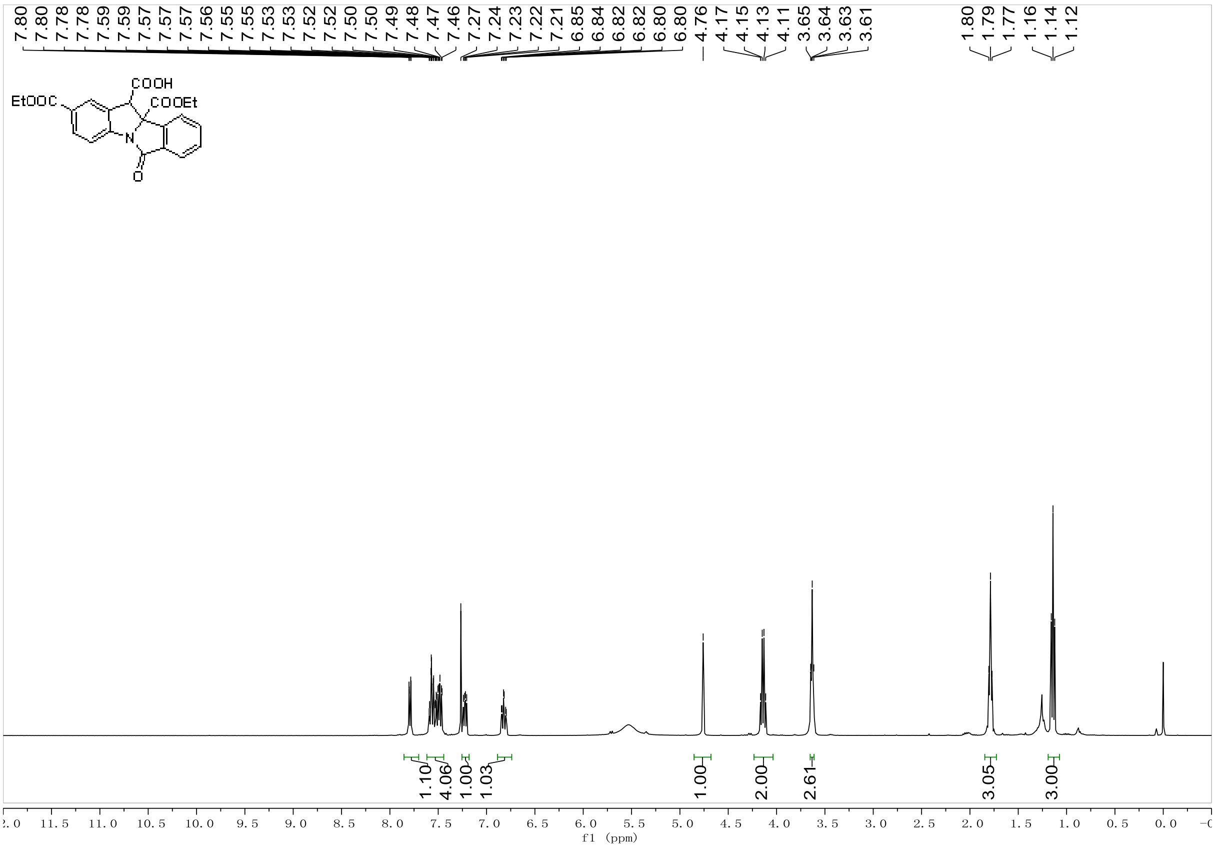

Supplementary Figure 44. ^{1}H NMR Spectra of compound **18**.

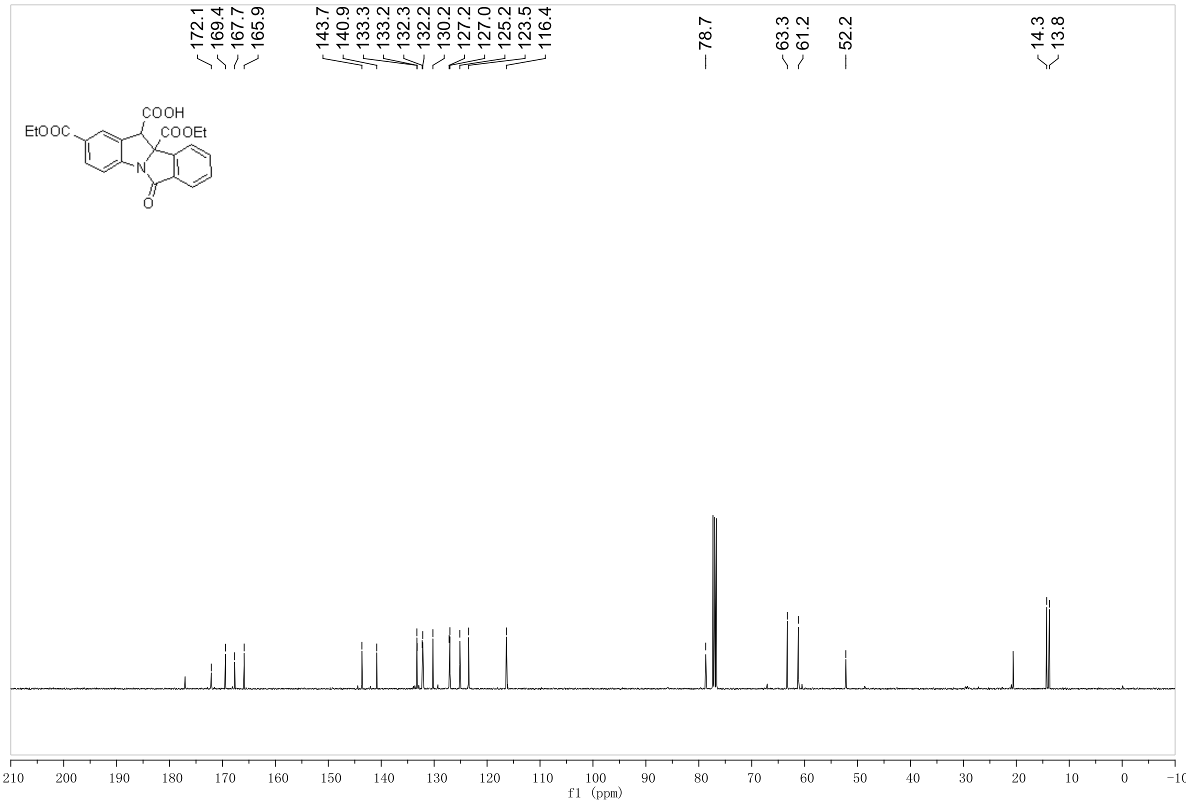

Supplementary Figure 45. ^{13}C NMR Spectra of compound **18**.

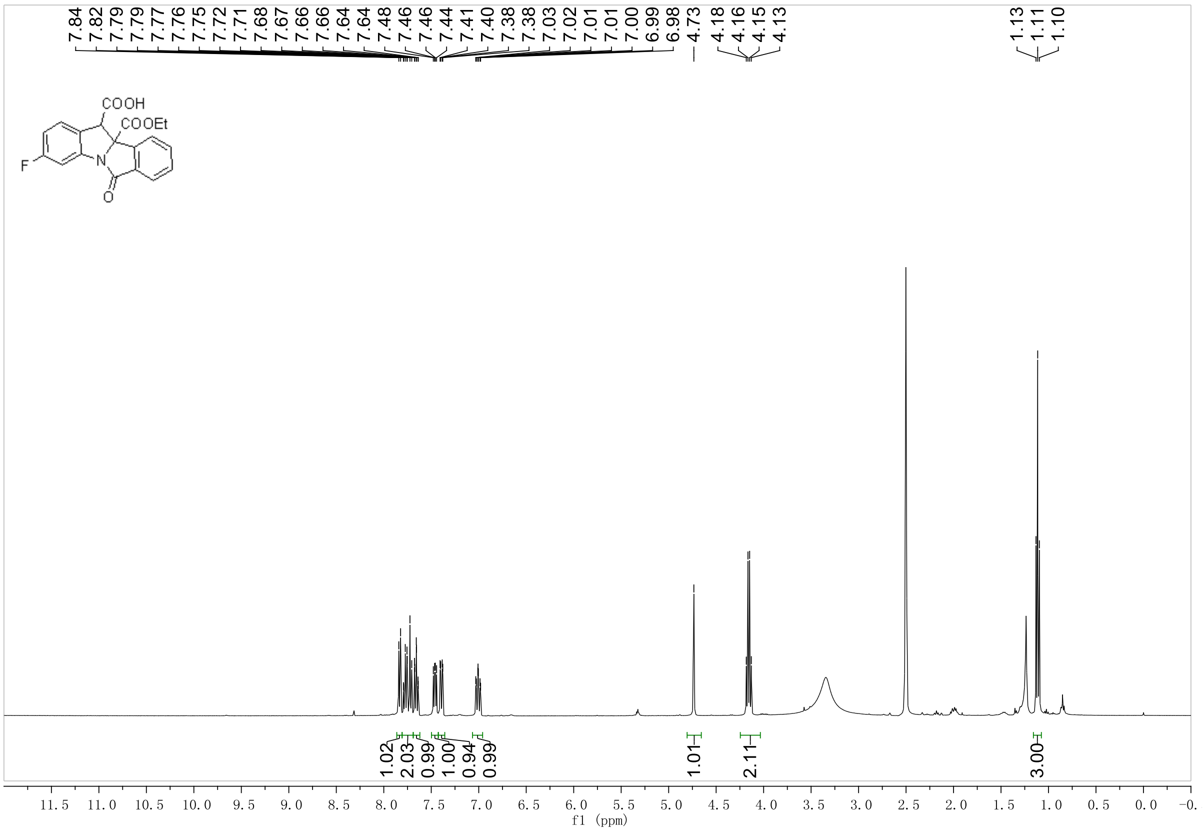

Supplementary Figure 46. ^{1}H NMR Spectra of compound **19**.

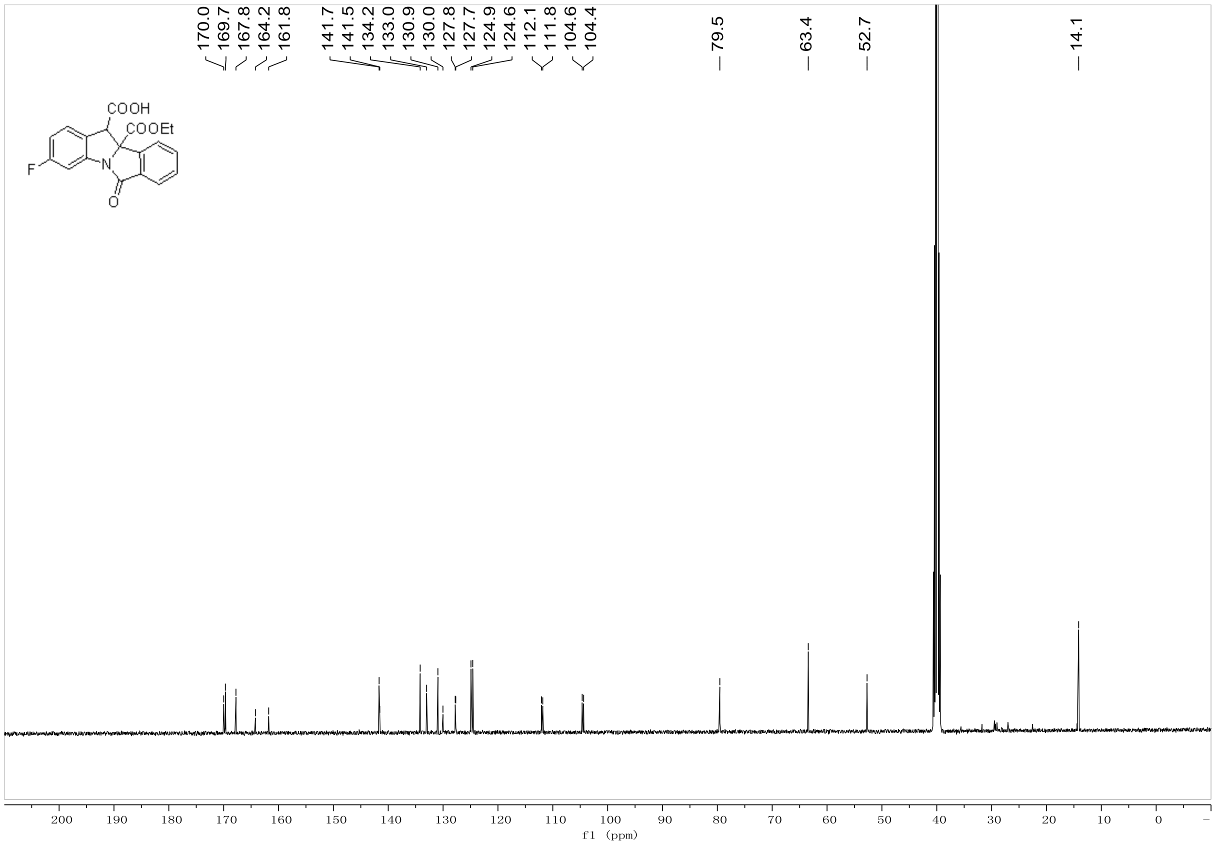

Supplementary Figure 47. ^{13}C NMR Spectra of compound **19**.

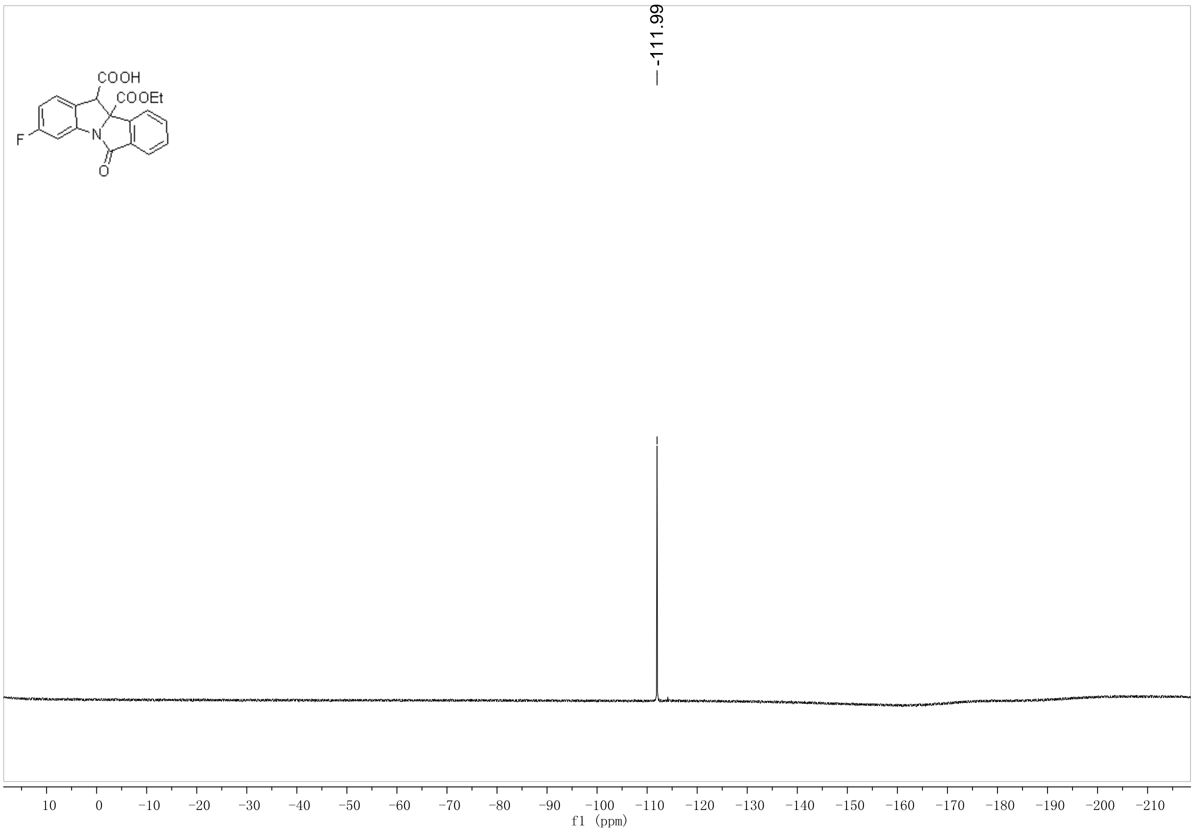

Supplementary Figure 48. ^{19}F NMR Spectra of compound **19**.

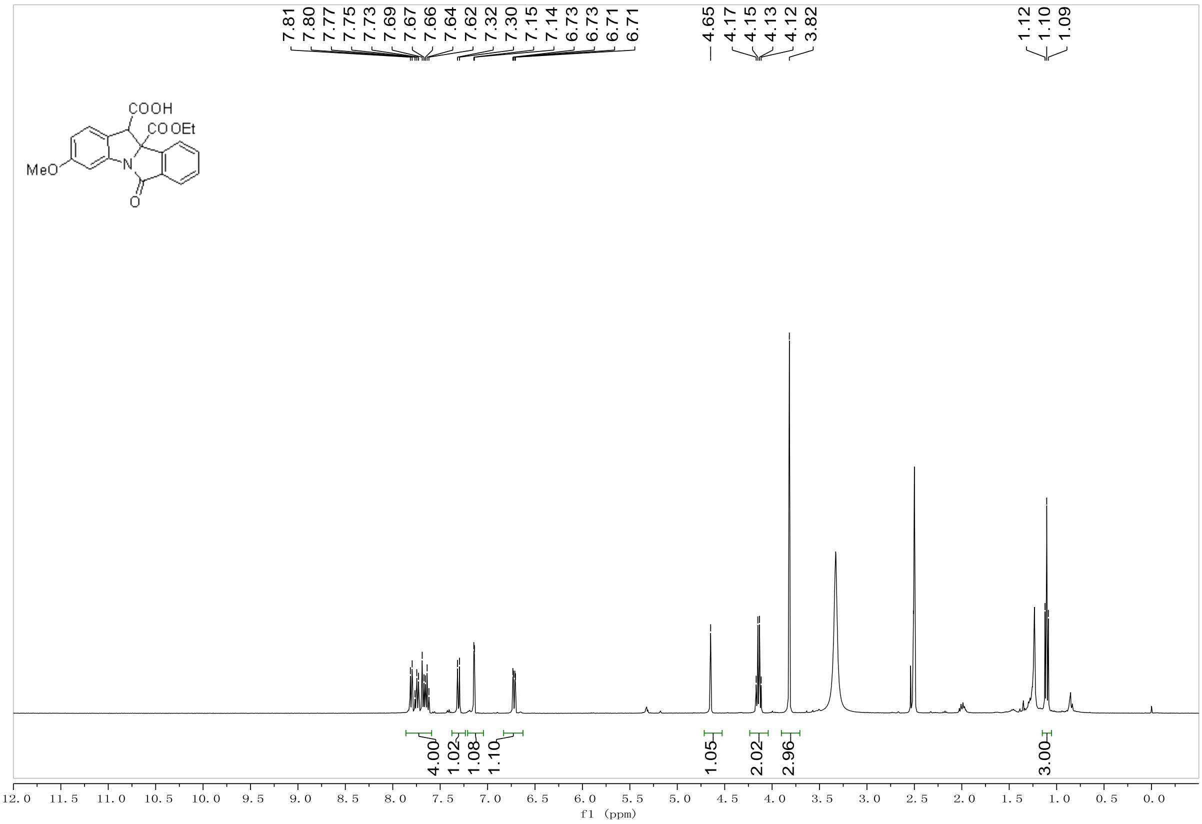

Supplementary Figure 49. ^{1}H NMR Spectra of compound **20**.

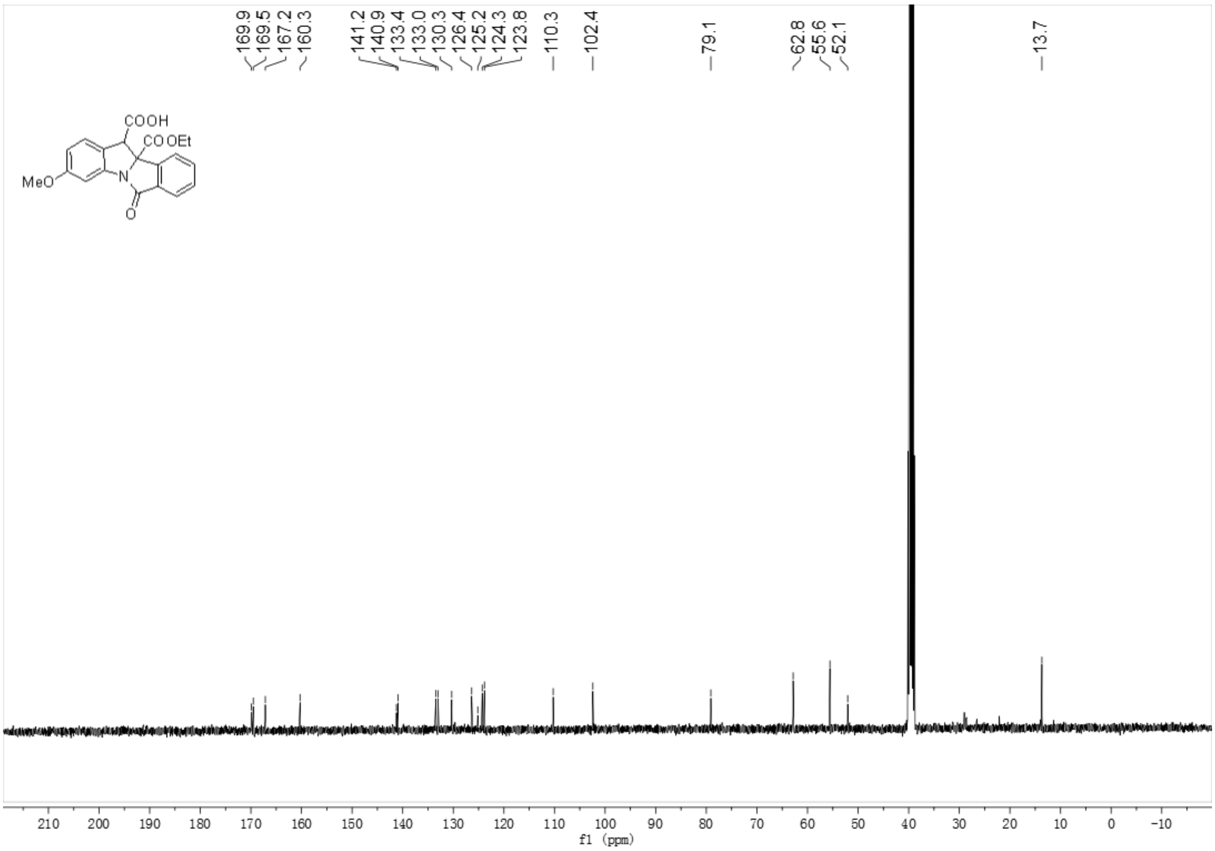

Supplementary Figure 50. ^{13}C NMR Spectra of compound **20**.

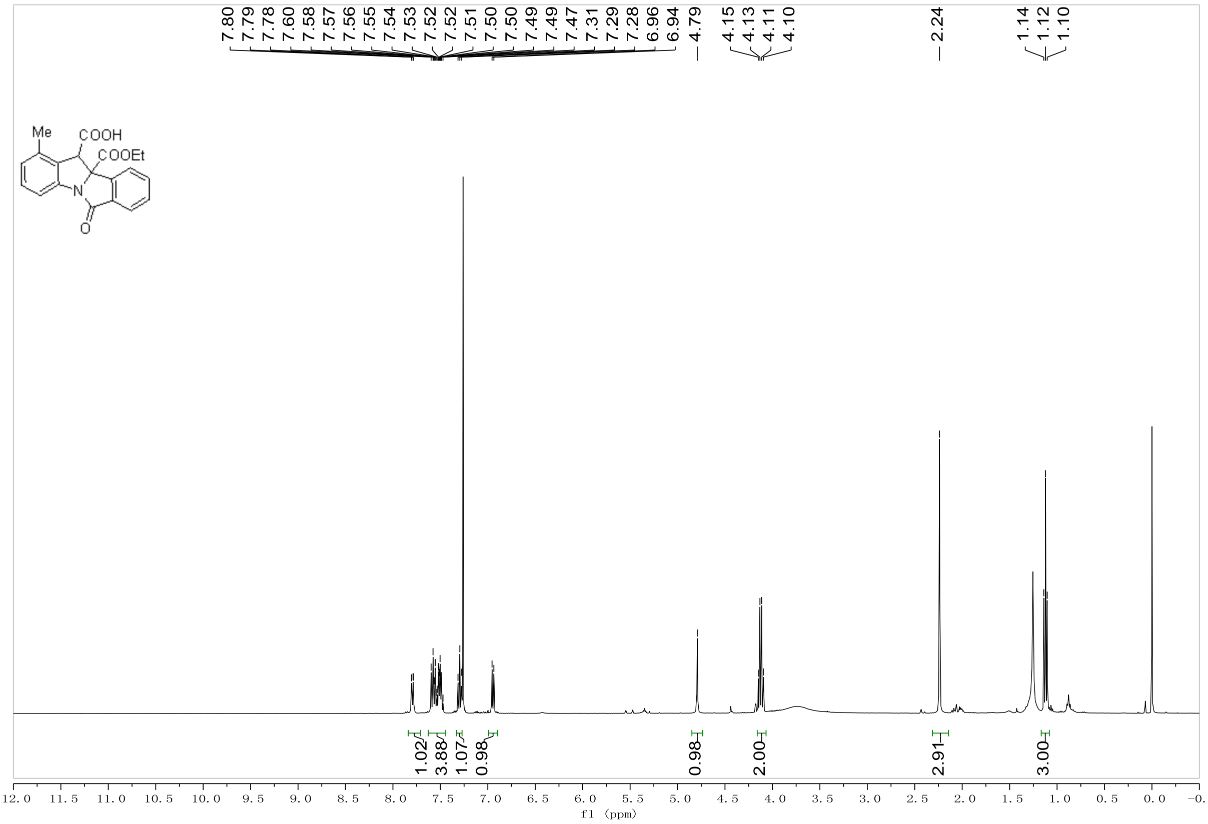

Supplementary Figure 51. ^{1}H NMR Spectra of compound **21**.

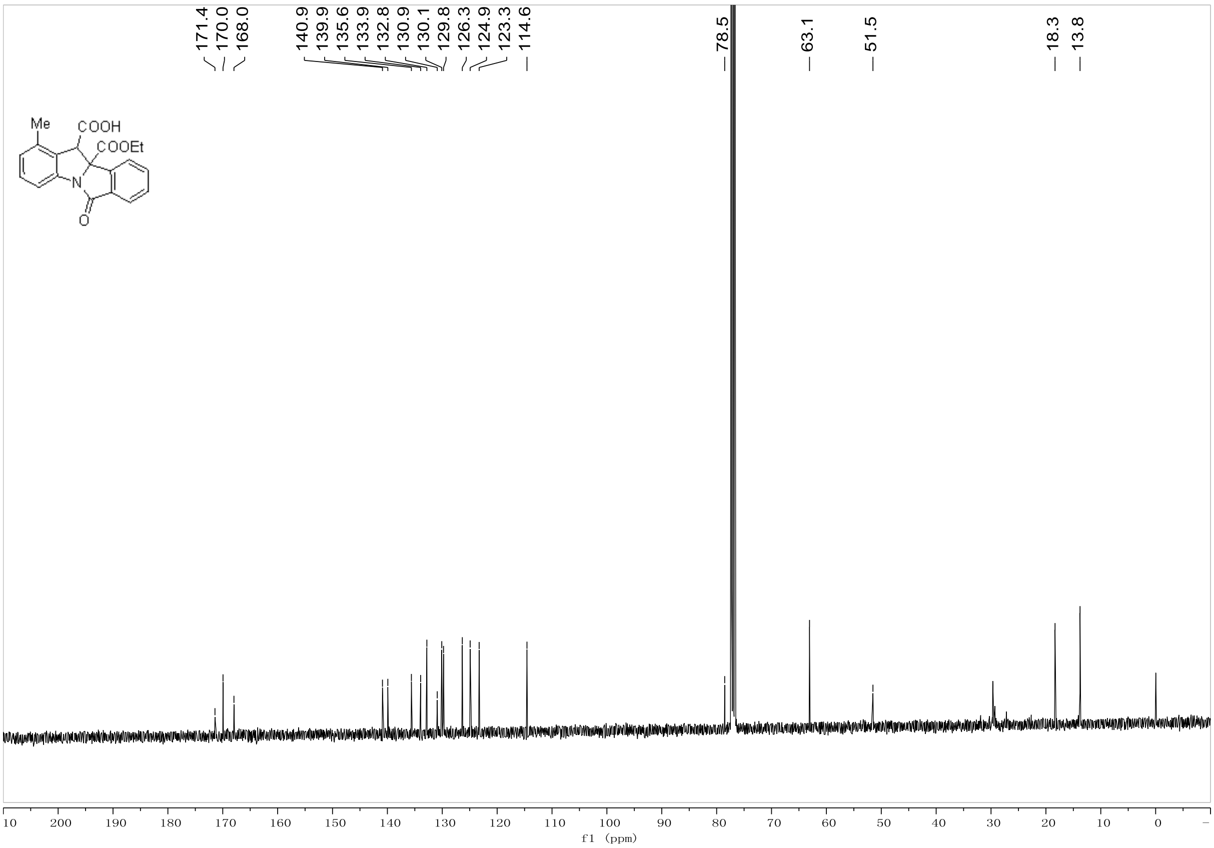

Supplementary Figure 52. ^{13}C NMR Spectra of compound **21**.

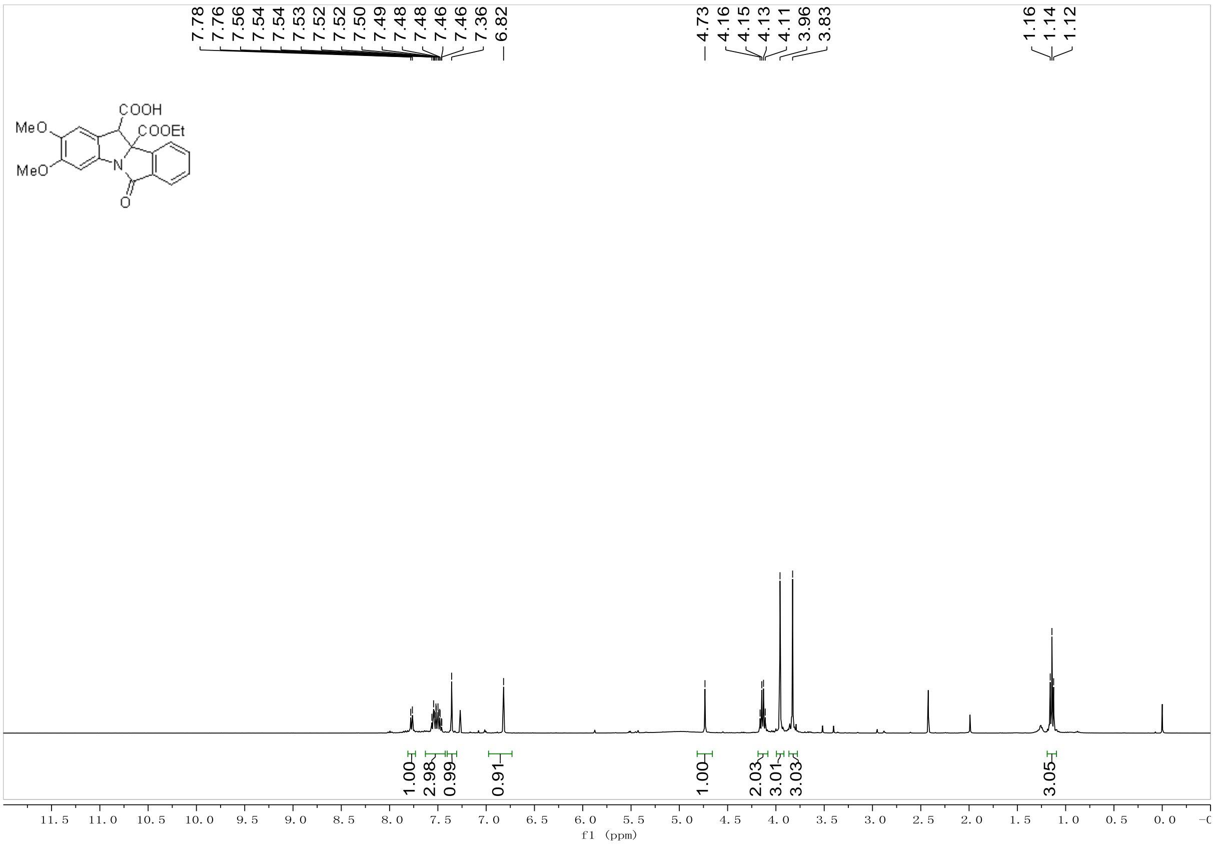

Supplementary Figure 53. ^{1}H NMR Spectra of compound **22**.

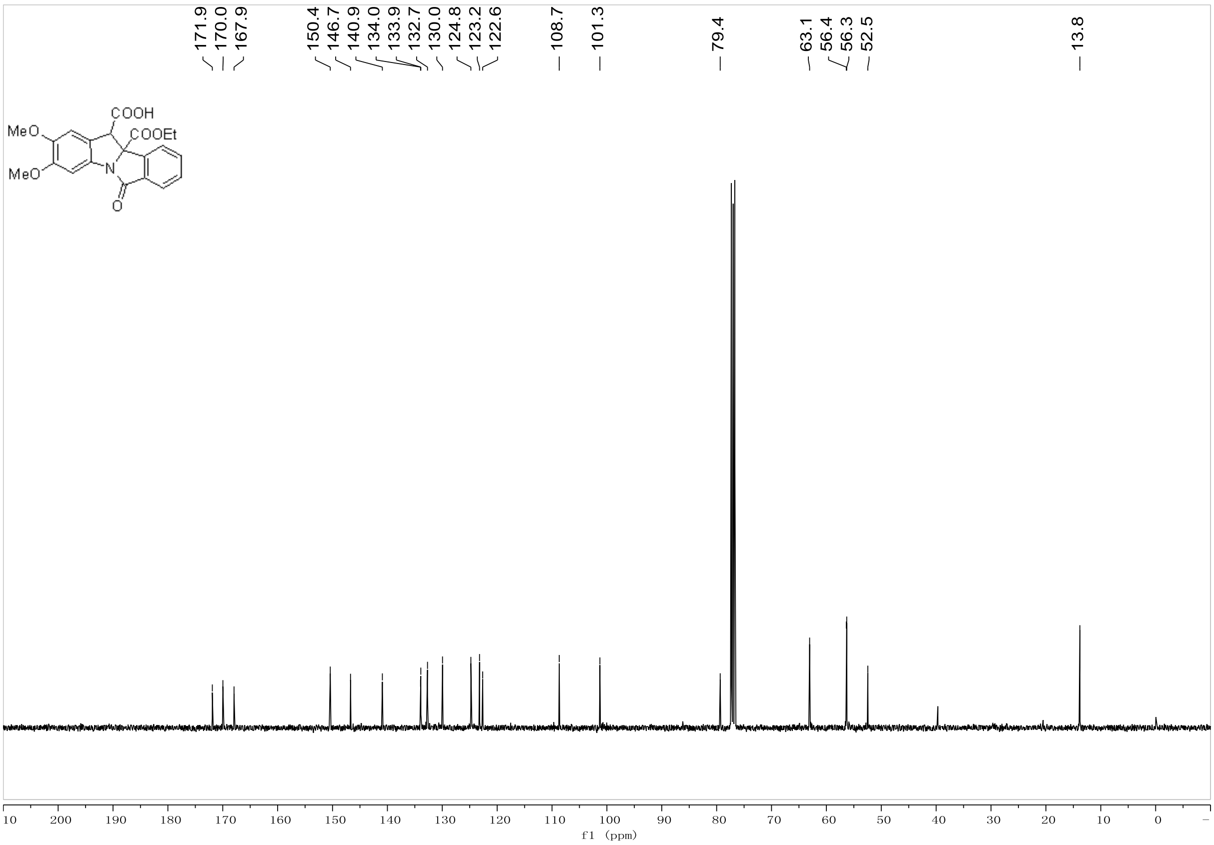

Supplementary Figure 54. ^{13}C NMR Spectra of compound **22**.

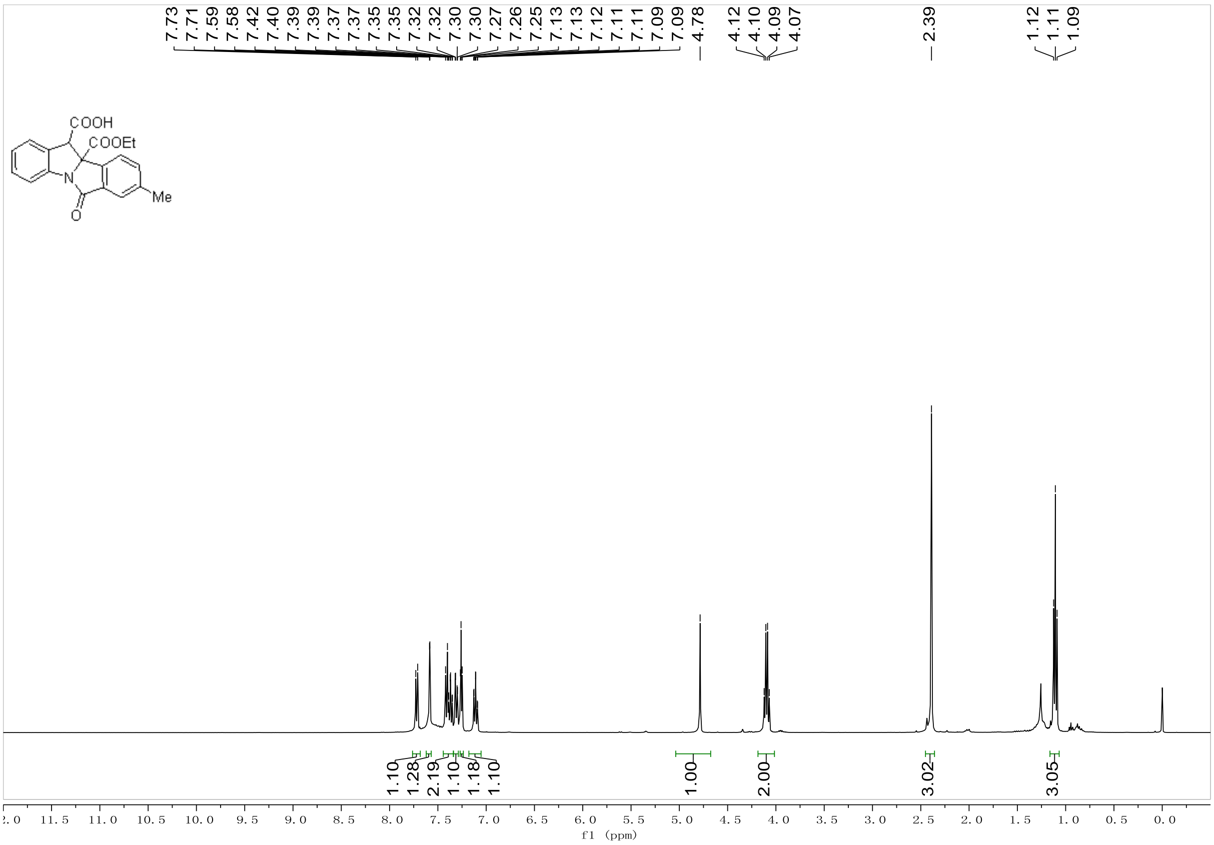

Supplementary Figure 55. ^{1}H NMR Spectra of compound **23**.

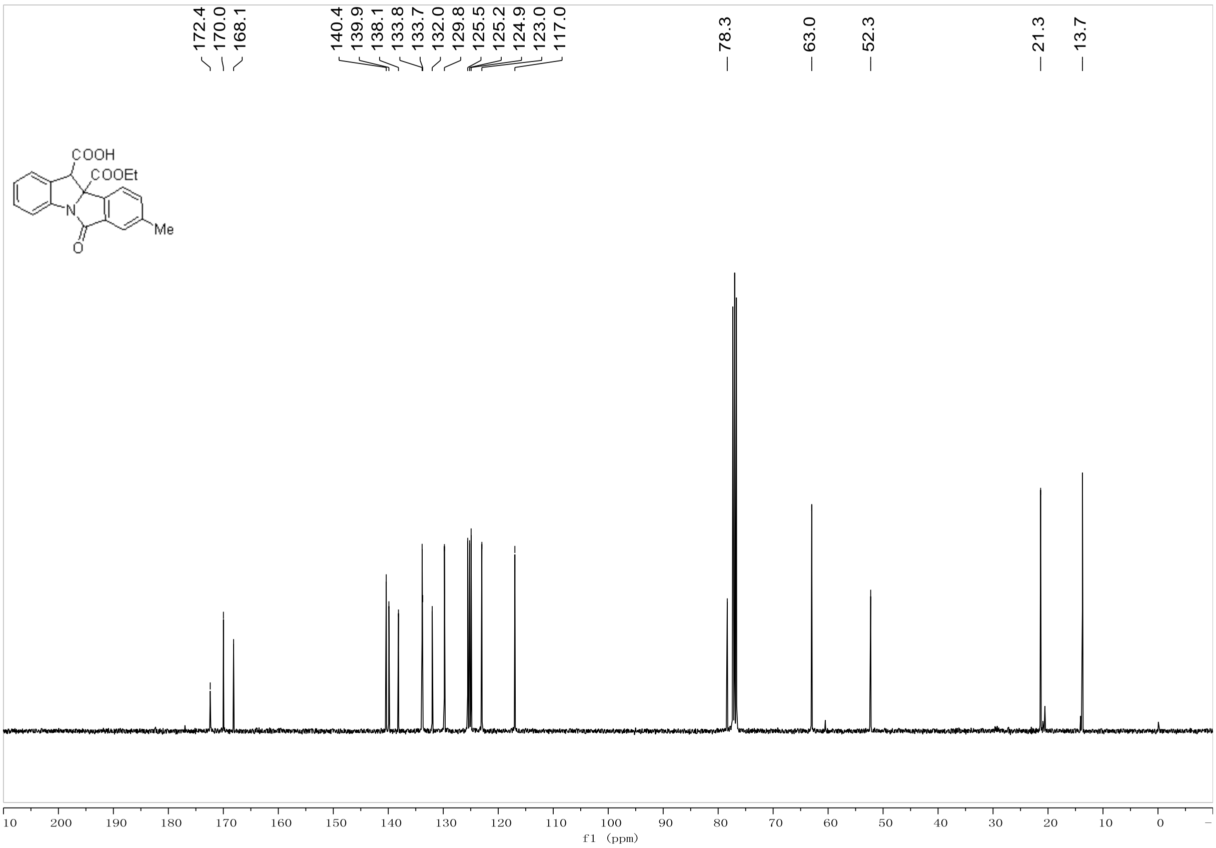

Supplementary Figure 56. ^{13}C NMR Spectra of compound **23**.

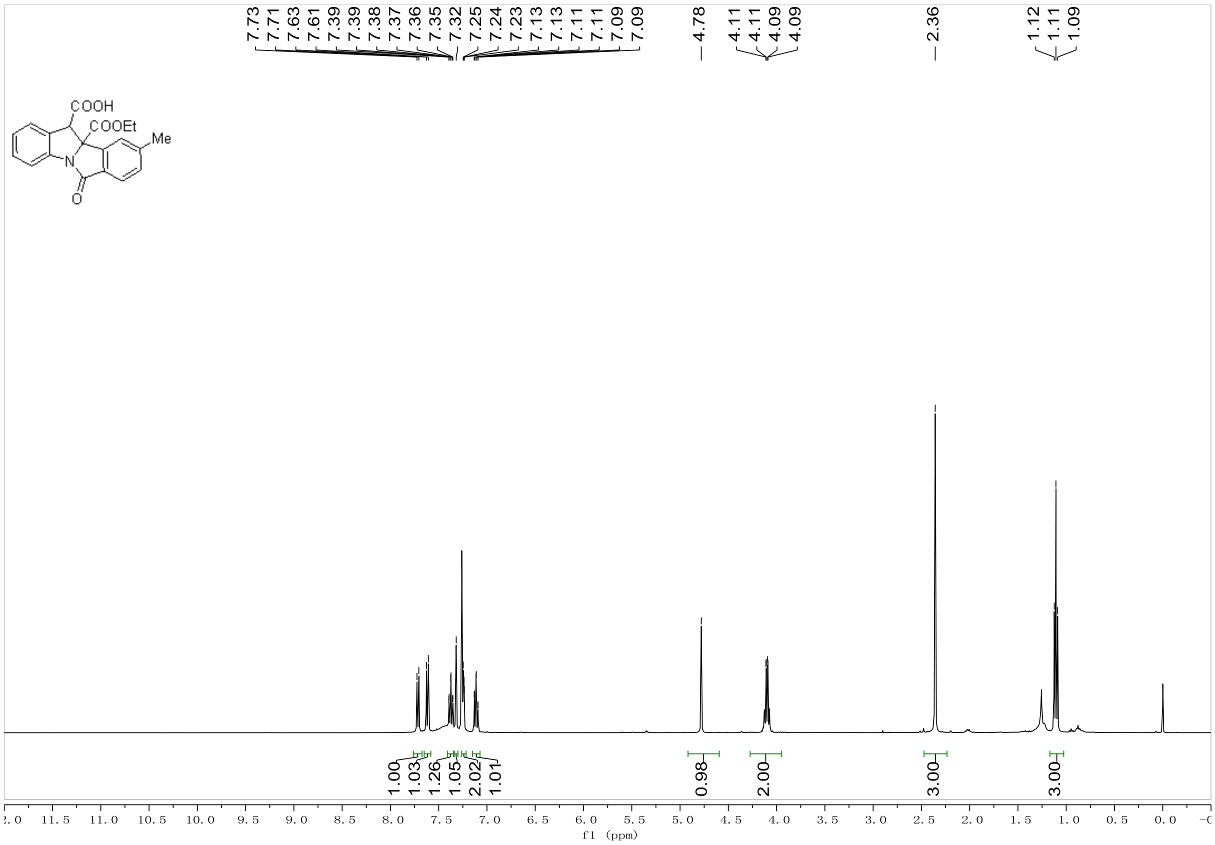

Supplementary Figure 57. ^{1}H NMR Spectra of compound **24**.

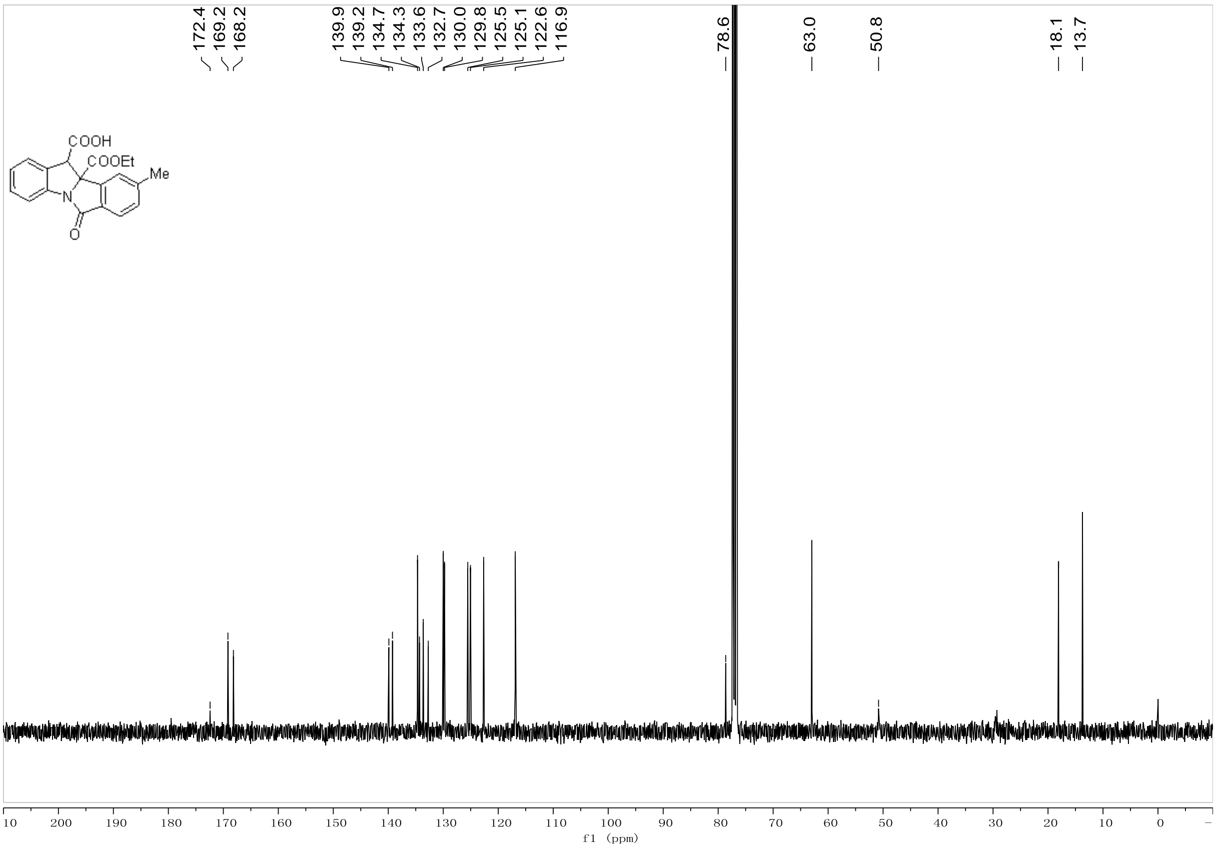

Supplementary Figure 58. ^{13}C NMR Spectra of compound **24**.

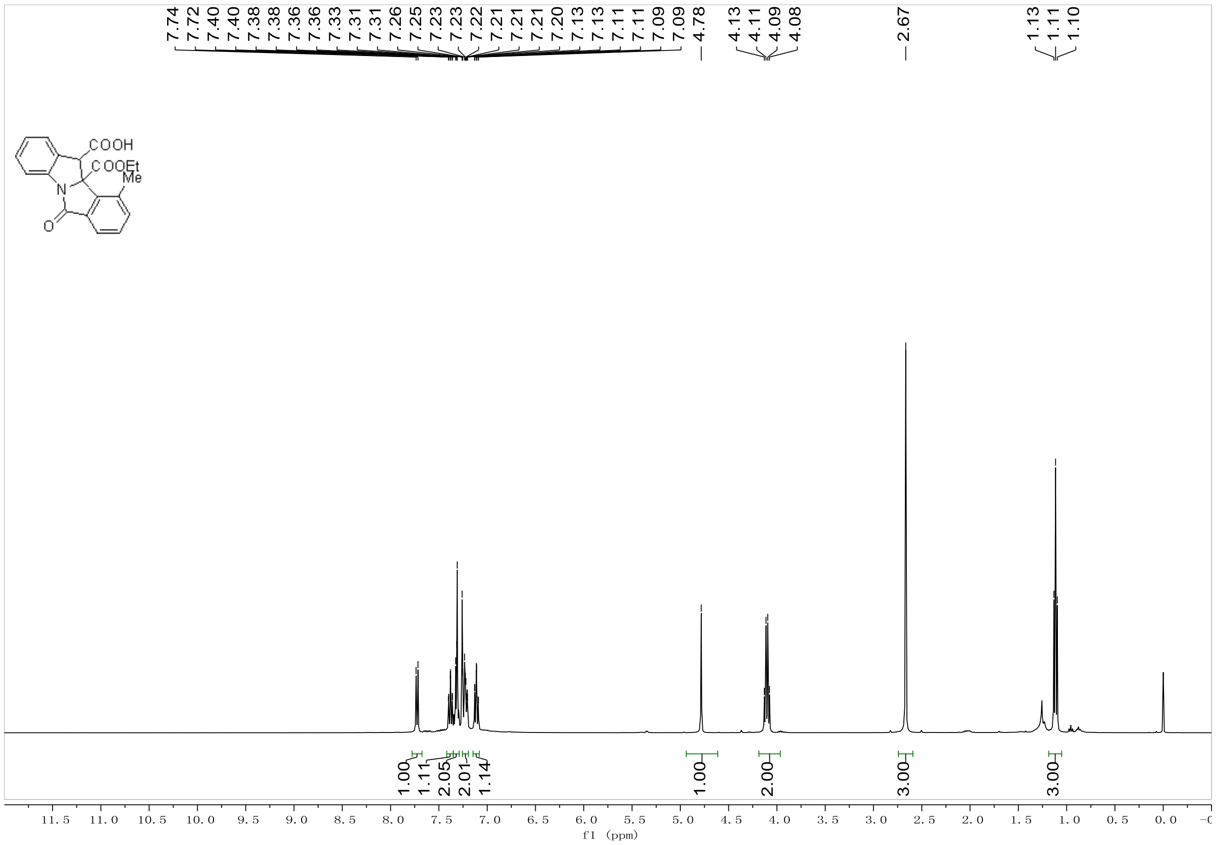

Supplementary Figure 59. ^{1}H NMR Spectra of compound **25**.

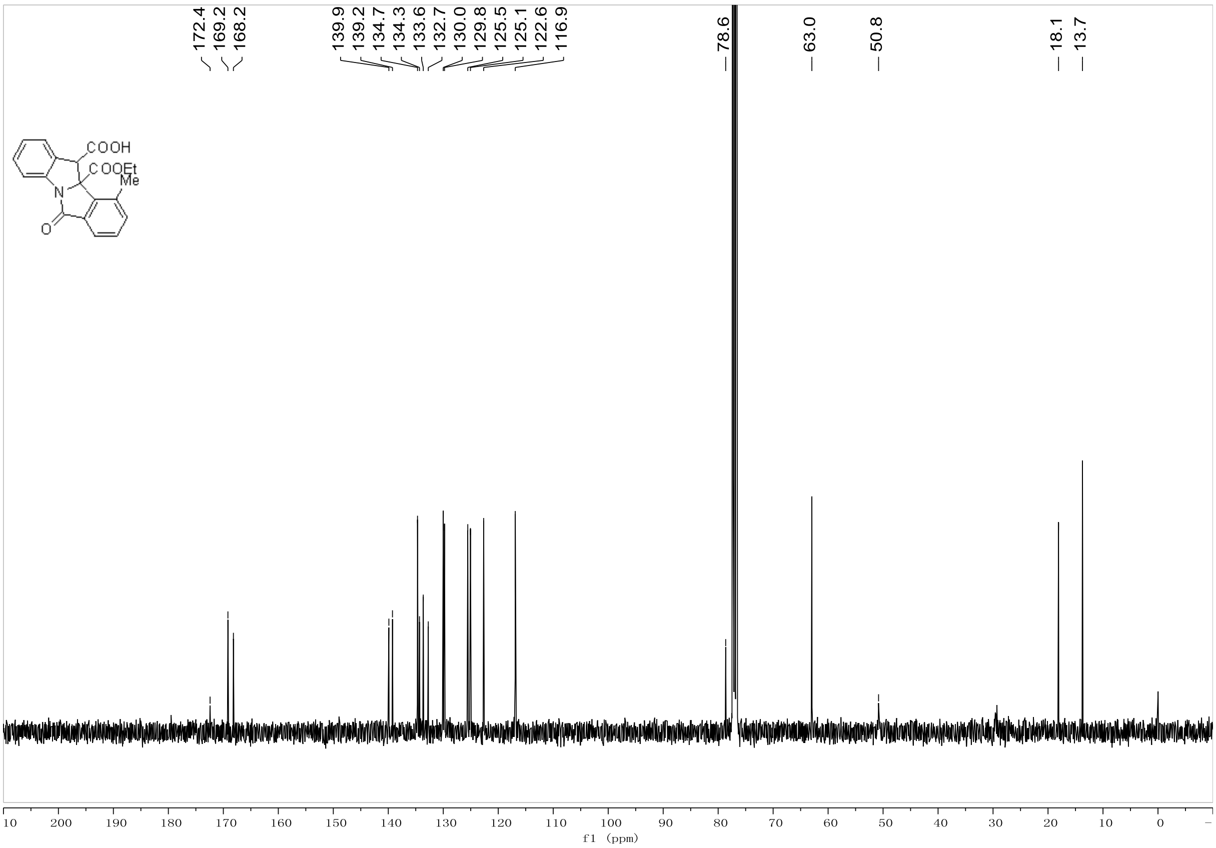

Supplementary Figure 60. ^{13}C NMR Spectra of compound **25**.

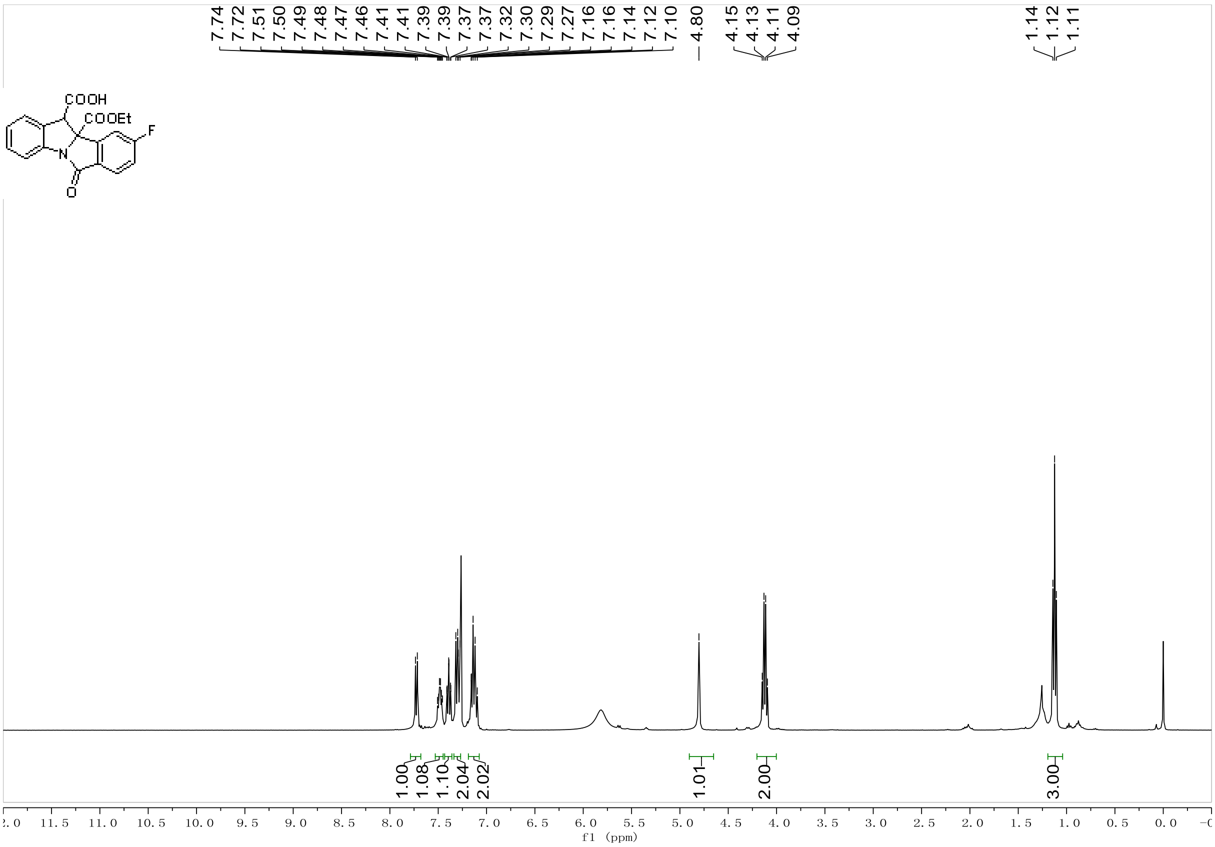

Supplementary Figure 61. ^{1}H NMR Spectra of compound **26**.

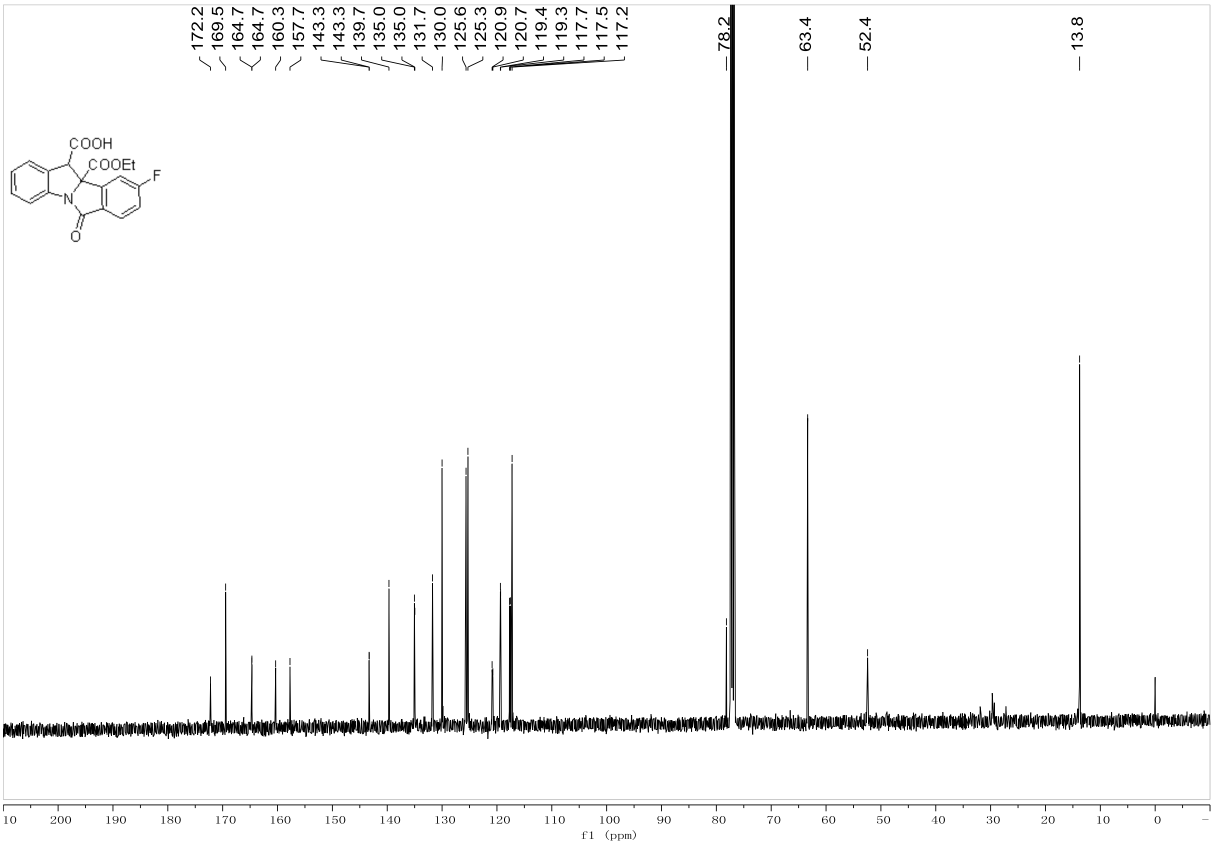

Supplementary Figure 62. ^{13}C NMR Spectra of compound **26**.

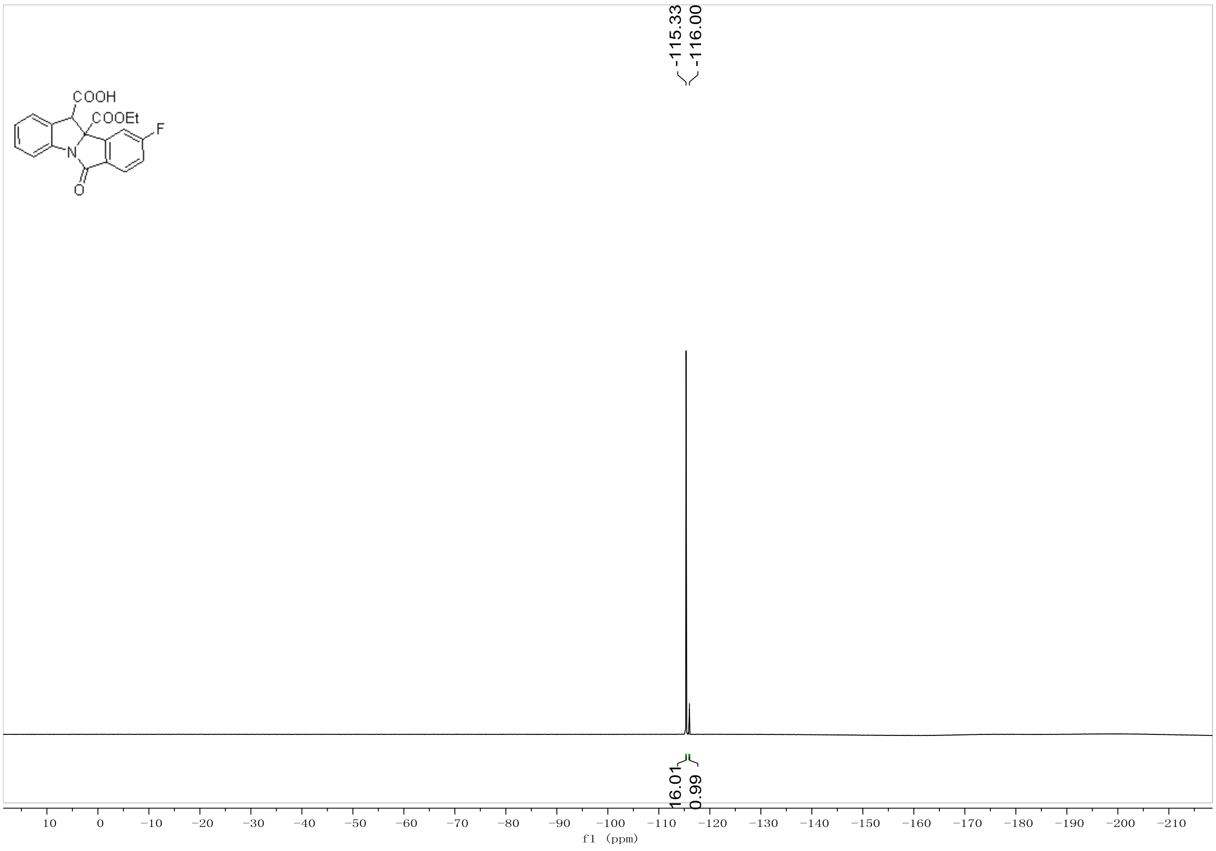

Supplementary Figure 63. ^{19}F NMR Spectra of compound **26**.

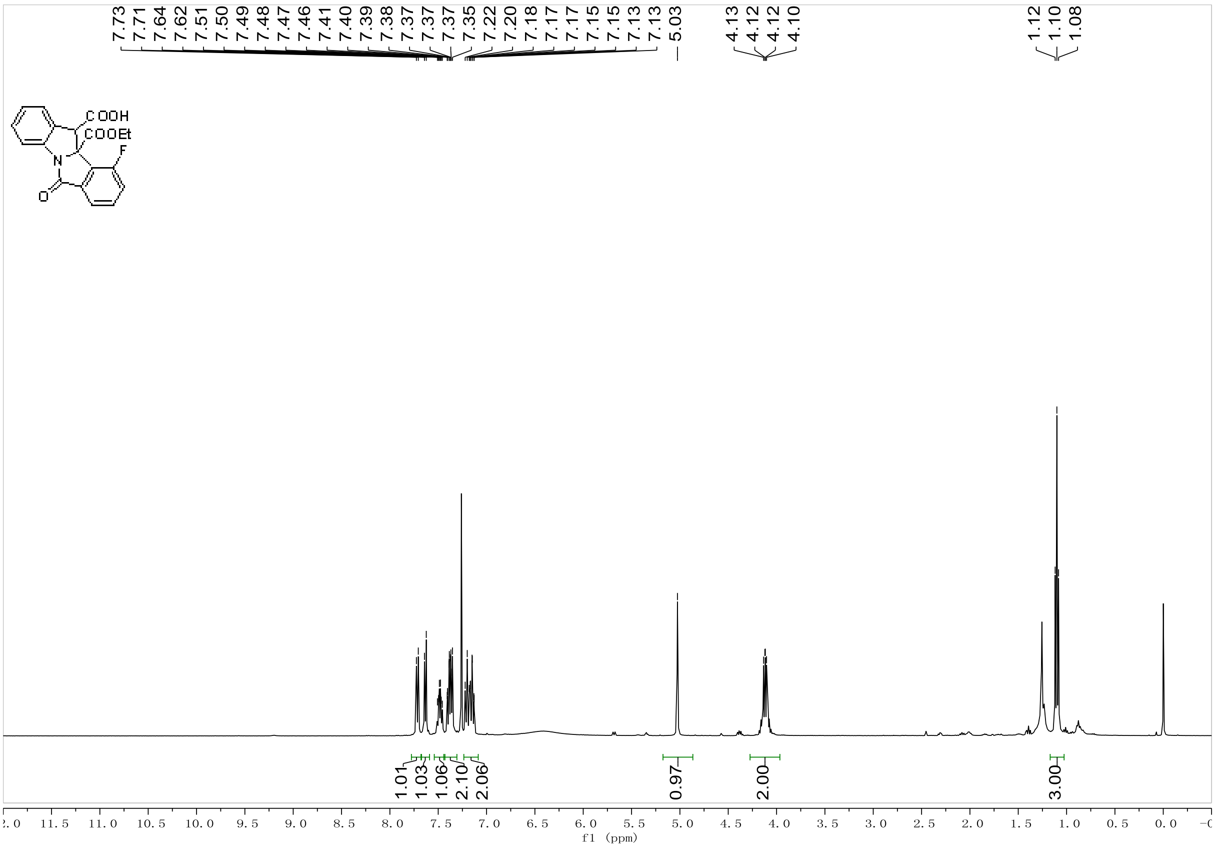

Supplementary Figure 64. ^{1}H NMR Spectra of compound **27**.

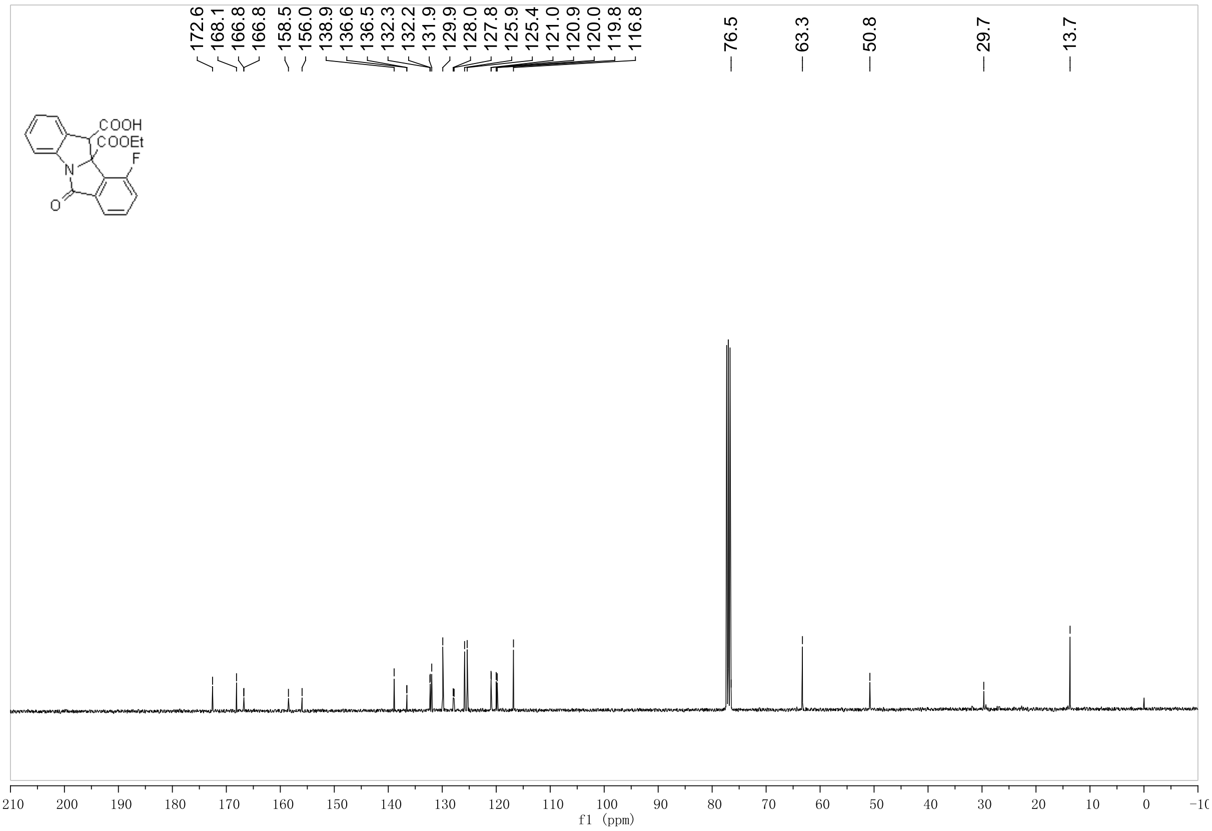

Supplementary Figure 65. ^{13}C NMR Spectra of compound **27**.

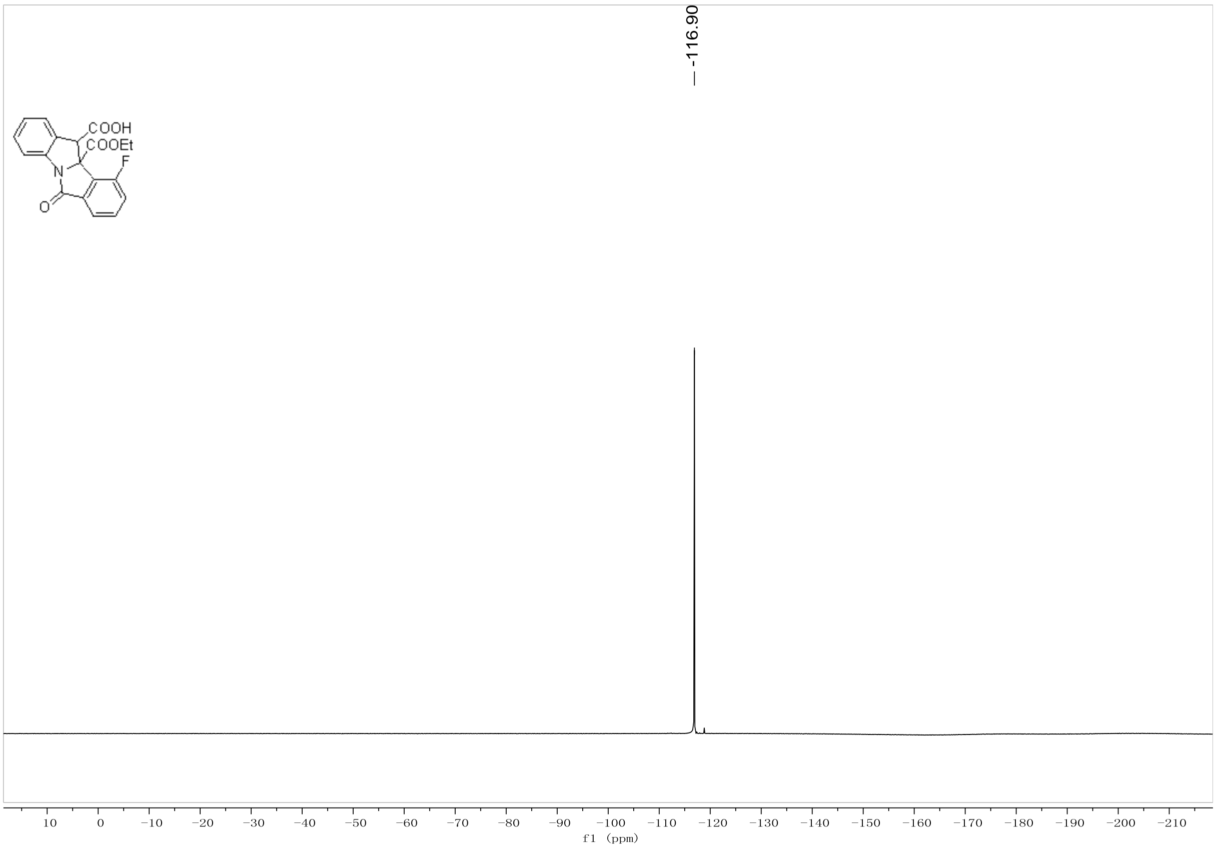

Supplementary Figure 66. ^{19}F NMR Spectra of compound **27**.

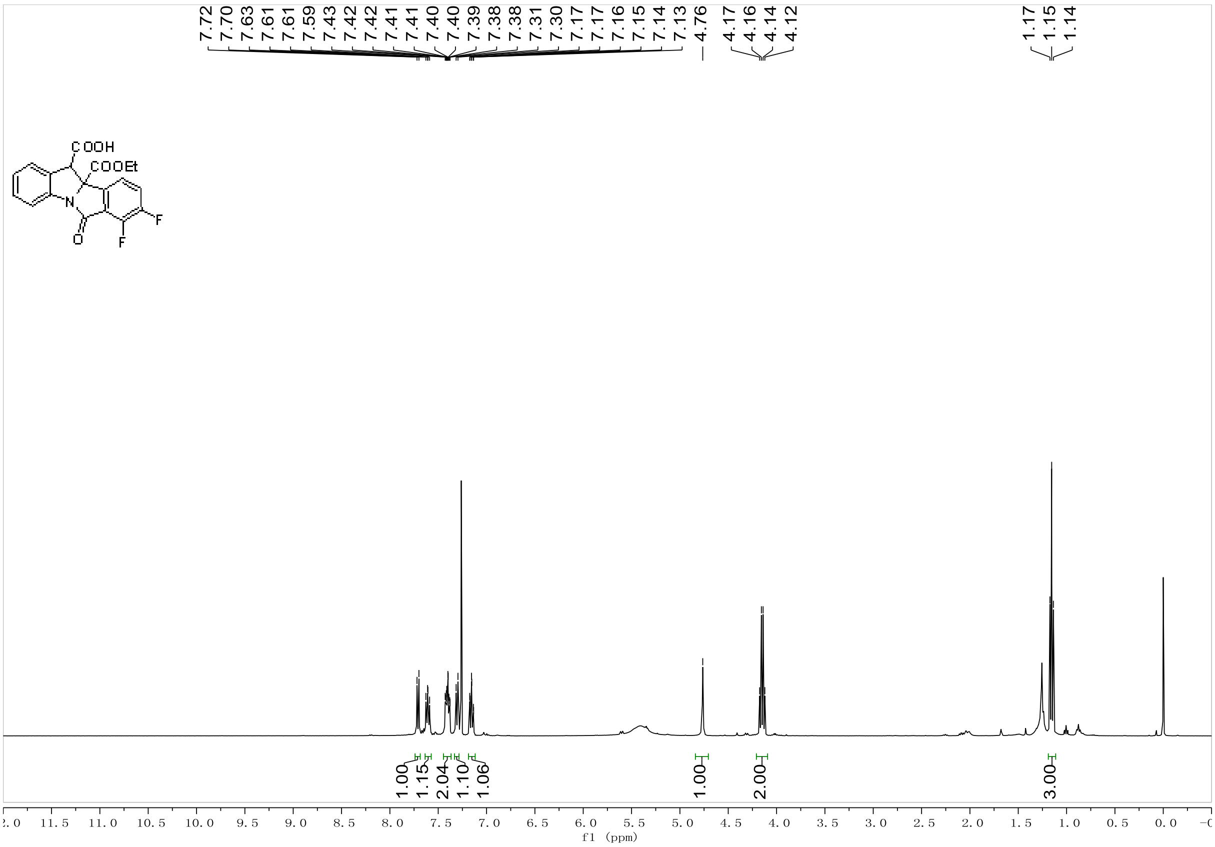

Supplementary Figure 67. ^{1}H NMR Spectra of compound **28**.

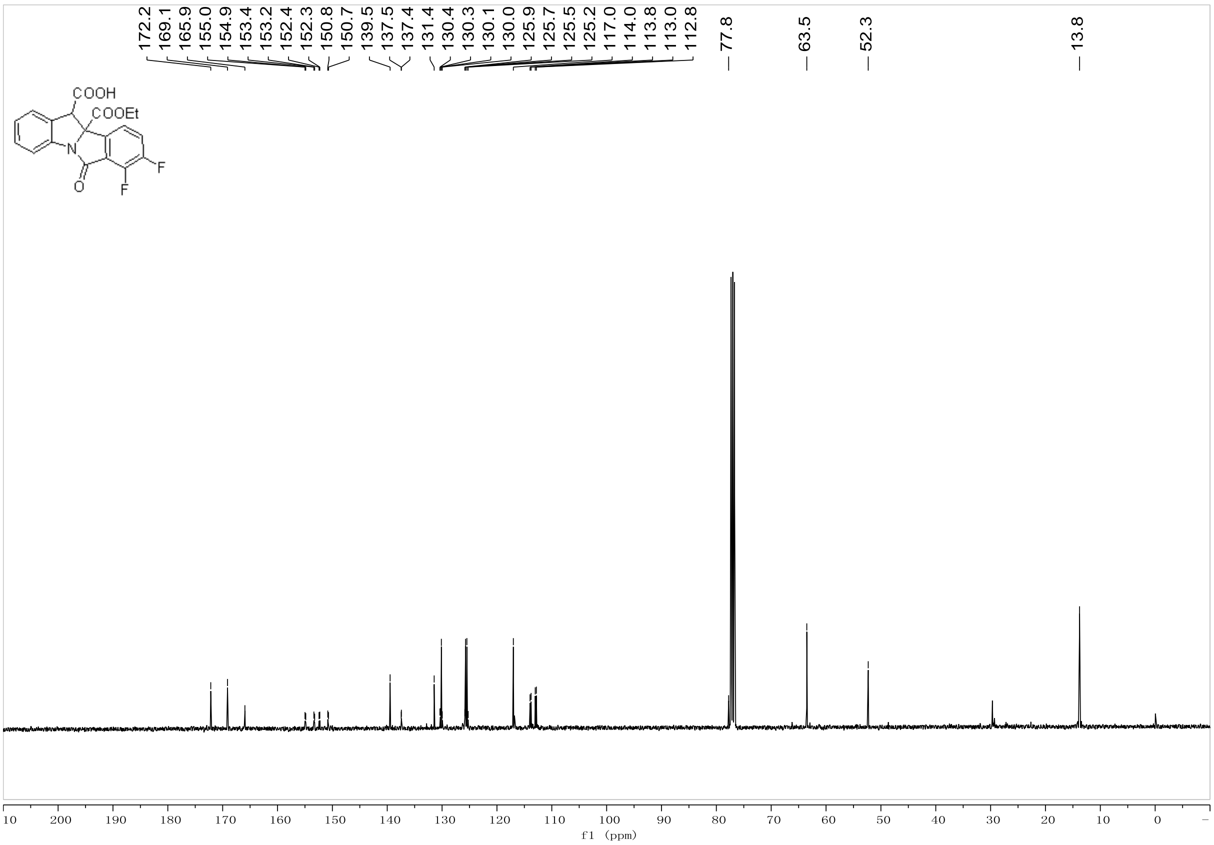

Supplementary Figure 68. ^{13}C NMR Spectra of compound **28**.

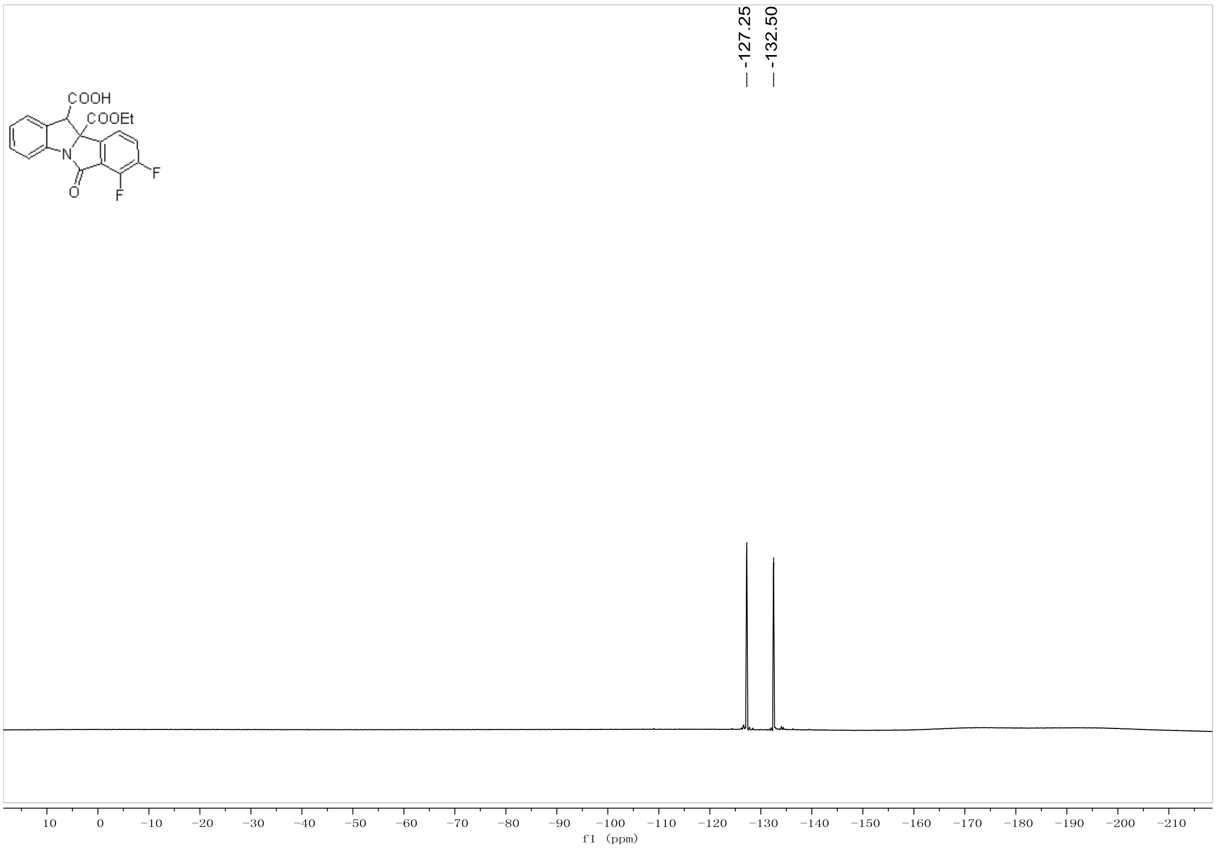

Supplementary Figure 69. ^{19}F NMR Spectra of compound **28**.

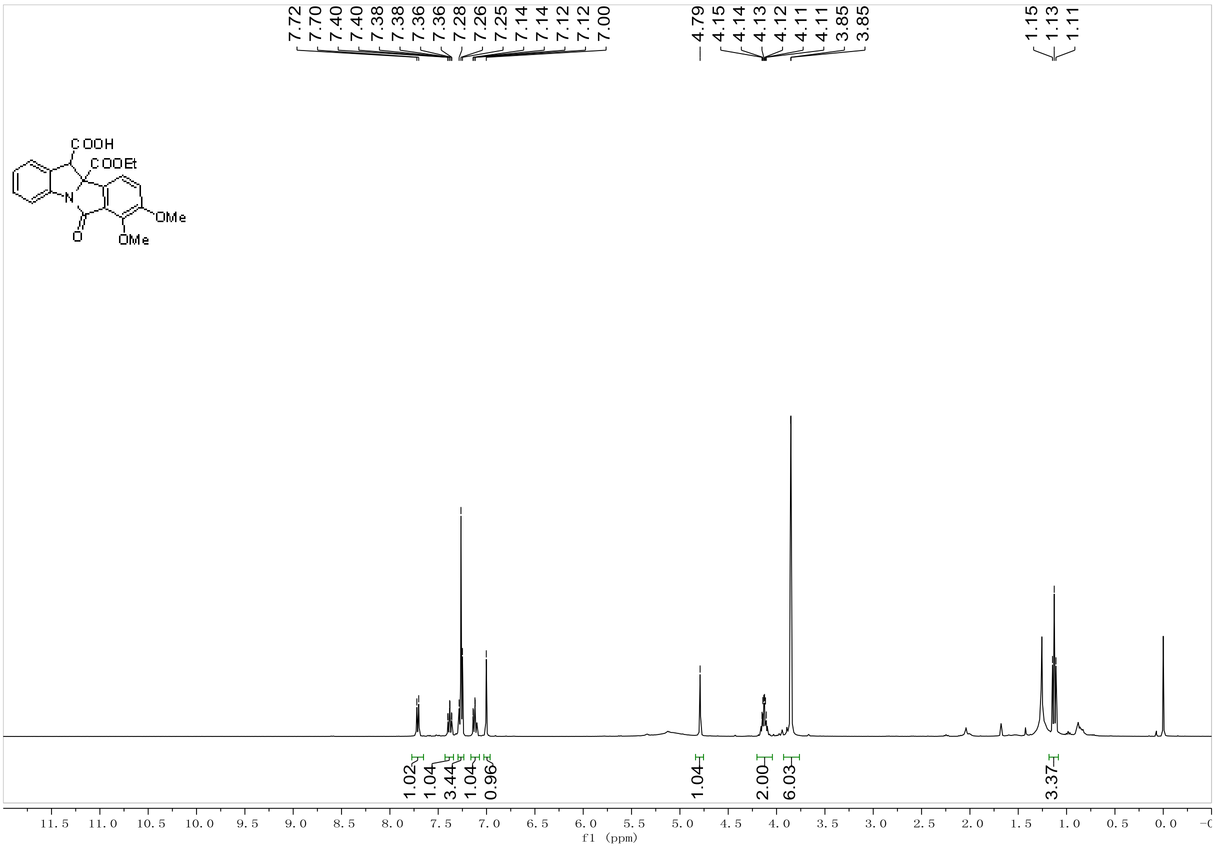

Supplementary Figure 70. ^{1}H NMR Spectra of compound **29**.

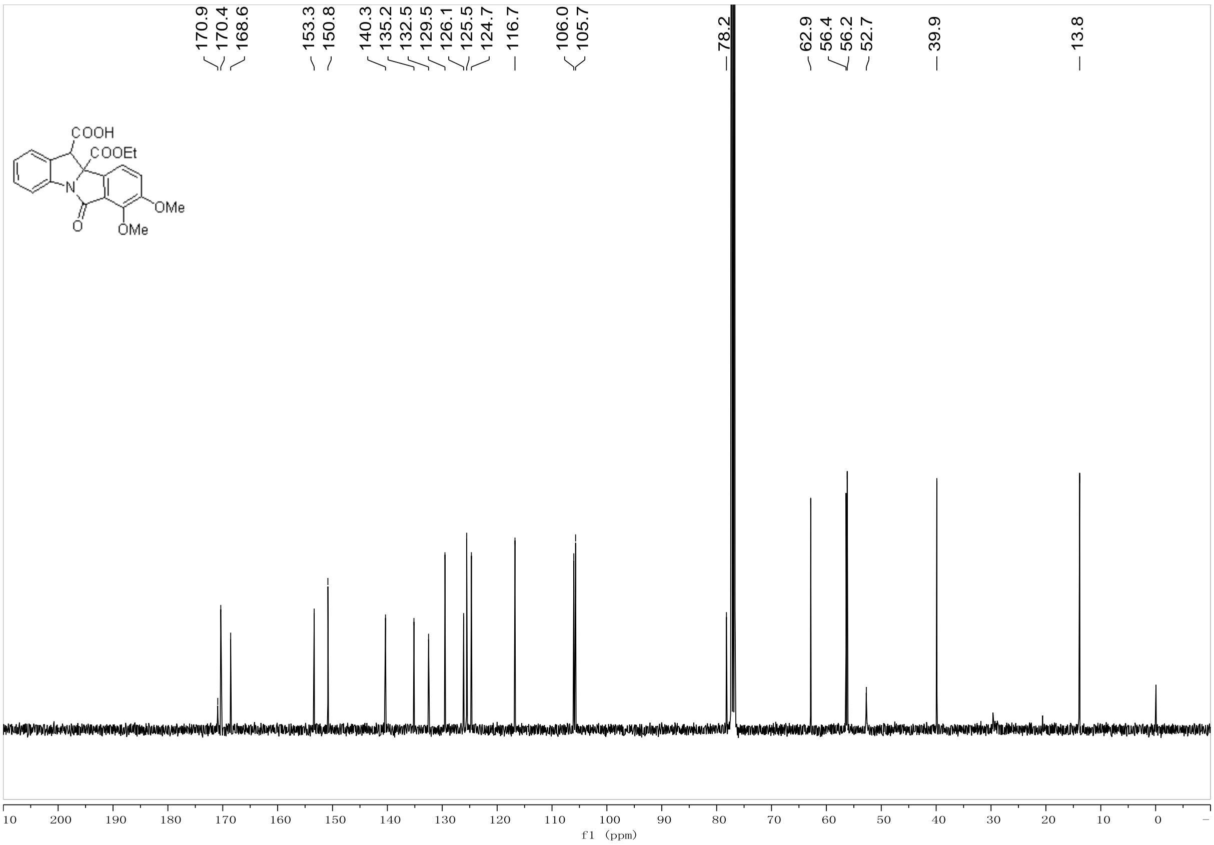

Supplementary Figure 71. ^{13}C NMR Spectra of compound **29**.

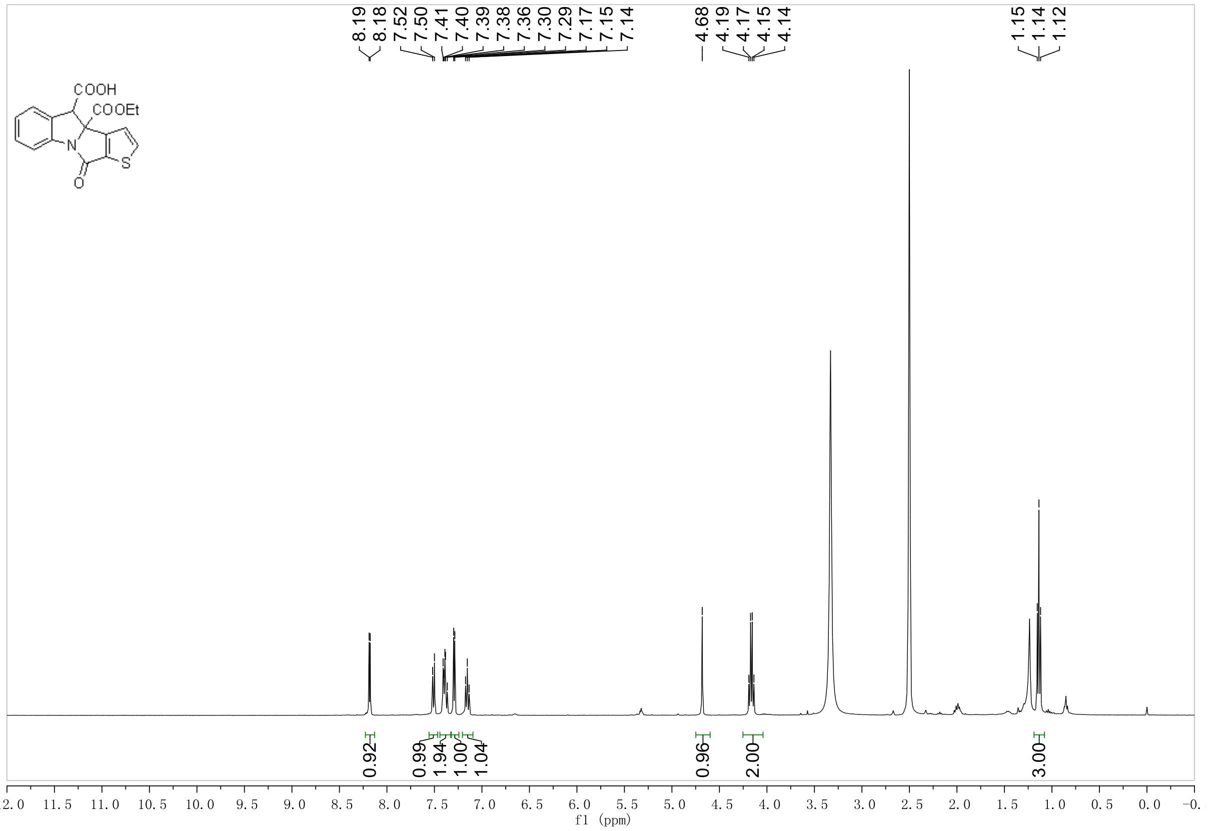

Supplementary Figure 72. ^{1}H NMR Spectra of compound **30**.

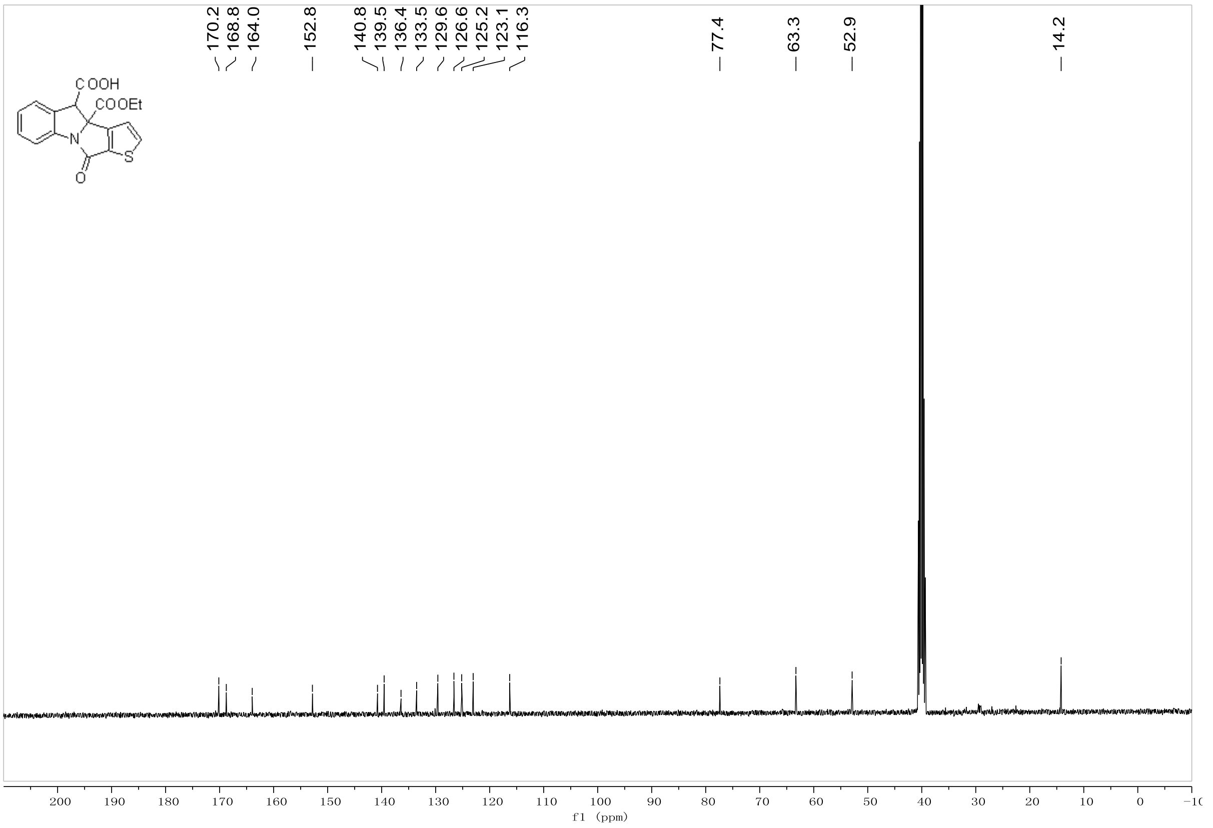

Supplementary Figure 73. ^{13}C NMR Spectra of compound **30**.

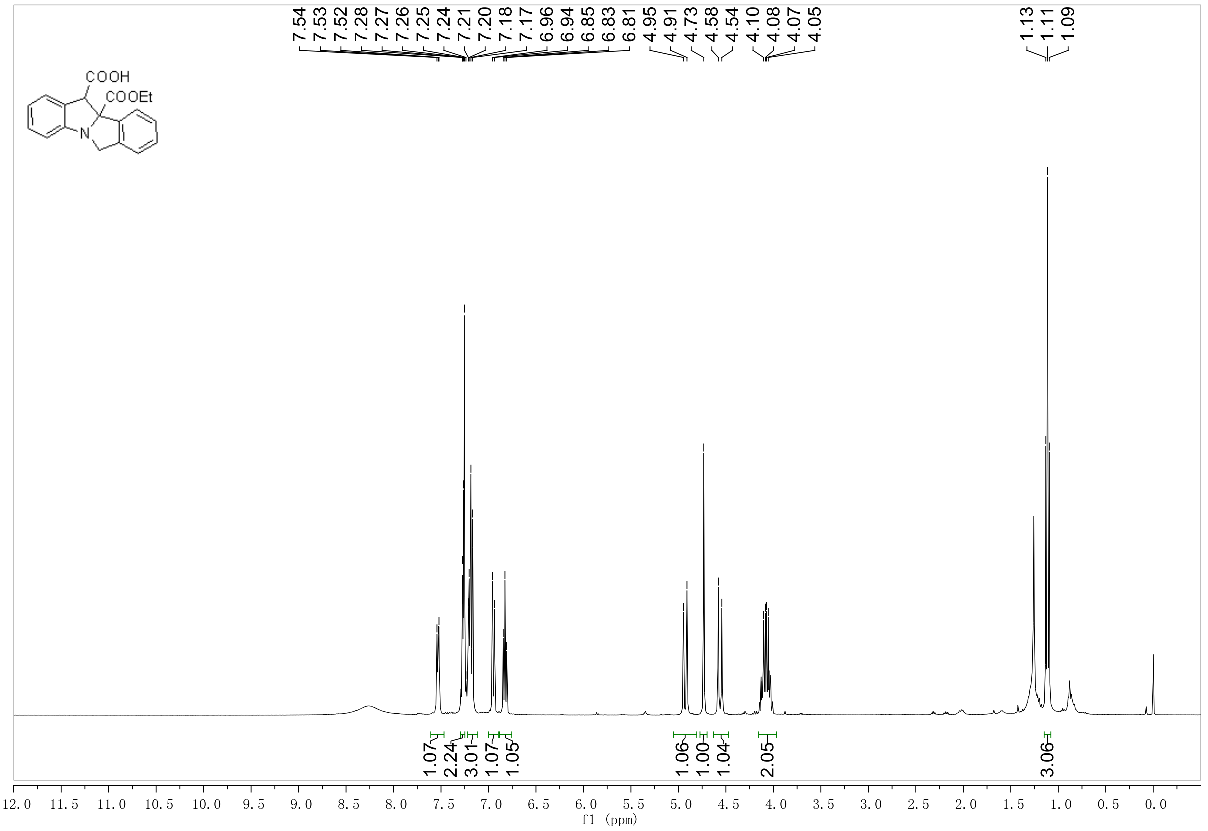

Supplementary Figure 74. ^{1}H NMR Spectra of compound **31**.

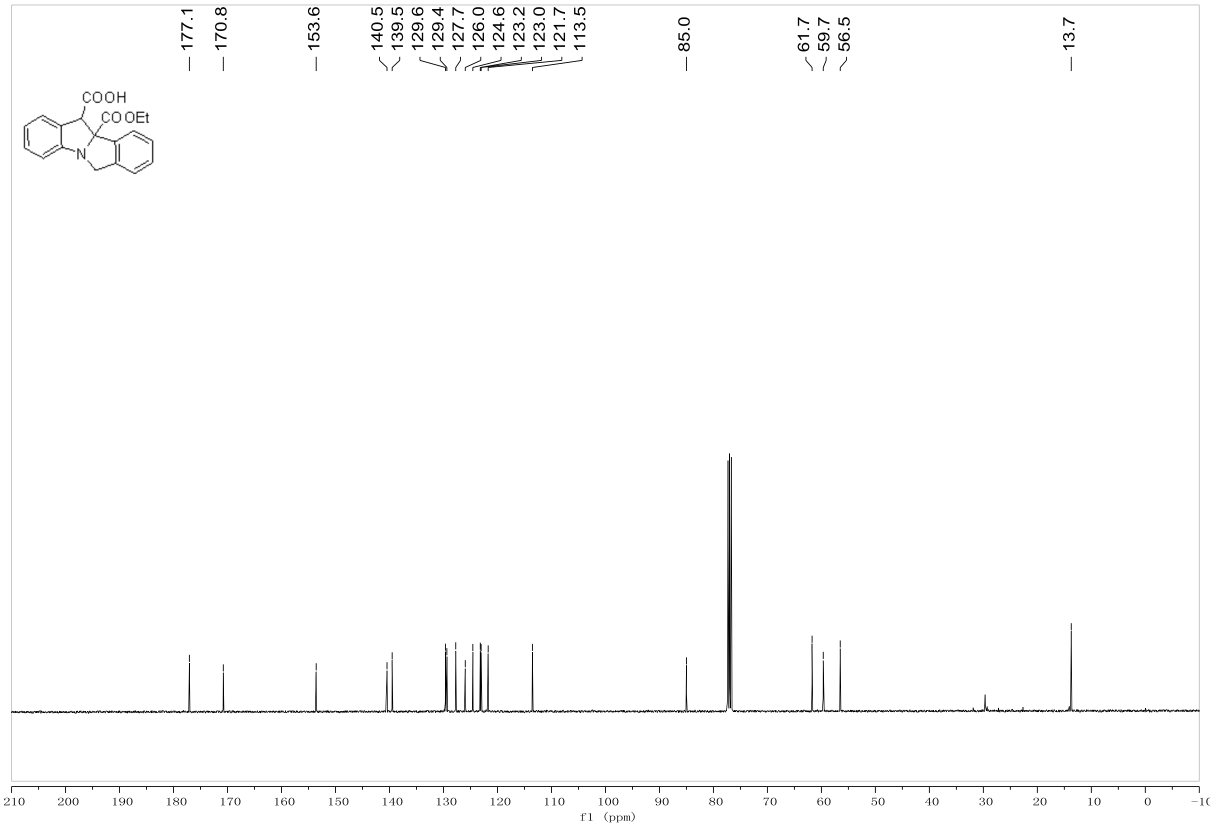

Supplementary Figure 75. ^{13}C NMR Spectra of compound **31**.

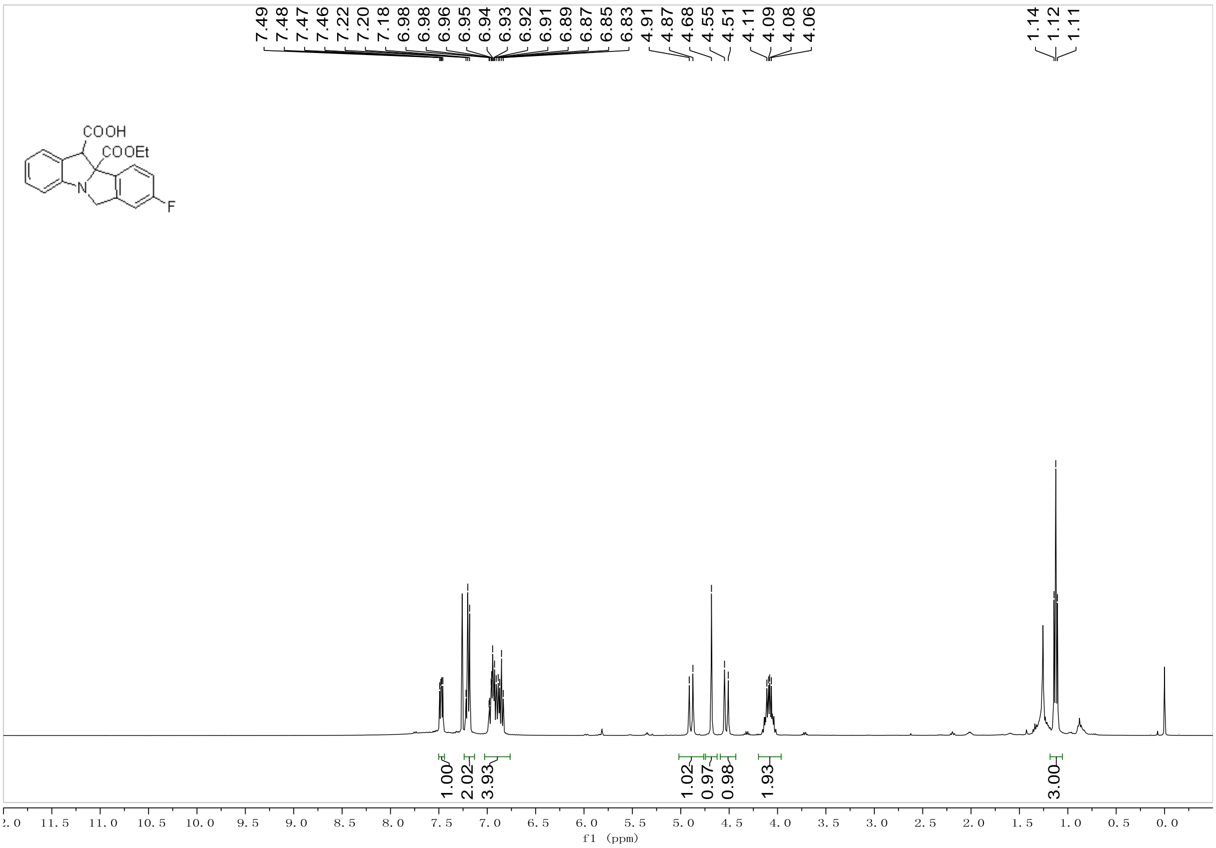

Supplementary Figure 76. ^{1}H NMR Spectra of compound **32**.

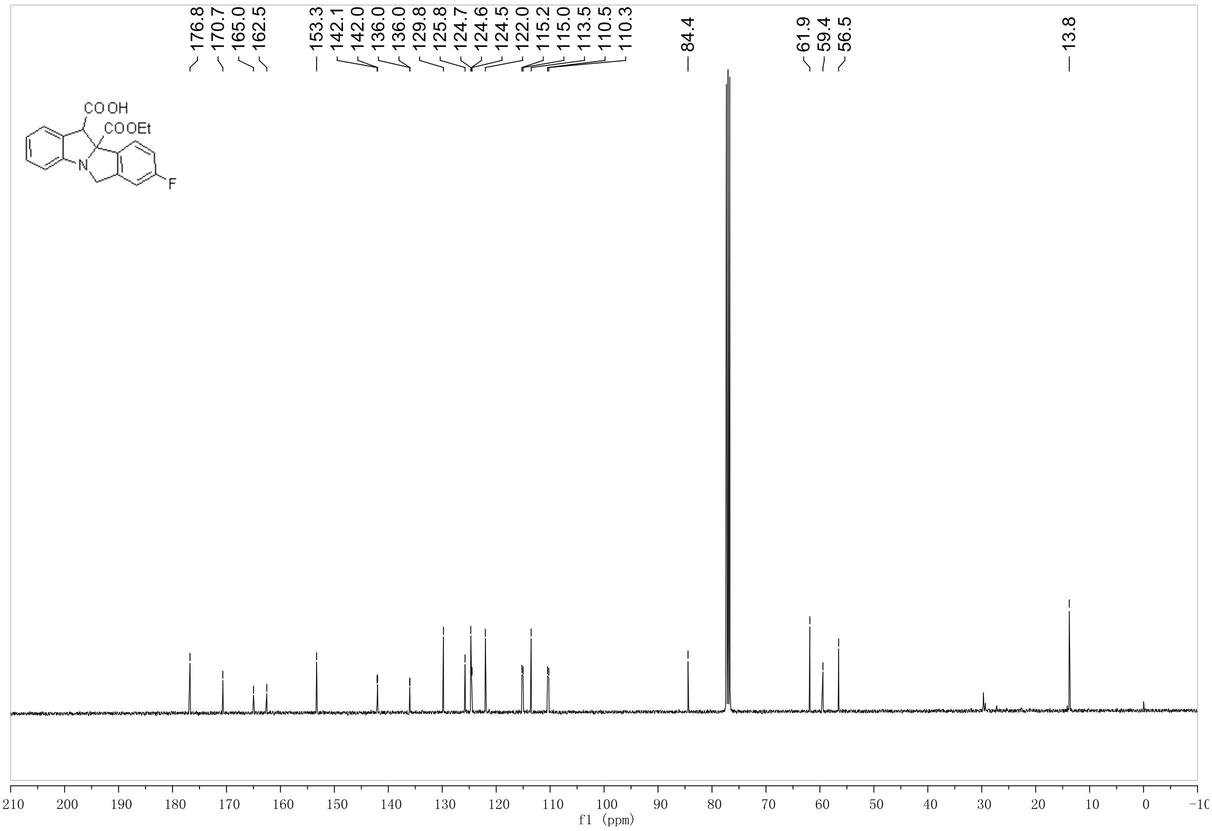

Supplementary Figure 77. ^{13}C NMR Spectra of compound **32**.

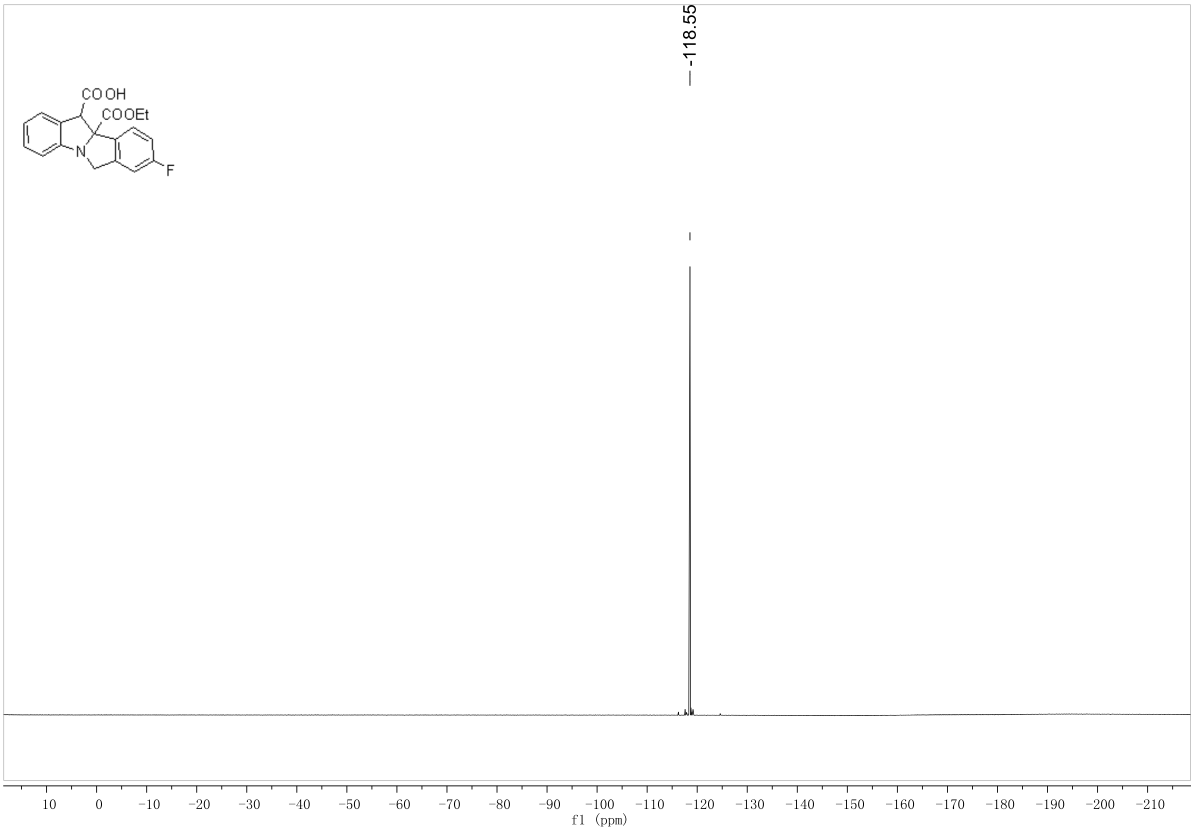

Supplementary Figure 78. ^{19}F NMR Spectra of compound **32**.

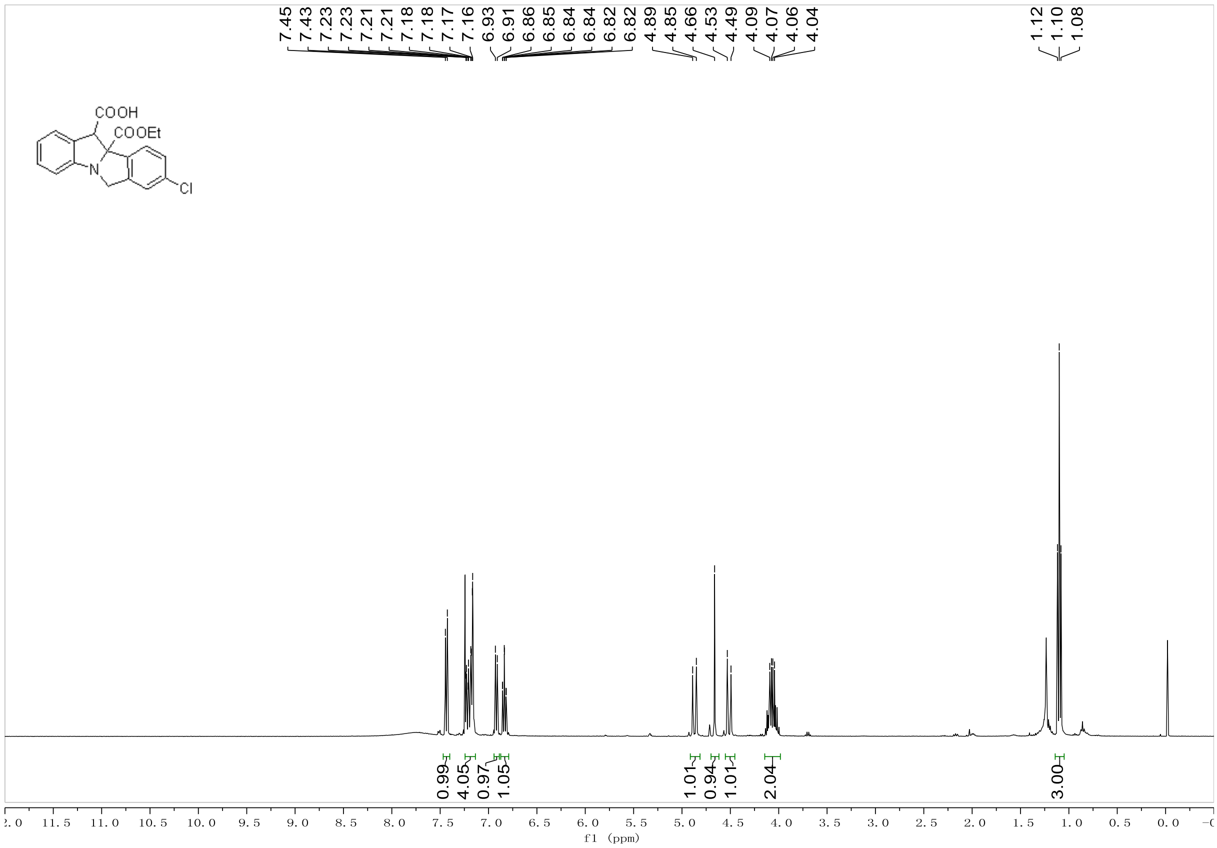

Supplementary Figure 79. ^{1}H NMR Spectra of compound **33**.

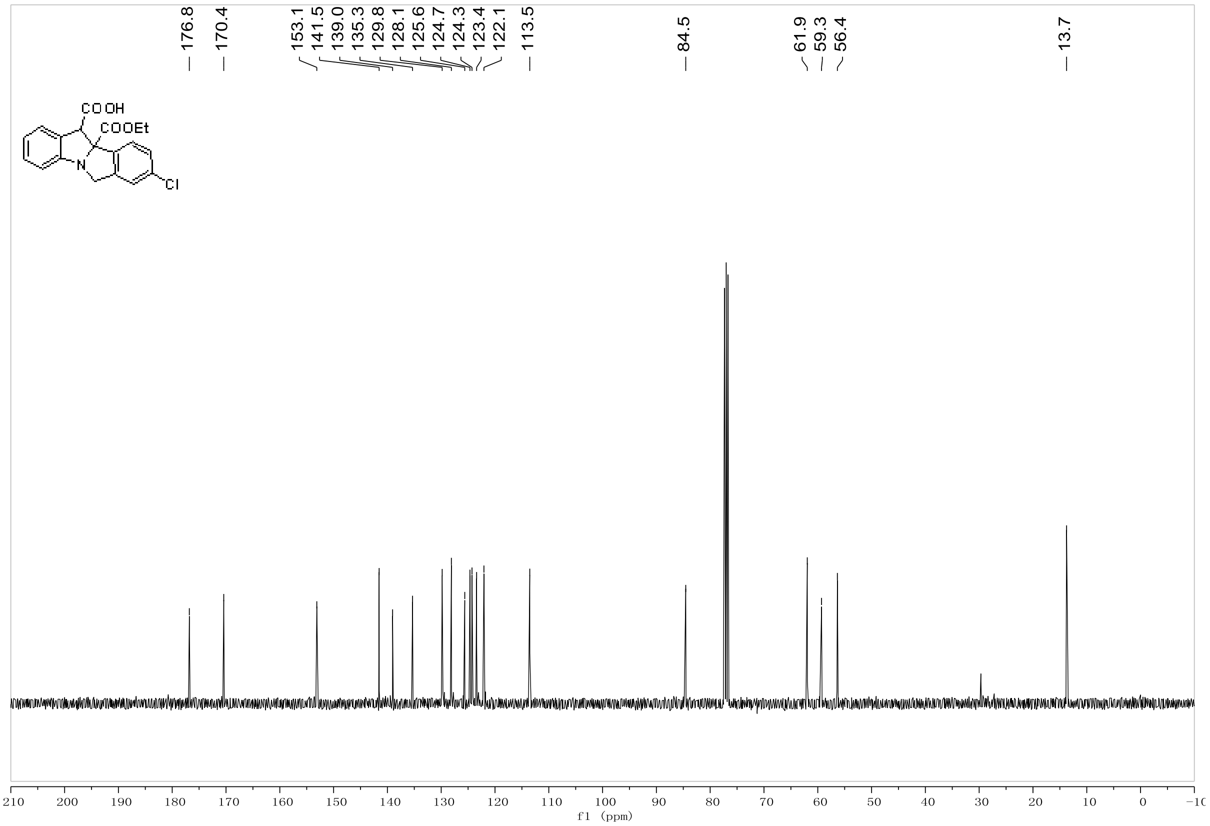

Supplementary Figure 80. ^{13}C NMR Spectra of compound **33**.

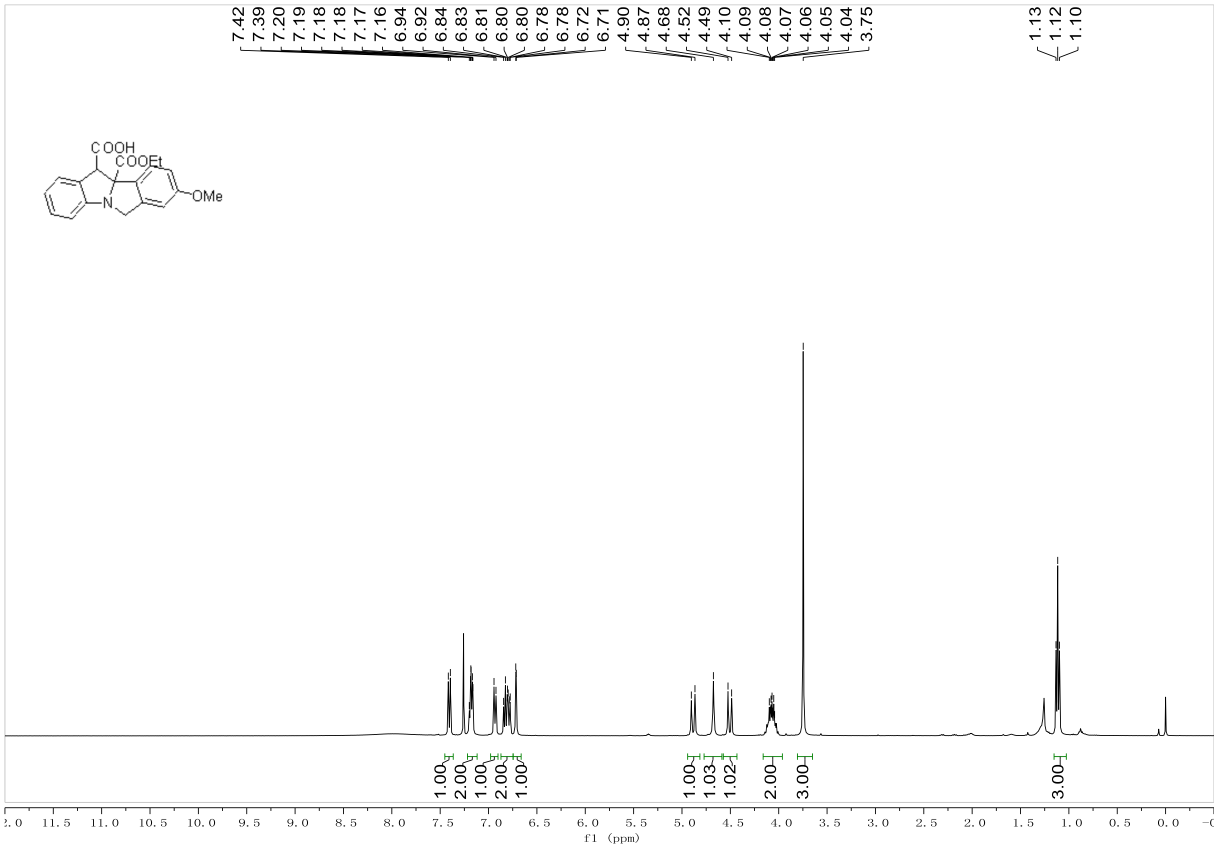

Supplementary Figure 81. ^{1}H NMR Spectra of compound **34.**

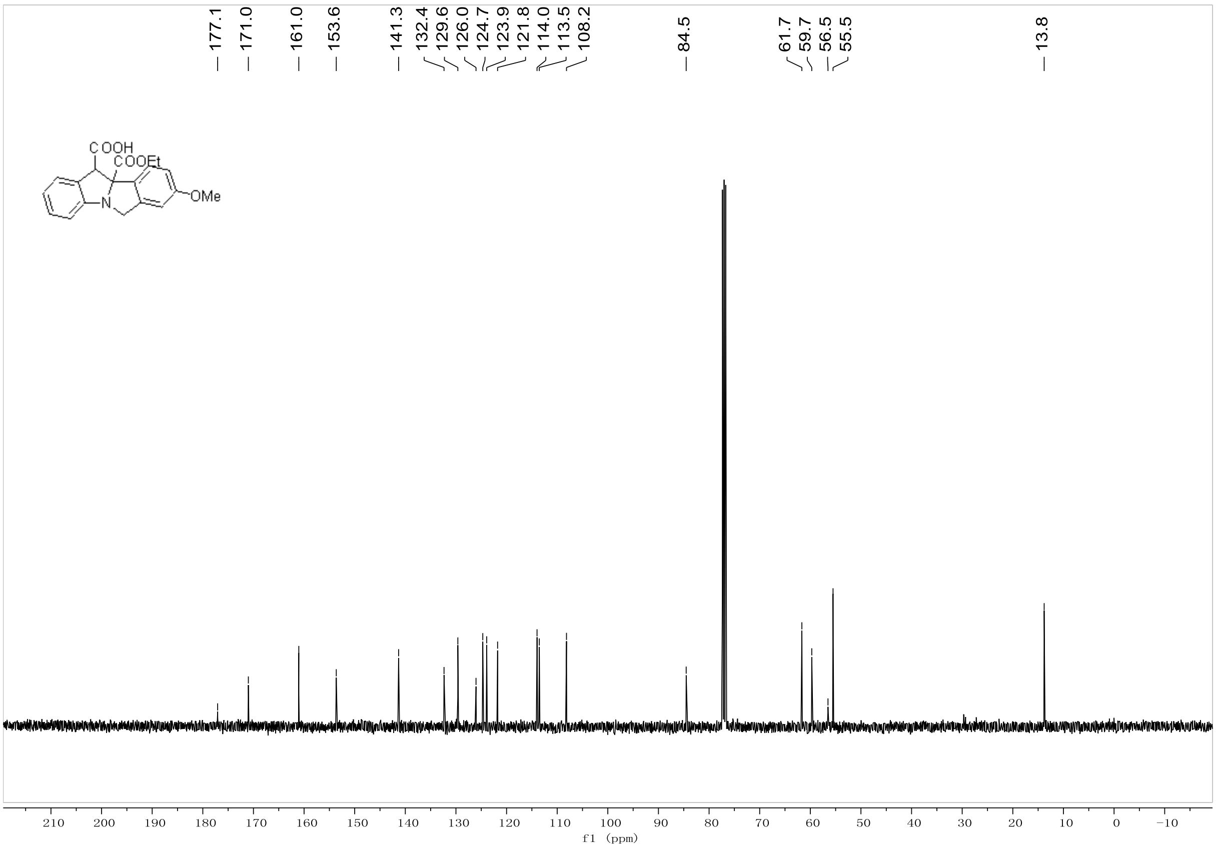

Supplementary Figure 82. ^{13}C NMR Spectra of compound **34**.

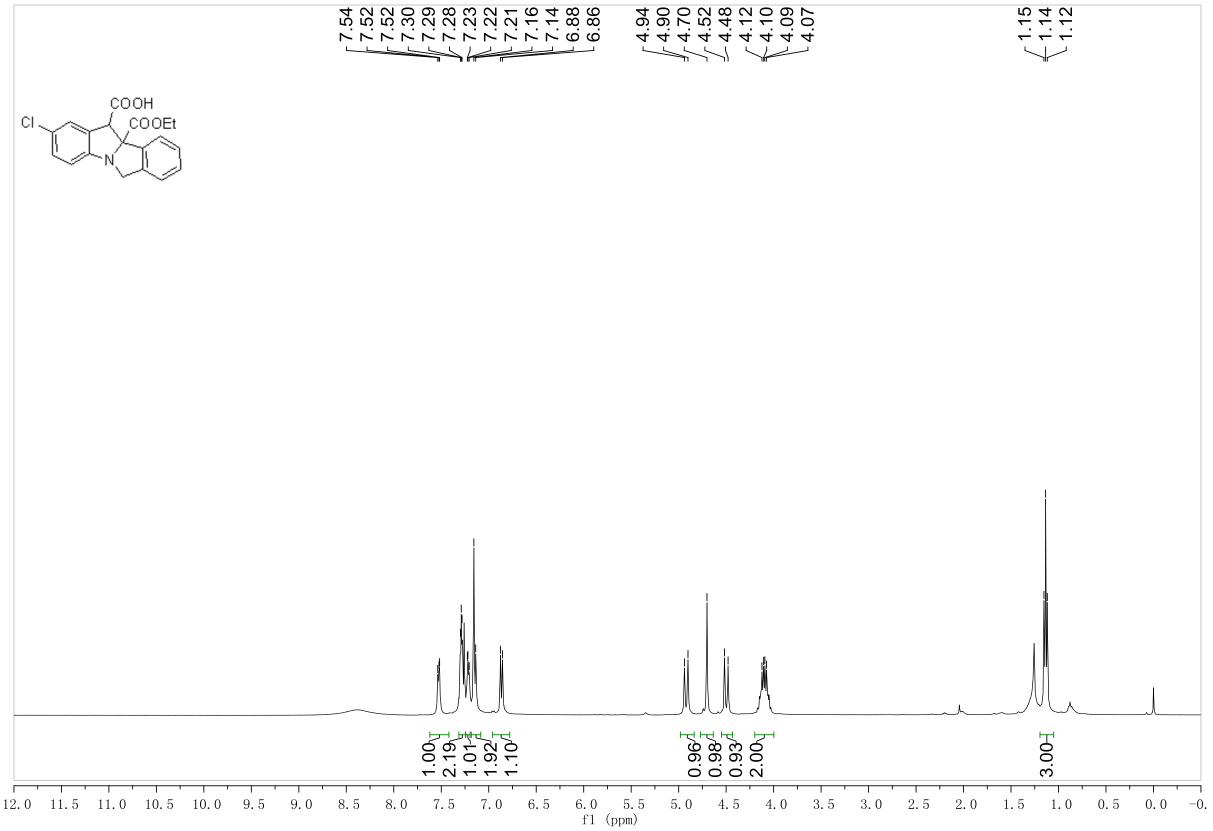

Supplementary Figure 83. ^{1}H NMR Spectra of compound **35**.

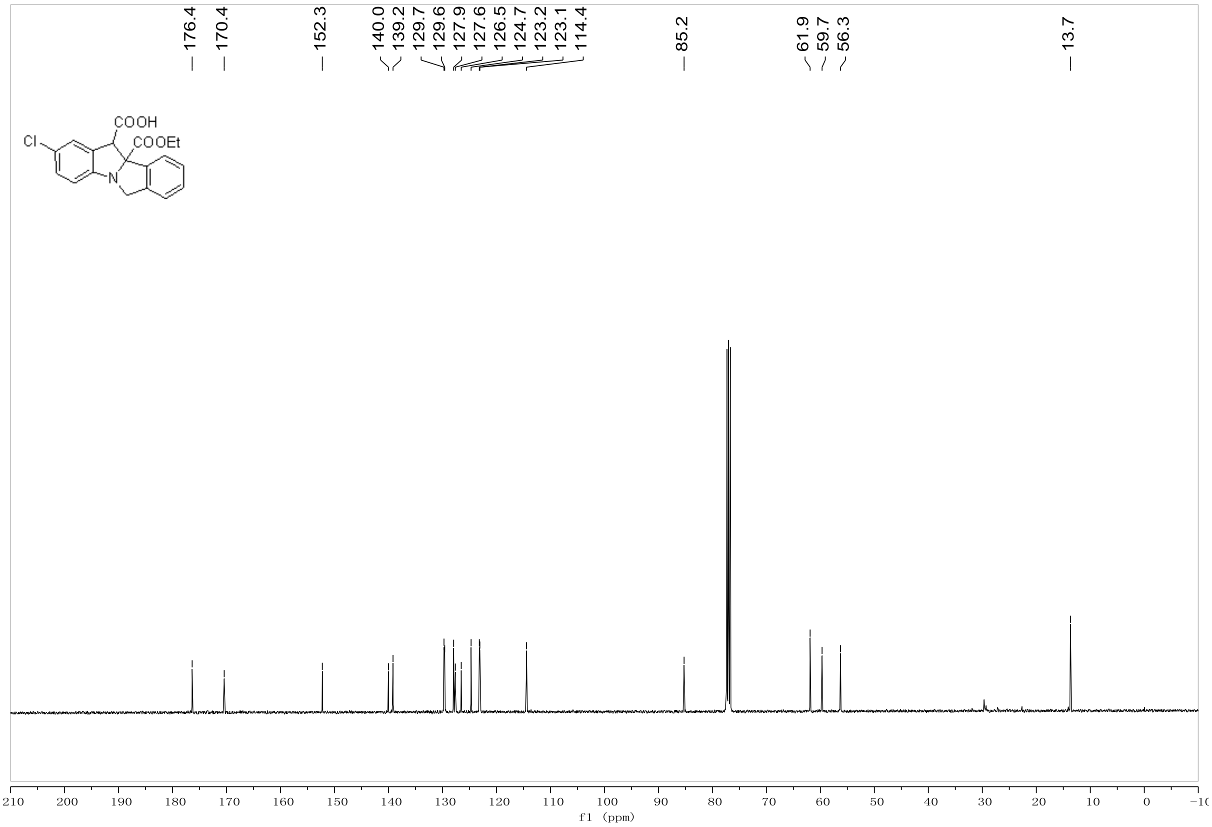

Supplementary Figure 84. ^{13}C NMR Spectra of compound **35**.

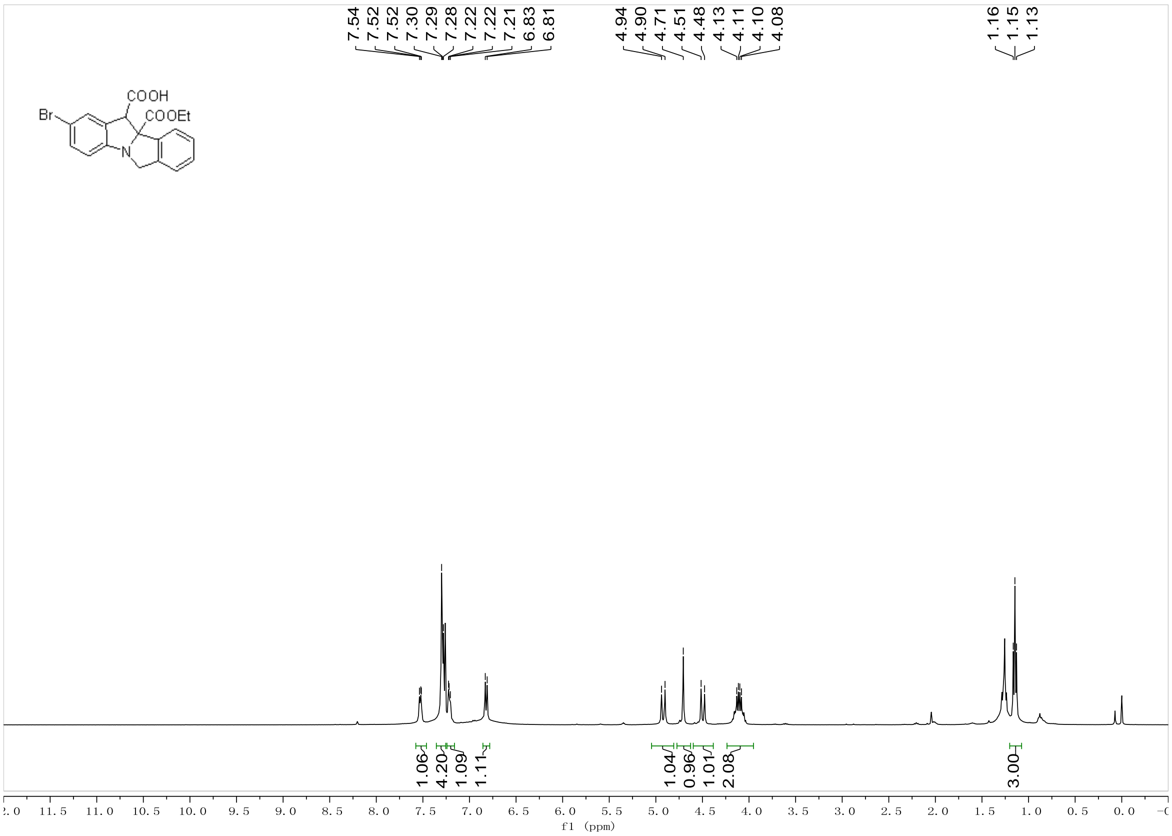

Supplementary Figure 85. ^{1}H NMR Spectra of compound **36**.

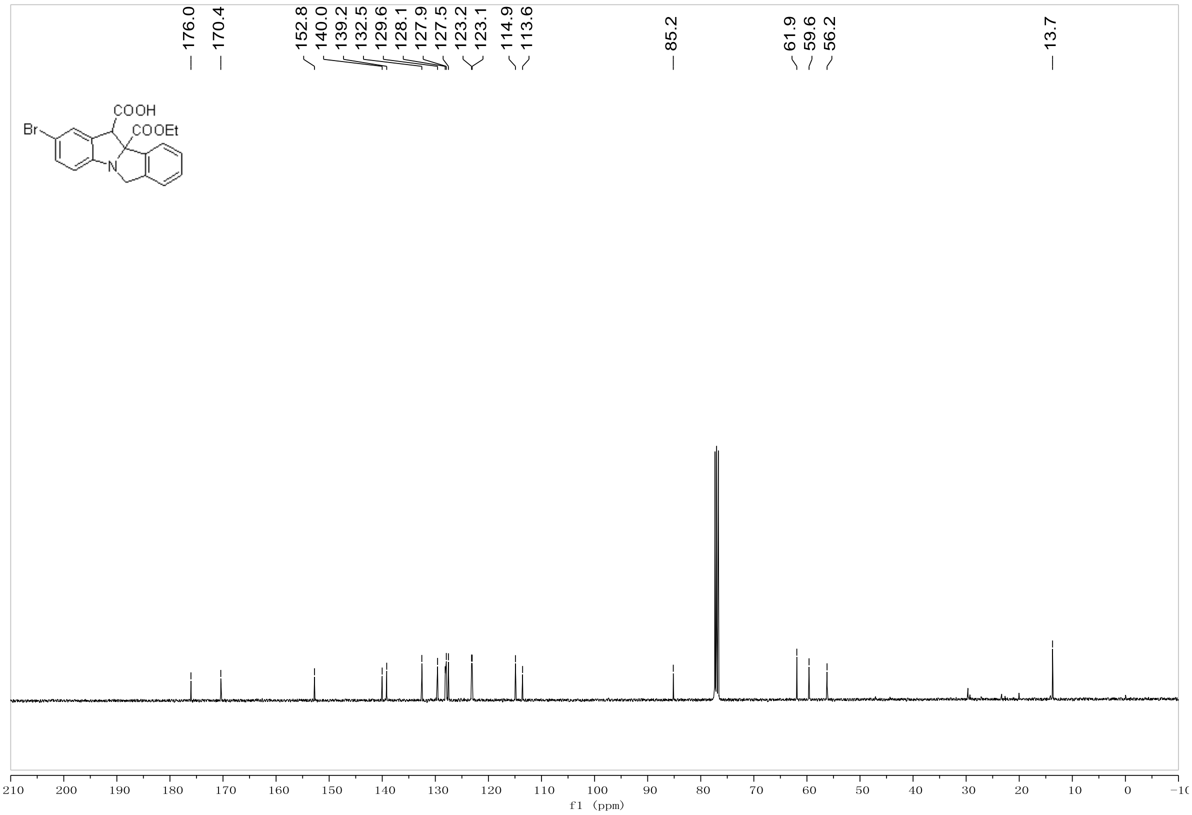

Supplementary Figure 86. ^{13}C NMR Spectra of compound **36**.

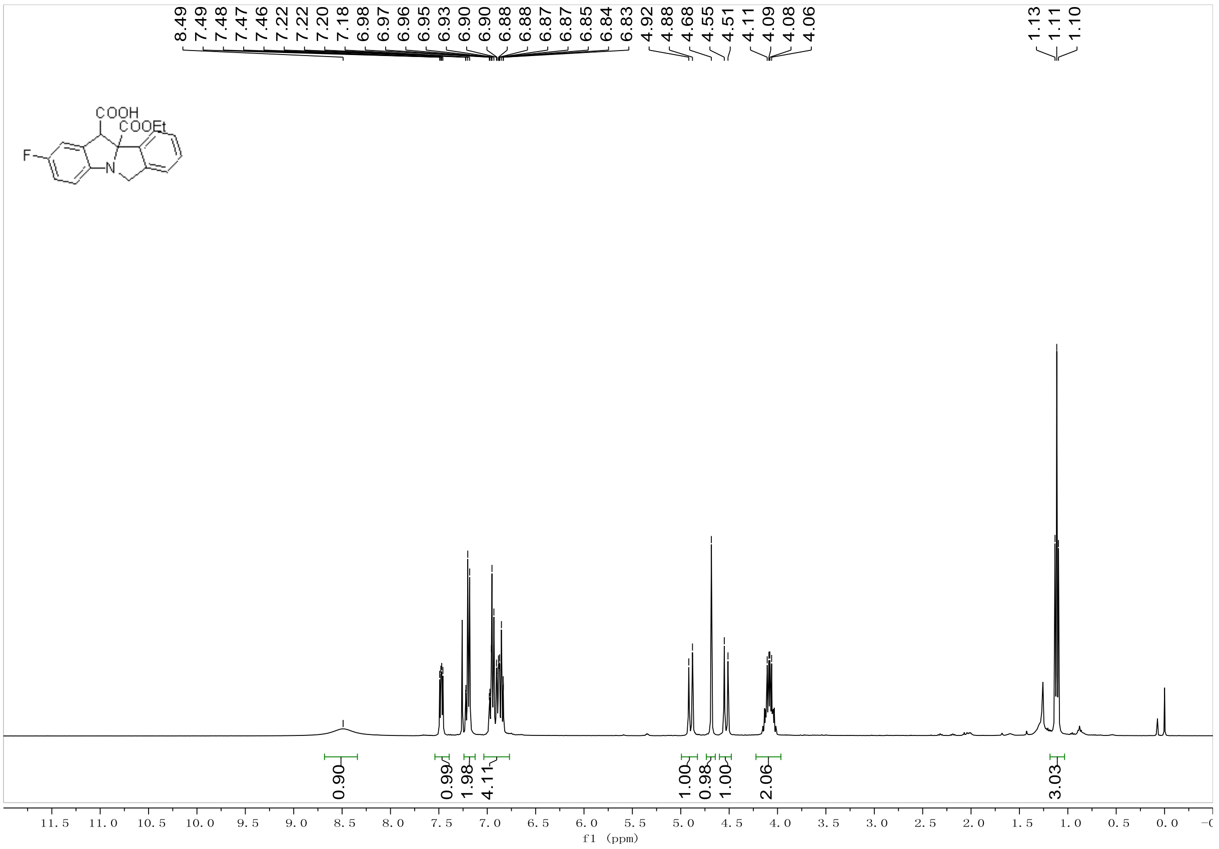

Supplementary Figure 87. ^{1}H NMR Spectra of compound **37**.

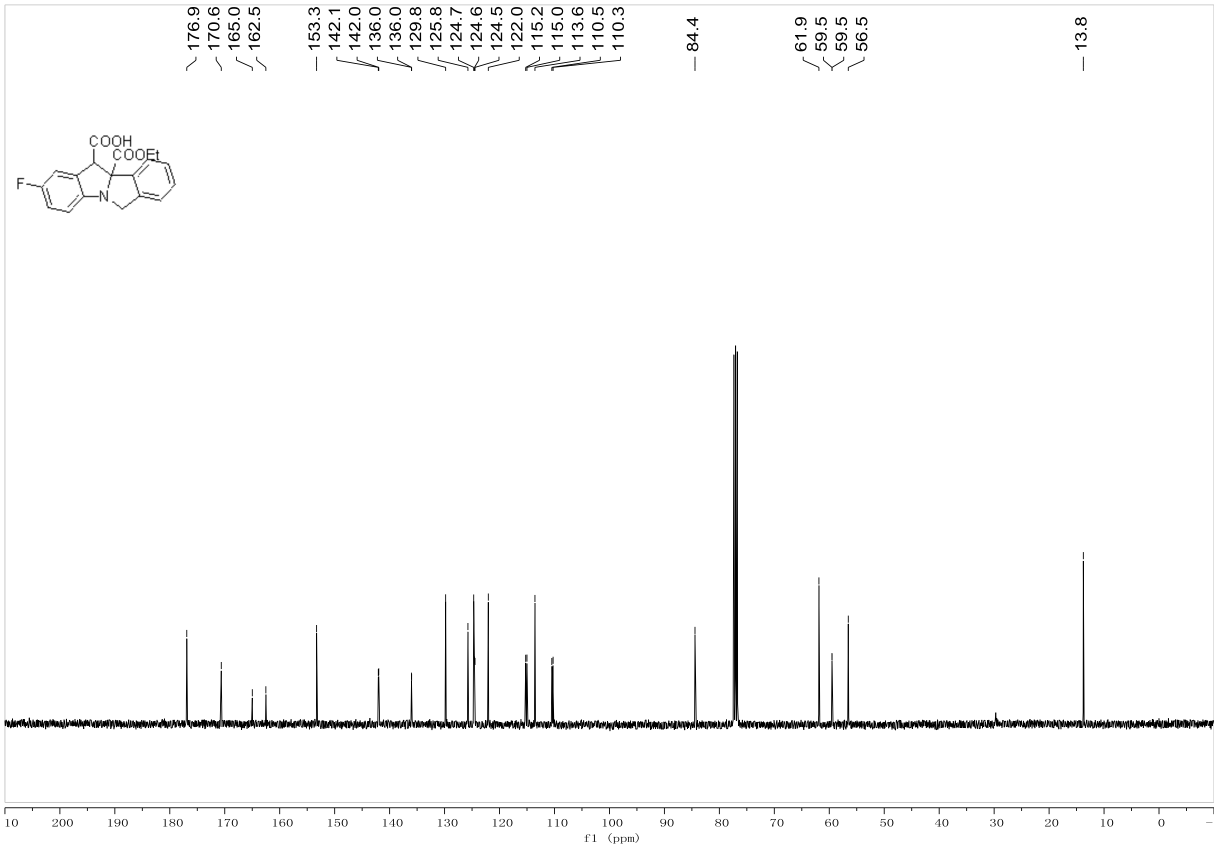

Supplementary Figure 88. ^{13}C NMR Spectra of compound **37**.

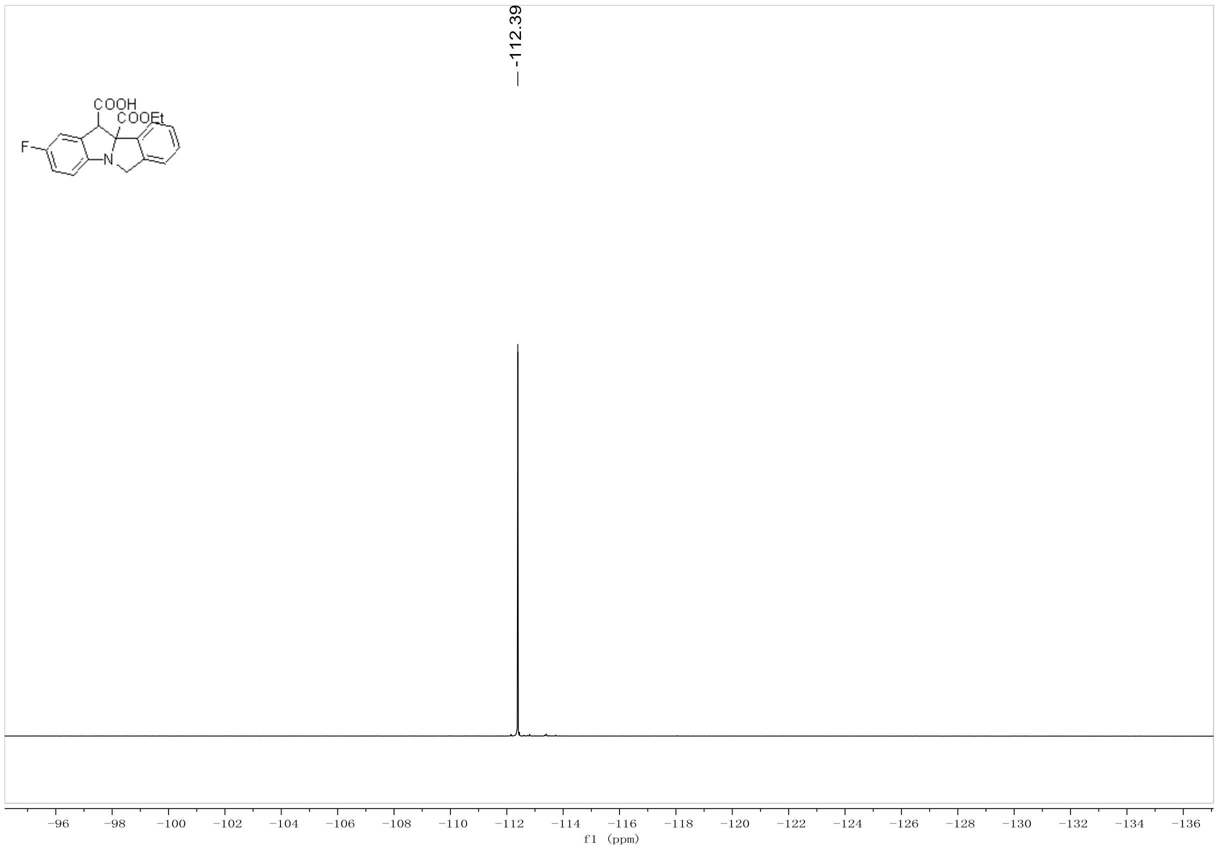

Supplementary Figure 89. ^{19}F NMR Spectra of compound **37**.

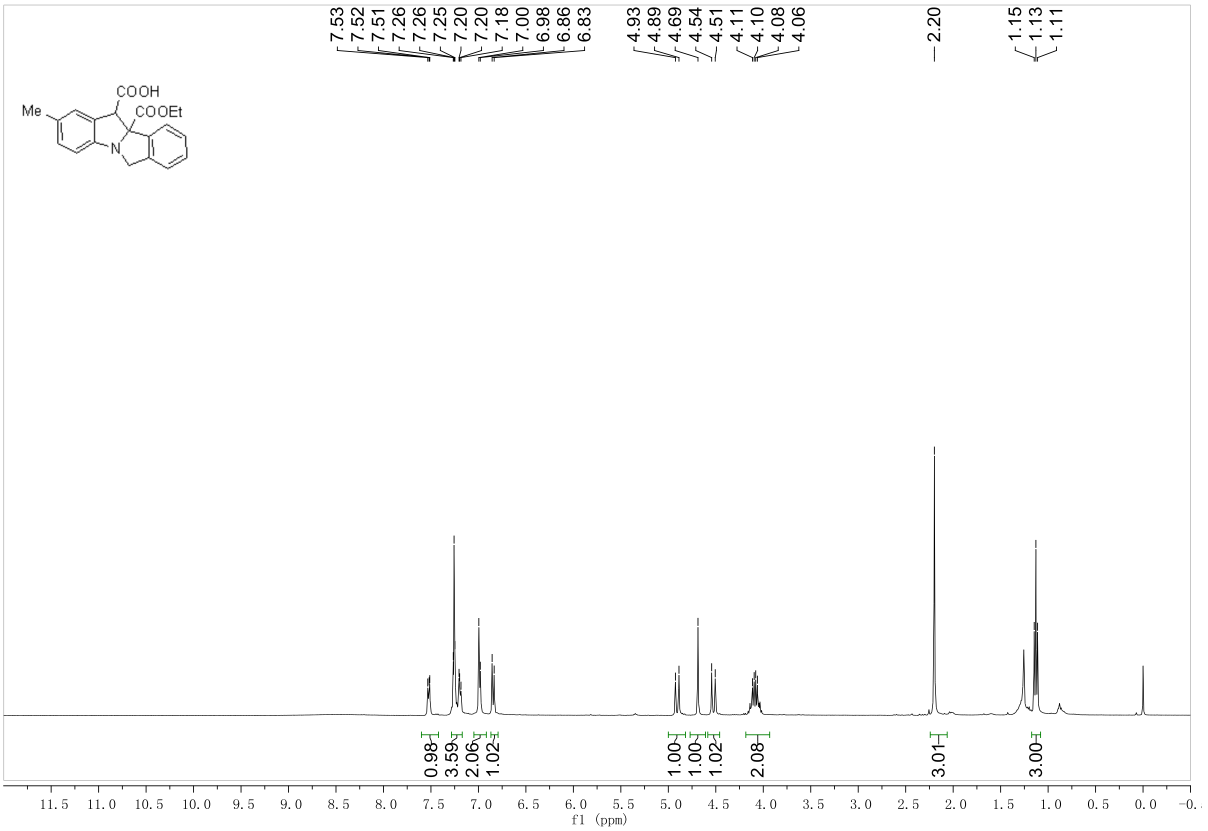

Supplementary Figure 90. ^{1}H NMR Spectra of compound **38**.

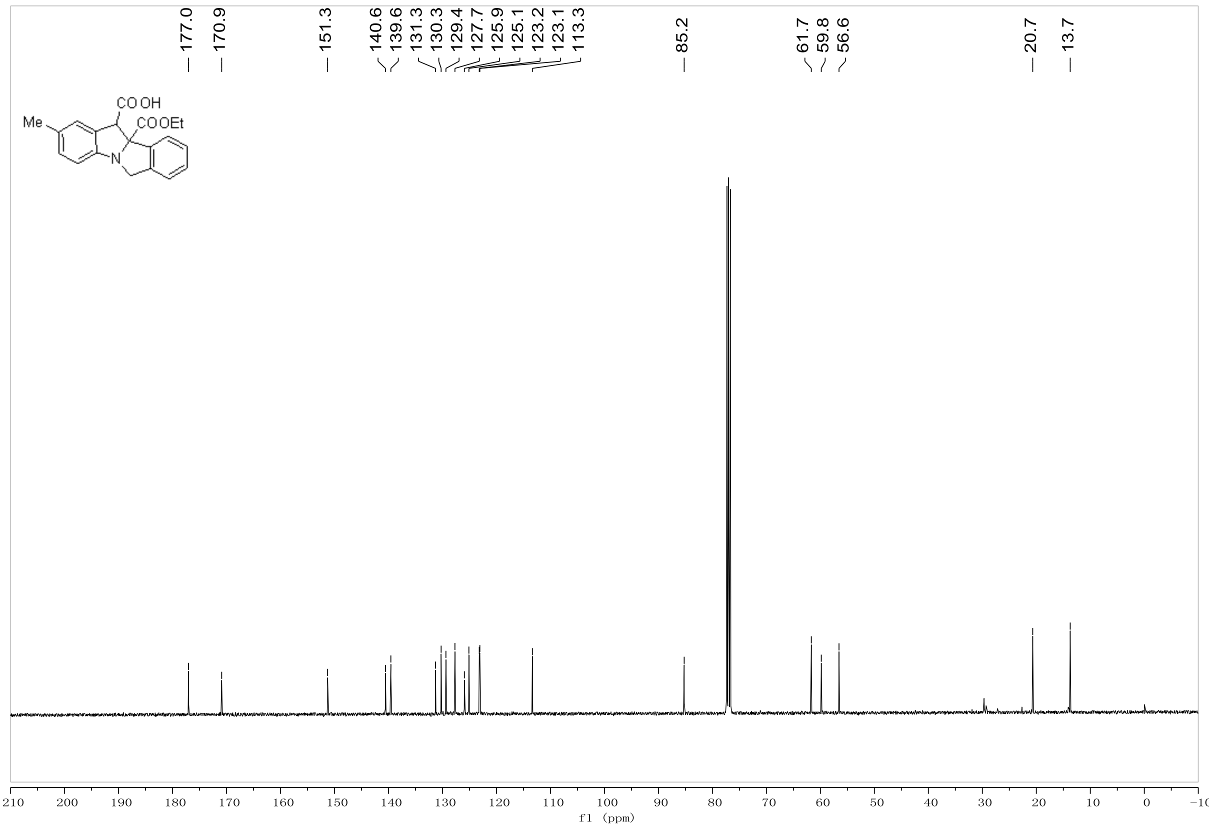

Supplementary Figure 91. ^{13}C NMR Spectra of compound **38**.

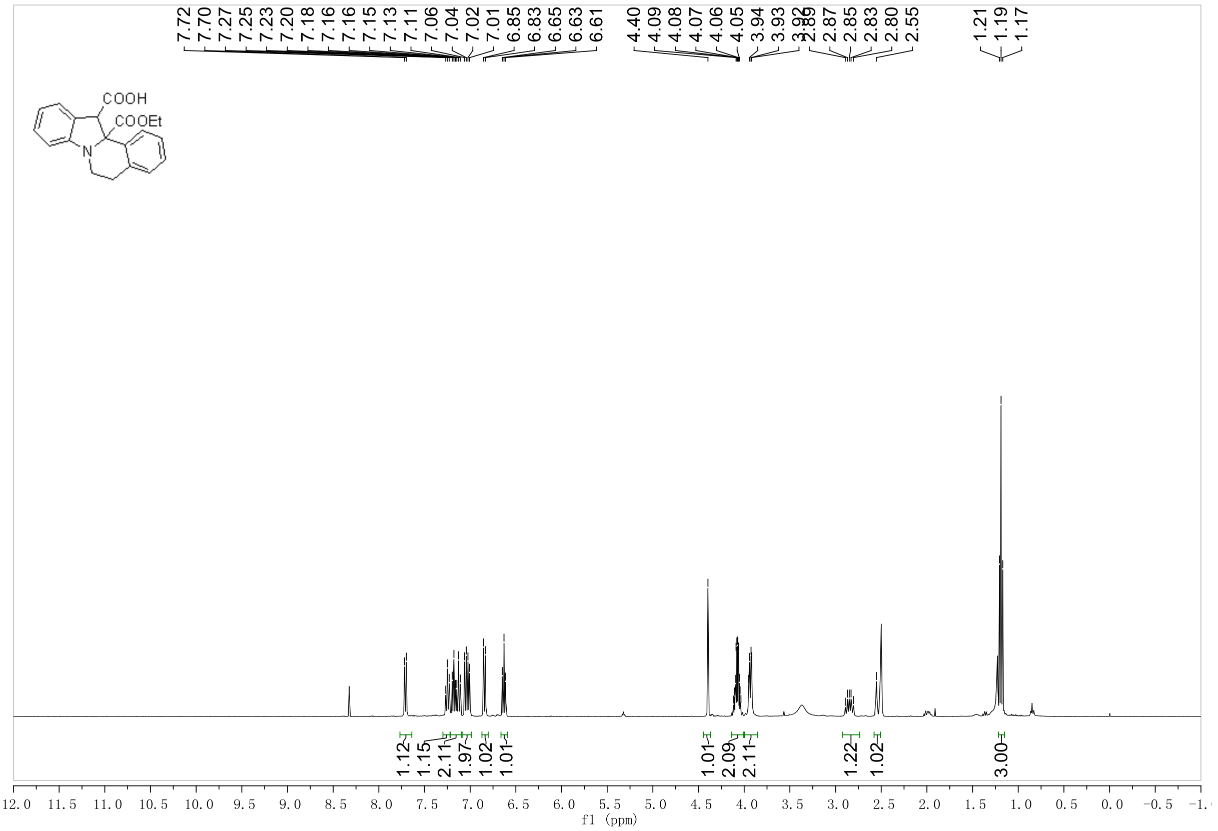

Supplementary Figure 92. ^{1}H NMR Spectra of compound **39**.

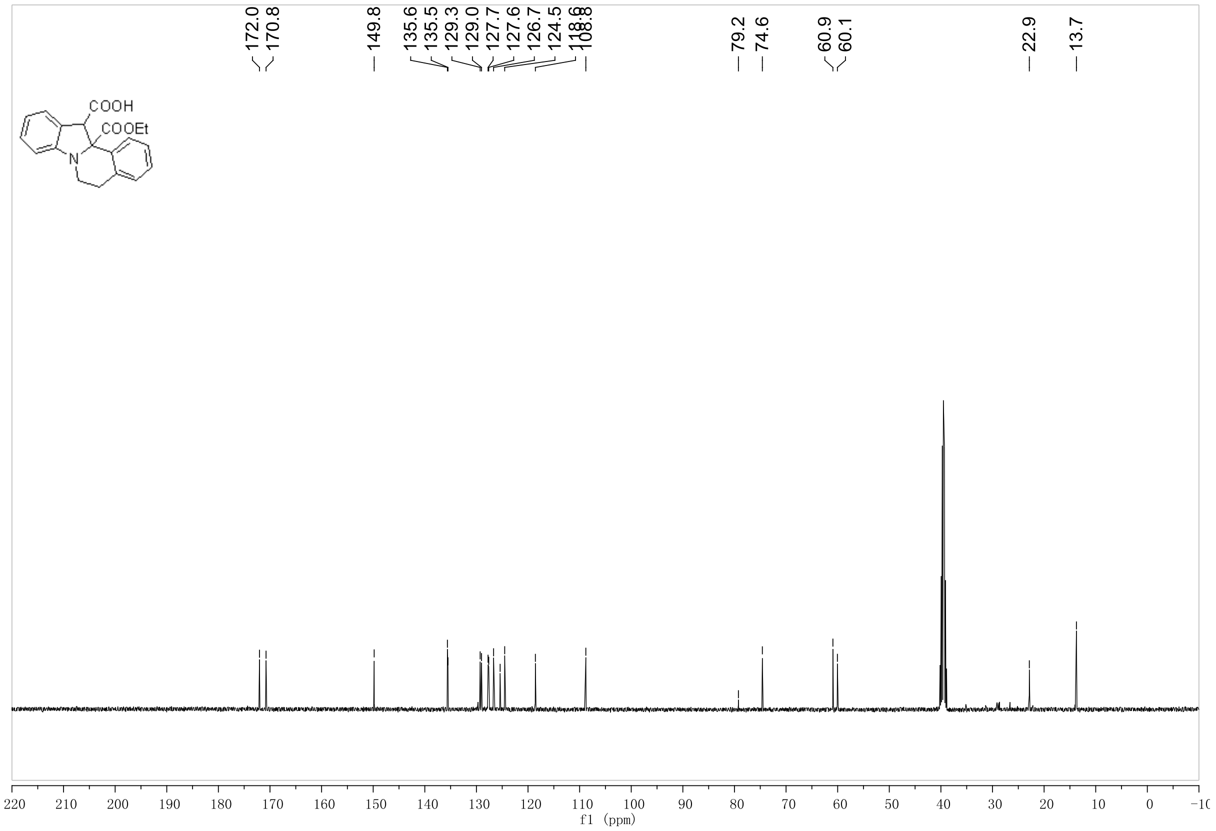

Supplementary Figure 93. ^{13}C NMR Spectra of compound **39**.

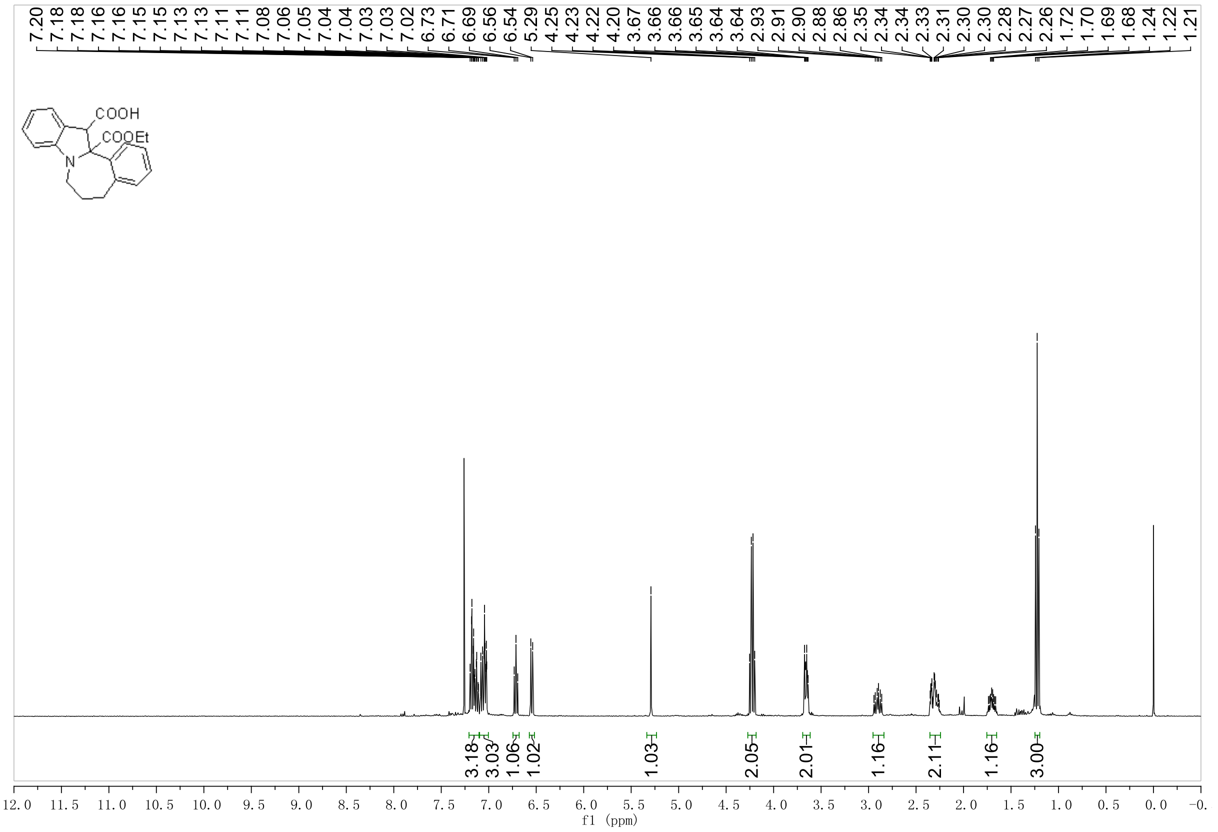

Supplementary Figure 94. ^{1}H NMR Spectra of compound **40**.

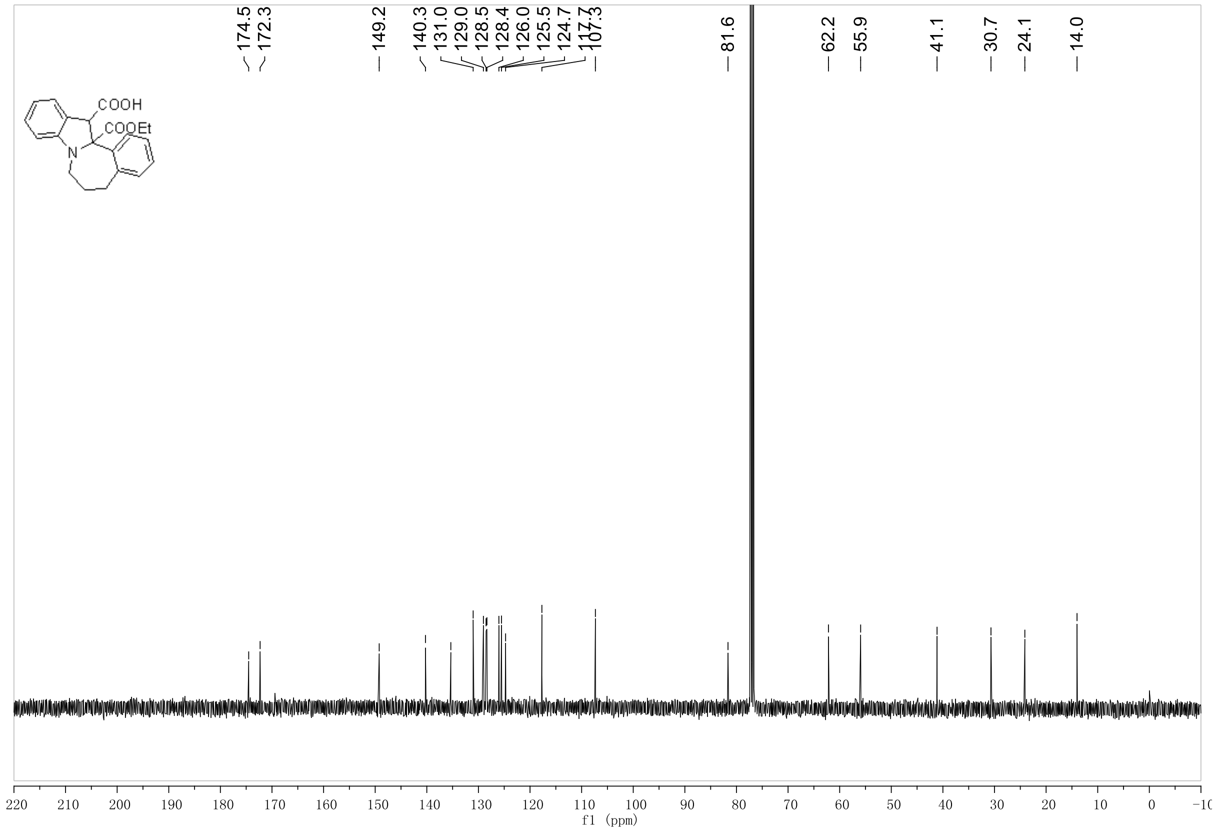

Supplementary Figure 95. ^{13}C NMR Spectra of compound **40**.

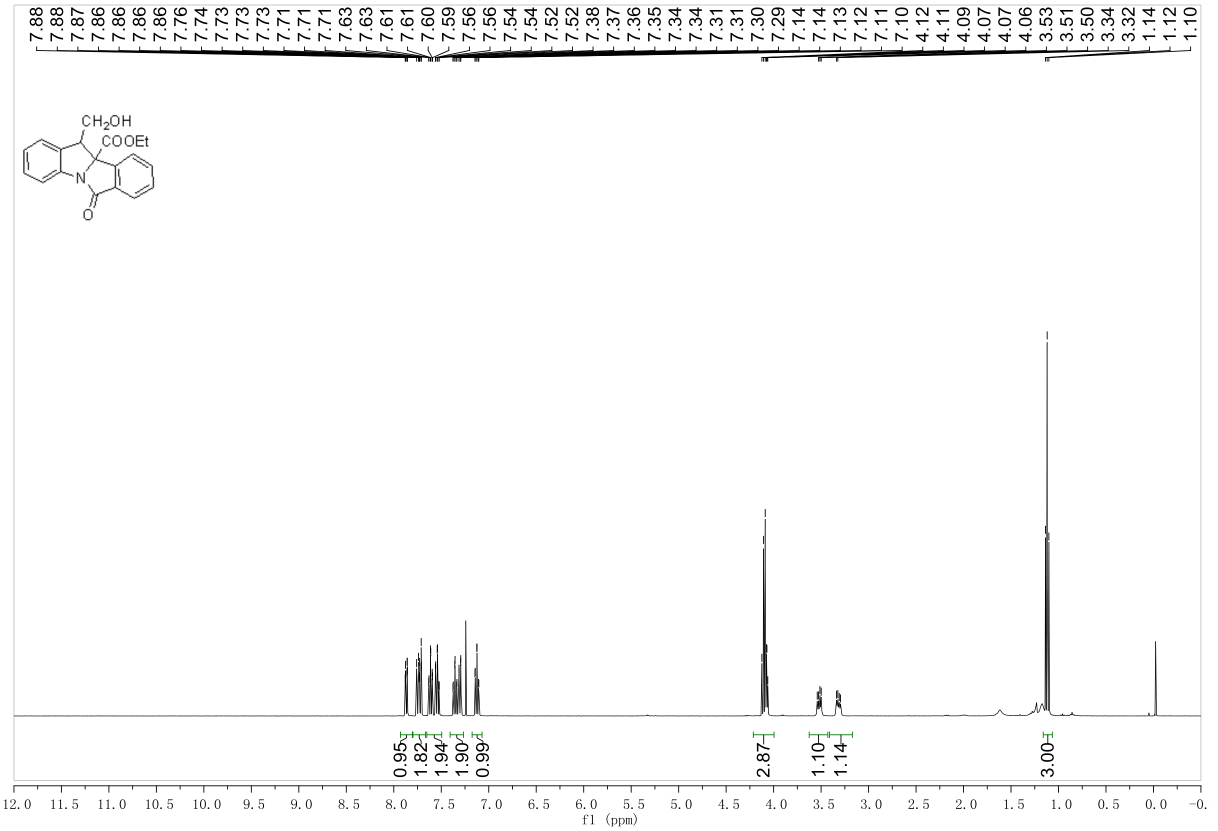

Supplementary Figure 96. ^{1}H NMR Spectra of compound **41**.

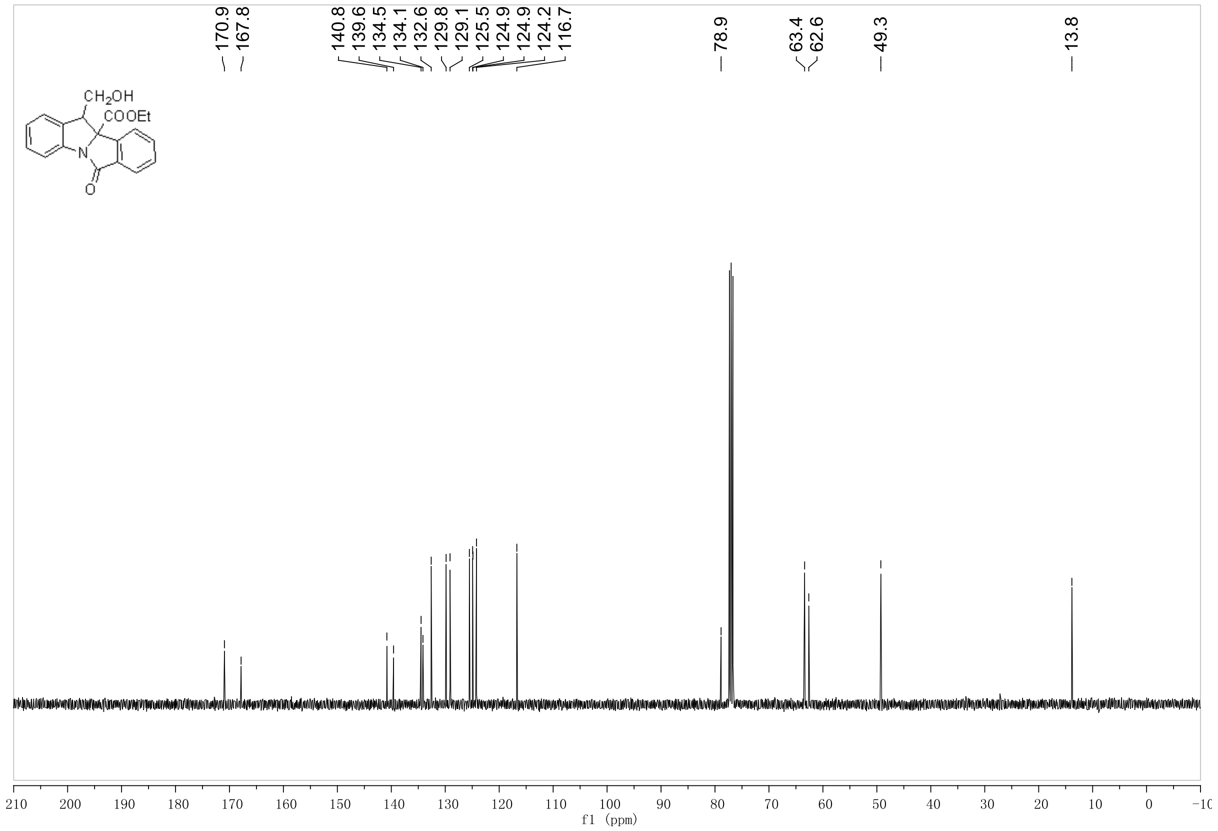

Supplementary Figure 97. ^{13}C NMR Spectra of compound **41**.

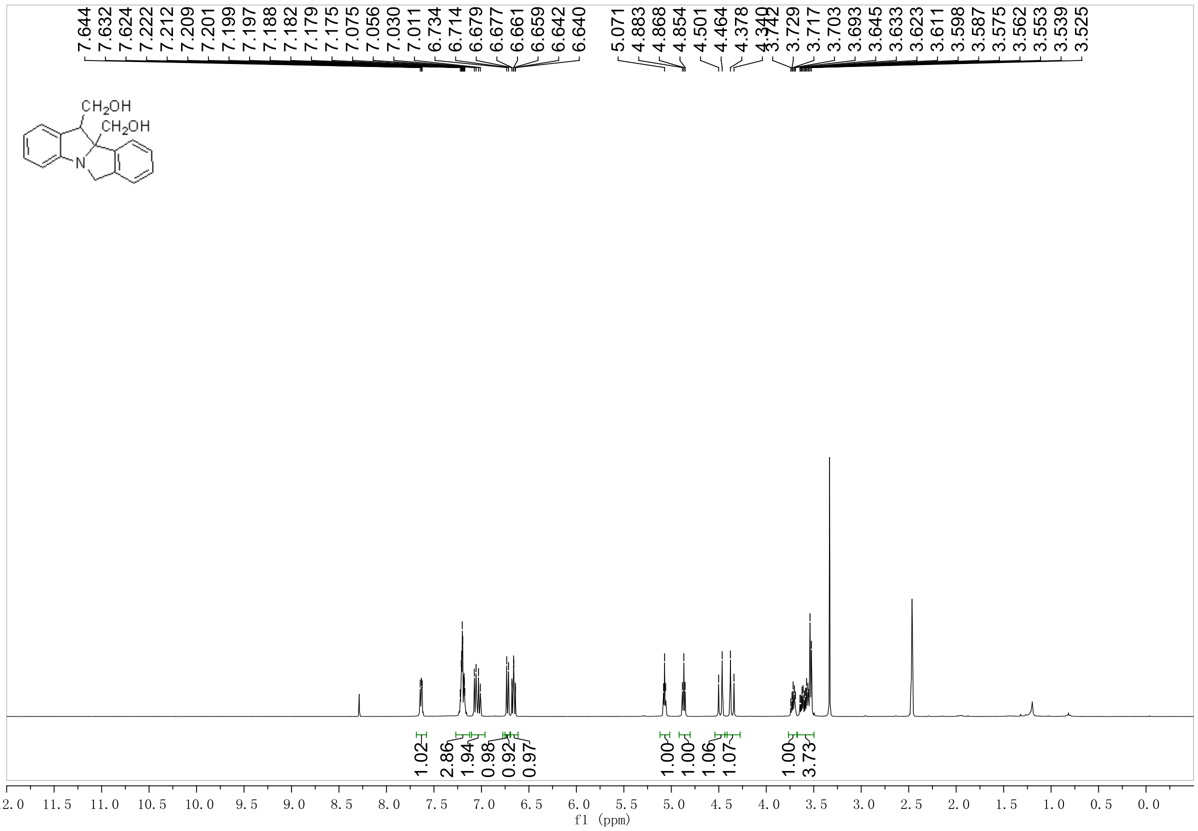

Supplementary Figure 98. ^{1}H NMR Spectra of compound **42**.

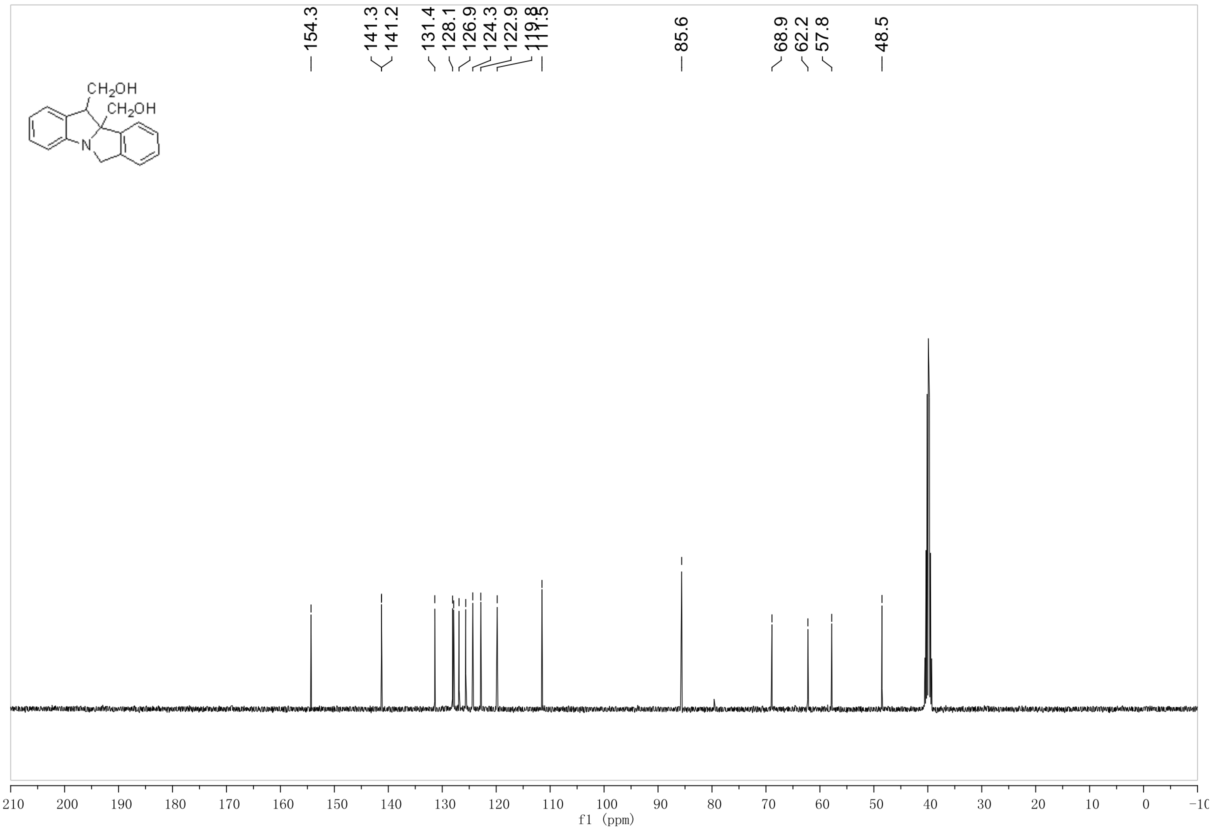

Supplementary Figure 99. ^{13}C NMR Spectra of compound **42**.

X-ray Crystallographic Data of Compound

Compound 2

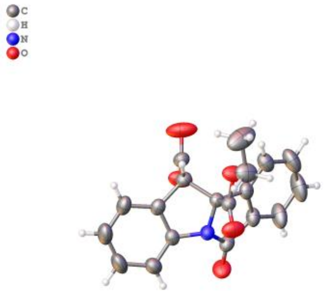

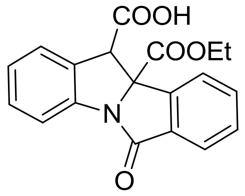

CCDC 1950734

Bond precision:	C-C = 0.0034 A		Wavelength=1.54184	
Cell:	a=22.48722 (19)	b=10.88093 (10)		c=27.2839 (3)
	alpha=90	beta=90		gamma=90
Temperature:	300 K			
	Calculated		Reported	
Volume	6675.88 (11)		6675.88 (11)	
Space group	P b c a		P b c a	
Hall group	-P 2ac 2ab		-P 2ac 2ab	
Moiety formula	$C_{19}H_{15}NO_5$		2 ($C_{19}H_{15}NO_5$)	
Sum formula	$C_{19}H_{15}NO_5$		$C_{38}H_{30}N_2O_{10}$	

Mr	337.32	674.64
Dx,g cm^{-3}	1.342	1.342
Z	16	8
Mu (mm^{-1})	0.817	0.817
F000	2816.0	2816.0
F000’	2825.55	
h, k, lmax	26, 12, 32	26, 12, 32
Nref	5957	5954
Tmin,Tmax	0.721, 0.745	0.650, 1.000
Tmin’	0.654	

Correction method= # Reported T Limits: Tmin= 0.650 Tmax= 1.000

AbsCorr = MULTI-SCAN

Data completeness= 0.999	Theta(max)= 67.078
R(reflections)= 0.0649 (4983)	wR2(reflections)= 0.1834 (5954)
S = 1.031	Npar= 455

Displacement ellipsoids are drawn at 30% probability level

X-ray Crystallographic Data of Major Isomer of Compound 31

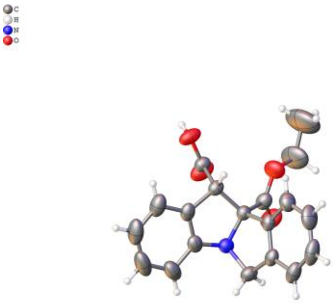

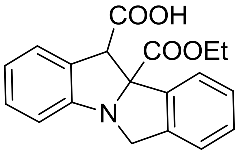

CCDC 1972327

Bond precision:	C-C = 0.0037 A		Wavelength=1.54184
Cell:	a=18.4665 (5)	b=11.13009 (19)	c=19.5242 (5)
	alpha=90	beta=109.979 (3)	gamma=90
Temperature:	292 K		

	Calculated	Reported
Volume	3771.38 (17)	3771.36 (17)
Space group	P 21/n	P 1 21/n 1
Hall group	-P 2yn	-P 2yn
Moiety formula	$C_{19}H_{17}NO_4$ [+ solvent]	2 ($C_{19}H_{17}NO_4$)
Sum formula	$C_{19}H_{17}NO_4$ [+ solvent]	$C_{38}H_{34}N_2O_8$
Mr	323.34	646.67
Dx,g cm^{-3}	1.139	1.139

Z	8	4
Mu (mm^{-1})	0.659	0.659
F000	1360.0	1360.0
F000’	1364.39	
h, k, lmax	22, 13, 24	22, 13, 24
Nref	7373	7220
Tmin,Tmax	0.662, 0.821	0.560, 1.000
Tmin’	0.600	
Correction method= # Reported T Limits: Tmin= 0.560 Tmax= 1.000		
AbsCorr = MULTI-SCAN		
Data completeness= 0.979	Theta(max)= 71.606	
R(reflections)= 0.0763 (5544)	wR2(reflections)= 0.2409 (7220)	
S = 1.032	Npar= 437	
Displacement ellipsoids are drawn at 30% probability level		

Supplementary References

1. Marchese, A. D., Lind, F., Mahon, Á. E., Yoon, H., & Lautens, M. Forming Benzylic Iodides via a Nickel Catalyzed Diastereoselective Dearomative Carboiodination Reaction of Indoles. *Angew. Chem. Int. Ed*. **58**, 5095-5099 (2019).

2. Petrone, D. A., Yen, A., Zeidan, N., & Lautens, M. Dearomative Indole Bisfunctionalization via a Diastereoselective Palladium-Catalyzed Arylcyanation. *Org. Lett*. **17**, 4838-4841 (2015)

3. Barbero, N., SanMartin, R., & Dom ńguez, E. Divergent synthesis of isoindolo[2,1-*a*]indole and indolo[1,2-*a*]indole through copper-catalysed C- and N-arylations. *Tetrahedron Lett*. **50**, 2129-2131 (2009).

4. Qin, X., Lee, M. W. Y., & Zhou J. S. Nickel ‐ Catalyzed Asymmetric Reductive Heck Cyclization of Aryl Halides to Afford Indolines. *Angew. Chem. Int. Ed*. **56**, 12723-12726 (2017).

5. Zimmer, R., Collas, M., Czerwonka, R., Hain, U., & Reissig, H. U. A New Access to Pyrrolizidine Derivatives: Ring Contraction of Methyl (E)-[1,2-Oxazin-3-yl]propenoates. *Synthesis* **2008**, 237-244 (2008).

6. Franchi, P., et al. Combining Magnetic Resonance Spectroscopies, Mass Spectrometry, and Molecular Dynamics:Investigation of Chiral Recognition by 2,6-di-O-Methyl-β-cyclodextrin. *J. Am. Chem. Soc.* **126**, 4343-4354 (2004).

7. Sayes, M., Benoit, G., & Charette, A. B. Borocyclopropanation of Styrenes Mediated by UV ‐ light Under Continuous Flow Conditions. *Angew. Chem. Int. Ed*. **57**, 13514-13518 (2018).

8. Luo, J., & Zhang, J. Donor–Acceptor Fluorophores for Visible-Light-Promoted Organic Synthesis: Photoredox/Ni Dual Catalytic C(sp^3)–C(sp^2) Cross-Coupling. *ACS Catal*. **6**, 873-877 (2016).
